# Association between B-cell depletion and attack risk in neuromyelitis optica spectrum disorder: An exploratory analysis from N-MOmentum, a double-blind, randomised, placebo-controlled, multicentre phase 2/3 trial

**DOI:** 10.1016/j.ebiom.2022.104321

**Published:** 2022-11-10

**Authors:** Jeffrey L. Bennett, Orhan Aktas, William A. Rees, Michael A. Smith, Michele Gunsior, Li Yan, Dewei She, Daniel Cimbora, Sean J. Pittock, Brian G. Weinshenker, Friedemann Paul, Romain Marignier, Dean Wingerchuk, Gary Cutter, Ari Green, Hans-Peter Hartung, Ho Jin Kim, Kazuo Fujihara, Michael Levy, Eliezer Katz, Bruce A.C. Cree, N-MOmentum study investigators

**Affiliations:** a University of Colorado School of Medicine, Anschutz Medical Campus, University of Colorado, Aurora, CO, USA; b Medical Faculty, Heinrich Heine University Düsseldorf, Düsseldorf, Germany; c Horizon Therapeutics plc, Gaithersburg, MD, USA; d Mayo Clinic and Center for MS and Autoimmune Neurology, Rochester, MN, USA; e Mayo Clinic, Rochester, MN, USA; f Experimental and Clinical Research Center, Max Delbrück Center for Molecular Medicine and Charité – Universitätsmedizin Berlin, Corporate Member of Freie Universität Berlin and Humboldt-Universität zu Berlin, Berlin, Germany; g Service de Neurologie, Sclérose en Plaques, Pathologies de la Myéline et Neuroinflammation, Hôpital Neurologique Pierre Wertheimer, Hospices Civils de Lyon, Lyon, France; h Department of Neurology, Mayo Clinic, Scottsdale, AZ, USA; i University of Alabama at Birmingham, Birmingham, AL, USA; j UCSF Weill Institute for Neurosciences, Department of Neurology and Department of Ophthalmology, University of California San Francisco, San Francisco, CA, USA; k Brain and Mind Centre, University of Sydney, Sydney, NSW, Australia; l Department of Neurology, Medical University Vienna, Vienna, Austria; m Department of Neurology, Palacky University in Olomouc, Olomouc, Czech Republic; n Department of Neurology, Research Institute and Hospital of National Cancer Center, Goyang, South Korea; o Department of Multiple Sclerosis Therapeutics, Fukushima Medical University and Multiple Sclerosis and Neuromyelitis Optica Center, Southern Tohoku Research Institute for Neuroscience, Koriyama, Japan; p Department of Neurology, Massachusetts General Hospital and Harvard Medical School, Boston, MA, USA; q UCSF Weill Institute for Neurosciences, Department of Neurology, University of California San Francisco, San Francisco, CA, USA

**Keywords:** Aquaporin-4 antibody-positive neuromyelitis optica spectrum disorder, Devic disease, Anti-CD19 monoclonal antibody, B-cell suppression

## Abstract

**Background:**

Inebilizumab is an anti-CD19 antibody approved for the treatment of neuromyelitis optica spectrum disorder (NMOSD) in adults with aquaporin-4 autoantibodies. The relationship between B-cell, plasma-cell (PC), and immunoglobulin depletion with longitudinal reductions in NMOSD activity after inebilizumab treatment was characterised *post hoc* in an exploratory analysis from the N-MOmentum study (NCT02200770).

**Methods:**

Peripheral blood CD20+ B cells, PC gene signature, and immunoglobulin levels were assessed throughout N-MOmentum (follow-up ≥2.5 years); correlations with clinical metrics and magnetic resonance imaging (MRI) lesion activity were assessed.

**Findings:**

Inebilizumab induced durable B-cell and PC depletion within 1 week versus placebo. Although no association was observed between B-cell counts at time of attack and NMOSD activity, depth of B-cell depletion after the first dosing period correlated with clinical outcomes. All participants receiving inebilizumab demonstrated a robust long-term therapeutic response, and participants with ≤4 cells/μL after the first 6-month dosing interval had persistently deeper B-cell depletion, lower annualised attack rates (estimated rate [95% CI]: 0.034 [0.024–0.04] vs 0.086 [0.056–0.12]; *p* = 0.045), fewer new/enlarging T2 MRI lesions (0.49 [0.43–0.56] vs 1.36 [1.12–1.61]; *p* < 0.0001), and a trend towards decreased Expanded Disability Status Scale worsening (0.076 [0.06–0.10] vs 0.14 [0.10–0.18]; *p* = 0.093). Antibodies to inebilizumab, although present in a proportion of treated participants, did not alter outcomes.

**Interpretation:**

This analysis suggests that compared with placebo, inebilizumab can provide specific, rapid, and durable depletion of B cells in participants with NMOSD. Although deep and persistent CD20+ B-cell depletion correlates with long-term clinical stability, early, deep B-cell depletion correlates with improved disease activity metrics in the first 2 years.

**Funding:**

Horizon Therapeutics (formerly from Viela Bio/MedImmune).

**Research in context**

**Evidence before this study**

A search from inception to 15 September 2021 was conducted in PubMed with the search string “(((neuromyelitis optica spectrum disorder[Title]) OR (NMO[Title]) OR (NMOSD[Title])) AND ((B cell) OR (CD19) OR (CD20)) AND (depletion))” using no date or language filters but excluding records relating to SARS-CoV-2 or COVID-19. The search returned 22 articles, which were further screened for relevance, focusing the search on B-cell-depleting therapies. Of 22 records, 11 were dismissed because they were either reviews or case studies, and three were dismissed because of limited relevance.

In total, eight publications describing investigations on the effect of B-cell-depleting therapies in neuromyelitis optica spectrum disorder (NMOSD) were identified. From these, two reported results from N-MOmentum, the randomised, placebo-controlled study of inebilizumab (anti-CD19); five were uncontrolled, prospective, retrospective, or real-world studies on the use of rituximab (anti-CD20); and one was a pilot, open-label, phase 1b study of ublituximab (anti-CD20) in five participants. Notably, neither rituximab nor ublituximab is approved for the treatment of NMOSD.

Rituximab has been used as an off-label treatment based on low-level evidence because of the previous lack of proven alternatives. We found six studies (four retrospective and two prospective) in which CD19+ B cells in peripheral blood were monitored in an attempt to guide re-infusion of anti-CD20 therapy; however, the correlations between B-cell numbers and clinical activity metrics with continuous therapy were not evaluated in larger cohorts.

**Added value of this study**

Since 2019, three different therapies have been approved for NMOSD; however, inebilizumab is the only one that results in targeted B-cell depletion.

The pivotal phase 2/3 N-MOmentum trial demonstrated that inebilizumab treatment in NMOSD was associated with significant improvements in clinical outcomes compared with placebo. The current investigation of possible associations between depth of B-cell depletion and long-term clinical outcomes in N-MOmentum with over 2.5 years of follow-up provides evidence that, while all participants benefitted from treatment, greater suppression of attack activity is found with deep depletion of B cells to 4 cells/μL or below at the end of the first dosing period. The study also shows that all participants treated with inebilizumab reached deep and sustained B-cell depletion with multiple doses, and all had significant long-term improvements in NMOSD clinical outcomes, irrespective of whether they had deep B-cell depletion after the first inebilizumab dose.

**Implications of the available evidence**

Characterising the relationship between clinical outcomes and the pharmacodynamic effects of inebilizumab on B cells may support monitoring of B-cell levels in patients with NMOSD treated with inebilizumab. It remains to be investigated whether patients showing partial depletion and/or early reconstitution of B cells may achieve deeper or more sustained B-cell depletion with dose adjustments or more frequent administration.

## Introduction

Neuromyelitis optica spectrum disorder (NMOSD) is a rare, autoimmune disease of the central nervous system (CNS) in which approximately 75–90% of patients harbour pathogenic immunoglobulin (Ig) G autoantibodies against the aquaporin-4 water channel (AQP4-IgG).^1^ Recurrent, severe attacks of optic neuritis and myelitis are typical manifestations of the disease, although brain and brainstem lesions also occur.^2^^,^^3^ B cells play a prominent role in NMOSD pathogenesis, acting through several mechanisms. First, B-cell maturation leads to the generation of plasmablasts and plasma cells producing pathogenic AQP4-IgG.^4^ Second, B-cell secretion of pro-inflammatory cytokines, such as interleukin (IL)-6, may stimulate pathogenic, pro-inflammatory immune responses. Third, B cells may act as antigen-presenting cells to promote the development and activation of autoimmune T cells.^2^^,^^4^ Last, B-cell depletion and reconstitution restore B regulatory cell numbers and function.^5^

Monoclonal antibodies can induce B-cell depletion through several mechanisms, including complement-dependent cytotoxicity,^6^ apoptosis,^7^^,^^8^ and antibody-dependent cell cytotoxicity (ADCC).^9^ Anti-CD20 antibodies historically were used as therapeutic agents for NMOSD^10^^,^^11^; however, B-cell depletion with anti-CD19 monoclonal antibodies offers an attractive treatment approach in autoantibody-driven autoimmune diseases because the pattern of CD19 expression on B cells is broader than that of CD20.^12^ Unlike CD20, CD19 is expressed on plasmablasts as well as on a proportion of long-lived plasma cells and antibody-secreting cells in secondary lymphoid organs and bone marrow (Supplementary Figure S1).^12^ Furthermore, CD19 is expressed earlier in the B-cell lineage than CD20^13^^,^^14^; thus, anti-CD19 therapy may be more effective in blunting the emergence of autoimmune populations.^2^^,^^15^

Inebilizumab is a humanised, affinity-optimised, anti-CD19 monoclonal antibody^12^ that depletes a wide range of B cells in preclinical studies^12^ and phase 1 clinical trials in systemic sclerosis and relapsing multiple sclerosis.^16^^,^^17^ Inebilizumab is approved in China, the European Union, Japan, South Korea, and the USA for treatment of NMOSD in adult AQP4-IgG-seropositive patients.^18^ The efficacy and safety profile of inebilizumab-induced B-cell depletion, including plasmablasts and some plasma cells, was assessed in the randomised, double-masked, phase 2/3 N-MOmentum study in NMOSD. In N-MOmentum, inebilizumab reduced the risk of an adjudicated NMOSD attack by 77.3% compared with placebo in AQP4-IgG-seropositive participants.^19^ Here, we report exploratory, *post hoc* analyses that investigated whether the impact of inebilizumab on NMOSD activity, including clinical attacks, disability worsening, CNS magnetic resonance imaging (MRI) lesions, and NMOSD-related hospitalisations, was associated with the extent of peripheral blood CD19+ B-cell depletion.

## Methods

### Study design and participants

The N-MOmentum study was an international, multicentre, double-blind, randomised, placebo-controlled, phase 2/3 trial with an open-label extension (ClinicalTrials.gov identifier: NCT02200770).^19^ Adults (aged ≥18 years) with NMOSD were eligible for inclusion if they had an Expanded Disability Status Scale (EDSS) score of 8.0 or less, and a history of at least one attack requiring rescue therapy in the previous year, or of at least two attacks requiring rescue therapy in the previous 2 years. AQP4-IgG serostatus was determined centrally using the Euroimmune cell-based assay as described.^19^ Patients who were AQP4-IgG seropositive and those who were AQP4-IgG seronegative according to the 2006 neuromyelitis optica diagnostic criteria were eligible.^20^ Further study details can be found in the Supplementary Methods; full study details were published previously.^19^ Both the study protocol and statistical analysis plan are available at ClinicalTrials.gov: https://clinicaltrials.gov/ct2/show/NCT02200770.

### Randomisation

Eligible participants were randomly allocated (3:1) to intravenous inebilizumab 300 mg or placebo, administered on days 1 and 15 of the randomised controlled period (RCP) (Supplementary Figure S2). A central interactive voice system and interactive web response system, and a permuted block randomisation scheme were used to assign participants randomly to a subject identification number and treatment group, and to provide a masked investigational product kit number.^19^ The subject identification number was used to identify the participant throughout the study. A participant was considered randomised into the study after the investigator had notified the central system that they met eligibility criteria and the system had assigned them a product kit number.

All participants received oral corticosteroid therapy during the initial treatment period (tapered off at day 21); use of other immunosuppressants was not permitted during the RCP. Participants continued in the RCP for up to 28 weeks or until the occurrence of an adjudicated attack, after which they had the option to enter an open-label period (OLP) of at least 2 years. Furthermore, the RCP was halted early on the recommendation of the data-monitoring committee, owing to the clear evidence of efficacy. Thus, participants who were still in the RCP were eligible to enter the OLP immediately. All participants received inebilizumab on day 1 of the OLP, and then every 6 months for the duration of their enrolment in the OLP. Participants randomised to placebo in the RCP received an additional dose of inebilizumab 300 mg on day 15 of the OLP.

### Masking

All study participants, investigators, treating and evaluating staff, the adjudication committee (AC), and the sponsor remained masked during treatment and throughout the study. Placebo and inebilizumab were identical in appearance. Participants randomised to inebilizumab in the RCP received a placebo dose on day 15 of OLP to maintain masking.

### Outcomes

Data were captured in a validated electronic system, and processed (including cleaning, reconciling, and quality control processing) and stored in a controlled access environment. Clinical data were analysed according to the approved statistical plan, available at https://clinicaltrials.gov/ProvidedDocs/70/NCT02200770/SAP_001.pdf.

The primary efficacy endpoint was the time to onset of an adjudicated NMOSD attack during the RCP. An attack was defined as the presence of one or more new symptoms, or worsening of one or more existing symptoms related to NMOSD, that met at least one of the 18 protocol-defined neurological examination criteria for an attack. These criteria comprised myelitis, optic neuritis, and the brain/brainstem domain symptoms.^21^ The study investigator evaluated all potential attacks within 72 hours. An independent committee of three expert physicians (two neurologists and one neuro-ophthalmologist) adjudicated the attacks in the 17 days after the assessment visit. NMOSD activity outcomes included the annualised attack rate (AAR), EDSS worsening,^22^ cumulative new/enlarging T2 MRI lesions, and NMOSD-related inpatient hospitalisations. Systematic MRI imaging of all relevant CNS domains (spinal cord, optic nerve, and brain/brainstem) were performed on all participants at screening, at the end of the RCP (week 28, ±7 days), at the time of attack assessment (in the 5 days after initiation of attack assessment), and every 52 weeks (±7 days) during the OLP.

The study included an adaptive design element in that a futility analysis was conducted by the independent data monitoring committee when approximately 50% of the planned AC-adjudicated NMOSD attacks had occurred (34 AC-adjudicated attacks). The trial was considered futile if the predictive power was <20%. Because the futility analysis considers only failure, and not success, assessing futility is a one-sided test (conditional power) and does not affect Type I error (falsely claiming success when there is none); therefore, no estimate for bias on outcomes caused by the futility analysis was performed.

### Evaluation of B-cell pharmacodynamics

During the RCP, blood samples were collected during study visits at baseline and weeks 1, 2, 4, 8, 12, 16, 22, and 28 to assess B-cell counts; at baseline and weeks 2, 4, 8, 12, 16, and 28 to evaluate plasma-cell-specific gene expression; and at weeks 12 and 28 to evaluate Ig levels. During the OLP, blood samples were collected every 13 weeks for assessment of B-cell counts, plasma-cell-specific gene expression, and Ig levels. Data on T-cell subsets were not collected. Blood samples to assess B-cell counts and plasma-cell-specific gene expression were also collected during any assessment visit for new or worsening NMOSD symptoms during the RCP and OLP. Samples for attack assessments were taken before initiation of treatment for NMOSD attacks.

B-cell levels in peripheral blood were measured by flow cytometry using fluorescence-activated cell sorting (FACS), conducted at a central laboratory in the 72 hours after sample collection. Whole blood samples were collected in Streck Cyto-chex cell preservative tubes (Streck, La Vista, NE, USA) and shipped to the central laboratory on the day of collection. A minimum of 100,000 lymphocyte events were acquired. B-lineage cells were counted using CD20 as a FACS marker (bound inebilizumab interferes with CD19-based FACS). The B-cell subsets that were measured included CD20^+^ B cells, defined as CD45^hi^ [CD3−, CD14–, CD56−], CD33−, and CD20+. In addition, an analysis of plasmablast/plasma cell counts (CD45^hi^ [CD3−, CD14−, CD56−], CD27+, HLA-DR^hi/low^, and CD38+) was performed on a subset of participants using FACS. The lower limit of quantification (LLOQ) for all cell populations was set at 0.2 cells/μL. Samples with cell counts below the LLOQ for any given cell population were imputed to 0.05 cells/μL. Further details on the reagents used for flow cytometry, including Research Resource Identifier tags, can be found in Supplementary Table S1.

Plasma-cell-specific gene expression was assessed by quantitative reverse transcription polymerase chain reaction of blood RNA samples. The plasma-cell gene signature was based on expression analysis of four genes (*IGHA1*, *IGJ*, *IGKV4-1*, and *TNFRSF17*) that are expressed predominantly by plasma cells in blood.^23^ The signature was calculated as the average expression of the four plasma-cell-specific genes minus the average expression of five control genes (*B2M*, *GAPDH*, *TFRC*, *GUSB*, and *UBC*) at each time point. The fold-change in plasma-cell gene expression signature at each time point was calculated relative to a pool of 10 healthy donor samples and interpreted as plasma-cell abundance relative to expected prevalence under a non-activated immune state.

TaqMan single nucleotide polymorphism profiling was used to investigate possible associations between the rs396991 polymorphism in the *FCGR3A* gene and impaired B-cell depletion in a subgroup of participants who provided consent for genomic analysis. The rs396991 polymorphism is widespread and encodes a valine/phenylalanine substitution at position 158 of FCGR3A that is associated with decreased binding affinity for Ig Fc,^24^^,^^25^ thus impairing ADCC mechanisms for which the Fc region of inebilizumab has been optimised.

### Statistics

The analyses presented in this article are exploratory and *p* values are provided for hypothesis generation only. Descriptive statistics were calculated for changes in B-cell subset counts over time. The statistical analyses were conducted in R 4.1.3. Differences in B-cell counts, total Ig levels, and plasma-cell signatures were assessed using the Mann–Whitney *U* test. Relationships between depletion groups and treatment outcomes for summarisation were estimated by negative binomial regression (details are included in the Supplement) or Poisson regression when appropriate, and included an offset term to account for the varying intervals of time for which participants were included in the RCP. Glm.nb from MASS package in R.4.1.3 was used to fit the model. Differences in gene expression, including *FCGR3A* F/F genotype between depletion groups, were assessed using Fisher's exact test. The Benjamini–Hochberg method was used to adjust the false discovery rate to <10% when predictors of elevated B-cell counts at the end of the first dosing interval were investigated. B-cell counts at week 28 of the RCP were designated ‘6-month B-cell counts’ for most participants randomised to inebilizumab during the RCP. Among participants randomised to placebo during the RCP, B-cell counts at week 26 of the OLP were used as the ‘6-month B-cell counts’. For inebilizumab-treated participants who did not complete week 28 of the RCP, either owing to experiencing an adjudicated attack or discontinuing the RCP early because of evidence of efficacy, week 26 OLP B-cell counts were included as ‘6-month B-cell counts’ for regression analysis.

To characterize the relationship between B-cell concentrations and future NMOSD activity further, sensitivity analyses were performed for different cut-off points and time points. A B-cell count cut-off point was explored using negative binomial regression analyses of week 26 to week 28 B-cell counts against the post-6-month AAR, rate of new/enlarging T2 MRI lesions, rate of EDSS worsening, and rate of NMOSD-related inpatient hospitalisations.

Missing data were accounted for by assuming to be missing completely at random. Given that there was a small proportion of missing data at each time point, imputation is not expected to have had a significant effect on the observed results.^26^ Details on sample size calculations performed for the original endpoint can be found in the Supplementary Methods, and further information about the core statistical design for N-MOmentum, including power calculations, was published previously.^19^ N-MOmentum was designed to detect a relative reduction due to inebilizumab of 60% in time to NMOSD attack during the RCP with at least 90% power and a two-sided significance level of 5%, assuming an unequal randomisation ratio of 3:1 (inebilizumab: placebo) and that participants randomised to placebo would only receive placebo for a max of 197 days. The inclusion of 212 participants was estimated to result in 67 adjudicated attacks.

### Ethics

All participants provided written, informed consent. Ethics committees or institutional review boards at each study site approved the protocol (Supplementary Table S2). The study was conducted in accordance with the provisions of the International Conference on Harmonisation Good Clinical Practice Guidelines and the principles of the Declaration of Helsinki in its currently applicable version. Further details about ethical considerations were published previously.^19^

### Role of funders

The N-MOmentum trial was funded by MedImmune and Viela Bio (now part of Horizon Therapeutics). Horizon Therapeutics supported the development of this manuscript, provided data analyses according to the direction of the authors, and paid for medical writing support, provided by Oxford PharmaGenesis, Oxford, UK. Medical writing and editorial support were provided by Dr Ester Baixauli and Grant Womack of Oxford PharmaGenesis, according to the direction of the authors, and funded by Horizon Therapeutics.

## Results

### Study participants

**Fig. 1:**
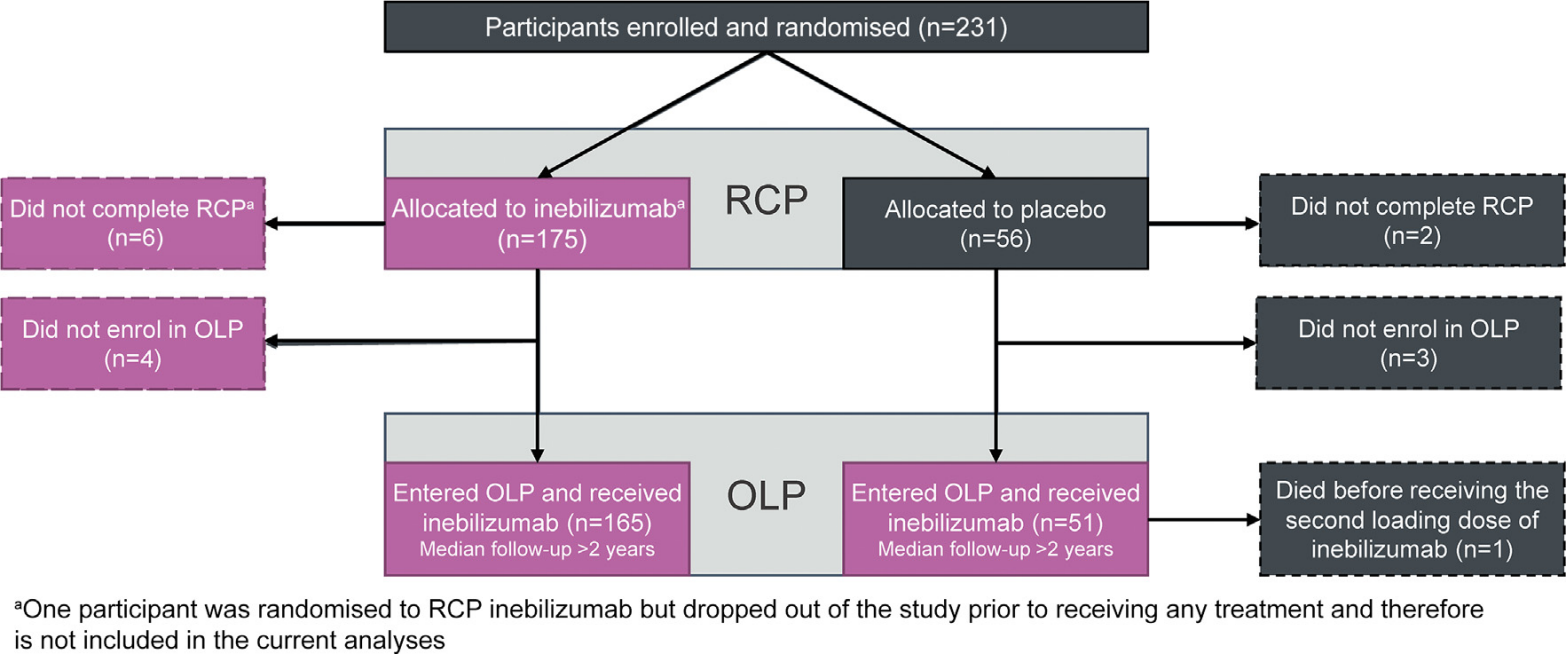
**Participant flow in N-MOmentum.** For analysis purposes, participants were divided into three groups: “RCP inebilizumab” (participants randomised to inebilizumab during the RCP), “RCP placebo” (participants randomised to placebo during the RCP), and “any inebilizumab” (participants who received inebilizumab at any point during the study, including those from the RCP inebilizumab group and all participants in the OLP; shaded in pink). OLP = open-label period, RCP = randomised controlled period.

In N-MOmentum, 230 randomised participants received study treatment (RCP: inebilizumab, n = 174; placebo, n = 56; “any inebilizumab”, n = 225; Fig. 1). Most participants in the inebilizumab group were women (91%), approximately half were white (53%), and most were AQP4-IgG seropositive (93%). Participant characteristics were similar in the two treatment groups (Table 1).^19^ Full details on baseline demographics and the primary outcome and secondary outcomes of the study were reported.^19^

**Table 1:** Baseline demographics and characteristics.

Demographic/characteristic	Overall ITT population (n = 230)	
	Placebo (n = 56)	Inebilizumab (n = 174)
Age		
Mean (SD), years	42.6 (13.9)	43.0 (11.6)
Median (range)	42.5 (18–74)	43.0 (18–73)
Sex		
Women	50 (89.3)	159 (91.4)
Race^a^		
American Indian or Alaskan Native	5 (8.9)	14 (8.0)
Asian	8 (14.3)	39 (22.4)
Black or African American	5 (8.9)	15 (8.6)
White	28 (50.0)	92 (52.9)
Other	10 (17.9)	13 (7.5)
Multiple categories checked	0 (0.0)	1 (0.6)
Ethnicity		
Hispanic or Latino	15 (26.8)	28 (16.1)
Disease duration, years		
Mean (SD)	2.77 (3.45)	2.41 (3.30)
Median (range)	1.38 (0.2–16.9)	1.06 (0.1–22.2)
Type of most recent attack		
Optic neuritis	21 (37.5)	85 (48.9)
Myelitis	34 (60.7)	99 (56.9)
Brain/brainstem	10 (17.9)	8 (4.6)
Prior treatment		
Any therapy^b^	55 (98.2)	172 (98.9)
Plasmapheresis	27 (48.2)	67 (38.5)
Intravenous immunoglobulin	3 (5.4)	8 (4.6)
Any prior maintenance therapy	38 (67.9)	114 (65.5)
Corticosteroids	23 (41.1)	79 (45.4)
Non-biologic immunosuppression^c^	26 (46.4)	79 (45.4)
Azathioprine	22 (39.3)	63 (36.2)
Mycophenolate mofetil	7 (12.5)	26 (14.9)
Methotrexate	0 (0.0)	2 (1.1)
Biologic agent	5 (8.9)	25 (14.4)
Rituximab	4 (7.1)	13 (7.5)
Interferon beta	1 (1.8)	7 (4.0)
Natalizumab	0 (0.0)	2 (1.1)
Glatiramer acetate	0 (0.0)	2 (1.1)
No prior maintenance therapy	18 (32.1)	60 (34.5)
Baseline Gd-enhancing lesions		
Mean (SD)	0.9 (0.9)	1.2 (1.2)
Median (range)	1.0 (0.0–4.0)	1.0 (0.0–5.0)
Baseline EDSS score		
Mean (SD)	4.19 (1.68)	3.81 (1.81)
Median (range)	4.0 (1.0–8.0)	3.5 (0.0–8.0)

EDSS = Expanded Disability Status Scale, Gd = gadolinium, ITT = intention-to-treat, SD = standard deviation.

Data are n (%) unless stated otherwise.

a Race was self-reported by patients; ‘Other’ included Mestizo (n = 12), Mixed (n = 6), Arab, Hispanic, Vietnamese, Caucasian/Latino, and New Zealand Maori (all n = 1).

b Includes any prior treatment for neuromyelitis optica spectrum disorder, including rescue and maintenance therapy; some patients received more than one maintenance therapy.

c All other non-biologic, non-corticosteroid treatments including azathioprine, cetirizine, cyclophosphamide, cyclosporine, fingolimod, methotrexate, mitoxantrone, mizoribine, mycophenolate mofetil, or pentoxifylline.

### Inebilizumab treatment rapidly depletes B cells and maintains treatment efficacy

**Fig. 2:**
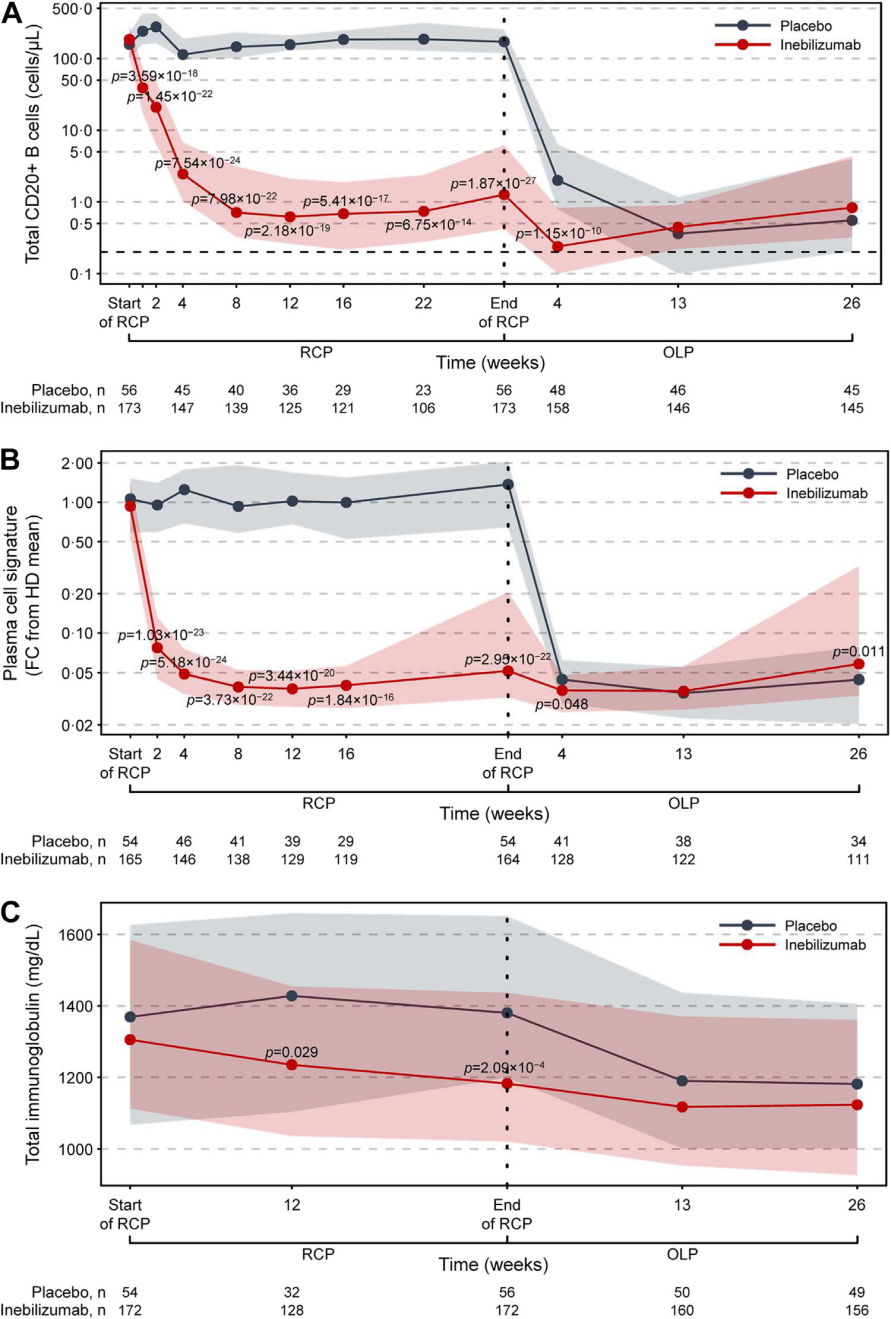

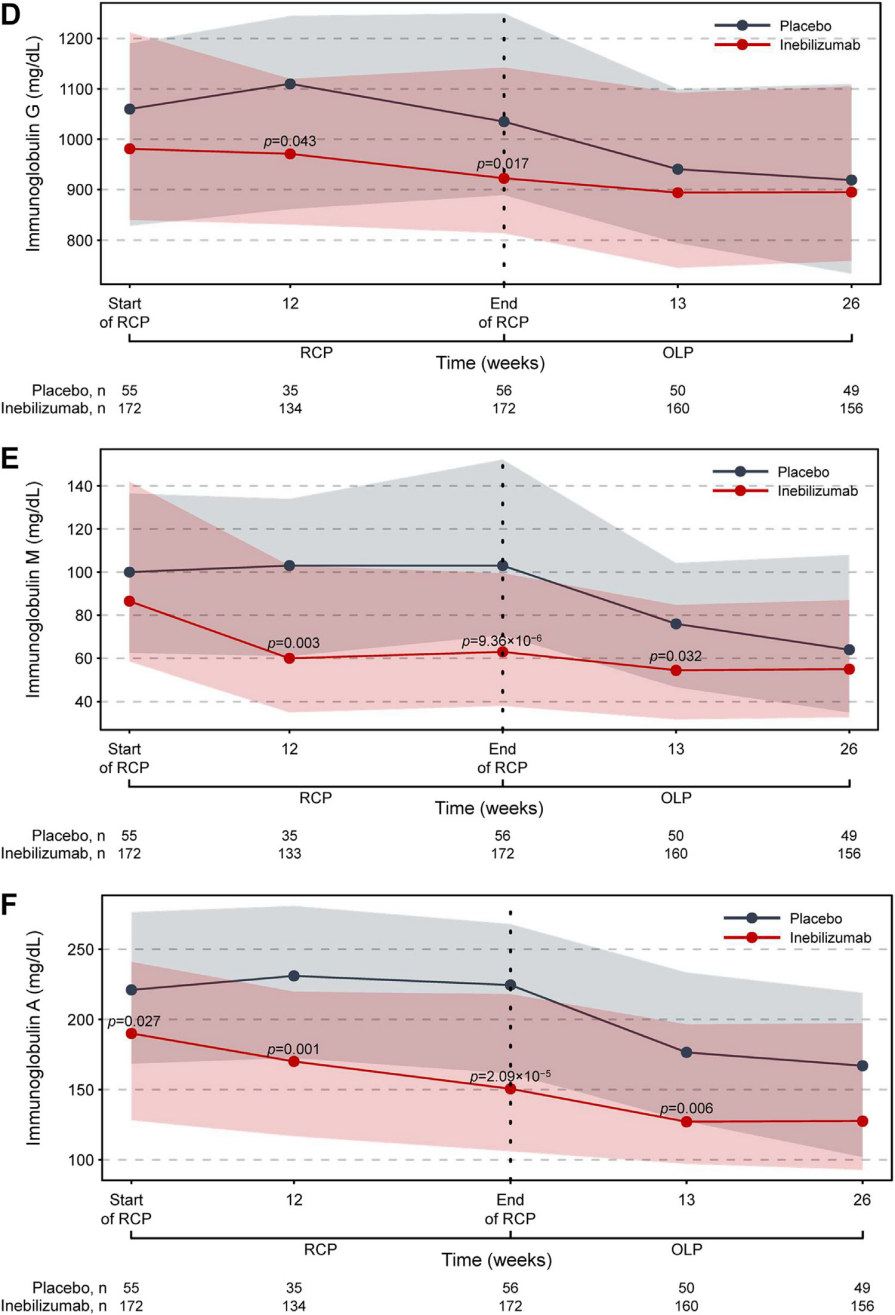

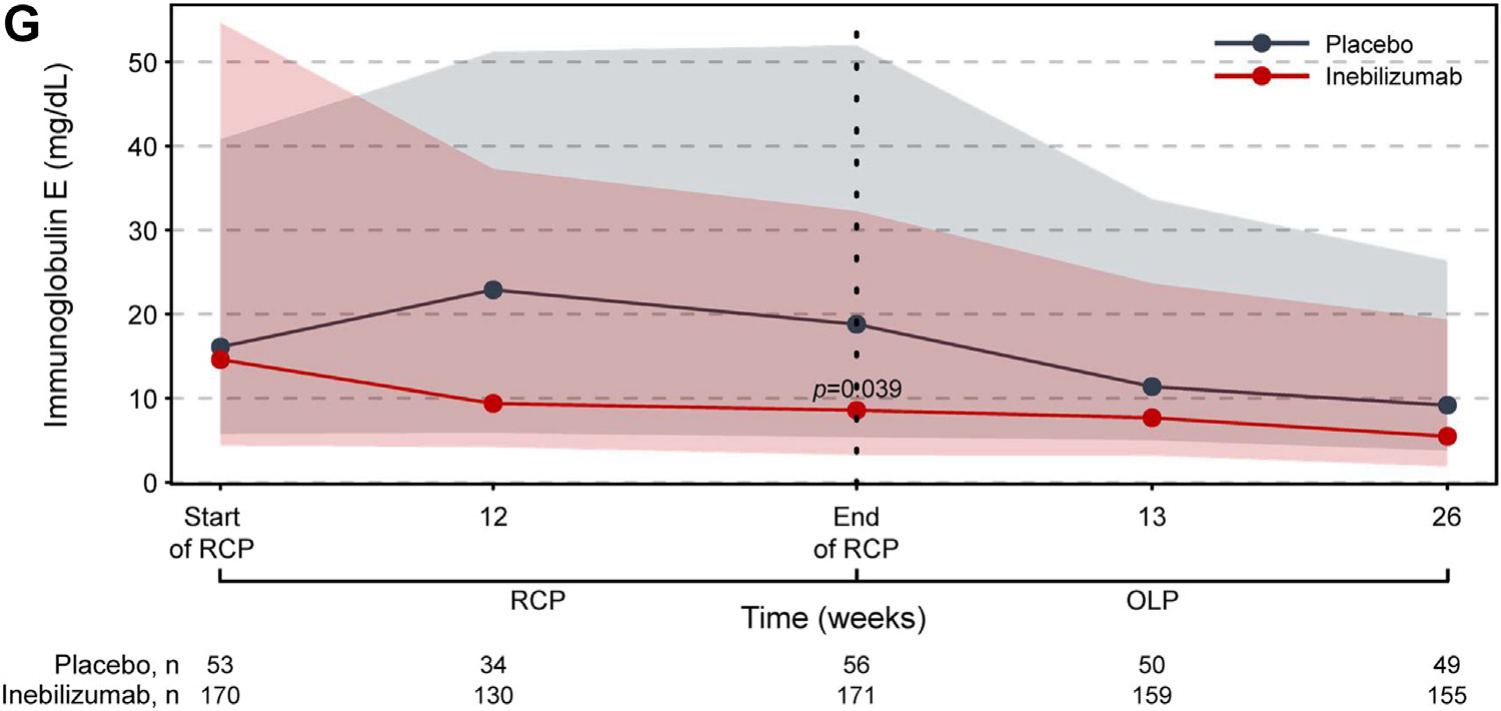
**Kinetics of B-cell and immunoglobulin depletion during the study. (a)** Total CD20+ B-cell counts. **(b)** Plasma-cell gene signature normalised to a pool of 10 HDs using the ddCT method. **(c)** Total immunoglobulin, **(d)** immunoglobulin G, **(e)** immunoglobulin M, **(f)** immunoglobulin A, and **(g)** immunoglobulin E concentrations over time during the RCP in placebo-treated (in grey) and inebilizumab-treated (in red) participants. Points show medians and shaded regions show IQRs. Dashed black lines show LLOQ of 0.2 cells/μL for FACS assays. Samples with counts below the LLOQ were imputed to 0.05 cells/μL. Mann–Whitney *U* test between dose groups; *p* > 0.05 where *p* not noted. Vertical dashed lines show when participants were transitioned to the OLP and the placebo cohort was administered two inebilizumab doses; the inebilizumab cohort was given an additional maintenance dose. ddCT = delta delta cycle threshold, FACS = fluorescence-activated cell sorting, FC = fold change, HD = healthy donor, IQR = interquartile range, LLOQ = lower limit of quantification, OLP = open-label period, RCP = randomised controlled period.

**Fig. 3:**
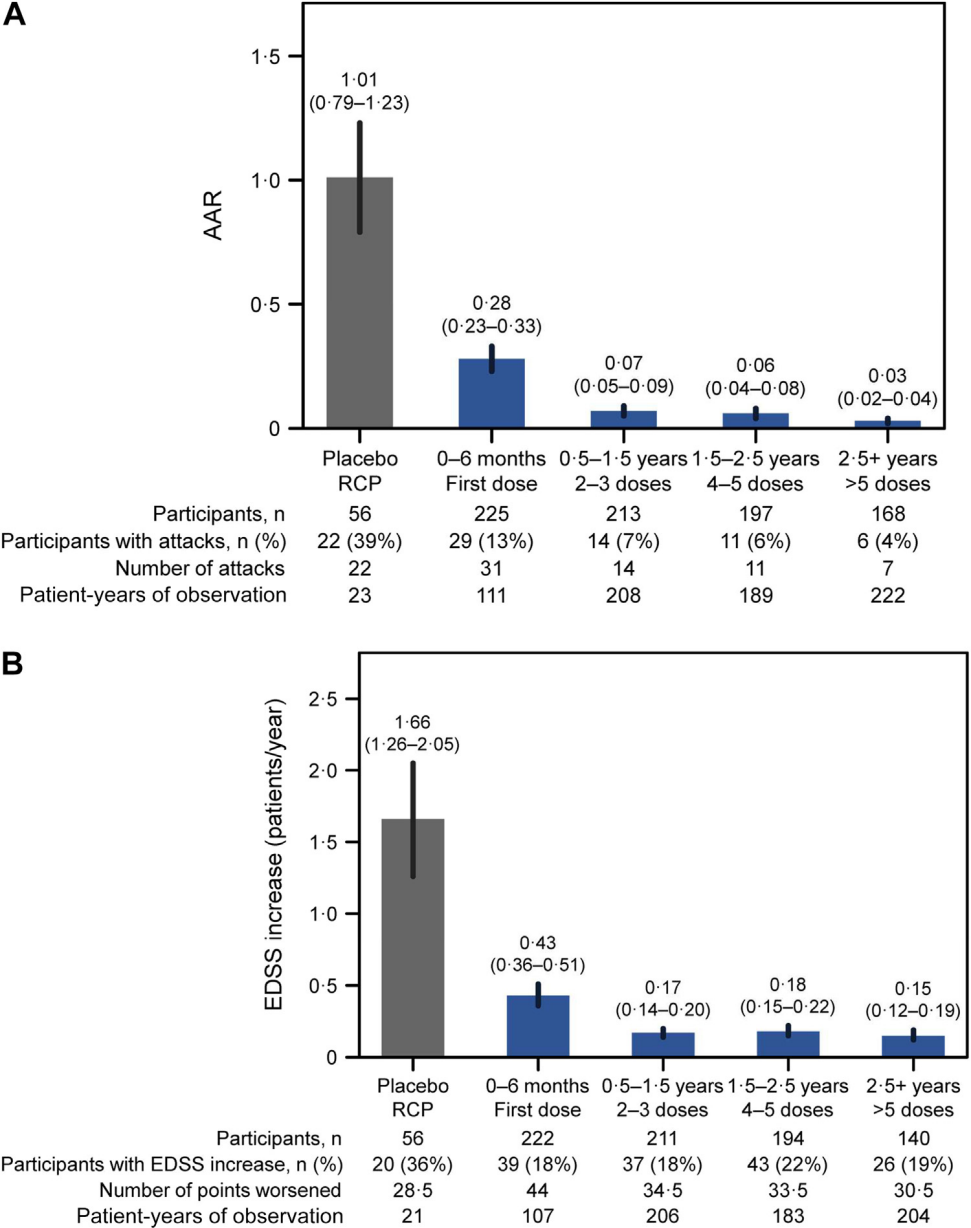

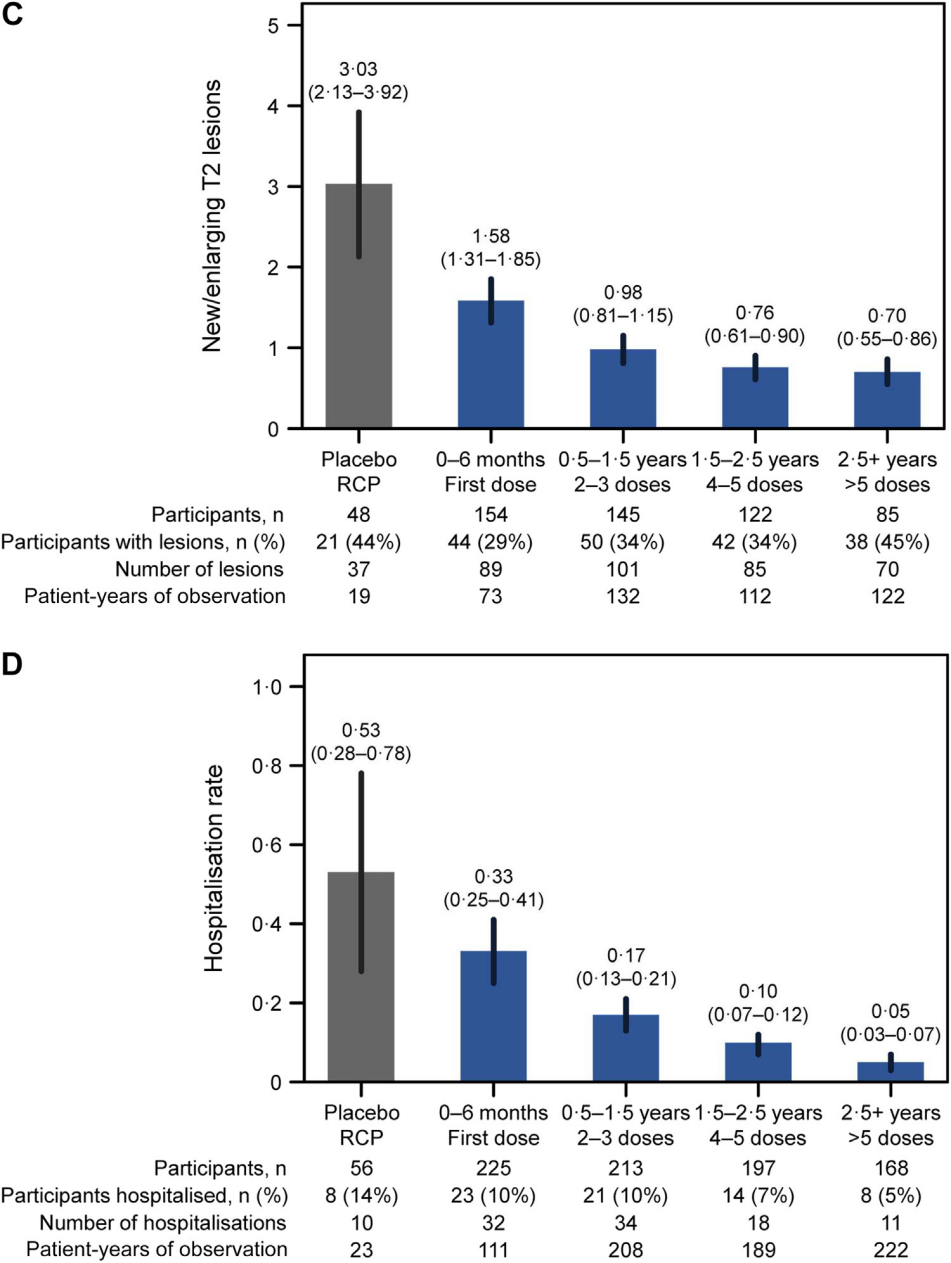
**Decreases in NMOSD progression observed with long-term inebilizumab treatment.(a)** AAR. **(b)** Annualised rate of EDSS worsening. **(c)** Annualised rate of new/enlarging MRI T2 lesions (only participants randomised to inebilizumab are included). **(d)** Annualised NMOSD-related inpatient hospitalisation rate. Plots show rates in placebo-treated participants during RCP (in grey) and in all participants during the first dosing period of inebilizumab treatment (first dosing interval), and yearly afterwards (in blue). The final bar in each plot displays the combined rate of each endpoint after 2.5 years or more of continued inebilizumab treatment. Mean data with error bars showing 95% CI estimated by negative binomial regression. AAR = annualised attack rate, CI = confidence interval, EDSS = Expanded Disability Status Scale, MRI = magnetic resonance imaging, NMOSD = neuromyelitis optica spectrum disorder, RCP = randomised controlled period.

Inebilizumab treatment significantly reduced circulating levels of CD20+ B cells and the plasma-cell gene signature versus placebo during the RCP (Fig. 2; Supplementary Table S3). Total Ig levels were also decreased, with the greatest reductions seen in IgE, IgA, and IgM classes. Overall, NMOSD activity decreased significantly with inebilizumab treatment during the RCP^19^ and continued to decline with further doses of inebilizumab (Fig. 3; Supplementary Table S4). After 2.5 years of inebilizumab treatment, progressive reductions in AAR, annualised rate of new/enlarging T2 MRI lesions, EDSS worsening, and NMOSD-related inpatient hospitalisations were observed.

### Deep B-cell depletion at 6 months is associated with lower long-term NMOSD activity

**Fig. 4:**
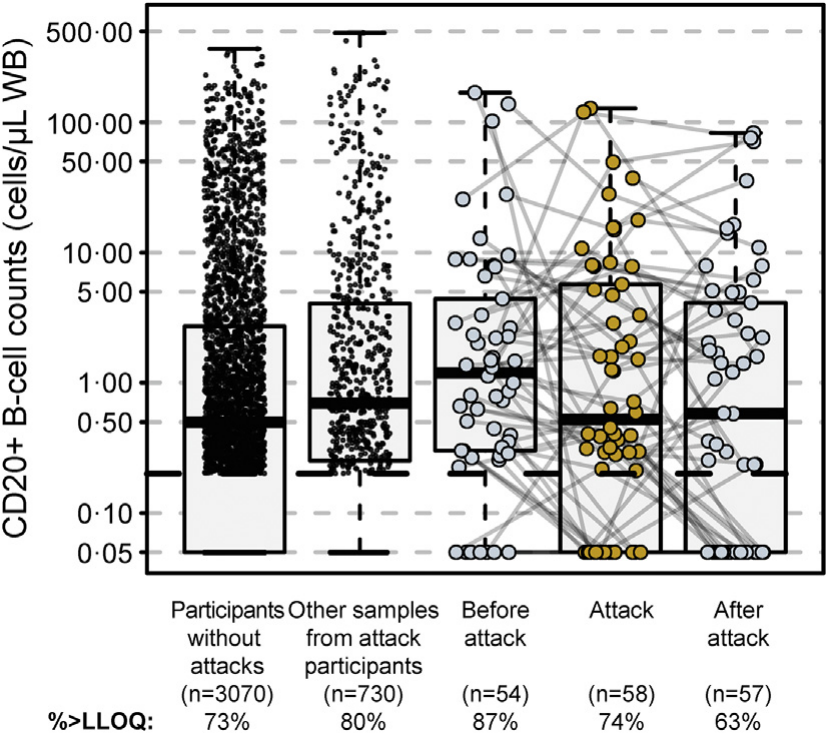
**Temporal relationship between CD20+ B-cell counts and attacks.** Box plot of CD20+ B-cell counts in all samples drawn from participants who never experienced attacks, in samples drawn preceding attacks (sample draws 15–115 days preceding attacks), during attack assessment, and after attacks (sample draws 8–200 days after attack). “Other samples” refers to samples that were drawn from participants who experienced attacks, but not directly preceding the attack, directly after the attack, or during the attack. The bold bar shows the median, the box shows the IQR, and the whiskers show the range of data. IQR = interquartile range, LLOQ = lower limit of quantification, WB = whole blood.

Given that we observed a progressive reduction in NMOSD activity with prolonged inebilizumab treatment, we hypothesised that the depth of B-cell depletion may correlate with therapeutic efficacy. Peripheral blood CD20+ B-cell counts were examined in participants with and without attacks, through the RCP and OLP, during attack-independent, pre-attack, peri-attack, and post-attack epochs (Fig. 4). Statistically significant increases were not observed in peripheral blood CD20+ B-cell levels at the time of the attack in samples from all participants or from the subgroups of participants receiving three or more doses of inebilizumab (Fig. 4; Supplementary Figure S3). No statistically significant increases in B-cell counts were observed during attack assessments, irrespective of whether the attacks occurred during or after the first dosing period.

Therefore, we investigated the extent of B-cell depletion during the first dosing interval, and the extent to which it might predict the subsequent depth of B-cell depletion and reduction in NMOSD activity. A negative binomial regression analysis revealed a linear association between B-cell counts at the conclusion of the first 6-month dosing interval and disease activity that occurred after the first inebilizumab dosing period (Table 2; Supplementary Table S5). Several additional time points correlated with future disease activity in the regression analysis. Indeed, B-cell counts from samples drawn as early as 3 months after inebilizumab treatment initiation correlated with future NMOSD activity after the first dosing period; however, B-cell counts from these early time points did not correlate with NMOSD activity during the first period of treatment (data not shown).

**Table 2:** 6-month B-cell counts versus long-term disease activity.

	RR (95% CI)	Number of participants	Total patient-years of observation	*p*
Annualised attack rate				
Log_10_ memory B cells	2.06 (1.11−3.81)	170	527	0.022
Log_10_ 6-month CD20+ B cells	1.61 (1.04−2.49)	197	582	0.031
Rate of new/enlarging T2 MRI lesions				
Log_10_ memory B cells	1.93 (1.44−2.59)	145	392	<0.0001
Log_10_ 6-month CD20+ B cells	1.62 (1.32−1.99)	166	432	<0.0001
EDSS worsening rate				
Log_10_ memory B cells	1.56 (0.99−2.47)	169	508	0.058
Log_10_ 6-month CD20+ B cells	1.24 (0.89−1.74)	196	562	0.210
NMOSD-related inpatient hospitalisation rate				
Log_10_ memory B cells	1.87 (1.01–3.45)	170	533	0.047
Log_10_ 6-month CD20+ B cells	1.56 (0.99−2.45)	197	589	0.056

CI = confidence interval, EDSS = Expanded Disability Status Scale, MRI = magnetic resonance imaging, NMOSD = neuromyelitis optica spectrum disorder, OLP = open-label period, RCP = randomised controlled period, RR = rate ratio, W26 = week 26 after treatment initiation, W28 = week 28 after treatment initiation.

Negative binomial regression analysis of B-cell counts at 6 months after treatment (W28 RCP for participants treated with inebilizumab who completed the RCP, W26 OLP for participants randomised to placebo and participants treated with inebilizumab who transitioned into the OLP early) versus the annualised attack rate, rate of new/enlarging T2 MRI lesions, EDSS worsening rate (participants/year), and rate of NMOSD-related inpatient hospitalisations that occurred after the first dosing period of inebilizumab treatment.

**Fig. 5:**
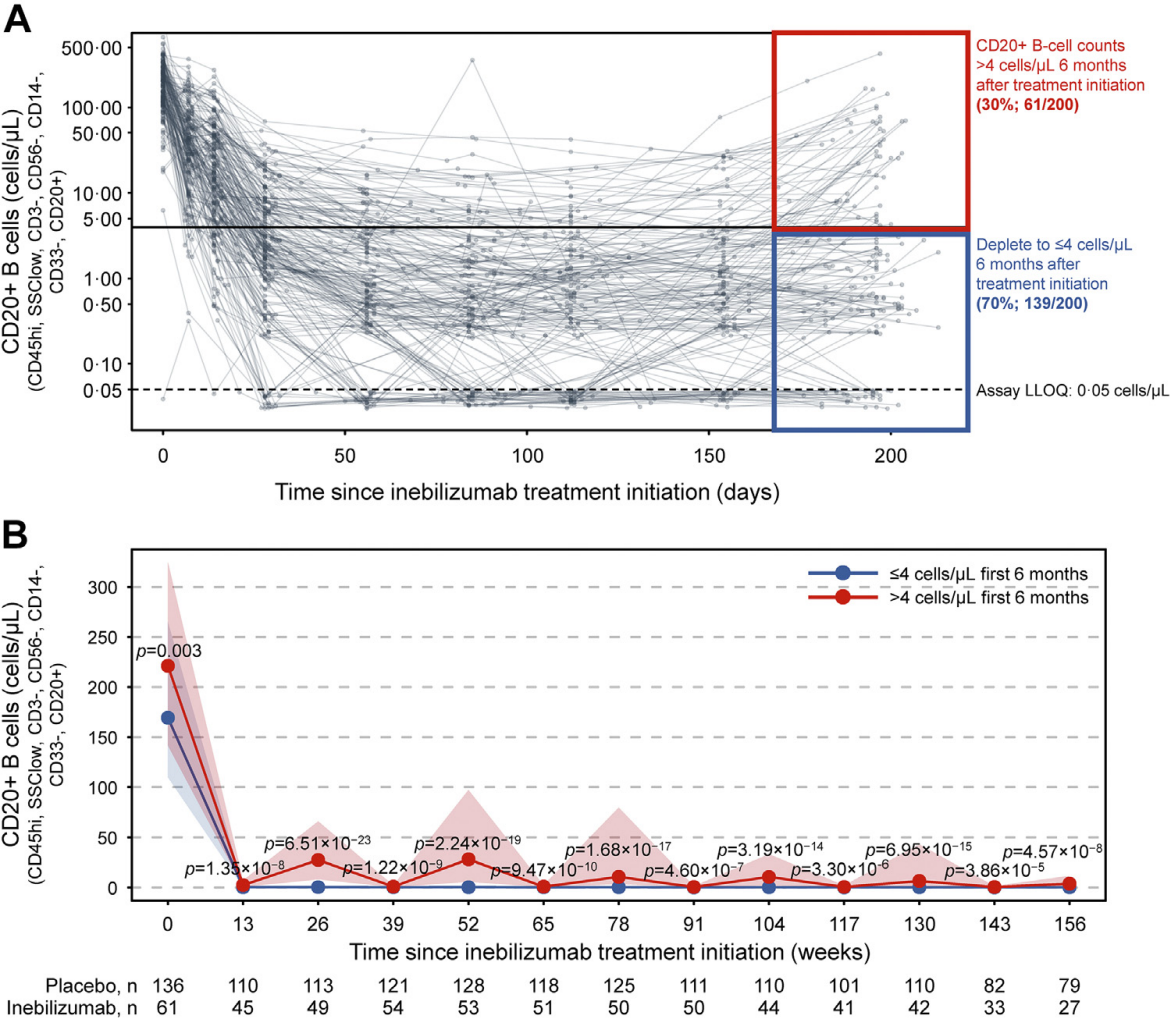
**B-cell kinetics in the first dosing interval of inebilizumab predict long-term deep B-cell depletion. (a)** Profile plot of CD20+ B-cell counts in individual patients throughout the first dosing interval with inebilizumab. **(b)** Total CD20+ B-cell counts over 3 years of inebilizumab treatment in patients with B-cell counts >4 cells/μL and ≤4 cells/μL at the end of the first dosing period. Points show medians ± IQR. Mann–Whitney *U* test was used to calculate *p* values. IQR = interquartile range, LLOQ = lower limit of quantification.

At 6 months (28 weeks) and before the next inebilizumab infusion, a cut-off point for CD20+ B cells of 4 cells/μL separated participants with a decreased risk of NMOSD activity during subsequent inebilizumab dosing. Sensitivity analyses showed that a range of B-cell concentrations maintained a favourable rate ratio across the multiple NMOSD activity metrics, but 4 cells/μL was the highest cut-off by which there appeared to be a sizeable difference in longitudinal outcomes between participant groups (Supplementary Figure S4). In total, 139/200 participants (70%) had B-cell counts ≤4 cells/μL at the end of the first dosing interval, and they maintained durable B-cell depletion with continued treatment (Fig. 5). Compared with participants with B-cell counts >4 cells/μL, those with ≤4 cells/μL at the end of the first dosing interval had persistently lower B-cell counts (Fig. 5b), lower AAR (estimated rate [95% CI]: 0.034 [0.024–0.04] vs 0.086 [0.056–0.12]; *p* = 0.045), fewer new/enlarging T2 MRI lesions (estimated rate [95% CI]: 0.49 [0.43–0.56] vs 1.36 [1.12–1.61]; *p* < 0.0001), and trended towards less EDSS worsening (estimated rate [95% CI]: 0.076 [0.06–0.10] vs 0.14 [0.10–0.18]; *p* = 0.093), and had fewer NMOSD-related inpatient hospitalisations (estimated rate [95% CI]: 0.08 [0.058–0.104] vs 0.18 [0.11–0.25]; *p* = 0.11; Table 3). Similar disease activity outcomes were observed when the AQP4-IgG-seropositive participant population was analysed separately (Table 3; Supplementary Figure S5). Notably, in AQP4-IgG-seropositive participants, a trend towards lower AAR was noted in those with ≤4 cells/μL, but it did not reach statistical significance (*p* = 0.07).

**Table 3:** Long-term outcomes in participants with 6-month B-cell counts ≤4 cells/μL versus >4 cells/μL.

	Rate/year (95% CI) Placebo RCP	Rate/year (95% CI) ≤4 cells/μL after first dosing period	Rate/year (95% CI) >4 cells/μL after first dosing period	RR (95% CI)	*p*
Post-6-month NMOSD attacks	1.01 (0.79–1.23)	0.034 (0.024–0.04)	0.086 (0.056–0.12)	0.40 (0.16–0.98)	0.045
Post-6-month NMOSD attacks, AQP4+ participants only	1.08 (0.85–1.31)	0.038 (0.026–0.052)	0.088 (0.058–0.12)	0.44 (0.18–1.07)	0.07
Post-6-month EDSS worsening, participants/year	1.66 (1.26–2.05)	0.076 (0.06–0.10)	0.14 (0.10–0.18)	0.55 (0.27–1.11)	0.093
Post-6-month EDSS worsening, participants/year; AQP4+ participants only	1.76 (1.34–2.18)	0.07 (0.06–0.09)	0.15 (0.10–0.19)	0.50 (0.24–1.05)	0.07
Post-6-month new/enlarging T2 MRI lesions	3.03 (2.13–3.92)	0.49 (0.43–0.56)	1.36 (1.12–1.61)	0.36 (0.23–0.56)	<0.0001
Post-6-month new T2 MRI lesions, AQP4+ participants only	3.14 (2.27–4.01)	0.51 (0.44–0.58)	1.38 (1.13–1.63)	0.37 (0.24–0.58)	<0.0001
Post-6-month NMOSD-related inpatient hospitalisations	0.53 (0.28–0.78)	0.08 (0.058–0.104)	0.18 (0.11–0.25)	0.45 (0.17–1.18)	0.11
Post-6-month NMOSD-related inpatient hospitalisations, AQP4+ participants only	0.55 (0.27–0.82)	0.08 (0.05–0.10)	0.19 (0.11–0.26)	0.41 (0.15–1.08)	0.07

AQP4+ = seropositive for immunoglobulin G autoantibodies against the aquaporin-4 water channel, CI = confidence interval, EDSS = Expanded Disability Status Scale, MRI = magnetic resonance imaging, NMOSD = neuromyelitis optica spectrum disorder, RCP = randomised controlled period, RR = rate ratio, W26 = week 26 after treatment initiation.

RR (95% CI) versus W26 B-cell cut-off point of attacks, new/enlarging T2 MRI lesions, EDSS worsening, and NMOSD-related inpatient hospitalisations that occurred after the first dosing period. RRs were calculated by comparing the rate/year at which each endpoint occurred in patients with B cells ≤4 cells/μL versus >4 cells/μL after the first dosing period; *p* values are shown for this comparison.

With continued inebilizumab administration, durable B-cell depletion was still observed in the >4 cells/μL subgroup (Fig. 5b) and, by week 117, median CD20+ B-cell counts were similar among participants in both the >4 cells/μL and the ≤4 cells/μL groups.

### Longitudinal NMOSD activity in 6-month B-cell count subgroups

**Fig. 6:**
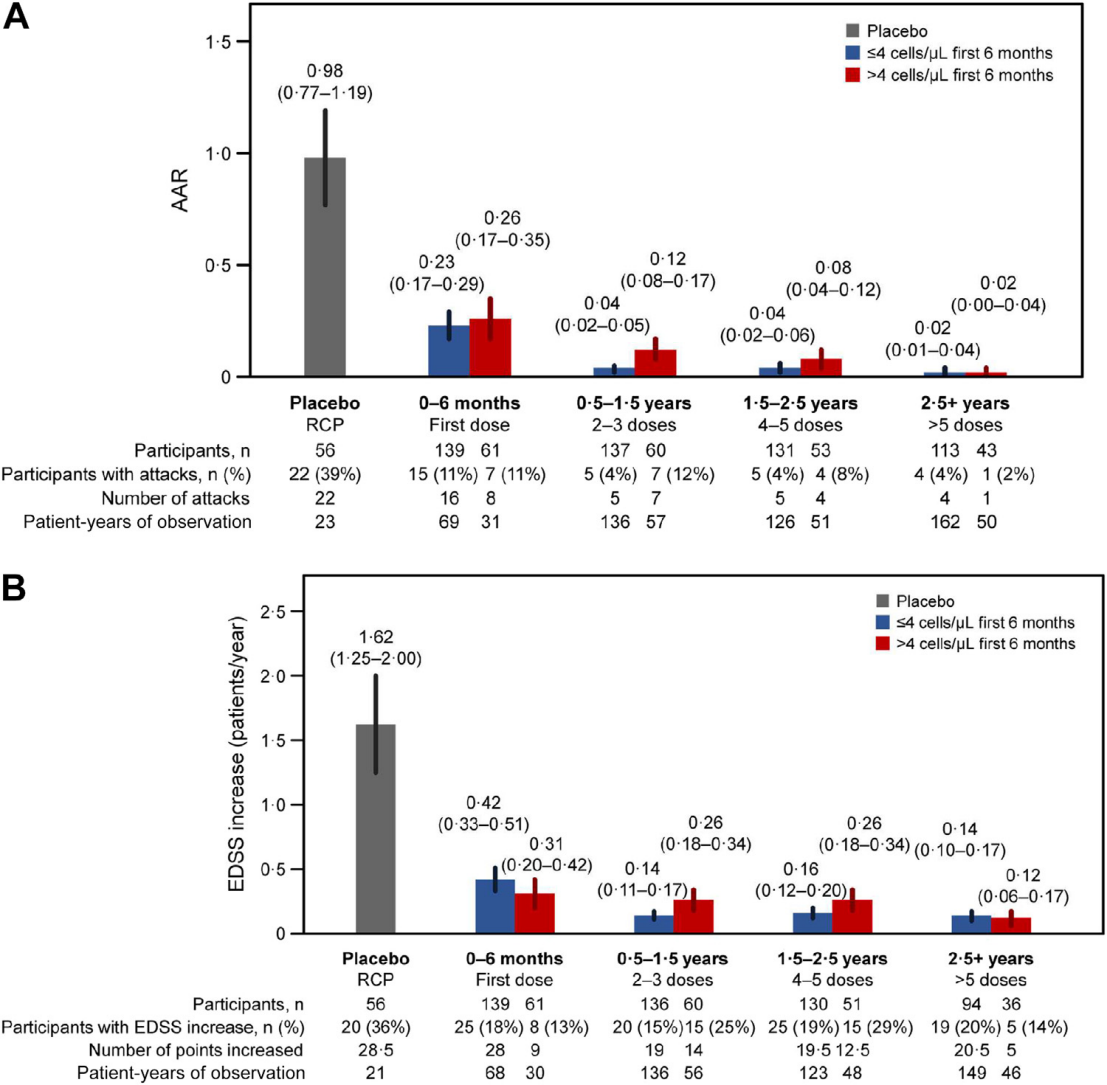

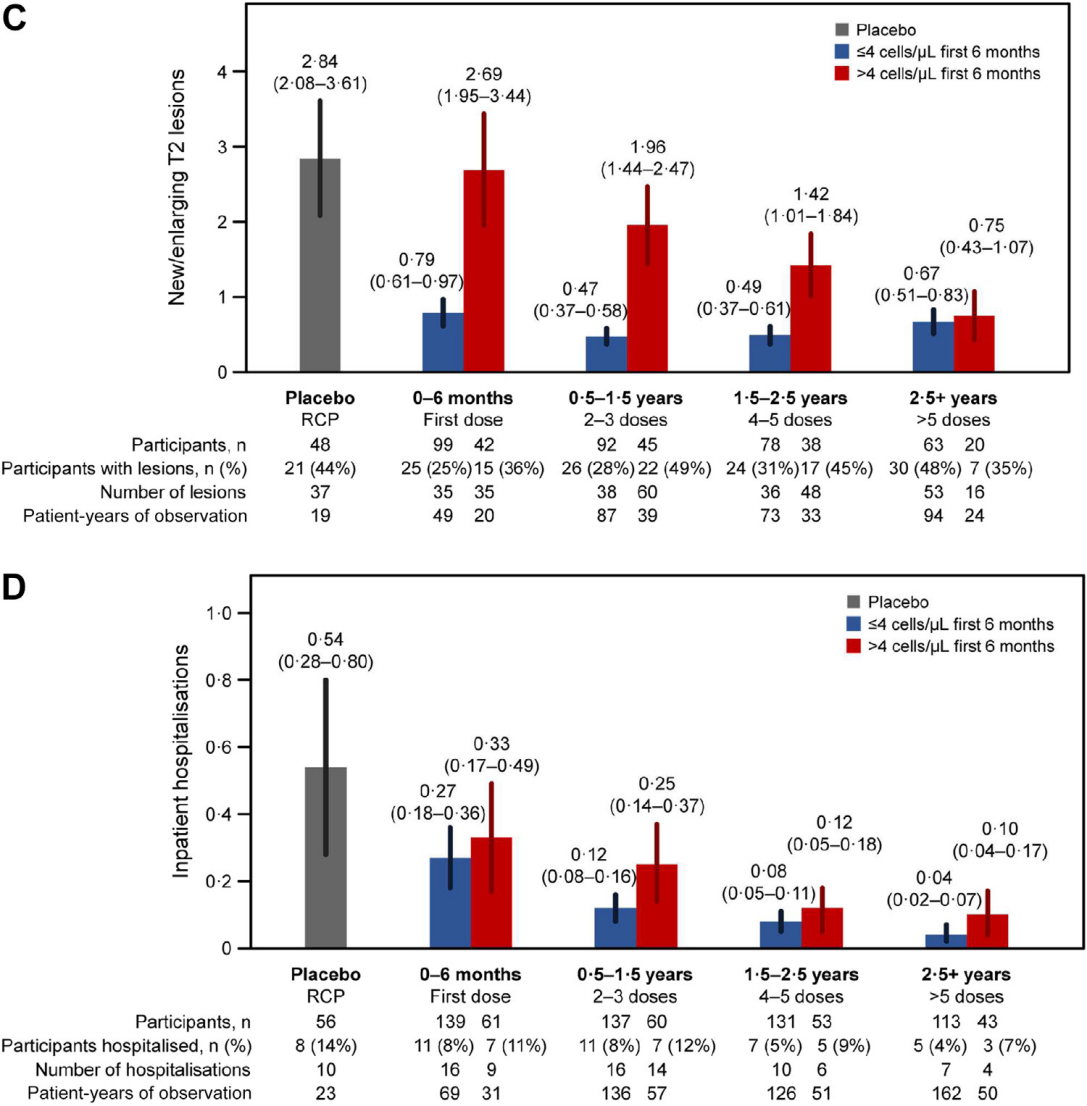
**Improvement occurs in all participant subgroups over time.(a)** AAR. **(b)** Annualised rate of EDSS worsening. **(c)** Annualised rate of new/enlarging T2 MRI lesions (only participants randomised to inebilizumab are included). **(d)** Annualised inpatient hospitalisation rate split by patients with ≤4 cells/μL or >4 cells/μL after the first inebilizumab dosing interval (6 months after treatment). Plot shows rates in placebo-treated participants during the RCP (in grey), then in both inebilizumab-treated participant groups during the first dosing period of inebilizumab treatment (first dosing interval), and yearly afterwards (in blue and red). The final bars in each plot display the combined rate of each endpoint after 2.5 years or more of continued inebilizumab treatment. Mean data with error bars showing 95% CI estimated by negative binomial regression. AAR = annualised attack rate, CI = confidence interval, EDSS = Expanded Disability Status Scale, MRI = magnetic resonance imaging, RCP = randomised controlled period.

We observed longitudinal decreases in NMOSD activity with long-term inebilizumab treatment. Therefore, the short- and long-term relationships between clinical and imaging metrics and subgroups with 6-month CD20+ B-cell counts >4 cells/μL or ≤4 cells/μL was explored. NMOSD activity decreased in both subgroups after the first dosing period of inebilizumab treatment compared with placebo. Subsequent doses of inebilizumab further decreased disease activity in both subgroups over time. NMOSD activity, especially new/enlarging T2 MRI lesions, decreased more rapidly among participants with CD20+ B-cell levels ≤4 cells/μL than in those with counts >4 cells/μL; however, after 2.5 years of inebilizumab exposure, all participants showed similar levels of NMOSD activity (Fig. 6).

### Assessment of participant subgroups

Three separate, but not mutually exclusive, mechanisms could affect B-cell counts at the end of the first inebilizumab dosing interval.

### Pharmacokinetic loss of inebilizumab may lead to B-cell repletion

Pharmacokinetic (PK) loss of inebilizumab after administration of the two doses on days 1 and 15 could have led to repletion of B cells in some participants by the end of the first dosing interval. PK profiles of inebilizumab were compared in the subgroups with B-cell counts >4 cells/μL and ≤4 cells/μL at the end of the first dosing interval. Participants with less B-cell depletion had lower inebilizumab concentrations than those with stable B-cell depletion: inebilizumab was undetectable at week 22 in the serum of most participants with B-cell counts >4 cells/μL (LLOQ: 100.5 ng/mL, Supplementary Figure S6). Additional analysis of baseline covariates revealed that age, sex, race, weight, and body mass index did not have a statistically significant association with higher B-cell counts (defined as >4 cells/μL at week 28; data not shown). Prior rituximab use was also not associated with B-cell counts (data not shown). Participants with B-cell counts >4 cells/μL after the first dosing period displayed statistically significant elevated CD19+ B-cell counts, plasma-cell signature, and total Ig concentrations on day 1 of the RCP (false discovery rate <0.10; *p* < 0.01 Supplementary Figure S7).

### Relationship between B-cell depletion and rs396991 polymorphism in *FCGR3A*

An exploratory analysis of the relationship between B-cell counts at the end of the first dosing interval and the rs396991 polymorphism in *FCGR3A* was performed. The *FCGR3A* genotype was profiled in a subgroup of participants who consented to genomic analysis. F/F homozygosity was noted in 100% of participants (10/10) who never achieved CD20+ B-cell counts of ≤4 cells/μL during the first dosing interval, in 42.9% of participants (12/28) whose CD20+ B cells depleted to ≤4 cells/μL but repleted to >4 cells/μL by the end of the first 6-month treatment period, and in 45.2% of participants (38/84) with sustained CD20+ B-cell depletion ≤4 cells/μL (Supplementary Figure S8).

### Relationship between anti-drug antibodies and B-cell depletion

Anti-drug antibodies (ADAs) that bind to or neutralise inebilizumab could affect changes in B-cell counts. In total, 33/225 participants (14.7%) treated with inebilizumab tested positive for ADAs intermittently on study. Of these participants, 7.2% had treatment-emergent ADAs, or ADAs that are treatment-induced or a baseline titre boosted to a fourfold higher level. The median of maximum titre was 100 (lowest titre 1:50 dilution), and these titres decreased with long-term inebilizumab treatment (Supplementary Figure S9a). In general, B-cell counts were modestly increased with ADA positivity, but no significant overlap was found between participants positive for ADAs and participants with B-cell levels >4 cells/μL before completing the first dosing interval (Supplementary Figure S9b). Median B-cell counts in samples drawn 90 days from periods of ADA positivity were negligibly higher than in samples from participants who were persistently ADA-negative (1.5 cells/μL vs 0.4 cells/μL; Supplementary Figure S9c). There was no impact on PK, pharmacodynamics, safety, or efficacy in ADA-positive participants compared with those negative for ADAs.

## Discussion

N-MOmentum demonstrated the efficacy of the anti-CD19 monoclonal antibody inebilizumab in reducing disease activity in participants with NMOSD compared with placebo.^19^ This outcome was likely related to the B-cell depletion observed in participants treated with inebilizumab; the density of sampling, especially during the RCP, helped to elucidate the pharmacodynamic effect of treatment on B-cell function in NMOSD. All participants experienced B-cell depletion in the first week after the first inebilizumab treatment and maintained B-cell counts lower than those before treatment by the end of the first dosing period. Inebilizumab-treated participants also had significant plasma-cell gene signature and Ig depletion, providing evidence that inebilizumab also depletes plasmablasts and antibody-secreting cells. The correlation between depth of B-cell depletion and reduction in NMOSD activity with long-term inebilizumab treatment underscores a central role for B cells in NMOSD pathogenesis and suggests that early monitoring of B-cell counts could be important for management of inebilizumab-treated patients with NMOSD. Furthermore, monitoring after the second full dose of inebilizumab at 6 months may not be necessary, given the consistent therapeutic response over time. The results included in this manuscript are part of an exploratory analysis: thus, the *p* values reported are for hypothesis generation only. Validation studies that confirm the correlation of duration and depth of B-cell depletion with clinical response could establish a new paradigm for monitoring and treating patients with inebilizumab. Therapeutic response biomarkers have yet to be developed for CNS inflammatory diseases, and the relative ease with which B-cell counts can be monitored makes this biomarker particularly attractive for clinical practice.

Interestingly, CD20+ B-cell counts were not found to be associated with disease activity during NMOSD relapses. It may be that total CD20+ B-cell counts are not a sensitive enough biomarker of disease activity during NMOSD attacks; indeed, monitoring CD27+ memory B cells was key to reducing relapse risk in patients with NMOSD treated intermittently with rituximab.^27^ Further work is needed to determine whether or not a defined B-cell subset (such as CD27+ memory B cells or plasmablasts) may be a more sensitive predictor of disease activity.

It is encouraging that, following initial treatment with inebilizumab, 70% of participants in N-MOmentum experienced deep B-cell depletion (≤4 cells/μL) after the first dosing period that was maintained with subsequent treatments and was associated with long-term sustained remission. Even those with less substantial B-cell depletion benefitted from treatment but remained at a somewhat higher risk of attack until approximately 2.5 years after treatment initiation, when there were no longer differences in disease activity between the groups. It was not possible to evaluate the correlation of disease activity with B-cell depletion before 6 months owing to the low number of attacks between 3 months and 6 months.

A significant correlation between depth of B-cell depletion and new/enlarging T2 MRI lesions was observed. Prior studies demonstrated that subclinical lesions in the brain are rare in NMOSD^28^, ^29^, ^30^; however, this is the first study in patients with NMOSD that performed serial imaging of the optic nerve and spinal cord. At this time, it is unclear whether or not these lesions bear any clinical significance, because we did not observe a proportional increase in NMOSD attacks or EDSS worsening. Future work will be necessary to characterise these lesions and to assess their clinical relevance.

Several factors may contribute to the variation in B-cell responses observed in different subgroups. Participants with elevated B-cell counts after the first dosing period had significantly higher CD19+ B-cell, plasma-cell, and total Ig concentrations at baseline than those showing sustained B-cell depletion. This may be attributable to a higher B-cell ‘load’ or to an active immune system when treatment was initiated. An increased B-cell load may also explain differences in inebilizumab PK between the groups, with these participants requiring a higher inebilizumab antibody titre than those with a lower B-cell load at baseline to reduce B-cell concentrations. Furthermore, patients showing elevated levels of CD19+ B cells at baseline could have an elevated rate of B-cell production, increasing the rate of inebilizumab consumption, and thus accelerating the loss of PK and subsequent repletion of B cells.

The *FCGR3A* F/F variant is known to reduce the efficacy of ADCC-mediated effects of monoclonal antibody therapies. Notably, inebilizumab was glycoengineered for enhanced binding to the low-affinity immunoglobulin gamma Fc region receptor III and, therefore, enhanced ADCC activity. Although the homozygous polymorphism was more prevalent in the subgroup of participants with elevated CD20+ B-cell counts than in those with CD20+ B-cell counts ≤4 cells/μL, the number of genotyped participants in the shallow depletion group was very small. Further studies are necessary to determine the impact of the *FCGR3A* F/F polymorphism on B-cell depletion and disease activity in patients treated with inebilizumab.

Few participants were tested positive for inebilizumab-ADAs, and both ADA incidence and titre decreased over time on treatment. Importantly, although ADAs were observed in a subset of participants, these had no impact on the efficacy of inebilizumab. Investigation of a possible relationship between B-cell counts and ADA positivity found that B-cell counts were slightly elevated within 90 days of an ADA-positive sample, but the median level was below the 4 cells/μL cut-off point associated with an increased risk of attacks. It is unclear whether this association between ADAs and increased B-cell counts was driven by ADAs or whether the slight delay in reaching deep B-cell depletion in some participants led to the development of low-level immunogenicity against inebilizumab. The general decrease in ADA titres observed over time may be attributable to the deep and consistent B-cell depletion achieved with prolonged inebilizumab treatment. The low frequency of ADAs, together with the associated limited impact on B-cell counts, supports the primacy of B-cell counts as an immediate reflection of efficacy.

It is important to note that, despite short-term differences in disease activity between the ≤4 cells/μL and >4 cells/μL subgroups, all participants treated with inebilizumab displayed immediate and significant reductions in NMOSD activity compared with those receiving placebo. Furthermore, disease activity was similar in both subgroups after 2.5 years of continued inebilizumab treatment. Retention rates in N-MOmentum were high (97% of participants completed the RCP; 81% of the 216 participants entering the OLP completed it), so this convergence was a treatment effect and not attributable to dropout among partial responders. In light of the treatment effects of inebilizumab, it would be of interest to ascertain which therapeutic benefits relate to this specific mechanism of action compared with anti-CD20-targeted antibody therapies. Although the presumption is that any additional efficacy seen with inebilizumab would be related to the wider expression pattern of CD19 in the B-cell lineage (including plasmablasts, plasma cells, and pro-/pre-B cells), there are currently no head-to-head data with which to compare therapeutic efficacy accurately. Further studies are necessary to determine which B-cell subsets are most impactful in driving NMOSD pathology.

In conclusion, this analysis suggests that inebilizumab treatment may be associated with specific, rapid, and durable depletion of CD19+ B cells, including plasmablasts, in participants with NMOSD compared with placebo. Our data suggest that deep and persistent B-cell depletion is beneficial in preventing attacks in patients with NMOSD, and that regular monitoring of B-cell counts may be valuable in informing the most effective use of inebilizumab for managing patients with NMOSD.

## Contributors

J.L.B.: Conceptualization; Methodology; Investigation; Writing – Review & Editing.

O.A.: Conceptualization; Methodology; Investigation; Writing – Review & Editing.

W.A.R.: Conceptualization; Methodology; Investigation; Formal analysis; Data Curation; Writing – Review & Editing.

M.A.S.: Conceptualization; Methodology; Investigation, Formal analysis; Data Curation; Writing – Review & Editing.

M.G.: Methodology; Investigation; Data Curation; Writing – Review & Editing.

L.Y.: Data Curation; Writing – Review & Editing.

D.S.: Conceptualization; Methodology; Investigation; Formal analysis; Data Curation; Writing – Review & Editing.

D.C.: Conceptualization; Methodology; Investigation; Writing – Review & Editing.

S.J.P.: Conceptualization; Methodology; Investigation; Writing – Review & Editing.

B.G.W.: Conceptualization; Methodology; Investigation; Writing – Review & Editing.

F.P.: Conceptualization; Methodology; Investigation; Writing – Review & Editing.

R.M.: Conceptualization; Methodology; Investigation; Writing – Review & Editing.

D.W.: Conceptualization; Methodology; Investigation; Writing – Review & Editing.

G.C.: Formal analysis; Data Curation; Writing – Review & Editing.

A.G.: Conceptualization; Methodology; Investigation; Writing – Review & Editing.

H.-P.H.: Conceptualization; Methodology; Investigation, Writing – Review & Editing.

H.J.K.: Conceptualization; Methodology; Investigation; Writing – Review & Editing.

K.F.: Conceptualization; Methodology; Investigation; Writing – Review & Editing.

M.L.: Conceptualization; Methodology; Investigation; Writing – Review & Editing.

E.K.: Conceptualization; Methodology; Investigation; Writing – Review & Editing.

B.A.C.C.: Conceptualization; Methodology; Investigation; Writing – Review & Editing.

J.L.B. and B.A.C.C. had access to data for verification; all authors read and approved the final version of this manuscript for submission.

## Data sharing statement

Access to anonymised, individual, and trial-level data (analysis data sets) may be granted upon reasonable request to qualified researchers for independent scientific research, provided the trials are not part of an ongoing or planned regulatory submission (including clinical trial data for unlicensed products and indications). Clinical trial data can be requested by submitting a research proposal and statistical analysis plan to Horizon Therapeutics. Data will be provided following review and approval of the plan and execution of a data sharing agreement. For more information, or to submit a request, please email medicalinformation@horizontherapeutics.com.

## Declaration of interests

**J.L.Bennett** reports payment for study design/consultation from MedImmune; personal fees from AbbVie, Alexion, Antigenomycs, BeiGene, Chugai, Clene Nanomedicine, Genentech, Genzyme, Reistone Bio, Roche, and TG; grants from Alexion, the National Institutes of Health, and Novartis. In addition, Dr Bennett has a patent ‘Compositions and methods for the treatment of neuromyelitis optica’.

**O.Aktas** reports grants from the German Research Foundation (DFG) and the German Ministry of Education and Research (BMBF); grants and personal fees from Bayer HealthCare, Biogen, Genzyme, Horizon Therapeutics (formerly from Viela Bio), Novartis, and Teva; and personal fees from Almirall, MedImmune, Merck Serono, and Roche.

**W.A.Rees, M.A.Smith, D.She,** and **D.** **Cimbora** are employees of Horizon Therapeutics (formerly Viela Bio) and own stock.

**L.Yan**, **M.Gunsior**, and **E.Katz** are former employees of Horizon Therapeutics.

**S.J.Pittock** reports grants, personal fees, and non-financial support from Alexion Pharmaceuticals, Inc.; grants from Autoimmune Encephalitis Alliance and Grifols; grants, personal fees, non-financial support, and other from Horizon Therapeutics (formerly from Viela Bio) and MedImmune; personal fees for consulting services from Astellas; grants, personal fees, non-financial support and other from Roche/Genentech; personal fees for consulting services from UCB; and has a patent #9,891,219 (Application#12-573942) ’Methods for Treating Neuromyelitis Optica (NMO) by Administration of Eculizumab to an individual that is Aquaporin-4 (AQP4)-IgG Autoantibody Positive’.

**B.G.Weinshenker** received payments for serving as chair of attack adjudication committees for clinical trials in NMOSD for Alexion, MedImmune, and Viela Bio/Horizon Therapeutics; has consulted with Chugai, Genentech, Horizon Therapeutics, Mitsubishi Tanabe Pharma, and Roche Pharmaceuticals; and has a patent for NMO-IgG for diagnosis of neuromyelitis optica, with royalties paid by Hospices Civils de Lyon, MVZ Labor PD Dr. Volkmann und Kollegen GbR, University of Oxford, and RSR.

**F.Paul** has received research support, speaker honoraria, and travel reimbursement from Bayer, Biogen Idec, Merck Serono, Novartis, Sanofi Genzyme, and Teva; is supported by the German Research Council (DFG Exc 257) and the German Competence Network for Multiple Sclerosis; has received travel reimbursement from the Guthy-Jackson Charitable Foundation; and serves on the steering committee of the OCTIMS study, sponsored by Novartis.

**R.Marignier** reports personal fees for consulting from Alexion, Horizon Therapeutics (formerly Viela Bio), Roche, and UCB.

**D.Wingerchuk** reports personal fees from Biogen, Celgene, Genentech, MedImmune, Mitsubishi Tanabe, Novartis, Reistone Biopharma, and TG Therapeutics; research support paid to the Mayo Clinic by Alexion and Terumo BCT; and has served on a clinical trial adjudication committee for Horizon Therapeutics (formerly Viela Bio) and MedImmune.

**G.Cutter** has received personal fees for participation on Data and Safety Monitoring Boards from AstraZeneca, Avexis Pharmaceuticals, BioLineRx, Brainstorm Cell Therapeutics, Bristol Myers Squibb/Celgene, CSL Behring, the Eunice Kennedy Shriver National Institute of Child Health and Human Development (Obesity Policy Research Unit oversight committee), Galmed Pharmaceuticals, Hisun Pharmaceutical, Horizon Pharmaceuticals (formerly Viela Bio), Mapi Pharma, Merck, Merck/Pfizer, the National Heart, Lung, and Blood Institute (Protocol Review Committee), Neurim Pharmaceuticals, Novartis, OncoImmune, OPKO Biologics, Orphazyme, Reata Pharmaceuticals, Sanofi-Aventis, Teva Pharmaceuticals, and Vivus; personal fees for consulting or advisory board participation from BioDelivery Sciences International, Biogen, Click Therapeutics, Genentech, Genzyme, GW Pharmaceuticals, Immunic, Klein Buendel, MedDay, MedImmune, NeuroGenesis, Novartis, Osmotica Pharmaceuticals, Perception Neurosciences, Recursion/Cerexis Pharmaceuticals, Roche, and TG Therapeutics; is employed by the University of Alabama at Birmingham, AL, USA; and is President of Pythagoras, Inc., a private consulting company based in Birmingham, AL, USA.

**A.Green** reports grants from the Conrad N. Hilton Foundation and the Tom Sherak MS Hope Foundation; other financial relationships (for activities as expert witness, associate editor, advisory board/steering committee participation, and endpoint adjudication) with Bionure, Inception Sciences, *JAMA Neurology*, MedImmune/Horizon Therapeutics (formerly Viela Bio), Mylan, Synthon, and Trims Pharma; and personal fees from and other financial relationships with Pipeline Therapeutics.

**H.-P.Hartung** has received fees for consulting, speaking, and serving on steering committees from Bayer HealthCare, Biogen Idec, Celgene Receptos, CSL Behring, GeNeuro, Genzyme, Horizon Therapeutics (formerly Viela Bio), MedDay, MedImmune, Merck Serono, Novartis, Roche, Sanofi, and TG Therapeutics with approval by the Rector of Heinrich Heine University Düsseldorf.

**H.J.Kim** has received a grant from the National Research Foundation of Korea; consultancy/speaker fees or research support from Alexion, AprilBio, Celltrion, Daewoong Pharmaceutical, Eisai, GC Pharma, HanAll Biopharma, Horizon Therapeutics (formerly Viela Bio), Kolon Life Science, MedImmune, Merck Serono, Mitsubishi Tanabe Pharma, Novartis, Sanofi Genzyme, Teva-Handok, and UCB; and is a co-editor for the *Multiple Sclerosis Journal* and an associate editor for the *Journal of Clinical Neurology*.

**K.Fujihara** has received fees for consulting, speaking, and serving on steering committees from AbbVie, Alexion, Asahi Kasei Kuraray Medical Co., Biogen, Chugai/Roche, Eisai, Japan Tobacco, MedImmune/Viela Bio, Merck, Merck Biopharma, Mitsubishi Tanabe Pharma, Novartis, Teijin, Takeda Pharmaceutical Company, and UCB; and a grant-in-aid for scientific research from the Ministry of Health, Labour and Welfare of Japan.

**M.Levy** currently receives research support from Acorda Therapeutics, Alexion, Alnylam Pharmaceuticals, ApoPharma, Maryland Technology Development Corporation, the National Institutes of Health, Sanofi Genzyme, and Shire/Takeda; has received personal compensation for consultation with Acorda Therapeutics, Alexion, and Genzyme; and serves on the scientific advisory boards for Acorda Therapeutics, Alexion, and Quest Diagnostics.

**B.A.C.Cree** reports personal compensation for consulting from Alexion, Atara Biotherapeutics, Autobahn Therapeutics, Avotres Inc., Biogen, Boston Pharma, EMD Serono, Gossamer Bio, Hexal/Sandoz, Horizon Therapeutics, Neuron23, Novartis, Sanofi, Siemens, TG Therapeutics, and Therini Bio; and has received research support from Genentech.

## Appendix A Supplementary data

Captions for Supplementary Material

Supplementary Material

Study Protocol
