# Supplementary material for: Association between B-cell depletion and attack risk in neuromyelitis optica spectrum disorder: An exploratory analysis from N-MOmentum, a double-blind, randomised, placebo-controlled, multicentre phase 2/3 trial
Source: eBioMedicine. 2022 Nov 10;86:104321. doi: 10.1016/j.ebiom.2022.104321 (PMC9664402; doi:10.1016/j.ebiom.2022.104321)
Supplement: Captions for Supplementary Material [file mmc1.docx]

**Association between B-cell depletion and attack risk in neuromyelitis optica spectrum disorder: An exploratory analysis from N-MOmentum, a double-blind, randomised, placebo-controlled, multicentre phase 2/3 trial**

**Captions for online supplementary information**

Supplementary Methods

Supplementary Figure 1: CD19: a differentiated target for B-cell depletion.

Supplementary Figure 2: N-MOmentum study.

Supplementary Figure 3: B-cell counts for individual participants at the time of attacks.

Supplementary Figure 4. Rate ratios of disease activity based on cut-off points of B-cell level following first dosing interval and sensitivity analyses.

Supplementary Figure 5. Disease activity outcomes over time with inebilizumab treatment.

Supplementary Figure 6. PK of inebilizumab in participants with 6-month B-cell counts >4 cells/μL or ≤4 cells/μL.

Supplementary Figure 7. Baseline B-cell count, plasma cell gene signature, and total Ig of participants with >4 cells/μL or ≤4 cells/μL after the first dosing period with inebilizumab.

Supplementary Figure 8. CD20+ B-cell counts grouped according to W28 response and corresponding proportion of rs396991 F/F homozygotes.

Supplementary Figure 9. Relationship between CD20+ B-cell counts and ADA.

Supplementary Table 1. Reagents used for flow cytometry analyses.

Supplementary Table 2. List of institutional ethics committees or institutional review boards for N-MOmentum.

Supplementary Table 3. Kinetics of B-cell and immunoglobulin depletion during the study.

Supplementary Table 4. Decreases in NMOSD progression observed with long-term inebilizumab treatment.

Supplementary Table 5. 6-month B-cell counts versus long-term disease activity in participants divided by RCP treatment group.

Supplementary References.
