# Supplementary material for: Association between B-cell depletion and attack risk in neuromyelitis optica spectrum disorder: An exploratory analysis from N-MOmentum, a double-blind, randomised, placebo-controlled, multicentre phase 2/3 trial
Source: eBioMedicine. 2022 Nov 10;86:104321. doi: 10.1016/j.ebiom.2022.104321 (PMC9664402; doi:10.1016/j.ebiom.2022.104321)
Supplement: Supplementary Material [file mmc2.pdf]

Association between B-cell depletion and attack risk in neuromyelitis optica spectrum disorder: An exploratory analysis from N-MOmentum, a double-blind, randomised, placebo-controlled, multicentre phase 2/3 trial

Online supplementary information

Authors

Jeffrey L Bennett,[a] Orhan Aktas,[b] William A Rees,[c] Michael A Smith,[c] Michele Gunsior,[c]* Li Yan,[c]* Dewei She,[c] Daniel Cimbora,[c] Sean J Pittock,[d] Brian G Weinshenker,[e] Friedemann Paul,[f] Romain Marignier,[g] Dean Wingerchuk,[h] Gary Cutter,[i] Ari Green,[j] Hans-Peter Hartung,[b,k,l,m] Ho Jin Kim,[n] Kazuo Fujihara,[o] Michael Levy,[p] Eliezer Katz,[c]* and Bruce AC Cree,[q] on behalf of the N-MOmentum study investigators

Affiliations

[a]University of Colorado School of Medicine, Anschutz Medical Campus, University of Colorado, Aurora, CO, USA; [b]Medical Faculty, Heinrich Heine University Düsseldorf, Düsseldorf, Germany; [c]Horizon Therapeutics plc, Gaithersburg, MD, USA; [d]Mayo Clinic and Center for MS and Autoimmune Neurology, Rochester, MN, USA; [e]Mayo Clinic, Rochester, MN, USA; [f]Experimental and Clinical Research Center, Max Delbrück Center for Molecular Medicine and Charité – Universitätsmedizin Berlin, Corporate Member of Freie Universität Berlin and Humboldt-Universität zu Berlin, Berlin, Germany; [g]Service de Neurologie, Sclérose en Plaques, Pathologies de la Myéline et Neuroinflammation, Hôpital Neurologique Pierre Wertheimer, Hospices Civils de Lyon, Lyon, France; [h]Department of Neurology, Mayo Clinic, Scottsdale, AZ, USA; [i]University of Alabama at Birmingham, Birmingham, AL, USA; [j]UCSF Weill Institute for Neurosciences, Department of Neurology and Department of Ophthalmology, University of California San Francisco, San Francisco, CA, USA; [k]Brain and Mind Centre, University of Sydney, Sydney, NSW, Australia; [l]Department of Neurology, Medical University Vienna, Vienna, Austria; [m]Department of Neurology, Palacky University in Olomouc, Olomouc, Czech Republic; [n]Department of Neurology, Research Institute and Hospital of National Cancer Center, Goyang, South Korea; [o]Department of Multiple Sclerosis Therapeutics, Fukushima Medical University and Multiple Sclerosis and Neuromyelitis Optica Center, Southern Tohoku Research Institute for Neuroscience, Koriyama, Japan; [p]Department of Neurology, Massachusetts General Hospital and Harvard Medical School, Boston, MA, USA; [q]UCSF Weill Institute for Neurosciences, Department of Neurology, University of California San Francisco, San Francisco, CA, USA;

*Former employee of Horizon Therapeutics

Contents

Supplementary Methods…………………………………………………………………………..3

Supplementary Figure 1: CD19: a differentiated target for B-cell depletion. 6

Supplementary Figure 2: N-MOmentum study……………………………………………...……7

Supplementary Figure 3: B-cell counts for individual participants at the time of attacks. 10

Supplementary Figure 4. Rate ratios of disease activity based on cut-off points of B-cell level following first dosing interval and sensitivity analyses.. 13

Supplementary Figure 5. Disease activity outcomes over time with inebilizumab treatment.. 15

Supplementary Figure 6. PK of inebilizumab in participants with 6-month B-cell counts >4 cells/μL or ≤4 cells/μL.. 17

Supplementary Figure 7. Baseline B-cell count, plasma cell gene signature, and total Ig of participants with >4 cells/μL or ≤4 cells/μL after the first dosing period with inebilizumab. . 18

Supplementary Figure 8. CD20+ B-cell counts grouped according to W28 response and corresponding proportion of rs396991 F/F homozygotes. 19

Supplementary Figure 9. Relationship between CD20+ B-cell counts and ADA. 20

Supplementary Table 1. Reagents used for flow cytometry analyses……………...………21

Supplementary Table 2. List of institutional ethics committees or institutional review boards for N-MOmentum………………………………………………………………………...……22

Supplementary Table 3. Kinetics of B-cell and immunoglobulin depletion during the study..35

Supplementary Table 4. Decreases in NMOSD progression observed with long-term inebilizumab treatment………………………………………………………………………..38

Supplementary Table 5. 6-month B-cell counts versus long-term disease activity in participants divided by RCP treatment group……………………………………………………42

Supplementary References ……………………………………………………………………43

SUPPLEMENTARY METHODS

Study design of N-MOmentum

N-MOmentum was a multicentre, double-blind, randomised, placebo-controlled phase 2/3 study of the safety and efficacy of inebilizumab in adult participants with neuromyelitis optica spectrum disorder (NMOSD) that ran from April 2015 to November 2020. A total of 99 outpatient specialty clinics of hospitals from 25 countries participated in N-MOmentum, which was divided into two portions: the randomised controlled period (RCP) and the open-label period (OLP). Participants finished the RCP after 197 days in the study or after a diagnosed attack; after the RCP, participants could enter the OLP.

Participants in N-MOmentum

Inclusion criteria included adult age (≥18 years), diagnosis of NMOSD, Expanded Disability Status Scale (EDSS) score of 8·0 or less, and a history of either at least one attack requiring rescue therapy during the year before screening, or two attacks in the 2 years before screening. Participants who were seropositive for immunoglobulin (Ig) G antibodies against the aquaporin-4 water channel (AQP4-IgG) or AQP4-IgG seronegative were eligible; however, patients who were seronegative needed to meet the NMOSD criteria described by Wingerchuck and colleagues.[1] Exclusion criteria included potential pregnancy, any condition that would interfere with the study results, uncontrolled hypertension, concurrent enrolment in another clinical study involving investigational treatment, or inability to undergo magnetic resonance imaging (MRI).

Treatment in N-MOmentum

Participants were randomly allocated 3:1 to receive inebilizumab or placebo during the RCP. This unequal randomisation scheme was used to minimise the total number of placebo participants needed. During the RCP, participants received intravenous inebilizumab 300 mg or placebo on days 1 and 15. Participants finished the RCP after 197 days in the study or after a diagnosed attack; after the RCP, participants could enter the OLP. Participants in the OLP received intravenous inebilizumab 300 mg on day 1 of the OLP and every 6 months afterwards for the duration of the enrolment; participants who had been randomised to placebo during the RCP received an extra dose of inebilizumab 300 mg on day 15 of the OLP. For analysis purposes, participants were divided into three groups: “RCP inebilizumab” (participants randomised to inebilizumab during the RCP), “RCP placebo” (participants randomised to placebo during the RCP), and “any inebilizumab” (participants who received

inebilizumab at any point during the study, including those from the RCP inebilizumab group and all participants in the OLP).

Key outcomes in N-MOmentum

The primary endpoint of N-MOmentum was time from day 1 of the RCP to the onset of a diagnosed NMOSD attack on or before the end of the RCP. Key secondary endpoints included worsening of EDSS score from baseline, cumulative number of new/enlarging T2 MRI lesions, and cumulative number of NMOSD-related inpatient hospitalisations.

This study had an independent data monitoring committee (IDMC) that monitored trial safety and progress and convened at 12 pre-planned review meetings and additional ad hoc meetings before data were unblinded. The IDMC planned to perform an unblinded interim futility analysis when half of the expected NMOSD attacks had occurred; this analysis was performed on 14 November 2017, and the study was found not futile because the predictive power was calculated to be over 20%. The IDMC was firewalled from the rest of the blinded sponsor study team, and the sponsor and sites remained blinded to the treatment analyses during the entire study. The protocol included a planned enrolment halt when 67 NMOSD attacks were diagnosed, when 252 participants had been randomised and dosed, or following an IDMC recommendation, whichever happened first. On 7 September 2018, the IDMC recommended stopping the RCP because of a clear demonstration of efficacy of treatment and owing to the unethical nature of continuing to expose placebo-treated participants to further risk of attacks. This demonstration of efficacy was unlikely to be altered by continuing enrolment or the study. Following this recommendation, recruitment was stopped; the number of participants was 22 below the target and the number of diagnosed NMOSD attacks was 24 below the target. The final proportion of randomised participants to each treatment maintained the planned 3:1 ratio of inebilizumab to placebo treatment allocation.

Statistics in N-MOmentum

The study was designed to detect a relative reduction related to inebilizumab of 60% in time to NMOSD attack during the RCP with at least 90% power and a two-sided significance level of 5%, assuming an unequal randomisation ratio of 3:1 (inebilizumab : placebo) and that placebo participants would only receive placebo for 197 days at the most. The original sample size calculation concluded that 212 participants would need to be recruited to reach 67 attacks during the RCP. This target was calculated by assuming hazard rates of 1·5 per year and 1·0 per year for an attack in the placebo arm for AQP4-IgG seropositive and

seronegative participants, respectively. These hazard rates were based on observed attack rates in published NMOSD open-label cohort studies.[2-6]

An interim sample size re-assessment was initially planned to be executed before a planned futility analysis to protect against the loss of statistical power in the seropositive cohort, as well as in the overall population owing to the required number of events not being achieved. However, the protocol was amended on 15 December 2016 in collaboration with the US Food and Drug Administration and the pre-planned sample size re-assessment was removed. The enrolment target was revised to 252, which was calculated to provide a 90% probability of reaching 67 attacks based on the attack rate observed for the first 78 participants enrolled.

Models used for B-cell dynamics analyses

Relationships between depletion groups and treatment outcomes for summarisation were estimated by negative binomial regression using the following models.

- Figure 2: the glm.nb function from MASS: glm.nb(Count ~ time + offset(log(timeElapsed))), where each time point 'time' was coded as a factor. The predict and confint functions were used to generate point estimates for each time interval along with 95% confidence intervals (CIs).
- Figure 5: we used a similar model to the one described above: glm.nb(value ~ group:time+time+group+offset(log(timeElapsed))).
- Supplementary Figure 4: the same model was used as in Figure 5 but with AQP4-IgG seropositive participants instead of the intention-to-treat population.
- Table 1: glm.nb(value ~ log10(B cell counts) + offset(log(timeElapsed))).
- Table 2: the analysis substituted in the rates estimated from the placebo group in the RCP in Figure 5 for the first column, then we used the model:glm.nb(value ~ group+offset(log(timeElapsed))) and predict and confint functions to generate point estimates and 95% CIs to estimate post-6-month disease activity as a function of B-cell counts >4 cells/μL or ≤4 cells/μL at 6 months.

SUPPLEMENTARY FIGURES

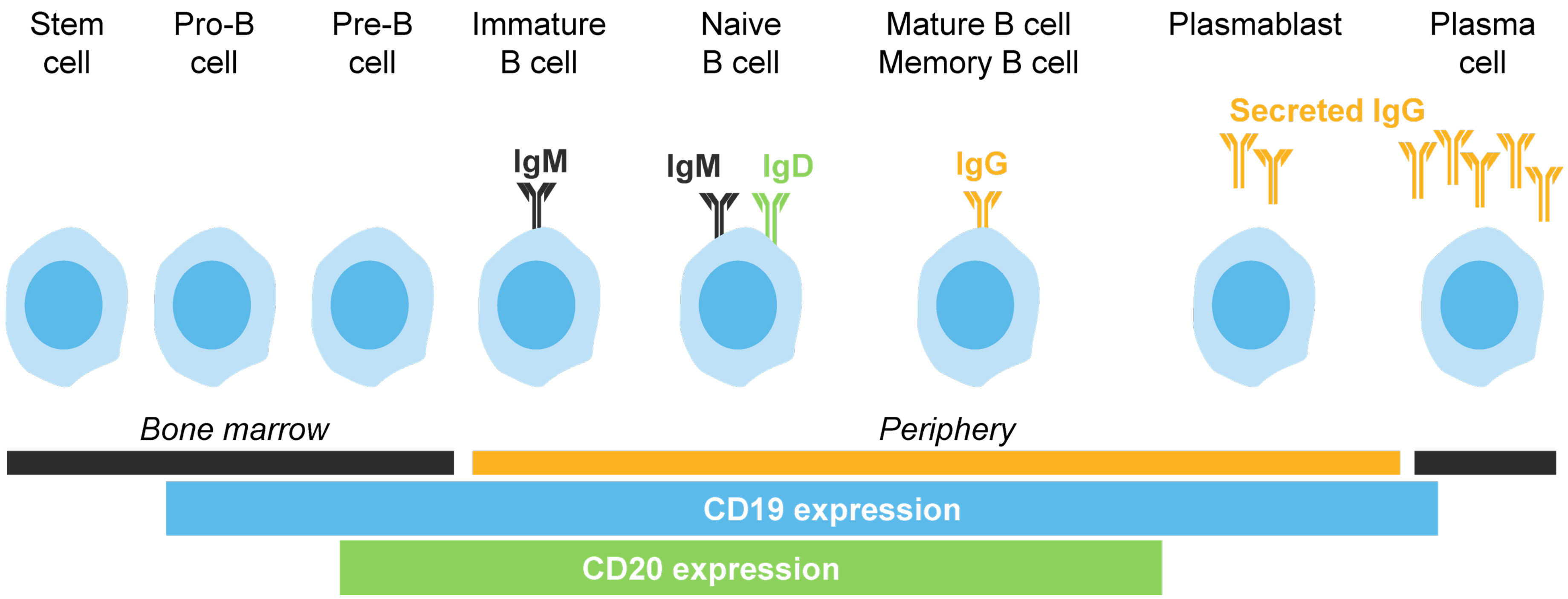

***Supplementary Figure S1*. CD19: a differentiated target for B-cell depletion.**
Ig=Immunoglobulin.

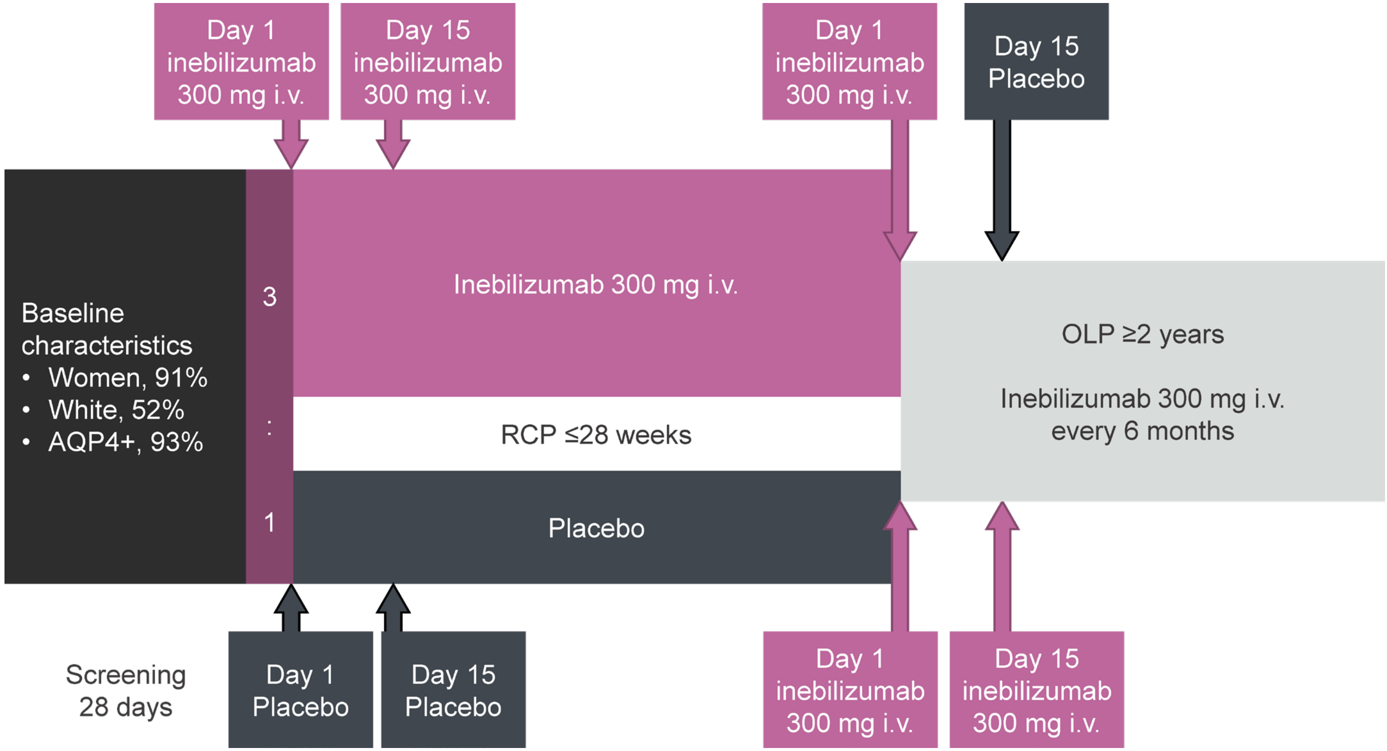

***Supplementary Figure S2.* N-MOmentum study design.** N-MOmentum was a multicentre, double-blind, randomised, placebo-controlled phase 2/3 study of the safety and efficacy of inebilizumab in adult participants with NMOSD that ran from April 2015 to November 2020. A total of 99 outpatient specialty clinics of hospitals from 25 countries participated in N-MOmentum, which was divided into two portions: RCP and OLP. During the RCP, participants received inebilizumab 300 mg i.v. or placebo on days 1 and 15. Participants finished the RCP after 197 days in the study or after a diagnosed attack; after the RCP, participants could enter the OLP. Participants in the OLP received inebilizumab 300 mg i.v. on day 1 of the OLP and every 6 months afterwards for the duration of the enrolment; participants who had been randomised to placebo during the RCP received an extra dose of inebilizumab 300 mg on day 15 of the OLP. This study had an independent data monitoring committee (IDMC) that monitored trial safety and progress and convened at 12 pre-planned review meetings and additional ad hoc meetings before data were unblinded. The IDMC planned to perform an unblinded interim futility analysis when half of the expected NMOSD attacks had occurred; this analysis was performed on 14 November 2017, and the study was found not futile because the predictive power was calculated to be over 20%. The IDMC was firewalled from the rest of the blinded sponsor study team, and the sponsor and sites remained blinded to the treatment analyses during the entire study. The protocol included a planned enrolment halt when 67 NMOSD attacks were diagnosed, when

252 participants had been randomised and dosed, or following an IDMC recommendation, whichever happened first. On 7 September 2018, the IDMC recommended stopping the RCP because of a clear demonstration of efficacy of treatment and owing to the unethical nature of continuing to expose placebo-treated participants to further risk of attacks. Following this recommendation, recruitment was stopped; the number of participants was 22 below the target and the number of diagnosed NMOSD attacks was 24 below the target.

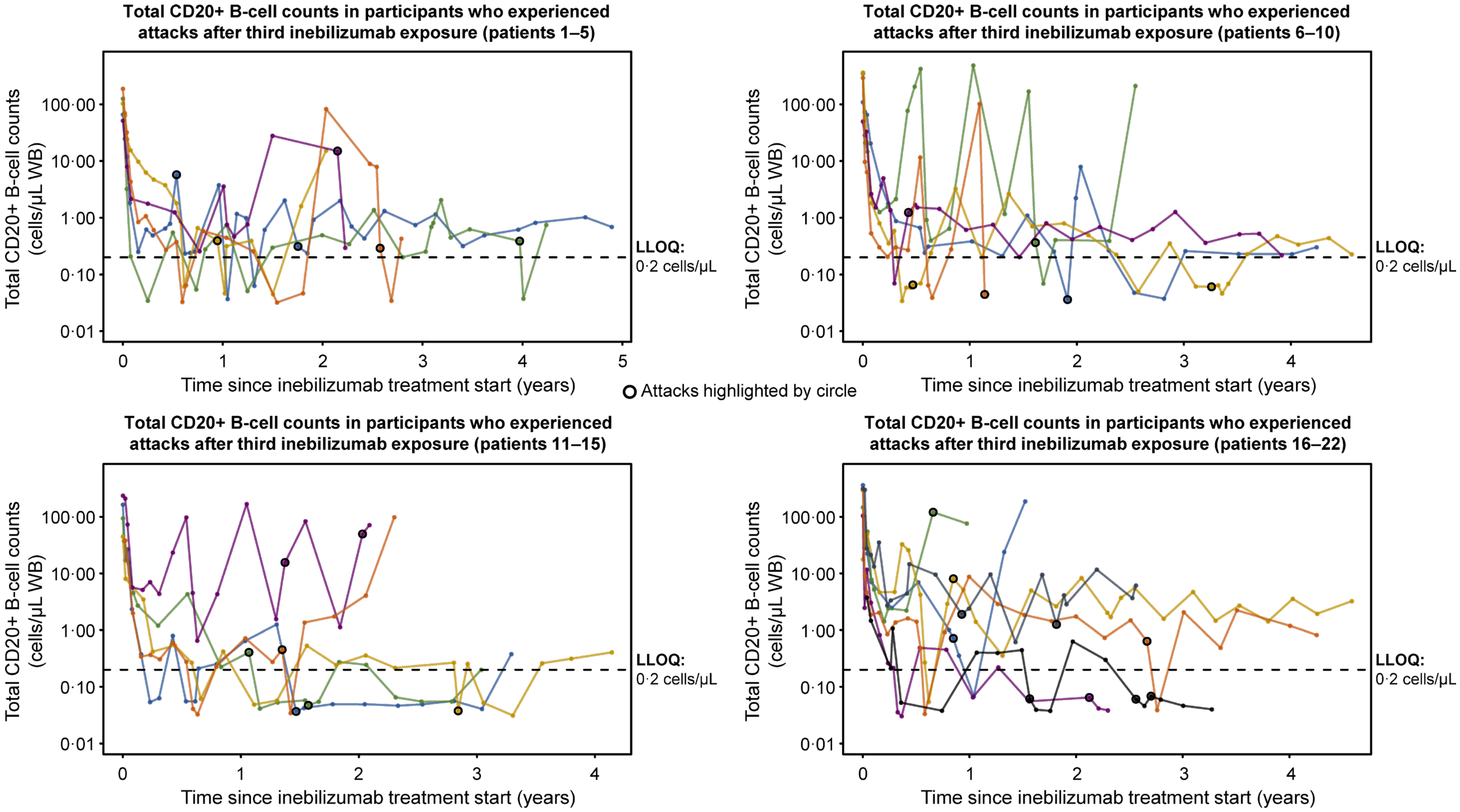
Total CD20+ B-cell counts in participants who experienced attacks after third inebilizumab exposure (patients 1–5)
Total CD20+ B-cell counts in participants who experienced attacks after third inebilizumab exposure (patients 6–10)
Total CD20+ B-cell counts in participants who experienced attacks after third inebilizumab exposure (patients 11–15)
Total CD20+ B-cell counts in participants who experienced attacks after third inebilizumab exposure (patients 16–22)
Total CD20+ B-cell counts (cells/μL WB)
100·00
10·00
1·00
0·10
0·01
LLOQ:
0·2 cells/μL
Time since inebilizumab treatment start (years)
0
1
2
3
4
5
Attacks highlighted by circle

b

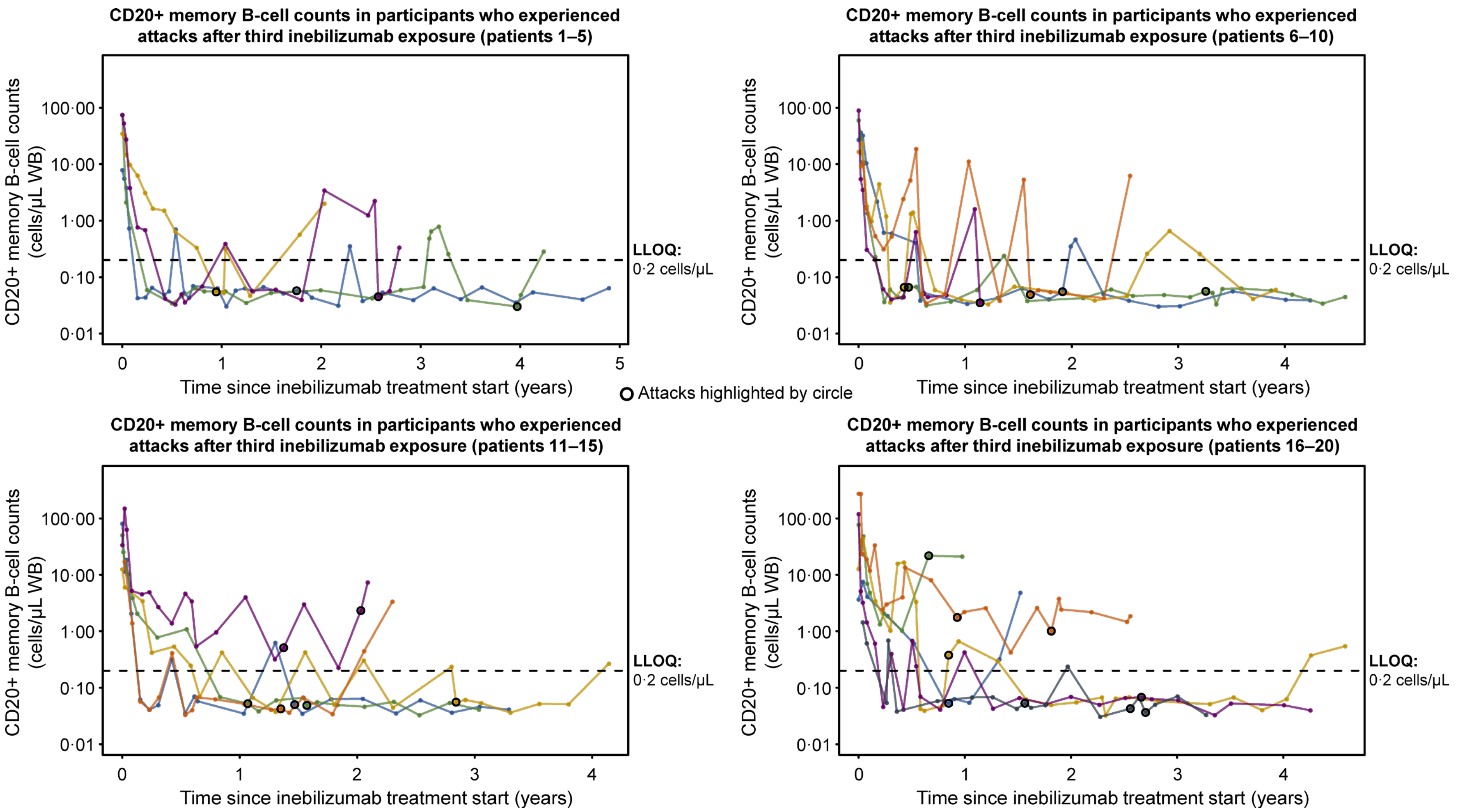
CD20+ memory B-cell counts in participants who experienced attacks after third inebilizumab exposure (patients 1–5)
CD20+ memory B-cell counts in participants who experienced attacks after third inebilizumab exposure (patients 6–10)
CD20+ memory B-cell counts in participants who experienced attacks after third inebilizumab exposure (patients 11–15)
CD20+ memory B-cell counts in participants who experienced attacks after third inebilizumab exposure (patients 16–20)
CD20+ memory B-cell counts (cells/µL WB)
100·00
10·00
1·00
0·10
0·01
Time since inebilizumab treatment start (years)
0
1
2
3
4
5
LLOQ: 0·2 cells/µL
Attacks highlighted by circle

Supplementary Figure S3. **B-cell counts for individual participants at the time of attacks. (a)** Total CD20+ and **(b)** CD20+ memory B-cell counts over time of participants with more than two inebilizumab doses who experienced attacks. Each sub-panel contains data from five participants; each participant is represented by a coloured line. LLOQ=Lower Limit Of Quantification, WB=Whole Blood.

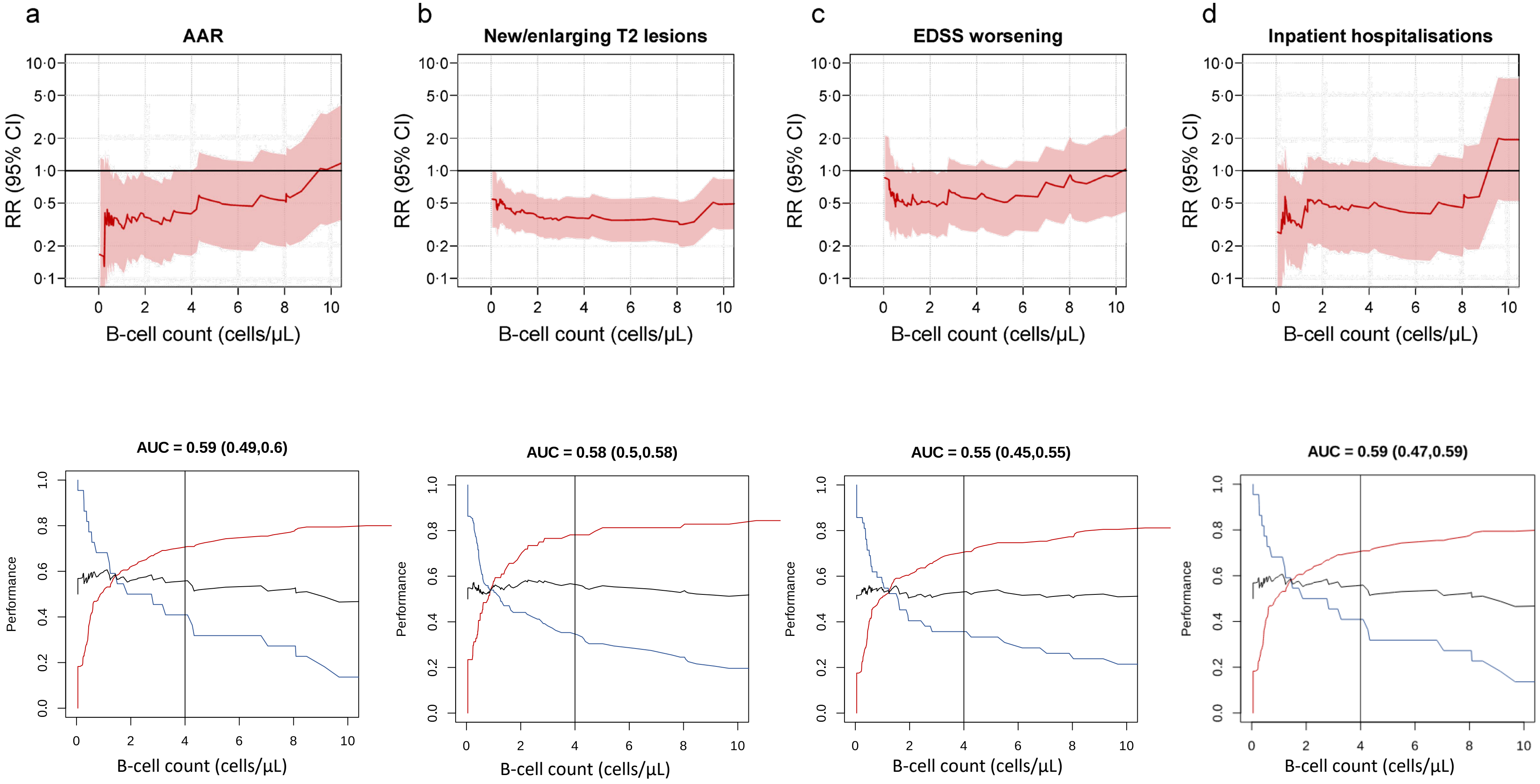

***Supplementary Figure S4*. Rate ratios of disease activity based on cut-off points of B-cell level following first dosing interval and sensitivity analyses.** Rate ratio (±95% CI shaded region) versus W26 B-cell cut-off point and sensitivity analyses of **(a)** attacks, **(b)**

new/enlarging T2 lesions, **(c)** EDSS worsening (participants/year), and **(d)** inpatient hospitalisations that occurred after the first dosing period with inebilizumab. Rate ratios were calculated by comparing the rate at which each endpoint occurred in participants below versus above each B-cell cut-off point. Sensitivity analyses for at least one event (blue), specificity for at least one event (red), and mean sensitivity and specificity (grey) as a function of the cut-off; ±95% CI, calculated through 500 bootstrap replicates using pROC package in R. AAR=Annualised Attack Rate, AUC=Area Under the Curve, CI=Confidence Interval, EDSS=Expanded Disability Status Scale, RR=Rate Ratio, W26=Week 26 After Treatment Initiation.

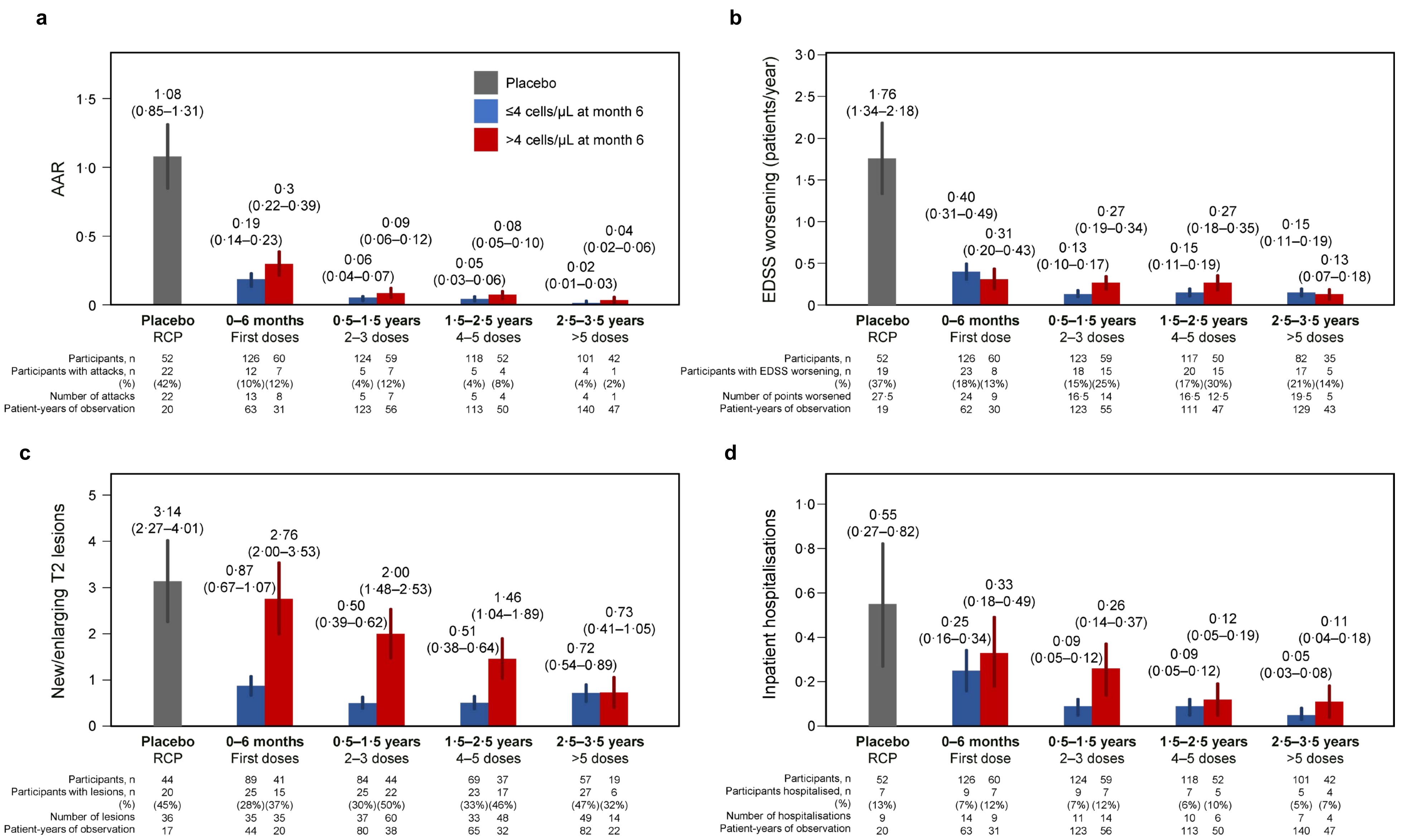

a

	Placebo RCP	0–6 months First doses		0·5–1·5 years 2–3 doses		1·5–2·5 years 4–5 doses		2·5–3·5 years >5 doses	
Participants, n	52	126	60	124	59	118	52	101	42
Participants with attacks, n	22	12	7	5	7	5	4	4	1
(%)	(42%)	(10%)	(12%)	(4%)	(12%)	(4%)	(8%)	(4%)	(2%)
Number of attacks	22	13	8	5	7	5	4	4	1
Patient-years of observation	20	63	31	123	56	113	50	140	47

b

	Placebo RCP	0–6 months First dose		0·5–1·5 years 2–3 doses		1·5–2·5 years 4–5 doses		2·5–3·5 years >5 doses	
Participants, n	52	126	60	123	59	117	50	82	35
Participants with EDSS worsening, n	19	23	8	18	15	20	15	17	5
(%)	(37%)	(18%)	(13%)	(15%)	(25%)	(17%)	(30%)	(21%)	(14%)
Number of points worsened	27·5	24	9	16·5	14	16·5	12·5	19·5	5
Patient-years of observation	19	62	30	123	55	111	47	129	43

c

	Placebo RCP	0–6 months First doses		0·5–1·5 years 2–3 doses		1·5–2·5 years 4–5 doses		2·5–3·5 years >5 doses	
Participants, n	44	89	41	84	44	69	37	57	19
Participants with lesions, n	20	25	15	25	22	23	17	27	6
(%)	(45%)	(28%)	(37%)	(30%)	(50%)	(33%)	(46%)	(47%)	(32%)
Number of lesions	36	35	35	37	60	33	48	49	14
Patient-years of observation	17	44	20	80	38	65	32	82	22

d

	Placebo RCP	0–6 months First doses		0·5–1·5 years 2–3 doses		1·5–2·5 years 4–5 doses		2·5–3·5 years >5 doses	
Participants, n	52	126	60	124	59	118	52	101	42
Participants hospitalised, n	7	9	7	9	7	7	5	5	4
(%)	(13%)	(7%)	(12%)	(7%)	(12%)	(6%)	(10%)	(5%)	(7%)
Number of hospitalisations	9	14	9	11	14	10	6	7	4
Patient-years of observation	20	63	31	123	56	113	50	140	47

Supplementary Figure S5. **Disease activity outcomes over time with inebilizumab treatment. (a)** AAR. **(b)** Annualised rate of EDSS worsening. **(c)** Annualised rate of new/enlarging T2 MRI lesions (only participants randomised to inebilizumab are included). **(d)** Annualised rate of inpatient hospitalisations**.** Data split by patients with B-cell counts >4 cells/μL or ≤4 cells/μL after the first inebilizumab dosing interval (6 months post-treatment) in AQP4-IgG seropositive participants.

Plots show rates in placebo-treated participants during the RCP (in grey), then in both participant groups treated with inebilizumab during the first dosing period (first dosing interval), and yearly afterwards (in blue and red). The final bar in each plot displays the combined rate of each endpoint after ≥2·5 years of continued inebilizumab treatment. Error bars show 95% CI estimated by negative binomial regression.

AQP4=Aquaporin-4 Water Channel, CI=Confidence Interval, EDSS=Expanded Disability Status Scale, IgG=Immunoglobulin G, RCP=Randomised Controlled Period.

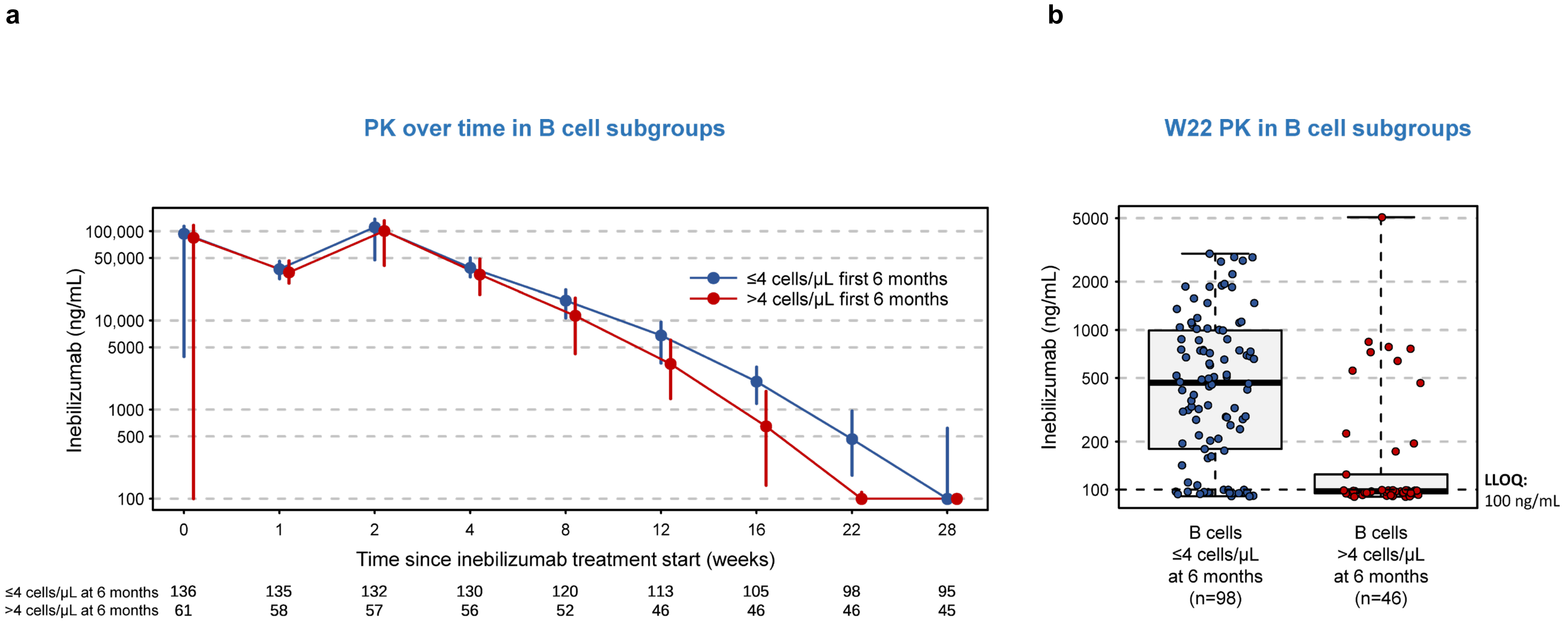

Supplementary Figure S6. **PK of inebilizumab in participants with 6-month B-cell counts >4 cells/μL or ≤4 cells/μL. (a)** Median (IQR) serum inebilizumab concentration in participants with B cells ≤4 cells/μL (blue) or >4 cells/μL (red) during the first dosing interval with inebilizumab. **(b)** Boxplot of serum inebilizumab concentration at week 22 of the RCP. IQR=Interquartile Range, LLOQ=Lower Limit Of Quantification, PK=Pharmacokinetics, RCP=Randomised Controlled Period.

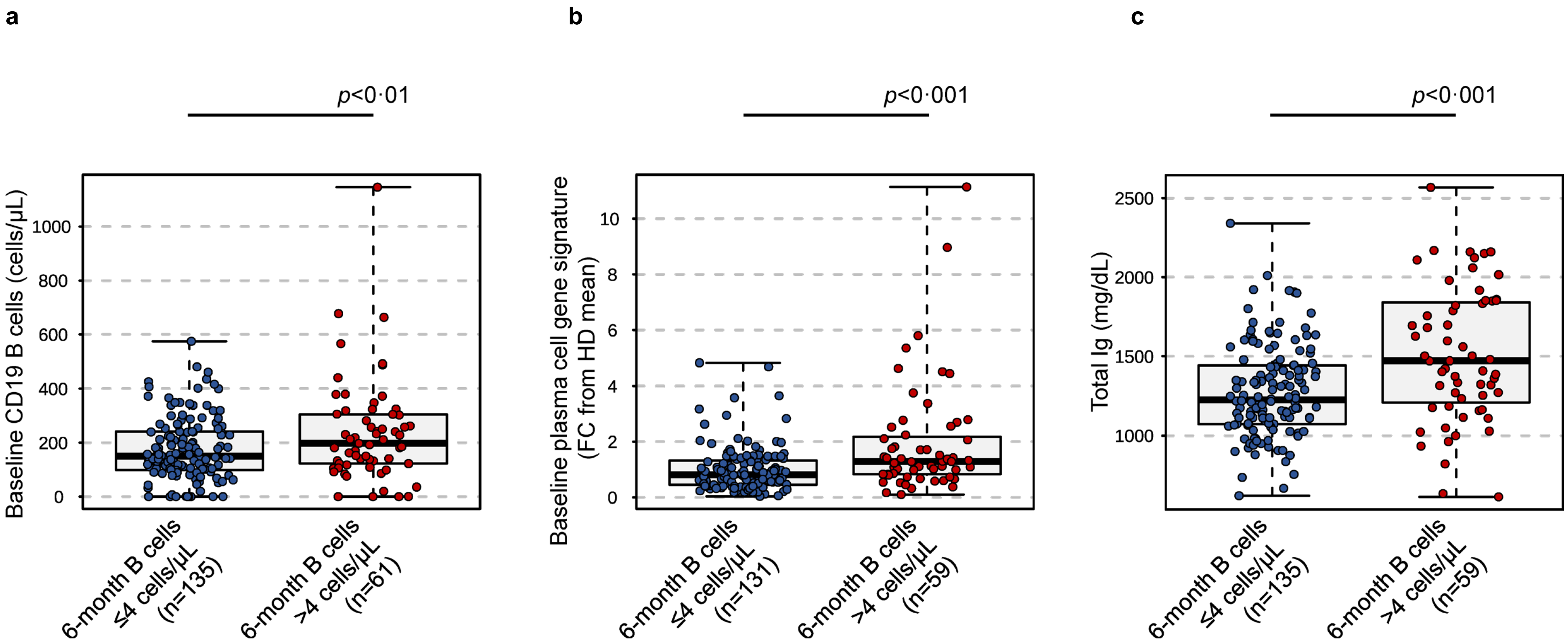

***Supplementary Figure S7*. Baseline B-cell count, plasma cell gene signature, and total Ig of participants with >4 cells/μL or ≤4 cells/μL after the first dosing period with inebilizumab. (a)** Baseline blood CD19+ B-cell counts, **(b)** plasma cell gene signature, and **(c)** total immunoglobulin across the two B-cell depletion subgroups after first dosing period with inebilizumab. Mann–Whitney U test. FC=Fold Change, HD=Healthy Donor, Ig=Immunoglobulin.

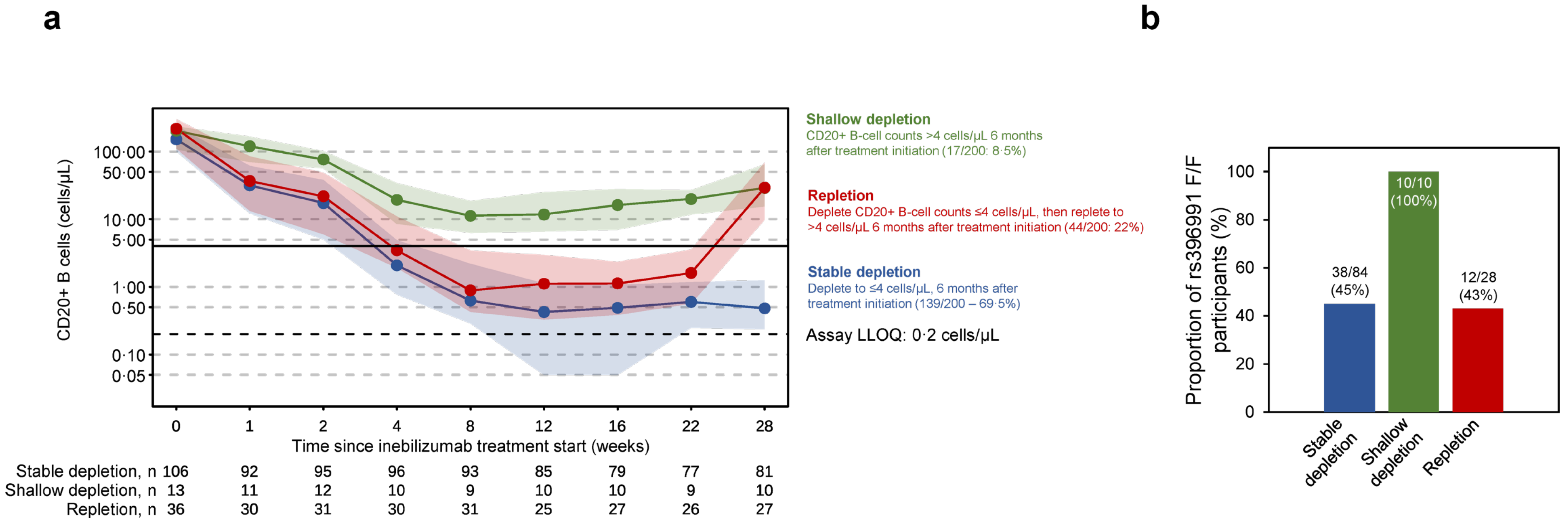

Supplementary Figure S8. **CD20+ B-cell counts grouped according to W28 response and corresponding proportion of rs396991 F/F homozygotes. (a)** Median B-cell counts from the RCP in participants randomised to inebilizumab with B-cell counts ≤4 cells/μL after the first dosing period of inebilizumab treatment (blue), and in those with B-cell counts >4 cells/μL at the end of the first dosing period further subdivided by whether participants displayed depletion ≤4 cells/μL at any point during this interval (red and blue) or whether their B-cell levels stayed >4 cells/μL (green). **(b)** Bar plot displaying proportion of participants in each subgroup who were rs396991 F/F homozygotes. LLOQ=Lower Limit of Quantification, RCP=Randomised Controlled Period, W28=Week 28 After Treatment Initiation.

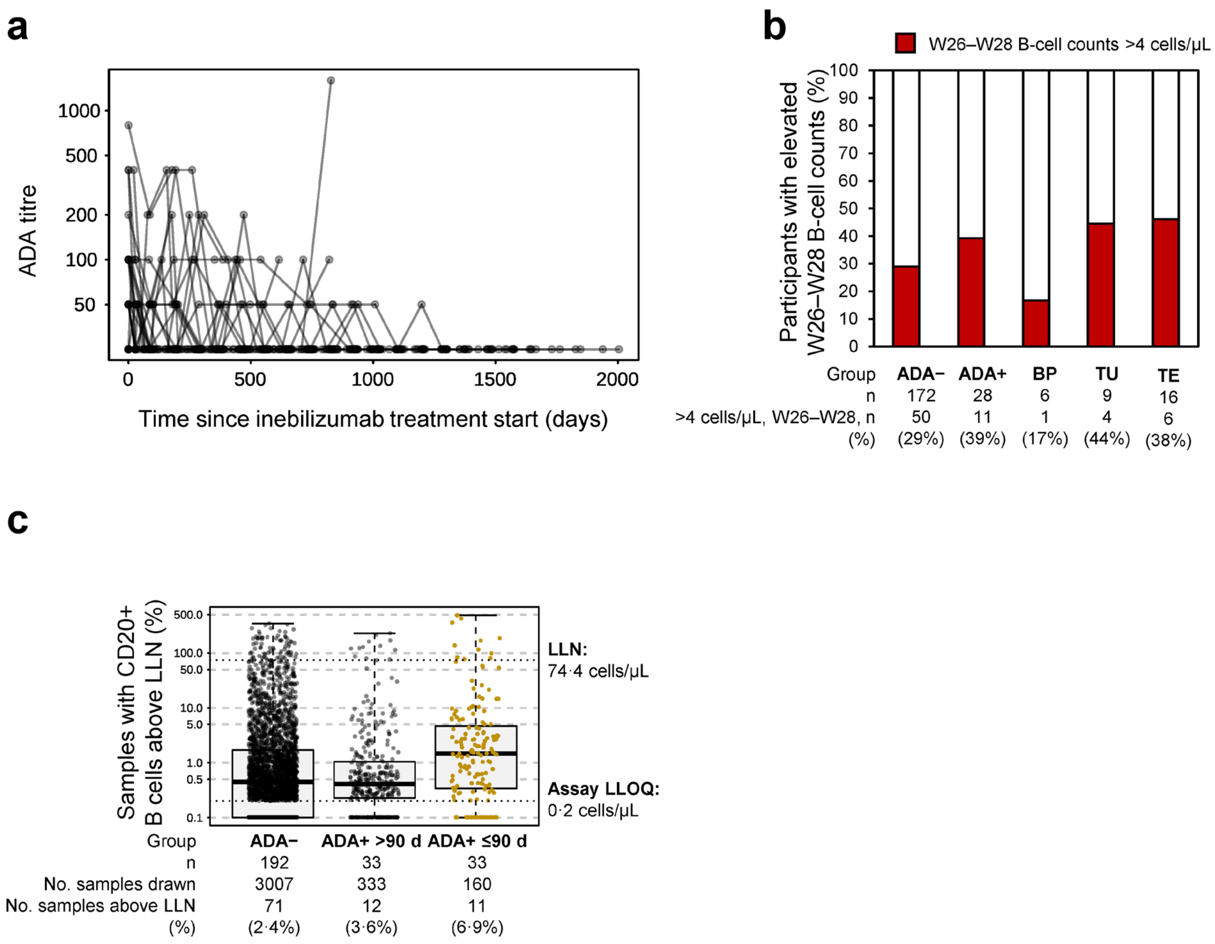

Supplementary Figure S9. **Relationship between CD20+ B-cell counts and ADA.**
(a) Profile plot of ADA titres as a function of time since start of inebilizumab treatment. **(b)** Chart displaying percentage of participants from different ADA subgroups with B-cell counts >4 cells/μL at W26–W28. **(c)** Boxplot displaying median B-cell counts in participants who were persistently ADA negative, at intervals >90 days from a positive ADA result in those who intermittently displayed ADA positivity, and at intervals within 90 days of a positive ADA result from these 'intermittent ADA' participants.
ADA=Anti-Drug Antibody, BP=Baseline Positive, LLN=Lower Limit Of Normal, LLOQ=Lower Limit Of Quantification, TU=Treatment Unaffected, TE=Treatment Emergent, W26=Week 26 After Treatment Initiation, W28=Week 28 After Treatment Initiation.

SUPPLEMENTARY TABLES

Supplementary Table S1: **Reagents used for flow cytometry analyses.**

Reagent	Assay	Fluor	Clone	RRID
CD45	CD20+ B cells; PC/PB	APC-h7	2D1	AB_1645479
CD3	CD20+ B cells	FITC	UCHT1	AB_314059
CD56	CD20+ B cells	PerCP-Cy5.5	HCD56	AB_893391
CD14	CD20+ B cells	FITC	HCD14	AB_830676
CD33	CD20+ B cells	PerCP-Cy5.5	WM53	AB_2074242
CD20	CD20+ B cells	APC	2H7	AB_314257
CD3	PC/PB	BV510	UCHT1	AB_2563467
CD14	PC/PB	BV510	M5E2	AB_2561379
CD56	PC/PB	BV510	HCD56	AB_2561385
CD27	PC/PB	PE	O323	AB_314299
CD38	PC/PB	Pe-Cy7	HB-7	AB_2562576
HLA-DR	PC/PB	APC	L243	AB_314687

***Supplementary Table S2:* List of institutional ethics committees or institutional review boards for N-MOmentum.**

**Region**	**Country**	**Site number**	**Agency type**	**Agency**
Central institutional ethics committees or institutional review boards				
Asia Pacific	New Zealand	–	Ethics - Central	Southern Health and Disability Ethics Committee
EMEA	Bulgaria	–	Ethics - Central	Ethics Committee for Clinical Trials
EMEA	Czech Republic	–	Ethics - Central/Local	Eticka komise Vseobecne fakultni nemocnice v Praze
EMEA	Estonia	–	Ethics - Central	Research Ethics Committee of the University of Tartu
EMEA	Germany	–	Ethics - Central/Local	Ethikkommission an der medizinischen Fakultät der Heinrich-Heine-Universität
EMEA	Greece	–	Ethics - Central	National Ethics Committee
EMEA	Hungary	–	Ethics - Central	Egeszsegugyi Tudomanyos Tanacs Klinikai Farmakologiai Etikai Bizottsaga
EMEA	Moldova, Republic of	–	Ethics - Central	The National Committee for Ethical Review of Clinical Trials
EMEA	Netherlands	–	Ethics - Central	METC Erasmus MC
EMEA	Poland	–	Ethics - Central/Local	Komisja Bioetyczna przy Uniwersytecie Medycznym w Lodzi
EMEA	Portugal	–	Ethics - Central	Comissão de Ética para a Investigação Clínica - CEIC
EMEA	Russian Federation	–	Ethics - Central	The RF MoH, Department of State Regulation of Circulation of Medicines, Ethics Council
EMEA	Spain	–	Ethics - Central/Local	CEIC Hospital Clinico San Carlos
EMEA	Turkey	–	Ethics - Central	Istanbul Universitesi Cerrahpasa Tip Fakultesi Klinik Arastirmalar Etik Kurulu
Latin America	Brazil	–	Ethics - Central	Comissao Nacional de Etica em Pesquisa - CONEP
North America	Canada	–	Ethics - Central/Local	Quorum Review IRB

Region	Country	Site number	Agency type	Agency
North America	USA	–	Ethics - Central/Local	Quorum Review IRB
Local institutional ethics committees or institutional review boards				
Asia Pacific	Hong Kong	2000594	Ethics - Local	Joint Chinese University of Hong Kong - New Territories East Cluster Clinical Research Ethics Commit
Asia Pacific	Hong Kong	2000595	Ethics - Local	The Unviersity of Hong Kong/ Hospital Authority Hong Kong West Cluster Institutional Review Board
Asia Pacific	Hong Kong	2000597	Ethics - Local	Kowloon Central Cluster REC / Kowloon East Cluster REC
Asia Pacific	China	2003693	Ethics - Local	The Third Affiliated Hospital, Sun Yat-Sen University - EC
Asia Pacific	China	2003732	Ethics - Local	Tianjin Medical University-General Hospital EC office
Asia Pacific	China	2003755	Ethics - Local	Xuanwu Hospital Capital Medical University EC
Asia Pacific	India	2003588	Ethics - Local	Ethics Committee, Sir Ganga Ram Hospital
Asia Pacific	India	2003599	Ethics - Local	Central Ethics Committee, Nitte University, University Enclave
Asia Pacific	India	2003609	Ethics - Local	Institutional Ethics Committee, Amrita Institute of Medical Sciences and Research Centre
Asia Pacific	India	2003731	Ethics - Local	Institutional Ethics Committee I, Seth GS Medical College and KEM Hospital
Asia Pacific	India	2003737	Ethics - Local	Institutional Ethics Committee Sree Chitra Tirunal Institute
Asia Pacific	India	2003744	Ethics - Local	NIMS Institutional Ethics Committee
Asia Pacific	Japan	2000669	Ethics - Local	Kyoto Miniren Hospital Institutional Review Board
Asia Pacific	Japan	2000669	Ethics - Local	Kyoto University Hospital Institutional Review Board
Asia Pacific	Japan	2000711	Ethics - Local	Tokyo Women's Medical University Hospital Institutional Review Board

Region	Country	Site number	Agency type	Agency
Asia Pacific	Japan	2000846	Ethics - Local	Yamaguchi University Hospital IRB
Asia Pacific	Japan	2001081	Ethics - Local	National University Corporation Tohoku University Tohoku University Hospital IRB
Asia Pacific	Japan	2001128	Ethics - Local	Aomori Prefectural Central Hospital Institutional Review Board
Asia Pacific	Japan	2001230	Ethics - Local	University of Tsukuba Hospital Institutional Review Board
Asia Pacific	Japan	2002885	Ethics - Local	Ebara Hospital Institutional Review Board
Asia Pacific	Japan	2003213	Ethics - Local	Juntendo University Hospital IRB
Asia Pacific	Japan	2003552	Ethics - Local	National Hospital Organization Hokkaido Medical Center Institutional Review Board
Asia Pacific	Japan	2003605	Ethics - Local	Southern TOHOKU Research Institute for Neuroscience Southern TOHOKU General Hospital IRB
Asia Pacific	Korea, Republic of	2000601	Ethics - Local	National Cancer Center IRB
Asia Pacific	Korea, Republic of	2000602	Ethics - Local	Samsung Medical Center Institutional Review Board
Asia Pacific	Korea, Republic of	2000603	Ethics - Local	Seoul National University Hospital IRB
Asia Pacific	Korea, Republic of	2000604	Ethics - Local	Keimyung University Dongsan Hospital IRB
Asia Pacific	Korea, Republic of	2000605	Ethics - Local	Konkuk University Medical Center Institutional Review Board
Asia Pacific	Taiwan, Province of China	2000643	Ethics - Local	Institutional Review Board of National Cheng Kung University Hospital
Asia Pacific	Taiwan, Province of China	2000644	Ethics - Local	Cheng-Hsin General Hospital Institutional Review Board
Asia Pacific	Taiwan, Province of China	2000645	Ethics - Local	Institutional Review Board, Changhua Christian Hospital

Region	Country	Site number	Agency type	Agency
Asia Pacific	Taiwan, Province of China	2000646	Ethics - Local	Tzu Chi General Hospital Research Ethics Committee
Asia Pacific	Thailand	2000647	Ethics - Local	Office of The Khon Kaen University Ethics Committee in Human Research
Asia Pacific	Thailand	2000648	Ethics - Local	Research Ethics Committee, Faculty of Medicine, Chiang Mai University
Asia Pacific	Thailand	2000650	Ethics - Local	The Institutional Review Board of the Faculty of Medicine, Chulalongkorn University
Asia Pacific	Thailand	2000651	Ethics - Local	Siriraj Institutional Review Board
EMEA	Czech Republic	2000585	Ethics - Local	Eticka komise Krajska Zdravotni As Nemocnice Teplice Oz
EMEA	Israel	2000598	Ethics - Local	The Chaim Sheba Medical Center EC
EMEA	Israel	2000600	Ethics - Local	Barzilai Medical Center Local EC
EMEA	Israel	2002785	Ethics - Local	Tel Aviv Sourasky EC
EMEA	Israel	2002786	Ethics - Local	Hadassah University Hospital Local EC
EMEA	Russian Federation	2000632	Ethics - Local	Ethics Committee at City Clinical Hospital #3
EMEA	Russian Federation	2000633	Ethics - Local	Ethics Committee at Siberian Regional Medical Centre
EMEA	Russian Federation	2000634	Ethics - Local	Ethics Committee at Republic Clinical Hospital for Rehabilitation Treatment
EMEA	Russian Federation	2000635	Ethics - Local	Ethics Committee at Bashkiria State Medical University
EMEA	Russian Federation	2000636	Ethics - Local	Ethics Committee at City Clinical Hospital #31
EMEA	Russian Federation	2000639	Ethics - Local	Ethics Committee at City Clinical Hospital #11
EMEA	Russian Federation	2000640	Ethics - Local	Ethics Committee at Omsk State Medical University
EMEA	Russian Federation	2003146	Ethics - Local	Ethics Committee at Siberian Regional Medical Centre
EMEA	Russian Federation	2003147	Ethics - Local	Ethics Committee at Research Center of Neurology of RAMS

Region	Country	Site number	Agency type	Agency
EMEA	Russian Federation	2003745	Ethics - Local	Ethics Committee at Kirov City Hospital # 4
EMEA	Russian Federation	2003746	Ethics - Local	Ethics Committee at City Clinical Hospital a.n. Buyanov V. M.
EMEA	Serbia	2003142	Ethics - Local	LEC Clinical Centre Serbia
EMEA	Serbia	2003144	Ethics - Local	LEC Clinical Centre Nis
EMEA	Serbia	2003145	Ethics - Local	LEC General Hospital Uzice
EMEA	South Africa	2000641	Ethics - Local	University of Cape Town, Faculty of Health Sciences Human Research Ethics Committee
EMEA	South Africa	2000642	Ethics - Local	University of Stellenbosch, Health Research Ethics Committee
EMEA	Ukraine	2003736	Ethics - Local	CEQ of Branch Medical Center Cyber Clinic Spizhenko
EMEA	Ukraine	1-3PJSOU	Ethics - Local	Comission on Ethics Questions of Kyiv City Clinical Hospital #4
EMEA	Ukraine	1-GB3UGZ	Ethics - Local	Commission on Ethics Questions of Municipal Institution Odesa Regional Clinical Hospital
Latin America	Argentina	2000519	Ethics - Local	Comité Independiente de Etica en investigación clínica "Dr. Carlos A. Barclay
Latin America	Argentina	2000520	Ethics - Local	Comisión Conjunta de Investigación en Salud (CCIS)
Latin America	Argentina	2000520	Ethics - Local	Comité Institucional de Evaluación de la Facultad de Ciencias Biomédicas de la Universidad Austral
Latin America	Brazil	2000523	Ethics - Local	Comitê de Ética em Pesquisa em Seres Humanos da Universidade de Passo Fundo
Latin America	Brazil	2000524	Ethics - Local	Comite de Etica em Pesquisa em do Hospital Moinhos de Vento/RS
Latin America	Brazil	2000525	Ethics - Local	Comitê de Ética em Pesquisa em Seres Humanos da Universidade de Passo Fundo
Latin America	Brazil	2000745	Ethics - Local	Comitê de Ética em Pesquisa em Seres Humanos da Universidade Federal de Minas Gerais

Region	Country	Site number	Agency type	Agency
Latin America	Colombia	2000533	Ethics - Local	Comite de Etica en Investigacion Colegio Mayor de Nuestra Senora del Rosario
Latin America	Colombia	2000537	Ethics - Local	Comite de Etica e Investigacion Clinica de la Fundacion Cardioinfantil
Latin America	Mexico	2000606	Ethics - Local	Comité de Etica en Investigacion de Medica Sur Sociedad Anónima Bursátil de Capital Variable
Latin America	Mexico	2000607	Ethics - Local	Comite de Etica en Investigacion del Grupo Medico Camino SC
Latin America	Mexico	2000608	Ethics - Local	Comité de Ética en Investigación de Christus Muguerza del Parque S.A. de C.V.
Latin America	Mexico	2000609	Ethics - Local	Comite Institucional de Etica CRI y Comite Institucional de Investigacion CRI
Latin America	Mexico	2000610	Ethics - Local	Comite de ética del Antiguo Hospital Civil de Guadalajara Fray Antonio Alcalde
Latin America	Mexico	2000611	Ethics - Local	Comite de Etica en Investigacion del Hospital Central Doctor Ignacio Morones Prieto
Latin America	Mexico	2000612	Ethics - Local	Comite de Etica en Investigacion Winsett Rethman SA de CV
Latin America	Mexico	2000613	Ethics - Local	Comite de Etica de la Facultad de Medicina de la UANL y Hosp. Univ. Dr. Jose Eleuterio González
Latin America	Peru	2000618	Ethics - Local	Comite de Bioetica de la Red Asistencial Sabogal - EsSalud
Latin America	Peru	2000621	Ethics - Local	Comite Institucional de Etica en la Investigacion del Hospital Nacional Cayetano Heredia
Latin America	Peru	2000622	Ethics - Local	Comité de Etica en Investigación - Arequipa
North America	Canada	2000517	Ethics - Local	McGill University Health Center Montreal Hospital
North America	Canada	2000518	Ethics - Local	Ottawa Hospital Research Ethics Board

Region	Country	Site number	Agency type	Agency
North America	Canada	2000662	Ethics - Local	UBC Clinical Research Ethics Board
North America	USA	2000491	Ethics - Local	Henry Ford Health System Institutional Review Board
North America	USA	2000492	Ethics - Local	Johns Hopkins Medicine Office of Human Subjects Research Institutional Review Board
North America	USA	2000493	Ethics - Local	University of Chicago Hospitals Institutional Review Board
North America	USA	2000494	Ethics - Local	University of Maryland Human Research Protections Office
North America	USA	2000495	Ethics - Local	UT Southwestern IRB
North America	USA	2000496	Ethics - Local	UCSF Human Research Protection Program
North America	USA	2000498	Ethics - Local	Wayne State University IRB
North America	USA	2000501	Ethics - Local	Baylor College of Medicine IRB
North America	USA	2000502	Ethics - Local	Missouri Baptist Medical Center Institutional Review Board
North America	USA	2000509	Ethics - Local	UCDHS IRB
North America	USA	2000510	Ethics - Local	Kansas City Veterans Administration Medical Center
North America	USA	2000511	Ethics - Local	Vanderbilt Institutional Review Board
North America	USA	2000513	Ethics - Local	LSU-HSC-S IRB
North America	USA	2000514	Ethics - Local	Weill Cornell Medical College Institutional Review Board
North America	USA	2000515	Ethics - Local	Cleveland Clinic Institutional Review Board
North America	USA	2000516	Ethics - Local	University of Texas MD Anderson Cancer Center Institutional Review Board

Region	Country	Site number	Agency type	Agency
North America	USA	2003421	Ethics - Local	University of California Irvine Institutional Review Board
North America	USA	2003451	Ethics - Local	Mayo Clinic Institutional Review Board
North America	USA	2003524	Ethics - Local	University of Pennsylvania Institutional Review Board
North America	USA	2003601	Ethics - Local	SUNY Buffalo
Asia Pacific	Japan	2000669	Ethics - Local	Kyoto University Hospital Institutional Review Board
Asia Pacific	Japan	2000711	Ethics - Local	Tokyo Women's Medical University Hospital Institutional Review Board
Asia Pacific	Japan	2000846	Ethics - Local	Yamaguchi University Hospital IRB
Asia Pacific	Japan	2001081	Ethics - Local	National University Corporation Tohoku University Tohoku University Hospital IRB
Asia Pacific	Japan	2001128	Ethics - Local	Aomori Prefectural Central Hospital Institutional Review Board
Asia Pacific	Japan	2001230	Ethics - Local	University of Tsukuba Hospital Institutional Review Board
Asia Pacific	Japan	2002885	Ethics - Local	Ebara Hospital Institutional Review Board
Asia Pacific	Japan	2003213	Ethics - Local	Juntendo University Hospital IRB
Asia Pacific	Japan	2003552	Ethics - Local	National Hospital Organization Hokkaido Medical Center Institutional Review Board
Asia Pacific	Japan	2003605	Ethics - Local	Southern TOHOKU Research Institute for Neuroscience Southern TOHOKU General Hospital IRB
Asia Pacific	Korea, Republic of	2000601	Ethics - Local	National Cancer Center IRB
Asia Pacific	Korea, Republic of	2000602	Ethics - Local	Samsung Medical Center Institutional Review Board
Asia Pacific	Korea, Republic of	2000603	Ethics - Local	Seoul National University Hospital IRB

Region	Country	Site number	Agency type	Agency
Asia Pacific	Korea, Republic of	2000604	Ethics - Local	Keimyung University Dongsan Hospital IRB
Asia Pacific	Korea, Republic of	2000605	Ethics - Local	Konkuk University Medical Center Institutional Review Board
Asia Pacific	Taiwan, Province of China	2000643	Ethics - Local	Institutional Review Board of National Cheng Kung University Hospital
Asia Pacific	Taiwan, Province of China	2000644	Ethics - Local	Cheng-Hsin General Hospital Institutional Review Board
Asia Pacific	Taiwan, Province of China	2000645	Ethics - Local	Institutional Review Board, Changhua Christian Hospital
Asia Pacific	Taiwan, Province of China	2000646	Ethics - Local	Tzu Chi General Hospital Research Ethics Committee
Asia Pacific	Thailand	2000647	Ethics - Local	Office of The Khon Kaen University Ethics Committee in Human Research
Asia Pacific	Thailand	2000648	Ethics - Local	Research Ethics Committee, Faculty of Medicine, Chiang Mai University
Asia Pacific	Thailand	2000650	Ethics - Local	The Institutional Review Board of the Faculty of Medicine, Chulalongkorn University
Asia Pacific	Thailand	2000651	Ethics - Local	Siriraj Institutional Review Board
EMEA	Czech Republic	2000585	Ethics - Local	Eticka komise Krajska Zdravotni As Nemocnice Teplice Oz
EMEA	Israel	2000598	Ethics - Local	The Chaim Sheba Medical Center EC
EMEA	Israel	2000600	Ethics - Local	Barzilai Medical Center Local EC
EMEA	Israel	2002785	Ethics - Local	Tel Aviv Sourasky EC
EMEA	Israel	2002786	Ethics - Local	Hadassah University Hospital Local EC
EMEA	Russian Federation	2000632	Ethics - Local	Ethics Committee at City Clinical Hospital #3
EMEA	Russian Federation	2000633	Ethics - Local	Ethics Committee at Siberian Regional Medical Centre
EMEA	Russian Federation	2000634	Ethics - Local	Ethics Committee at Republic Clinical Hospital for Rehabilitation Treatment

Region	Country	Site number	Agency type	Agency
EMEA	Russian Federation	2000635	Ethics - Local	Ethics Committee at Bashkiria State Medical University
EMEA	Russian Federation	2000636	Ethics - Local	Ethics Committee at City Clinical Hospital #31
EMEA	Russian Federation	2000639	Ethics - Local	Ethics Committee at City Clinical Hospital #11
EMEA	Russian Federation	2000640	Ethics - Local	Ethics Committee at Omsk State Medical University
EMEA	Russian Federation	2003146	Ethics - Local	Ethics Committee at Siberian Regional Medical Centre
EMEA	Russian Federation	2003147	Ethics - Local	Ethics Committee at Research Center of Neurology of RAMS
EMEA	Russian Federation	2003745	Ethics - Local	Ethics Committee at Kirov City Hospital # 4
EMEA	Russian Federation	2003746	Ethics - Local	Ethics Committee at City Clinical Hospital a.n. Buyanov V. M.
EMEA	Serbia	2003142	Ethics - Local	LEC Clinical Centre Serbia
EMEA	Serbia	2003144	Ethics - Local	LEC Clinical Centre Nis
EMEA	Serbia	2003145	Ethics - Local	LEC General Hospital Uzice
EMEA	South Africa	2000641	Ethics - Local	University of Cape Town, Faculty of Health Sciences Human Research Ethics Committee
EMEA	South Africa	2000642	Ethics - Local	University of Stellenbosch, Health Research Ethics Committee
EMEA	Ukraine	2003736	Ethics - Local	CEQ of Branch Medical Center Cyber Clinic Spizhenko
EMEA	Ukraine	1-3PJSOU	Ethics - Local	Comission on Ethics Questions of Kyiv City Clinical Hospital #4
EMEA	Ukraine	1-GB3UGZ	Ethics - Local	Commission on Ethics Questions of Municipal Institution Odesa Regional Clinical Hospital
Latin America	Argentina	2000519	Ethics - Local	Comité Independiente de Etica en investigación clínica "Dr. Carlos A. Barclay
Latin America	Argentina	2000520	Ethics - Local	Comisión Conjunta de Investigación en Salud (CCIS)

Region	Country	Site number	Agency type	Agency
Latin America	Argentina	2000520	Ethics - Local	Comité Institucional de Evaluación de la Facultad de Ciencias Biomédicas de la Universidad Austral
Latin America	Brazil	2000523	Ethics - Local	Comitê de Ética em Pesquisa em Seres Humanos da Universidade de Passo Fundo
Latin America	Brazil	2000524	Ethics - Local	Comite de Etica em Pesquisa em do Hospital Moinhos de Vento/RS
Latin America	Brazil	2000525	Ethics - Local	Comitê de Ética em Pesquisa em Seres Humanos da Universidade de Passo Fundo
Latin America	Brazil	2000745	Ethics - Local	Comitê de Ética em Pesquisa em Seres Humanos da Universidade Federal de Minas Gerais
Latin America	Colombia	2000533	Ethics - Local	Comite de Etica en Investigacion Colegio Mayor de Nuestra Senora del Rosario
Latin America	Colombia	2000537	Ethics - Local	Comite de Etica e Investigacion Clinica de la Fundacion Cardioinfantil
Latin America	Mexico	2000606	Ethics - Local	Comité de Etica en Investigacion de Medica Sur Sociedad Anónima Bursátil de Capital Variable
Latin America	Mexico	2000607	Ethics - Local	Comite de Etica en Investigacion del Grupo Medico Camino SC
Latin America	Mexico	2000608	Ethics - Local	Comité de Ética en Investigación de Christus Muguerza del Parque S.A. de C.V.
Latin America	Mexico	2000609	Ethics - Local	Comite Institucional de Etica CRI y Comite Institucional de Investigacion CRI
Latin America	Mexico	2000610	Ethics - Local	Comite de ética del Antiguo Hospital Civil de Guadalajara Fray Antonio Alcalde
Latin America	Mexico	2000611	Ethics - Local	Comite de Etica en Investigacion del Hospital Central Doctor Ignacio Morones Prieto
Latin America	Mexico	2000612	Ethics - Local	Comite de Etica en Investigacion Winsett Rethman SA de CV

Region	Country	Site number	Agency type	Agency
Latin America	Mexico	2000613	Ethics - Local	Comite de Etica de la Facultad de Medicina de la UANL y Hosp. Univ. Dr. Jose Eleuterio González
Latin America	Peru	2000618	Ethics - Local	Comite de Bioetica de la Red Asistencial Sabogal - EsSalud
Latin America	Peru	2000621	Ethics - Local	Comite Institucional de Etica en la Investigacion del Hospital Nacional Cayetano Heredia
Latin America	Peru	2000622	Ethics - Local	Comité de Etica en Investigación - Arequipa
North America	Canada	2000517	Ethics - Local	McGill University Health Center Montreal Hospital
North America	Canada	2000518	Ethics - Local	Ottawa Hospital Research Ethics Board
North America	Canada	2000662	Ethics - Local	UBC Clinical Research Ethics Board
North America	USA	2000491	Ethics - Local	Henry Ford Health System Institutional Review Board
North America	USA	2000492	Ethics - Local	Johns Hopkins Medicine Office of Human Subjects Research Institutional Review Board
North America	USA	2000493	Ethics - Local	University of Chicago Hospitals Institutional Review Board
North America	USA	2000494	Ethics - Local	University of Maryland Human Research Protections Office
North America	USA	2000495	Ethics - Local	UT Southwestern IRB
North America	USA	2000496	Ethics - Local	UCSF Human Research Protection Program
North America	USA	2000498	Ethics - Local	Wayne State University IRB
North America	USA	2000501	Ethics - Local	Baylor College of Medicine IRB
North America	USA	2000502	Ethics - Local	Missouri Baptist Medical Center Institutional Review Board
North America	USA	2000509	Ethics - Local	UCDHS IRB

Region	Country	Site number	Agency type	Agency
North America	USA	2000510	Ethics - Local	Kansas City Veterans Administration Medical Center
North America	USA	2000511	Ethics - Local	Vanderbilt Institutional Review Board
North America	USA	2000513	Ethics - Local	LSU-HSC-S IRB
North America	USA	2000514	Ethics - Local	Weill Cornell Medical College Institutional Review Board
North America	USA	2000515	Ethics - Local	Cleveland Clinic Institutional Review Board
North America	USA	2000516	Ethics - Local	University of Texas MD Anderson Cancer Center Institutional Review Board
North America	USA	2003421	Ethics - Local	University of California Irvine Institutional Review Board
North America	USA	2003451	Ethics - Local	Mayo Clinic Institutional Review Board
North America	USA	2003524	Ethics - Local	University of Pennsylvania Institutional Review Board
North America	USA	2003601	Ethics - Local	SUNY Buffalo

Supplementary Table S3: **Kinetics of B-cell and immunoglobulin depletion during the study.**

**Group**	**Start of RCP**	**End of RCP**	**OLP W26**	**OLP W52**	**OLP W78**	**OLP W104**	**OLP W130**	**OLP W156**
**Total CD20+ B cells, cells/μL**								
RCP placebo	157·48	171·41	0·55	0·58	0·44	0·45	0·30	0·28
1Q	101·35	118·40	0·20	0·1	0·15	0·27	0·1	0·1
3Q	232.17	255.19	3.97	4.93	3.27	2.67	1.45	0.43
RCP inebilizumab	184·02	1·26	0·83	0·70	0·59	0·52	0·39	0·33
1Q	107.88	0.42	0.32	0.24	0.1	0.21	0.21	0.10
3Q	280·22	6·22	4·35	3·55	4·07	1·62	1·18	1·08
p value	0·298	1·87e-27	0·229	0·515	0·861	1.00	0·831	0·380
**Plasma cell signature, FC from HD mean**								
RCP placebo	1·06	1·37	0·04					
1Q	0·59	0·64	0·02					
3Q	1·52	2·05	0·08					
RCP inebilizumab	0·93	0·05	0·06					
1Q	0·52	0·03	0·03					
3Q	1·41	0.21	0.33					
p value	0·339	2·95e-22	0·011					
**Total immunoglobulin, mg/dL**								
RCP placebo	1369	1380·5	1181	1106	1060·5	1043	986·5	913
1Q	1066·25	1198·5	1001	828·5	871·75	822·5	735	707·5
3Q	1626·75	1651·75	1406	1373·5	1256·5	1246	1191·5	1060
RCP inebilizumab	1305·5	1182·5	1123	1056·5	1015	997	946·5	936
1Q	1112·75	1020·25	926	898	815	825	785	732·5
3Q	1584·5	1436·5	1361	1290·5	1262	1229	1258·5	1180
p value	0·498	2·09e-04	0·562	0·635	0·723	0·828	0·782	0·707
**Immunoglobulin G, mg/dL**								
RCP placebo	1060	1035	919	901	889	844	822·5	743
1Q	828	889·25	733	678	679·25	647·5	607·75	581·5
3Q	1190	1250	1110	1035	1015	1020	931·25	904·5

RCP inebilizumab	981	922·5	895	862	845	815	793·5	781
1Q	840·25	813·75	759·25	738·25	683	666	654·25	617·25
3Q	1212·5	1142·5	1105	1035	1030	1020	1007·5	987
p value	0·551	0·017	0·996	0·998	0·890	0·987	0·651	0·441
**Immunoglobulin M, mg/dL**								
RCP placebo	100	103	64	56	50·5	53	44·5	44
1Q	62·5	70·75	35	29·5	28	29	30·5	30·5
3Q	136·5	152·25	108	89	77·5	77	67·25	66·5
RCP inebilizumab	86·5	63	55	49·5	47	44	40·5	39
1Q	58·5	38	32·75	27·75	26	26	25	24
3Q	142	99·5	87	78	76	77	74	67·75
p value	0·703	9·365e-06	0·220	0·468	0·789	0·444	0·880	0·590
**Immunoglobulin A, mg/dL**								
RCP placebo	221	224·5	167	134	128·5	127	105·5	96
1Q	168·5	161·5	102	95·5	89·25	80	77·25	78·5
3Q	276·5	268	219	190	165·25	155·5	141·5	129·5
RCP inebilizumab	190	150·5	127·5	110·5	104	103	91	84·5
1Q	128	106	92·75	86	79	74	66·25	65
3Q	241	218	197·25	173	152	148	140·5	132·75
p value	0·027	2·095e-05	0·062	0·068	0·135	0·231	0·341	0·346
**Immunoglobulin E, mg/dL**								
RCP placebo	16·1	18·8	9·2	6·6	4·75	5·6	3·8	2·8
1Q	5·8	5·4	3·8	2·2	1·4625	0·75	0·75	0·75
3Q	40·8	52·025	26·4	18·2	16·7	13·05	6·325	6·9
RCP inebilizumab	14·6	8·6	5·5	4·8	3·9	4	3·9	3·2
1Q	4·4	3·25	1·9	0·75	0·75	0·75	0·75	0·75
3Q	54·675	32·25	19·35	17·9	15·35	18·1	15·925	14·85
p value	0·753	0·039	0·096	0·377	0·624	0·871	0·299	0·616

During the RCP, participants in the placebo group received placebo while participants in the inebilizumab received inebilizumab. During the OLP (which started after the end of the RCP), all participants received inebilizumab. By OLP W26, all participants had been receiving inebilizumab for at least 28 weeks (RCP placebo) and up to 54 weeks (RCP inebilizumab). Plasma cell signature was not measured after OLP W26. *p* values between RCP inebilizumab and RCP placebo participants were calculated using the Mann–Whitney U test. FC=Fold Change, HD=Healthy Donor, OLP=Open-Label Period, Q=Quartile, RCP=Randomised Controlled Period; W=Week.

Supplementary Table S4: **Decreases in NMOSD progression observed with long-term inebilizumab treatment.**

Adjudicated attacks

Time period	N	Number of participants with attack (%)	Total number of attacks	Total patient-years of observation	AAR	Lower 95% CI	Upper 95% CI
**ITT**							
Placebo RCP	56	22 (39%)	22	23	1·01	0·79	1·23
0-6 months after inebilizumab treatment start (1st dose)	225	29 (13%)	31	111	0·28	0·23	0·33
0·5–1·5 years after inebilizumab treatment start (2–3 doses)	213	14 (7%)	14	208	0·07	0·05	0·09
1·5–2·5 years after inebilizumab treatment start (4–5 doses)	197	11 (6%)	11	189	0·06	0·04	0·08
>2·5 years after inebilizumab treatment start (>5 doses)	168	6 (4%)	7	222	0·03	0·02	0·04
**Participants randomised to placebo during RCP**							
Placebo RCP	56	22 (39%)	22	23	1·12	0·86	1·38
0-6 months after inebilizumab treatment start (1st dose)	51	7 (14%)	9	25	0·35	0·23	0·48
0·5–1·5 years after inebilizumab treatment start (2–3 doses)	49	2 (4%)	2	48	0·04	0·01	0·07
1·5–2·5 years after inebilizumab treatment start (4–5 doses)	46	2 (4%)	2	41	0·05	0·01	0·08
>2·5 years after inebilizumab treatment start (>5 doses)	31	2 (6%)	3	41	0·07	0·03	0·12
**Participants randomised to inebilizumab during RCP**							
0–6 months after inebilizumab treatment start (1st dose)	174	22 (13%)	22	85	0·26	0·20	0·31
0·5–1·5 years after inebilizumab treatment start (2–3 doses)	164	12 (7%)	12	160	0·08	0·05	0·10
1·5–2·5 years after inebilizumab treatment start (4–5 doses)	151	9 (6%)	9	148	0·06	0·04	0·08
>2·5 years after inebilizumab treatment start (>5 doses)	137	4 (3%)	4	182	0·02	0·01	0·03

EDSS score worsening

Time period	N	Number of participants with EDSS score increase (%)	Total points increased	Total patient-years of observation	Rate/ year	Lower 95% CI	Upper 95% CI
**ITT**							
Placebo RCP	56	20 (36%)	28·5	21	1·66	1·26	2·05
0-6 months after inebilizumab treatment start (1st dose)	222	39 (18%)	44·0	107	0·43	0·36	0·51
0·5–1·5 years after inebilizumab treatment start (2–3 doses)	211	37 (18%)	34·5	206	0·17	0·14	0·20
1·5–2·5 years after inebilizumab treatment start (4–5 doses)	194	43 (22%)	33·5	183	0·18	0·15	0·22
>2·5 years after inebilizumab treatment start (>5 doses)	140	26 (19%)	30·5	204	0·15	0·12	0·19
**Participants randomised to placebo during RCP**							
Placebo RCP	56	20 (36%)	28·5	21	1·82	1·33	2·31
0–6 months after inebilizumab treatment start (1st dose)	50	7 (14%)	10·5	27	0·40	0·25	0·55
0·5–1·5 years after inebilizumab treatment start (2–3 doses)	49	12 (24%)	13·5	47	0·31	0·20	0·42
1·5–2·5 years after inebilizumab treatment start (4–5 doses)	44	9 (20%)	6·0	38	0·16	0·08	0·23
>2·5 years after inebilizumab treatment start (>5 doses)	30	5 (17%)	7·5	39	0·20	0·10	0·29
**Participants randomised to inebilizumab during RCP**							
0-6 months after inebilizumab treatment start (1st dose)	172	32 (19%)	33·5	80	0·43	0·35	0·51
0·5–1·5 years after inebilizumab treatment start (2–3 doses)	162	25 (15%)	21·0	159	0·13	0·10	0·16
1·5–2·5 years after inebilizumab treatment start (4–5 doses)	150	34 (23%)	27·5	145	0·19	0·15	0·23
>2·5 years after inebilizumab treatment start (>5 doses)	110	21 (19%)	23·0	166	0·14	0·11	0·17

New/enlarging T2 MRI lesions*

Time period	N	Number of participants with new/enlarging T2 MRI lesions (%)	Total number of lesions	Total patient-years of observation	Rate/ year	Lower 95% CI	Upper 95% CI
**Participants randomized to inebilizumab during RCP**							
0–6 months after inebilizumab treatment start (1st dose)	154	44 (29%)	89	73	1·58	1·31	1·85
0·5–1·5 years after inebilizumab treatment start (2–3 doses)	145	50 (34%)	101	132	0·98	0·81	1·15
1·5–2·5 years after inebilizumab treatment start (4–5 doses)	122	42 (34%)	85	112	0·76	0·61	0·90
>2·5 years after inebilizumab treatment start (>5 doses)	85	38 (45%)	70	122	0·7	0·55	0·86

Inpatient hospitalisations

Time period	N	Number of participants with hospitalisa tions (%)	Total number of hospitalisa tions	Total patient-years of observation	Rate/ year	Lower 95% CI	Upper 95% CI
**ITT**							
Placebo RCP	56	8 (14%)	10	23	0·53	0·28	0·78
0–6 months after inebilizumab treatment start (1st dose)	225	23 (10%)	32	111	0·33	0·25	0·41
0·5–1·5 years after inebilizumab treatment start (2–3 dose)	213	21 (10%)	34	208	0·17	0·13	0·21
1·5–2·5 years after inebilizumab treatment start (4–5 doses)	197	14 (7%)	18	189	0·10	0·07	0·12
>2·5 years after inebilizumab treatment start (>5 doses)	168	8 (5%)	11	222	0·05	0·03	0·07
**Participants randomised to placebo during RCP**							
Placebo RCP	56	8 (14%)	10	23	0·54	0·28	0·80
0–6 months after inebilizumab treatment start (1st dose)	51	5 (10%)	14	25	0·55	0·29	0·81
0·5–1·5 years after inebilizumab treatment start (2–3 doses)	49	5 (10%)	7	48	0·15	0·07	0·24

1·5–2·5 years after inebilizumab treatment start (4–5 doses)	46	4 (9%)	5	41	0·12	0·05	0·19
>2·5 years after inebilizumab treatment start (>5 doses)	31	1 (3%)	2	41	0·05	0·00	0·09
**Participants randomised to inebilizumab during RCP**							
0–6 months after inebilizumab treatment start (1st dose)	174	18 (10%)	18	85	0·25	0·17	0·32
0·5–1·5 years after inebilizumab treatment start (2–3 doses)	164	16 (10%)	27	160	0·17	0·12	0·22
1·5–2·5 years after inebilizumab treatment start (4–5 doses)	151	10 (7%)	13	148	0·09	0·06	0·12
>2·5 years after inebilizumab treatment start (>5 doses)	137	7 (5%)	9	182	0·05	0·03	0·08

All participants during the first dosing period of inebilizumab treatment (first dosing interval) and yearly afterwards divided by RCP randomisation and ITT.
*T2 MRI lesions in placebo RCP participants and in the combined ITT group could not be included in this analysis because MRI data during the OLP was only recorded annually and RCP placebo participants were on a schedule 6 months behind RCP inebilizumab participants.
AAR=Annualised Attack Rate, CI=Confidence Interval, EDSS=Expanded Disability Status Scale, ITT=Intention-to-treat, MRI=Magnetic Resonance Imaging, NMOSD=Neuromyelitis Optica Spectrum Disorder, RCP=Randomised Controlled Period.

Supplementary Table S5: **6-month B-cell counts versus long-term disease activity in participants divided by RCP treatment group.**

		RR (95% CI)	Number of participants	Total patient-years of observation	*p*
**Participants randomised to placebo during RCP**					
Annualised attack rate	Log_{10} memory B cells	0·95 (0·16–5·75)	37	103	0·955
	Log_{10} 6-month CD20+ B cells	1·44 (0·45–4·62)	43	112	0·541
Rate of new/enlarging T2 MRI lesions	Log_{10} memory B cells	0·91 (0·58–1·44)	33	75	0·689
	Log_{10} 6-month CD20+ B cells	0·97 (0·68–1·39)	37	81	0·864
EDSS worsening rate	Log_{10} memory B cells	3·86 (0·95–15·72)	37	99	0·060
	Log_{10} 6-month CD20+ B cells	2·65 (0·85–8·27)	43	108	0·093
Inpatient hospitalisation rate	Log_{10} memory B cells	2·23 (0·54–9·19)	37	105	0·267
	Log_{10} 6-month CD20+ B cells	1·55 (0·51–4·73)	43	114	0·445
**Participants randomised to inebilizumab during RCP**					
Annualised attack rate	Log_{10} memory B cells	2·22 (1·15–4·27)	133	424	0·017
	Log_{10} 6-month CD20+ B cells	1·58 (0·99–2·52)	154	470	0·053
Rate of new/enlarging T2 MRI lesions	Log_{10} memory B cells	2·27 (1·59–3·24)	112	317	6·460e-06
	Log_{10} 6-month CD20+ B cells	1·8 (1·41–2·29)	129	351	1·850e-06
EDSS worsening rate	Log_{10} memory B cells	1·47 (0·89–2·42)	132	409	0·133
	Log_{10} 6-month CD20+ B cells	1·15 (0·8–1·65)	153	454	0·455
Inpatient hospitalisation rate	Log_{10} memory B cells	1·84 (0·93–3·65)	133	428	0·082
	Log_{10} 6-month CD20+ B cells	1·63 (0·99–2·68)	154	474	0·054

Negative binomial regression analysis of B-cell counts at 6 months post-treatment (W28 RCP for participants treated with inebilizumab who completed the RCP, W26 OLP for participants randomised to placebo and participants treated with inebilizumab who transitioned into OLP early) versus the annualised attack rate, rate of new/enlarging T2 lesions, EDSS worsening (participants/year), and inpatient hospitalisations that occurred after the first dosing period of inebilizumab treatment. CI=Confidence Interval, EDSS=Expanded Disability Status Scale, MRI=Magnetic Resonance Imaging, RCP=Randomised Controlled Period, RR=Rate Ratio, W26=Week 26 After Treatment Initiation, W28=Week 28 After Treatment Initiation.

SUPPLEMENTARY REFERENCES

1. Wingerchuk DM, Lennon VA, Pittock SJ, Lucchinetti CF, Weinshenker BG. Revised diagnostic criteria for neuromyelitis optica. *Neurology* 2006; **66**: 1485-9.

2. Bedi GS, Brown AD, Delgado SR, Usmani N, Lam BL, Sheremata WA. Impact of rituximab on relapse rate and disability in neuromyelitis optica. *Mult Scler* 2011; **17**: 1225-30.

3. Costanzi C, Matiello M, Lucchinetti CF, et al. Azathioprine: tolerability, efficacy, and predictors of benefit in neuromyelitis optica. *Neurology* 2011; **77**: 659-66.

4. Jacob A, Weinshenker BG, Violich I, et al. Treatment of neuromyelitis optica with rituximab: retrospective analysis of 25 patients. *Arch Neurol* 2008; **65**: 1443-8.

5. Kim SH, Kim W, Li XF, Jung IJ, Kim HJ. Repeated treatment with rituximab based on the assessment of peripheral circulating memory B cells in patients with relapsing neuromyelitis optica over 2 years. *Arch Neurol* 2011; **68**: 1412-20.

6. Pittock SJ, Lennon VA, McKeon A, et al. Eculizumab in AQP4-IgG-positive relapsing neuromyelitis optica spectrum disorders: an open-label pilot study. *Lancet Neurol* 2013; **12**: 554-62.
