# Supplementary material for: Association between B-cell depletion and attack risk in neuromyelitis optica spectrum disorder: An exploratory analysis from N-MOmentum, a double-blind, randomised, placebo-controlled, multicentre phase 2/3 trial
Source: eBioMedicine. 2022 Nov 10;86:104321. doi: 10.1016/j.ebiom.2022.104321 (PMC9664402; doi:10.1016/j.ebiom.2022.104321)
Supplement: Study Protocol [file mmc3.pdf]

A Double-masked, Placebo-controlled Study with Open-label Period to Evaluate the Efficacy and Safety of MEDI-551 in Adult Subjects with Neuromyelitis Optica and Neuromyelitis Optica Spectrum Disorders

Sponsor Protocol Number: CD-IA-MEDI-551-1155

Application Number: IND 117295
EudraCT number: 2014-000253-36

Investigational Product: MEDI-551

Phase: 2/3

Sponsor: MedImmune, LLC, a wholly owned subsidiary of AstraZeneca PLC, One MedImmune Way, Gaithersburg, Maryland, 20878, USA

Medical Monitor: John Ratchford, MD
Director, Clinical Development, Viela Bio, Inc.
Phone: 1-240-920-0079; Fax: 1-240-772-9578

Protocol History, Date: Original Protocol, 13Jan2014
Amendment 1, 01Jul2014
Administrative Change 1, 18Feb2015
Amendment 2, 10Dec2015
Amendment 3, 18Oct2016
Amendment 4, 08Mar2017
Amendment 5, 16Jul2018
Amendment 6, 11Oct2018

Protocol Synopsis

TITLE

A Double-masked, Placebo-controlled Study with Open-label Period to Evaluate the Efficacy and Safety of MEDI-551 in Adult Subjects with Neuromyelitis Optica and Neuromyelitis Optica Spectrum Disorders

HYPOTHESES

The primary hypothesis for this study is that by depleting cluster of differentiation (CD)19+ B cells including plasmablasts and plasma cells, MEDI-551 is effective in reducing the risk of neuromyelitis optica/neuromyelitis optica- spectrum disorders (NMO/NMOSD) attack compared to placebo, thus effectively reducing the risk of irreversible disability caused by such attacks.

Secondary Hypotheses:

- Depletion of CD19+ B cells by MEDI-551 reduces worsening in Kurtzke Expanded Disability Severity Scale (EDSS) and decreases the loss of low-contrast visual acuity in adult subjects with NMO/NMOSD.
- Depletion of CD19+ B cells by MEDI-551 reduces NMO/NMOSD-related in-patient hospitalizations.
- Depletion of CD19+ B cells by MEDI-551 reduces the cumulative number of active lesions found on magnetic resonance imaging (MRI).

OBJECTIVES:

Primary Objective: To compare the efficacy of MEDI-551 versus placebo in reducing the risk of an NMO/NMOSD attack in subjects with NMO/NMOSD.

Secondary Objectives:

1. To compare the efficacy of MEDI-551 versus placebo on the reduction of EDSS worsening in subjects with NMO/NMOSD.
2. To compare the efficacy of MEDI-551 versus placebo on the change from baseline of low-contrast visual acuity score in subjects with NMO/NMOSD.
3. To compare the efficacy of MEDI-551 versus placebo in reducing the cumulative active MRI lesion count (new gadolinium [Gd]-enhancing or new/enlarging T2).
4. To compare the efficacy of MEDI-551 versus placebo in reducing NMO/NMOSD-related in-patient hospitalizations in subjects with NMO/NMOSD.
5. To characterize the long-term efficacy of MEDI-551 by means of annualized attack rate.
6. To evaluate the safety and tolerability of a single course of MEDI-551 in subjects with NMO/NMOSD in the Randomized-controlled Period (RCP) and repeated doses of MEDI-551 in the Open Label Period (OLP).
7. To characterize the pharmacokinetic (PK) profile and immunogenicity of MEDI-551 in NMO/NMOSD subjects.

Exploratory Objectives:

1. To compare the effect of MEDI-551 versus placebo on health-related quality of life (HRQoL) as measured by the 4-week recall Short Form-36 (SF-36) Health Survey physical component score (PCS) and mental component score (MCS) in NMO/NMOSD subjects.
2. To compare the effect of MEDI-551 versus placebo on pain as measured using the pain numeric rating scale (NRS).
3. To characterize the pharmacodynamic (PD) profile (B cells and plasma cell signature) of MEDI-551 in NMO/NMOSD subjects.

4. To compare the effect of MEDI-551 versus placebo on aquaporin-4-antibody (AQP4-IgG) titer.
5. To compare the effect of MEDI-551 versus placebo on soluble biomarkers (eg, cytokines, chemokines, and immunoglobulins) and genomic (ribonucleic acid [RNA; microRNA]) biomarkers and other relevant cells (eg, T cells, astrocytes) in NMO/NMOSD subjects.

STUDY ENDPOINTS

Primary Endpoint: The primary endpoint is the time (days) from Day 1 to onset of an Adjudication Committee (AC)-determined NMO/NMOSD attack on or before Day 197. The definition of an NMO/NMOSD attack is the presence of a new symptom(s) or worsening of an existing symptom(s) related to NMO/NMOSD that meets at least ONE of the protocol-defined criteria for an NMO/NMOSD attack.

Secondary Endpoints:

Endpoints 1, 2, 3, and 4 are key secondary endpoints to be considered for studywise Type I error control.

1. Worsening from baseline in EDSS at last visit during the RCP.
2. Change from baseline in low-contrast visual acuity binocular score measured by low-contrast Landolt C Broken Rings Chart, at last visit during RCP.
3. Cumulative total active MRI lesions (new Gd-enhancing or new/enlarging T2) during the RCP.
4. Number of NMO/NMOSD-related in-patient hospitalizations. In-patient hospitalization is defined as more than an overnight stay.
5. Annualized attack rate (total number of AC-determined NMO/NMOSD attacks normalized by personyears-) during any exposure to MEDI-551.
6. Treatment-emergent adverse events (TEAEs), including treatment-emergent serious adverse events (TESAEs). Laboratory measurements as well as their changes or shift from baseline over time.
7. Pharmacokinetic profile of MEDI-551.
8. Incidence of anti-drug antibodies (ADAs) directed against MEDI-551 for the duration of the study, both predose and postdose for each subject.

Exploratory Endpoints:

1. Change from baseline in the 4-week recall SF-36 PCS and MCS at the last visit during the RCP.
2. Change from baseline in pain NRS in 5 locations at the last visit during the RCP.
3. B-cell counts (total and subsets).
4. Change from baseline in plasma cell gene signature.
5. Serum AQP4-IgG titers.

STUDY DESIGN

This is a multicenter, multinational, randomized, double-masked, placebo-controlled- study with an open-label extension period to evaluate the efficacy and safety of intravenous (IV) MEDI-551 in adult subjects with AQP4-IgG seropositive and seronegative NMO and NMOSD. Enrollment of subjects into the study will stop when a total of 67 AC-determined NMOSD attacks have occurred, when 252 subjects have been randomized and dosed, or following a recommendation by the independent Data Monitoring Committee (DMC) to stop enrollment, whichever occurs first. Following a screening period of up to 28 days, subjects will be randomized in a 3:1 ratio to receive IV MEDI-551 (300 mg at Day 1 and 300 mg at Day 15) or placebo for a period of 197 days (RCP).

Subjects who complete the RCP without experiencing an NMO/NMOSD attack will be given the option to enroll into an OLP and will initiate or continue treatment with MEDI-551. Subjects who experience an AC-determined NMO/NMOSD attack during the RCP will be given the option to enroll- into the OLP following

rescue therapy. Subjects for whom the NMO/NMOSD attack is not determined by the AC will continue in the RCP until Day 197 (or until another attack occurs that is determined by the AC).

The OLP will continue for a minimum of 1 year and a maximum of 3 years after the last subject enters the OLP, or until regulatory approval for MEDI-551 as treatment for NMO in the participating country, or until the Sponsor discontinues development of MEDI-551 in this indication, whichever occurs first. Subjects can choose to exit the OLP at any time for any reason, including seeking alternative treatment options, at which point they will enter the Safety Follow-up Period (SFP; unless consent is withdrawn).

All subjects will continue in the SFP for a total of 12 months from last dose to evaluate the long-term safety of the investigational product.

Screening Period

Subjects with the diagnosis of NMO/NMOSD will be screened over a 28-day period to establish their eligibility to participate in the study based on the inclusion and exclusion criteria. AQP4-IgG serostatus will be determined by the central laboratory. For subjects who are found to be AQP4-IgG negative in the screening period, relevant data documenting their NMO disease will be assessed by an independent Eligibility Committee to ensure that the data are consistent with the diagnosis of NMO. All subjects who fulfill eligibility criteria will then be randomized into the study.

Subjects undergoing screening at the time when the 252nd subject is randomized and dosed, subjects in screening at the time the 67th AC-determined attack is confirmed, or subjects undergoing screening at the time enrollment is terminated for any other reason, will not be randomized.

Randomization (Day 1)

Two hundred and fifty-two subjects will be randomized into the study in a 3:1 ratio to receive IV MEDI-551 or placebo as described below. Randomization will occur on Day 1 and will be stratified by AQP4-IgG serostatus (in a ratio of approximately 80:20 seropositive and seronegative subjects, respectively) and by region (Japan vs non-Japan).

Randomized-controlled Period (Day 1 to Day 197)

Following randomization on Day 1, subjects will be treated with MEDI-551 or placebo on Day 1 and Day 15. An oral corticosteroid course will be initiated on Day 1 (prednisone 20 mg/day or equivalent oral glucocorticoid) and continue until Day 14. Tapering the oral corticosteroids will occur from Day 15 to Day 21. By Day 21, tapering must be completed.

The planned duration of the RCP for each subject will be 197 days. All subjects who complete the RCP without experiencing an NMO/NMOSD attack will be given the option to enter the OLP. Subjects who are in the RCP when a total of 67 AC-determined NMOSD attacks have occurred or subjects who are in the RCP when enrollment is discontinued upon recommendation of the independent DMC based on evidence of efficacy and safety, will discontinue the RCP as soon as possible, preferably within 14 days, and be given the option to enter the OLP.

Subjects will be monitored for new or worsening symptoms related to NMO/NMOSD during scheduled study visits and with follow-up phone calls every 2 weeks between study visits (or if a scheduled visit is missed).

When a possible new or worsening symptom(s) related to NMO/NMOSD is identified, subjects will be required to inform the site. If an Assessment Visit is deemed necessary, this must be scheduled as soon as possible but within 72 hours of reporting of the symptom to the site. At the Assessment Visit, subjects will initially undergo evaluations to determine if the symptoms are related to NMO/NMOSD; if related, the subjects will undergo further evaluations to determine if the symptoms meet at least ONE of the protocol-defined criteria for an NMO/NMOSD attack. In cases where a new or worsening symptom(s) does not meet at least one of the protocol-defined criteria for an NMO/NMOSD attack, the subject will continue in the RCP.

Assessment of new symptoms or worsening of existing symptoms should be completed within 5 days to determine if an attack has occurred. Treatment of an attack should preferably be initiated after completion of the attack assessment and the determination that the protocol attack criteria have been met. However, the Principal Investigator can initiate rescue therapy at any time before full assessment is completed. Rescue therapy will be given as directed by the investigator. This may include IV corticosteroids, intravenous immunoglobulin (IVIG), and/or plasma exchange (PLEX).

Upon completion of the Assessment Visit, the complete set of data generated from the assessments will be sent to the AC regardless of whether an NMO/NMOSD attack was diagnosed according to the protocol criteria by the Principal Investigator. The adjudication process will be completed within 14 days (+ 3 days) from initiation of the Assessment Visit and the AC determination will be communicated to the Principal Investigator.

Subjects for whom the diagnosis of an NMO/NMOSD attack is not determined by the AC will be given the option to continue in the RCP until Day 197. Subjects for whom the diagnosis of an NMO/NMOSD attack is determined by the AC will be given the option to enter the OLP. In addition, subjects who experience an NMO/NMOSD attack that requires rescue treatment and meets the protocol-defined criteria, regardless of the outcome of the AC review, will undergo an Attack Follow-up Visit 28 days from Day 1 of the Assessment Visit. This visit may correspond with an OLP or SFP visit or may be scheduled separately.

If subjects do not wish to enter the OLP or wish to leave the RCP at any point, they will continue to the SFP (unless consent is withdrawn).

Enrollment of subjects into the study will stop when a total of 67 AC-determined NMOSD attacks have occurred, when 252 subjects have been randomized and dosed, or following a recommendation by the independent Data Monitoring Committee (DMC) to stop enrollment, whichever occurs first.

Open-label Period

Subjects will be given the option to enter the OLP if they 1) complete 197 days of the RCP, or 2) experience an AC-determined NMO/NMOSD attack during the RCP, 3) are in the RCP at the time that 67 AC-determined attacks have occurred, or 4) are in the RCP at the time the Sponsor discontinues enrollment upon recommendation of the independent DMC based on evidence of efficacy and safety.

Subjects who discontinue the RCP for reasons other than one of these four mentioned above will not be eligible for the OLP. Reasons for subjects not entering the OLP will be captured. These subjects will be followed for safety in the SFP.

The first day of the OLP will be Day 1 (OLP Day 1). Upon entering the OLP, subjects will receive MEDI-551. During the OLP, subjects will be followed at scheduled study visits and will continue on MEDI-551 therapy. The OLP will continue for minimum of 1 year and a maximum of 3 years (after the last subject enters), or until regulatory approval for MEDI-551 in the participating country, or until the Sponsor discontinues development of MEDI-551 in this indication, whichever occurs first. Subjects can choose to exit the OLP at any time for any reason, including seeking alternative treatment options, at which point they will enter the SFP (unless consent is withdrawn).

Subjects will be followed for NMO/NMOSD attacks in the same fashion as in the RCP and events will be centrally adjudicated.

Safety Follow-up Period

The SFP will start when a subject prematurely discontinues from the RCP or OLP. The length of the SFP will be determined by the time elapsed from the time of the last dose of the investigational product to the time of the premature discontinuation, to complete a total of 52 weeks. During the SFP, the subject will be monitored for adverse events/serious adverse events, B-cell levels, ADAs, and immunoglobulin levels. During the SFP, a subject may receive standard treatment for their NMO/NMOSD at the discretion of the investigator.

TARGET SUBJECT POPULATION

The subjects in this study will be adult women and men (aged 18 years and above) with a diagnosis of NMO or NMOSD at the time of screening and a documented history of 1 or more NMO/NMOSD acute relapses that required rescue therapy within the last year or 2 or more NMO/NMOSD acute relapses that required rescue therapy within the 2 years prior to screening. Both AQP4-IgG seropositive and seronegative subjects will be enrolled in the study to fully capture the entire spectrum of this potentially fatal and rare demyelinating neurological disease.

INVESTIGATIONAL PRODUCT, DOSAGE, AND MODE OF ADMINISTRATION

Randomized-controlled Period

- Treatment Arm 1: 300 mg IV MEDI-551 on Day 1 and Day 15
- Treatment Arm 2: IV Placebo on Day 1 and Day 15

Additionally, all subjects entering the RCP will be treated for 2 weeks (Day 1 to Day 14) with oral corticosteroids (prednisone 20 mg/day or equivalent oral glucocorticoid). A tapering schedule will be implemented from Day 15 to Day 21.

Open-label Period

Subjects randomized to **Treatment Arm 1** during the RCP will receive:

- 300 mg IV MEDI-551 on OLP Day 1, masked IV placebo on OLP Day 15, then 300 mg IV MEDI-551 every 26 weeks (Q26W) thereafter

Subjects randomized to **Treatment Arm 2** during the RCP will receive:

- 300 mg IV MEDI-551 on OLP Day 1, masked 300 mg IV MEDI-551 on OLP Day 15, then 300 mg IV MEDI-551 Q26W thereafter

Dosing of subjects enrolling into the OLP following an adjudicated NMO/NMOSD attack, following the occurrence of the 67th adjudicated NMO/NMOSD attack, or following termination of enrollment upon recommendation of the independent DMC based on evidence of efficacy and safety, will follow the OLP dosing regimen described above.

For both the RCP and the OLP, investigational product will be administered as a 90-minute IV infusion via an infusion pump. All subjects will be premedicated on Day 1 and Day 15 (OLP Day 1 and OLP Day 15) and at subsequent dosing visits with IV methylprednisolone (100 mg or equivalent glucocorticoid), oral (PO) diphenhydramine (25-50 mg or equivalent antihistamine), and PO paracetamol (acetaminophen; 500-650 mg) prophylactically to prevent infusion reactions.

STATISTICAL ANALYSIS PLAN:

The primary analysis of time to onset of AC-determined attack employs a sequential testing procedure that initially compares treatment groups within AQP4-IgG seropositive subjects, and if statistically significant (two-sided $\alpha = 0.05$), further compares treatment groups (two-sided $\alpha = 0.05$) within the broader Intent-to-Treat (ITT) population, defined as all subjects who are randomized and receive at least one dose of investigational product. Furthermore, these analyses will analyze subjects according to the initial randomization regardless of actual treatment received. The treatment effect in the seropositive cohort will be assessed using the Cox proportional hazards model with treatment indicator (MEDI-551 or placebo) as an explanatory factor; whereas for the ITT population, the model will also include serostatus as an additional explanatory factor. Subjects who complete Day 197 of the RCP or discontinue the study before Day 197 for reasons other than an AC-determined- NMO/NMOSD attack will be censored in this model at the time of the Day 197/discontinuation visit. An evaluation of consistency of treatment effect in Japanese subjects with that observed in the global study will be determined. The data cutoff for the primary analysis will be when the last subject completes the discontinuation visit following the 67th AC-determined attack, after all subjects complete the RCP if 67 AC-determined attacks do not occur, or when the last subject completes the discontinuation visit following discontinuation of enrollment upon recommendation of the independent DMC

based on evidence of efficacy and safety. Subjects who are in the RCP when a total of 67 AC-determined NMOSD attacks have occurred, or when enrollment is discontinued upon recommendation of the independent DMC based on evidence of efficacy and safety, will discontinue the RCP as soon as possible, preferably within 14 days.

Control of Type I Error

The study is designed to strongly control the overall Type I error rate of $\alpha = 0.05$. The primary null hypothesis will be hierarchically tested first at $\alpha = 0.05$ in the seropositive cohort, and if significant, it will be further tested in the ITT population at $\alpha = 0.05$. If, and only if, the treatment group comparison is statistically significant within the ITT population, the secondary hypotheses will be tested. Null hypotheses for the 4 key secondary endpoints will follow the same sequential testing strategy as the primary analysis (testing within seropositive subjects first, followed by the ITT population if the comparison within seropositive subjects is statistically significant). Each secondary hypothesis will be initially tested based on the Bonferroni method at two-sided α=0.05/4 = 0.0125. If the null hypothesis for a particular secondary endpoint is rejected across both seropositive and ITT populations, the Type I error saved will be propagated equally to other non-rejected secondary null hypotheses. The testing procedure will be repeated until all null hypotheses are rejected or no further null hypothesis can be rejected.

The safety data will be presented using the as-treated population, which will include all subjects who receive any investigational product analyzed according to treatment received. Specifically, subjects randomized to MEDI-551 who received all placebo doses will be included in the placebo group for safety analyses; conversely, subjects who received at least one dose of MEDI-551 will be included in the active treatment group for safety analyses. Efficacy and safety data from the OLP will be presented based on subjects who receive at least one dose during the OLP. Unless otherwise specified, all statistical inference will be performed at a significance level of two-sided $\alpha = 0.05$.

Sample Size and Power Calculations

The current study is being planned to detect a target relative reduction of 60% in risk for time from Day 1 to onset of AC-determined NMO/NMOSD attack on or before Day 197 with at least 90% power and $\alpha = 0.05$ (two-sided). A total of 67 AC-determined NMO/NMOSD attacks are required for the ITT population. Subjects will be randomized in a 3:1 ratio to receive either MEDI-551 or placebo within both AQP4-IgG seropositive and seronegative strata. The stratification ratio is anticipated to be approximately 80:20 with higher allocation to the seropositive cohort. If the seropositive cohort has 80% of the attacks, the study will have approximately 82% power to detect the target relative reduction of 60%.

Two hundred and fifty-two subjects will be randomized and dosed. This sample size is based on a blinded review of the attack rate for the first 78 subjects to complete the RCP and a simulation based on these subjects that indicates a > 90% probability of achieving the required 67 AC-determined attacks with 252 subjects.

Within each AQP4-IgG stratum, randomization will also be stratified by region (Japan versus non-Japan). The number of Japanese subjects will be determined primarily by feasibility and will not depend on a minimum number of NMO/NMOSD attacks to be observed from Japanese subjects. The study may conclude enrollment regardless of the number of subjects in Japan versus non-Japan regions.

Interim Analysis

An unmasked interim analysis will be conducted for futility assessment by an independent Data Monitoring Committee when approximately 34 AC-determined NMO/NMOSD attacks occur in this study. The study may be declared as “futile” if calculated predictive power at interim is < 20%.

TABLE OF CONTENTS

1 INTRODUCTION 18
1.1 Disease Background 18
1.2 MEDI-551 Background 19
1.3 Summary of Nonclinical Experience 20
1.4 Summary of Clinical Experience 21
1.5 Rationale for Study 22
1.6 Research Hypotheses 23
1.6.1 Primary Hypothesis 23
1.6.2 Secondary Hypotheses 23
2 OBJECTIVES 23
2.1 Objectives 23
2.1.1 Primary Objective 23
2.1.2 Secondary Objectives 23
2.1.3 Exploratory Objectives 24
2.2 Study Endpoints 24
2.2.1 Primary Endpoint 24
2.2.2 Secondary Endpoints 24
2.2.3 Exploratory Endpoint(s) 25
3 STUDY DESIGN 25
3.1 Description of the Study 25
3.1.1 Overview 25
3.1.1.1 Screening Period 28
3.1.1.2 Randomization (Day 1) 28
3.1.1.3 Randomized-controlled Period (Day 1 to Day 197) 28
3.1.1.4 Open-label Period 30
3.1.1.5 Safety Follow-up Period 30
3.1.2 Treatment Regimen 30
3.1.3 Management of Study Medication Related Toxicities 32
3.2 Study Design and Dose Rationale 35
3.2.1 Study Design Rationale 35
3.2.2 Dose Rationale 36
3.2.3 Rationale for Study Population 37
3.2.4 Rationale for Endpoints 39
3.2.4.1 Primary Endpoint 39
3.2.4.2 Secondary Endpoints 39
3.2.4.3 Exploratory Endpoints 41
3.2.4.4 Exploratory Investigations 43
4 MATERIALS AND METHODS 43
4.1 Subjects 43
4.1.1 Number of Subjects 43
4.1.2 Inclusion Criteria 44

4.1.3 Exclusion Criteria 46
4.1.4 Subject Enrollment and Randomization 49
4.1.4.1 Enrollment 49
4.1.4.2 Randomization Process 50
4.1.5 Withdrawal from the Study 50
4.1.6 Discontinuation of Investigational Product 50
4.1.7 Replacement of Subjects 51
4.1.8 Withdrawal of Informed Consent for Donated Biological Samples 51
4.2 Schedule of Study Procedures 52
4.2.1 Enrollment/Screening Period 52
4.2.1.1 Eligibility Committee 55
4.2.2 Treatment Period 55
4.2.2.1 Randomized-controlled Treatment Period 55
4.2.2.2 Open-label Period 59
4.2.3 Study Procedures for Assessment Visit 64
4.2.4 Managing Subjects With Worsening Attacks 67
4.2.5 Study Procedures for Attack Follow-up Visit 67
4.2.6 Safety Follow-up Period 68
4.3 Description of Study Procedures 69
4.3.1 Efficacy 69
4.3.1.1 Identification, Assessment, and Adjudication of an NMO/NMOSD Attack 69
4.3.1.2 Neuroaxis Magnetic Resonance Imaging Scan 79
4.3.1.3 Independent Ophthalmology Assessments 80
4.3.1.4 Expanded Disability Status Scale and Functional Systems Scores 84
4.3.1.5 Independent Adjudication Committee 84
4.3.1.6 Healthcare Resource Utilization 85
4.3.2 Patient-reported Outcomes 86
4.3.2.1 Pain NRS 86
4.3.2.2 Short Form-36 Health Survey 87
4.3.2.3 Collection of PRO Endpoints 87
4.3.3 Medical History and Physical Examination, Electrocardiogram, Weight, Vital Signs, and Chest X-ray 87
4.3.3.1 Medical History 87
4.3.3.2 Physical and Neurological Examination 88
4.3.3.3 Body Weight and Height 88
4.3.3.4 Electrocardiogram 88
4.3.3.5 Vital Signs 89
4.3.3.6 Chest X-ray 89
4.3.4 Columbia-Suicide Severity Rating Scale 89
4.3.5 Modified Rankin Scale 90
4.3.6 Clinical Laboratory Tests 91
4.3.7 Pharmacokinetic Evaluation and Methods 93
4.3.8 Immunogenicity Evaluation and Methods 93
4.3.9 Biomarker Evaluations and Methods 93
4.3.9.1 Exploratory Investigations 94
4.3.10 Estimate of Volume of Blood to Be Collected 95

4.4 Study Suspension or Termination 96
4.5 Investigational Products 97
4.5.1 Identity of Investigational Product(s) 97
4.5.1.1 Investigational Product Dose Preparation 98
4.5.1.2 Treatment Administration 100
4.5.1.3 Monitoring of Dose Administration 103
4.5.1.4 Reporting Product Complaints 103
4.5.2 Additional Study Medications 104
4.5.2.1 Oral Corticosteroid Use 104
4.5.2.2 Rescue Medications 104
4.5.3 Dose Adjustments and Missed Doses 105
4.5.4 Labeling 105
4.5.5 Storage 105
4.5.6 Treatment Compliance 105
4.5.7 Accountability 105
4.6 Treatment Assignment and Masking 106
4.6.1 Methods for Assigning Treatment Groups 106
4.6.2 Methods for Ensuring Masking 106
4.6.3 Methods for Reducing Bias 106
4.6.4 Methods for Unmasking 107
4.6.4.1 Unmasking in the Event of a Medical Emergency 108
4.6.4.2 Unmasking for Interim Analysis Purposes 108
4.6.4.3 Unmasking due to Known or Hypothesized Effects of MEDI-551 108
4.7 Restrictions During the Study and Concomitant Treatment(s) 109
4.7.1 Permitted Concomitant Medications 109
4.7.1.1 Immunosuppressive Medications During the Screening Period 109
4.7.2 Prohibited Concomitant Medications 110
4.8 Statistical Evaluation 111
4.8.1 General Considerations 111
4.8.2 Sample Size and Power Calculations 111
4.8.3 Efficacy 112
4.8.3.1 Primary Efficacy Analysis 112
4.8.3.2 Additional Analyses of the Primary Endpoint 113
4.8.3.3 Secondary Efficacy Analyses 114
4.8.3.4 Subgroup Analyses 114
4.8.4 Safety 114
4.8.4.1 Analysis of Adverse Events 114
4.8.4.2 Analysis of Clinical Laboratory Parameters, Physical Examination Findings, and Vital Sign Measurements 115
4.8.5 Patient-reported Outcomes 115
4.8.5.1 Short Form-36 Health Survey 115
4.8.5.2 Pain NRS 115
4.8.6 Analysis of Immunogenicity, Pharmacokinetics, and Exploratory Biomarkers 115
4.8.6.1 Analysis of Immunogenicity 115
4.8.6.2 Analysis of Pharmacokinetics 115
4.8.6.3 Analysis of Exploratory Biomarkers 116

4.8.7 Additional Analyses 116
4.8.8 Data Monitoring Committee 116
4.8.9 Control of Type I Error 117
4.8.10 Interim Analysis for Futility 117

5 ASSESSMENT OF SAFETY 117
5.1 Definition of Adverse Events 117
5.2 Definition of Serious Adverse Events 118
5.3 Definition of Adverse Events of Special Interest 119
5.4 Recording of Adverse Events 119
5.4.1 Time Period for Collection of Adverse Events 120
5.4.2 Follow-up of Unresolved Adverse Events 120
5.5 Reporting of Serious Adverse Events 120
5.6 Other Events Requiring Immediate Reporting 121
5.6.1 Overdose 121
5.6.2 Hepatic Function Abnormality 121
5.6.3 Progressive Multifocal Leukoencephalopathy 122
5.6.4 Pregnancy 122

6 STUDY AND DATA MANAGEMENT 123
6.1 Training of Study Site Personnel 123
6.2 Monitoring of the Study 123
6.2.1 Source Data 124
6.2.1.1 Direct Access to Source Data 124
6.2.2 Study Agreements 124
6.2.3 Archiving of Study Documents 124
6.3 Study Timetable and End of Study 125
6.3.1 Discontinuation or Suspension of the Study Program 126
6.3.2 Completion of the Study 126
6.4 Data Management 126
6.5 Medical Monitor Coverage 126

7 ETHICAL AND REGULATORY REQUIREMENTS 127
7.1 Ethical Conduct of the Study 127
7.2 Subject Data Protection 127
7.3 Ethics and Regulatory Review 127
7.3.1 Ethics and Regulatory Review in Japan 128
7.4 Informed Consent 128
7.5 Changes to the Protocol and Informed Consent Form 129
7.5.1 Deviation From the Clinical Study Protocol 129
7.6 Audits and Inspections 130

8 REFERENCES 131

9 CHANGES TO THE PROTOCOL 136

LIST OF IN-TEXT TABLES

Table 1 Randomized-controlled Period Treatment Regimen 31
Table 2 Open-label Period Treatment Regimen 31
Table 3 An Approach to Management of Anaphylactic, Hypersensitivity, and Infusion Reactions 33
Table 4 Highly Effective Methods of Contraception 45
Table 5 Schedule of Screening Procedures 53
Table 6 Schedule of Randomized-controlled Treatment Period Study Procedures. 56
Table 7 Schedule of Open-label Period Study Procedures 61
Table 8 Schedule of Study Procedures for the Assessment Visit 65
Table 9 Schedule of Study Procedures for the Attack Follow-up Visit 67
Table 10 Schedule of Procedures for Safety Follow-up Period 68
Table 11 Protocol-defined Criteria for an NMO/NMOSD Attack 73
Table 12 Pupil Response Scoring Criteria for Relative Afferent Pupillary Defect ... 83
Table 13 Healthcare Resource Utilization Information to be Collected and Frequency 86
Table 14 Estimate of Blood Volume to Be Collected 95
Table 15 Identification of Investigational Products 98
Table 16 Infusion Times for IV Administration of MEDI-551 300 mg or Placebo 102
Table 17 Open-label Cohort Studies in Neuromyelitis Optica 202
Table 18 Estimated Pretreatment Hazard Rate for Time to Relapse 202
Table 19 Calculated Yearly Constant Hazard Rate Based on Relapse-free Subjects in First 12 Months of Treatment 206
Table 20 Relative Reduction in Risk of Relapse Based on Calculated Hazard Rate From Azathioprine and Rituximab NMO Studies 207
Table 21 List of Null Hypotheses Considered Under Multiplicity Adjustment Procedure 210
Table 22 Alpha Allocation Rule 212
Table 23 Alpha Propagation Rule 212

LIST OF IN-TEXT FIGURES

Figure 1 Study Flow Diagram ... 27

Figure 2 PD Model Simulation Supporting Dosing Regimen ... 37

Figure 3 Flow Chart of Assessment and Diagnosis of an NMO/NMOSD Attack ... 69

Figure 4 Process for Assessing Symptoms Affecting the Eye ... 75

Figure 5 Process for Assessing Symptoms Affecting the Spinal Cord ... 76

Figure 6 Process for Assessing Symptoms Affecting the Brain or Brainstem ... 77

Figure 7 Neuromyelitis Optica Spectrum of Disorders Relapses Before and After Treatment with Azathioprine for 70 Patients with More Than 12 Months Follow Up ... 203

Figure 8 Attack Frequency Before, During, and After Eculizumab Treatment ... 204

Figure 9 Relapses in Patients with Neuromyelitis Optica Before and After Treatment with Rituximab ... 204

Figure 10 Disease Course of Patients Treated with Rituximab ... 205

Figure 11 Relapses in Patients with Neuromyelitis Optica Before and After Treatment with Rituximab ... 206

Figure 12 Estimated Predictive Power Against Observed Interim Treatment Effect When 50% of AC-determined NMO/NMOSD Attacks are Available ... 209

Figure 13 Multiplicity Adjustment Strategy ... 211

LIST OF APPENDICES

Appendix 1 Signatures ... 167

Appendix 2 National Institute of Allergy and Infectious Diseases and Food Allergy and Anaphylaxis Network Guidance for Anaphylaxis Diagnosis ... 170

Appendix 3 Additional Safety Guidance ... 171

Appendix 4 Kurtzke Expanded Disability Status Scale and Functional Systems Scores ... 173

Appendix 5 Pain NRS ... 184

Appendix 6 Short Form-36v2 ... 186

Appendix 7 Columbia-Suicide Severity Rating Scale: Baseline/Screening Version ... 193

Appendix 8 Columbia-Suicide Severity Rating Scale: Since Last Visit Version ... 197

Appendix 9 Sample Size Calculation ... 201

Appendix 10 Details of Futility Analysis ... 209

Appendix 11 Type I Error Control ... 210

Appendix 12 Assessment and Collection of Adverse Events ... 213

List of Abbreviations

Abbreviation or Specialized Term	Definition
AC	Adjudication Committee
ADA	anti-drug antibody
ADCC	antibody-dependent cellular cytotoxicity
ADCP	antibody-dependent cellular phagocytosis
AE	adverse event
AESI	adverse event of special interest
ALT	alanine transaminase
AQP4	aquaporin-4
AQP4-IgG/NMO-IgG	autoantibodies against aquaporin-4
AST	aspartate transaminase
AZA	azathioprine
βHCG	beta human chorionic gonadotropin
BAFF	B-cell activating factor
BCG	Bacillus of Calmette and Guérin
BP	blood pressure
CD	cluster of differentiation
CD19+	CD19 positive
CD20+	CD20 positive
CD20-	CD20 negative
CDC	complement-dependent cytotoxicity
CDER	Center for Drug Evaluation and Research
CF	counting fingers
CI	confidence interval
CNS	central nervous system
CSF	cerebrospinal fluid
C-SSRS	Columbia-Suicide Severity Rating Scale
DEHP	di(2-ethylhexyl) phthalate
DMC	Data Monitoring Committee
DNA	deoxyribonucleic acid
ECG	electrocardiogram
eCRF	electronic case report form
EDSS	(Kurtzke) Expanded Disability Status Scale
EDV	Early Discontinuation Visit

Abbreviation or Specialized Term	Definition
EFD	embryo-fetal development
ETDRS	Early Treatment Diabetic Retinopathy Study
EU	European Union
FAAN	Food Allergy and Anaphylaxis Network
FDA	Food and Drug Administration
FS	Functional Systems
FSS	Functional Systems Scores
FWER	family wise error rate
GCP	Good Clinical Practice
Gd	gadolinium
GFR	glomerular filtration rate
GMP	Good Manufacturing Practice
HbA1c	glycosylated hemoglobin
HBc	hepatitis B core antigen
HCRU	Healthcare Resource Utilization
HIPAA	Health Insurance Portability and Accountability Act
HIV	human immunodeficiency virus
HM	hand movement
HR	hazard ratio
HRQoL	health-related quality of life
huCD19 TG	human CD19 transgenic
IB	Investigator's Brochure
ICF	informed consent form
ICH	International Council for Harmonisation
IEC	Independent Ethics Committee
Ig	immunoglobulin
IgA/E/G/M	immunoglobulin A/E/G/M
IgG1	immunoglobulin G1
IgG1κ	immunoglobulin G1 kappa
INR	international normalized ratio
IRB	Institutional Review Board
ITT	Intent-to-Treat
IV	intravenous
IVIG	intravenous immunoglobulin

Abbreviation or Specialized Term	Definition
IVRS	interactive voice response system
IWRS	interactive web response system
JCV	polyomavirus John Cunningham
kDa	kilodalton
KM	Kaplan-Meier
LETM	longitudinally extensive transverse myelitis
LLN	lower limit of normal
LP	light perception
mAb	monoclonal antibody
MCS	mental component score
MedDRA	Medical Dictionary for Regulatory Activities
MHLW	Ministry of Health, Labour, and Welfare (Japan)
MMF	mycophenolate mofetil
MOG	myelin-oligodendrocyte glycoprotein
MRI	magnetic resonance imaging
mRNA	messenger ribonucleic acid
mRS	Modified Rankin Scale
MS	multiple sclerosis
MTD	maximum tolerated dose
NaCl	sodium chloride
NCI CTCAE	National Cancer Institute Common Terminology Criteria for Adverse Events
NIAID	National Institute of Allergy and Infectious Diseases
NK	natural killer
NLP	no light perception
NMO	neuromyelitis optica
NMOSD	neuromyelitis optica spectrum disorders
NOAEL	no observed adverse effect level
NRS	numeric rating scale
OCT	optical coherence tomography
OLP	Open-label Period
ON	optic neuritis
PCS	physical component score
PD	pharmacodynamic
PEF	peak expiratory flow

Abbreviation or Specialized Term	Definition
PLEX	plasmapheresis; plasma exchange
PK	pharmacokinetic
PML	progressive multifocal leukoencephalopathy
PO	oral
PRO	patient-reported outcome
PVC	polyvinyl chloride
Q12W	every 12 weeks
Q26W	every 26 weeks
RAPD	relative afferent pupillary defect
RCP	Randomized-controlled Period
RNA	ribonucleic acid
RNFL	retinal nerve fiber layer
SAE	serious adverse event
SC	subcutaneous
SF-36	Short Form-36 Health Survey
SF-36v2	Short Form-36 Health Survey, version 2
SFP	Safety Follow-up Period
SID	subject identification
SSc	systemic sclerosis
SUSAR	suspected unexpected serious adverse reactions
TB	tuberculosis
TEAE	treatment-emergent adverse event
TESAE	treatment-emergent serious adverse event
TM	transverse myelitis
US FDA	United States Food and Drug Administration
ULN	upper limit of normal
USA	United States of America
w/v	weight per volume

1 INTRODUCTION

1.1 Disease Background

Neuromyelitis optica (NMO; also known as Devic's syndrome) is a rare, chronic, autoimmune, inflammatory, demyelinating disorder of the central nervous system (CNS) characterized by attacks of predominantly optic neuritis (ON) and longitudinally extensive transverse myelitis (LETM). Commonly reported symptoms include unilateral and bilateral loss of visual acuity, ocular pain, loss of sensation, severe paraplegia, bladder and bowel dysfunction, paroxysmal tonic spasms of the trunk and limbs, and Lhermitte's phenomenon. Brain and brain stem involvement are rare but can occur, usually as an extension of a severe cervical myelitis, and may cause symptoms such as nausea, intractable vomiting, hiccups, and acute neurogenic respiratory failure (Wingerchuk et al, 1999; Misu et al, 2005). Up to 90% of patients with NMO have relapsing episodes of ON and myelitis rather than a monophasic course (Ghezzi et al 2004; Wingerchuk et al 1999). Relapses occur within 1 year of onset in 60% of patients and within 3 years in 90%. Relapses can be severe and result in blindness, paralysis, and even death due to neurogenic respiratory failure (Oh and Levy, 2012). Incomplete recovery from attacks is typical, and accumulative disabilities arise from the severity and frequency of attacks. By some estimates, within 5 years, > 50% of patients are blind in one or both eyes or require ambulatory help (Wingerchuk et al, 2007). Historically, mortality in NMO was as high as 30% at 5 years, but a more recent study suggests 9% at 6 years (Kitley, Leite et al, 2012).

Once thought to be a variant of multiple sclerosis (MS), NMO/neuromyelitis optica spectrum disorders (NMOSD) are now recognized as a distinct disease (Wingerchuk et al, 2007). A defining feature of NMO is the presence of serum autoantibodies against aquaporin-4 (AQP4) (ie, AQP4-immunoglobulin G [IgG] or NMO-IgG), which is detected in 60% to 90% of NMO/NMOSD patients (Jarius and Wildemann, 2010). Aquaporin-4 is the most abundant water channel expressed on the plasma membrane of astrocytes throughout the CNS. AQP4-IgG is thought to be pathogenic by causing astrocyte loss through activation of lytic complement components, antibody-dependent cellular phagocytosis (ADCP) and antibody-dependent cellular cytotoxicity (ADCC) and/or by unfavorably altering astrocyte physiology by reducing expression of key water channel proteins. AQP4-IgG is produced by CD19 positive (CD19+) B-lineage plasmablasts (Chihara et al, 2011), and that the presence of these plasmablasts correlates with disease activity in NMO (Chihara et al, 2011; Kim et al, Sept 2011; Greenberg et al, 2012). It has been demonstrated that subpopulations of CD19+ and CD20 negative (CD20-) B cells showing morphological and phenotypical properties of plasmablasts are increased selectively in the peripheral blood of NMO patients and that anti-AQP4 antibodies are produced by these cells. These subsets of B cells have shown to expand in the 2 weeks prior to and during NMO attacks.

Neuromyelitis optica spectrum disorders include limited forms of NMO, which include severe or bilateral ON, transverse myelitis (TM), recurrent ON or TM, brainstem disorders (including intractable hiccup, nausea, or vomiting), hypothalamic disorders (including hypersomnia and syndrome of inappropriate antidiuretic hormone secretion; Popescu et al, 2011), and some encephalitic presentations but are unified by the detection of AQP4-IgG in serum or cerebrospinal fluid (Jacob et al, 2012).

Treatment for NMO/NMOSD includes management of acute attacks, prevention of attacks, monitoring adverse events (AEs), and evaluating changes in therapy for breakthrough disease or lack of tolerability. Currently, there are no approved treatments in any country indicated for the treatment of NMO/NMOSD, and to date, there have been no controlled clinical studies for this disease. Patients with NMO/NMOSD are typically treated prophylactically with off-label immunosuppressants such as azathioprine (AZA), mycophenolate mofetil (MMF), daily prednisone, or rituximab to prevent relapses. High-dose steroids and plasma exchange are generally used for the acute management of relapses. Results of uncontrolled studies with rituximab, a chimeric monoclonal antibody (mAb) against the human CD20 molecule (Browning, 2006), provide evidence for the therapeutic effect of B-cell depletion in NMO/NMOSD (Cree et al, 2005; Jacob et al, 2008; Bedi et al, 2011; Kim et al, Nov 2011; Ip et al, 2013). B-cell analysis of rituximab failures revealed the presence of CD19+/CD20- plasmablasts (Dr Larry Steinman, Professor, Stanford University, personal communication), which support the need for a more effective B-cell depleting mAb than rituximab in the treatment of this disease. Therefore, it is conceivable that direct suppression of CD19+ B cells could be more effective in mitigating the impact of plasmablasts on NMO/NMOSD attacks than suppression of CD20+ B cells. Anti-CD20 mAbs may work by starving CD19+ plasmablast generation (ie, by targeting CD19+ and CD positive (CD20+) memory B cells rather than CD19+ plasmablasts directly). This suggests that targeting CD19+ B cells has a potential advantage over targeting CD20+ B cells as a therapeutic intervention for treating NMO and NMOSD.

1.2 MEDI-551 Background

MEDI-551 is briefly described below. Refer to the current Investigator's Brochure (IB) for details.

MEDI-551 is a humanized, affinity-optimized, afucosylated immunoglobulin G1 kappa (IgG1κ) mAb that binds to the B-cell specific surface antigen CD19 resulting in the depletion of B cells. MEDI-551 is glycoengineered by expression of mAb 16C4 in a fucosyltransferase-deficient Chinese hamster ovary producer cell line (BioWa Potelligent® Technology), which generates a homogenously afucosylated antibody. The removal of fucose from the mAb Fc results in approximately 10-fold increased affinity for the activating Fcγ receptor IIIA and significantly enhances ADCC. The antigen-binding properties of MEDI-551 (high affinity, slow internalization, and slow off-rate) are favorable for an ADCC-dependent mechanism of

action. MEDI-551 is composed of 2 light chains and 2 heavy chains, with an overall molecular weight of approximately 149 kilodalton (kDa). MEDI-551 does not mediate complement-dependent cytotoxicity (CDC).

1.3 Summary of Nonclinical Experience

Nonclinical evaluation demonstrated that MEDI-551 specifically recognizes human CD19 and has poor or no cross-reactivity to CD19 expressed on cells from nonhuman primates, rodents, or rabbits. Therefore, the human CD19 transgenic (huCD19 TG) mouse was selected as the relevant animal model for testing the pharmacodynamics (PD) and toxicology of MEDI-551.

In huCD19 TG mice, MEDI-551 effectively depleted B cells in blood and tissue. The duration of B-cell depletion was dose-dependent and was sustained for more than 10 weeks after treatment with a single 250 µg injection of MEDI-551. An additional 4-6 weeks were required for the B cells to recover to levels and maturities similar to those in immunoglobulin (Ig) G control-treated animals. The effect of treatment with MEDI-551 was limited to B cells and did not have an impact on other immune cells in circulation, as assessed by flow cytometry. Thus, MEDI-551 selectively targets and depletes B cells. Furthermore, during the course of the study, the levels of Ig in serum increased in control animals. These increases in serum Ig were blocked in groups treated with MEDI-551, suggesting that MEDI-551 inhibits antibody production. In a murine model of experimental systemic sclerosis (SSc), where production of pathogenic autoantibodies requires CD19+ B cells, MEDI-551 was highly effective at reducing circulating and target tissue infiltrating B cells. Total serum Ig, autoantibodies, and deposition of complement proteins in target tissues were also reduced by MEDI-551. These findings demonstrate that treatment with MEDI-551 may result in clinical benefit in autoimmune diseases in which B-cell-dependent T-cell activation and autoantibodies are thought to have pathogenic roles.

Completed nonclinical toxicology studies with the huCD19 TG mouse model demonstrated that there were no adverse effects after a single intravenous (IV) dose (up to 50 mg/kg), 5 weekly IV doses (up to 36.6 mg/kg), or 13 or 26 weekly IV doses (up to 30 mg/kg). The only findings from these toxicology studies were related to the pharmacologic action of B-cell depletion. The no observed adverse effect level (NOAEL) for MEDI-551 as a single IV agent is at least 36.6 mg/kg/week for up to 1 month and 30 mg/kg/week for multiple IV doses up to 6 months. Results of a fertility and embryo-fetal development (EFD) study showed no adverse findings in the study, other than a treatment-related reduction in fertility index and the number of mice that were pregnant/number of mice in cohabitation. Importantly, there was no MEDI-551 impact on EFD. Treatment with MEDI-551 resulted in the expected pharmacological depletion of total B lymphocytes in peripheral blood of adult mice.

An additional 13-week repeat-dose study to evaluate the toxicity of weekly subcutaneous (SC) administration of MEDI-551 (3 or 30 mg/kg SC; 30 mg/kg IV comparator) has been

completed. While the findings during the dosing phase were similar to those identified in the previous repeat dose toxicology studies, during the recovery phase an increased incidence of background bronchiolo-alveolar adenomas (benign) was noted in the 30 mg/kg IV group only. Given that this finding is isolated to this study and was not observed in the longer duration, 26-week chronic toxicity study (which also included a 30 mg/kg IV group), the relevance to overall risk assessment for patients is unknown.

For detailed descriptions of nonclinical studies and results refer to the MEDI-551 IB.

1.4 Summary of Clinical Experience

The current clinical program for MEDI-551 consists of two completed Phase 1 studies, one in adults with SSc (Study MI-CP200) and one in adults with relapsing forms of MS (Study CD-IA-MEDI-551-1102), and 6 ongoing Phase 1 and Phase 2 studies in adults with B-cell malignancies (Studies MI-CP204, CD-ON-MEDI-551-1088, CD-ON-MEDI-551-1019, D2852C00004, J1340, and D2850C00001).

Across these studies, MEDI-551 has been and is being administered as an IV infusion at single doses up to 10 mg/kg, at multiple doses up to 12 mg/kg. For Study MI-CP204, the maximum tolerated dose (MTD) of MEDI-551 was not observed. The highest planned doses were 10 mg/kg (median exposure of 675 mg [480-1,034 mg]) in Study MI-CP200 and 12 mg/kg (maximum median average monthly exposure and duration of 1218.2 mg [618.7-2152 mg] and 6.0 months [1.0-24.1 months]) in Study MI-CP204.
Study CD-IA-MEDI-551-1102 in relapsing forms of MS evaluated the safety and tolerability of a single course of IV MEDI-551 (30, 100, or 600 mg) or placebo administered twice as a fixed IV dose on Day 1 and Day 15, or a single dose of SC MEDI-551 (60 or 300 mg) or placebo over a 24-week period with long-term follow up for all subjects for B-cell recovery.

The most frequently reported treatment-emergent adverse events (TEAEs) in studies of MEDI-551 in subjects with SSc or relapsing forms of MS include infusion-related reaction, nausea, fatigue, pain in extremity, arthralgia, and cough. The most frequently reported TEAEs in studies of MEDI-551 in subjects with advanced B-cell malignancies include infusion-related reaction, anemia, fatigue, chills, cough, diarrhea, nausea, vomiting, neutropenia, thrombocytopenia, pyrexia, hypocalcemia, hypomagnesemia, hypertriglyceridemia, and hypokalemia.

Infusion-related reaction is the only identified risk of IV MEDI-551 to date, and has occurred in both oncology and nononcology studies with MEDI-551. Although the exact mechanism of the MEDI-551-associated infusion-related reaction is unknown, such events can occur with the administration of foreign proteins such as other types of mAbs. The risk of infusion-related reaction is mitigated by premedicating subjects prior to administration of IV MEDI-551. While premedication may reduce infusion-related reactions, it may not completely

eliminate the risk. To date, no serious infusion-related reactions have been reported in subjects who received protocol-defined premedication prior to administration of IV MEDI-551.

Progressive multifocal leukoencephalopathy (PML) has been reported in the setting of B-cell depleting therapies and other immunosuppressive regimens (Calabrese et al, 2015) and is a potential risk of treatment with MEDI-551. A subject in Study CD-IA-MEDI-551-1155 developed altered mental state, seizures, and new brain lesions that on brain MRI raised suspicion of PML. Cerebrospinal fluid (CSF) testing for the John Cunningham virus (JCV) was performed, with inconclusive results (two negative results in reference laboratories and one positive result in a local laboratory). Brain tissue was not available for examination. The subject died from cardiopulmonary complications. Based on all available data, the differential diagnosis included acute disseminated encephalomyelitis (ADEM), atypical NMOSD attack, and PML. No conclusive diagnosis could be made.

Details of all clinical studies with MEDI-551 are provided in the IB.

1.5 Rationale for Study

MEDI-551 is a humanized, affinity-optimized, afucosylated IgG1κ mAb that binds to the B-cell specific surface antigen CD19 resulting in the depletion of B cells. It is currently in clinical development in diseases such as B-cell malignancies and relapsing forms of MS, where B-cell depletion may have or has been shown to have a therapeutic effect.

Neuromyelitis optica/NMOSD is a rare, chronic, relapsing disorder with debilitating effects. There are currently no medicinal products approved for the prevention of NMO/NMOSD relapses or the treatment of acute relapses. Off-label medications are currently being used for the prevention and treatment of NMO/NMOSD relapse based on low-level evidence and unproven efficacy. There is a high unmet medical need for more effective therapies in this patient population. Furthermore, to date, no randomized-controlled clinical trials have been conducted in this patient population.

Available research shows NMO/NMOSD to be an autoantibody-mediated, plasmablast-driven disease, and that depleting B cells may have an impact on delaying or preventing relapse and disease progression. Since the results of small, uncontrolled studies of the anti-CD20 mAb rituximab have provided evidence for the therapeutic effect of B-cell depletion in NMO/NMOSD (Cree et al, 2005; Jacob et al, 2008; Bedi et al, 2011; Kim et al, Nov 2011; Ip et al, 2013) and that a CD19+ B-cell depleting agent may be more effective than a CD20+ B-cell depleting agent in the treatment of this disease, this study is being conducted to evaluate the efficacy of MEDI-551 in this target population.

Results of preclinical and clinical studies with MEDI-551 demonstrate that MEDI-551 provides an acceptable risk-benefit ratio for patients with NMO/NMOSD and support the initiation of a registration study.

Further information regarding the rationales for the study design and dose-level selection are provided in Section 3.2.

1.6 Research Hypotheses

1.6.1 Primary Hypothesis

The primary hypothesis for this study is that by depleting CD19+ B cells including plasmablasts and plasma cells, MEDI-551 is effective in reducing the risk of NMO/NMOSD attack compared to placebo, thus effectively reducing the risk of irreversible disability caused by such attacks.

1.6.2 Secondary Hypotheses

The secondary hypotheses are:

- Depletion of CD19+ B cells by MEDI-551 reduces worsening in Kurtzke Expanded Disability Severity Scale (EDSS) and decreases the loss of low-contrast visual acuity in adult subjects with NMO/NMOSD.
- Depletion of CD19+ B cells by MEDI-551 reduces NMO/NMOSD-related in-patient hospitalizations.
- Depletion of CD19+ B cells by MEDI-551 reduces the cumulative number of active lesions found on MRI.

2 OBJECTIVES

2.1 Objectives

2.1.1 Primary Objective

To compare the efficacy of MEDI-551 versus placebo in reducing the risk of an NMO/NMOSD attack in subjects with NMO/NMOSD.

2.1.2 Secondary Objectives

1. To compare the efficacy of MEDI-551 versus placebo on the reduction of EDSS worsening in subjects with NMO/NMOSD.
2. To compare the efficacy of MEDI-551 versus placebo on the change from baseline of low-contrast visual acuity score in subjects with NMO/NMOSD.
3. To compare the efficacy of MEDI-551 versus placebo in reducing the cumulative active MRI lesion count (new gadolinium [Gd]-enhancing or new/enlarging T2).
4. To compare the efficacy of MEDI-551 versus placebo in reducing NMO/NMOSD-related in-patient hospitalizations in subjects with NMO/NMOSD.
5. To characterize the long-term efficacy of MEDI-551 by means of annualized attack rate.

6. To evaluate the safety and tolerability of a single course of MEDI-551 in subjects with NMO/NMOSD in the Randomized-controlled Period (RCP) and repeated doses of MEDI-551 in the Open-label- Period (OLP).
7. To characterize the pharmacokinetic (PK) profile and immunogenicity of MEDI-551 in NMO/NMOSD subjects.

2.1.3 Exploratory Objectives

1. To compare the effect of MEDI-551 versus placebo on health-related quality of life (HRQoL) as measured by the 4-week recall SF-36 Health Survey (SF-36) physical component score (PCS) and mental component score (MCS) in NMO/NMOSD subjects.
2. To compare the effect of MEDI-551 versus placebo on pain as measured using the pain numeric rating scale (NRS).
3. To characterize the PD profile (B cells and plasma cell signature) of MEDI-551 in NMO/NMOSD subjects.
4. To compare the effect of MEDI-551 versus placebo on AQP4-IgG titer.
5. To compare the effect of MEDI-551 versus placebo on soluble biomarkers (eg, cytokines, chemokines, and immunoglobulins) and genomic (ribonucleic acid [RNA; microRNA]) biomarkers and other relevant cells (eg, T cells, astrocytes) in NMO/NMOSD subjects.

2.2 Study Endpoints

2.2.1 Primary Endpoint

The primary endpoint is the time (days) from Day 1 to onset of an Adjudication Committee (AC)-determined NMO/NMOSD attack on or before Day 197. The definition of an NMO/NMOSD attack is the presence of a new symptom(s) or worsening of an existing symptom(s) related to NMO/NMOSD that meets at least ONE of the protocol-defined criteria for an NMO/NMOSD attack provided in Table 11.

2.2.2 Secondary Endpoints

Endpoints 1, 2, 3, and 4 are key secondary endpoints to be considered for studywise Type I error control.

1. Worsening from baseline in EDSS at last visit during the RCP.
2. Change from baseline in low-contrast visual acuity binocular score measured by low-contrast Landolt C Broken Rings Chart, at last visit during the RCP.
3. Cumulative total active MRI lesions (new Gd-enhancing or new/enlarging T2) during the RCP.

4 Number of NMO/NMOSD-related in-patient hospitalizations. In-patient hospitalization is defined as more than an overnight stay (see Section 4.3.1.6).

5 Annualized attack rate (total number of AC-determined NMO/NMOSD attacks normalized by person-years) during any exposure to MEDI-551.

6 Treatment-emergent adverse events, including treatment-emergent serious adverse events (TESAEs). Laboratory measurements as well as their changes or shift from baseline over time.

7 Pharmacokinetic profile of MEDI-551.

8 Incidence of anti-drug antibodies (ADAs) directed against MEDI-551 for the duration of the study, both predose and postdose for each subject.

2.2.3 Exploratory Endpoint(s)

1 Change from baseline in the 4-week recall SF-36 PCS and MCS at the last visit during the RCP.

2 Change from baseline in pain NRS in 5 locations at the last visit during the RCP.

3 B-cell counts (total and subsets).

4 Change from baseline in plasma cell gene signature.

5 Serum AQP4-IgG titers.

3 STUDY DESIGN

3.1 Description of the Study

3.1.1 Overview

This is a multicenter, multinational, randomized, double-masked, placebo-controlled- study with an open-label extension period to evaluate the efficacy and safety of IV MEDI-551 in adult subjects with AQP4-IgG seropositive and seronegative NMO and NMOSD. Following a screening period of up to 28 days, a maximum of 252 subjects with NMO/NMOSD will be randomized and dosed, in a 3:1 ratio [IV MEDI-551 (300 mg at Day 1 and 300 mg at Day 15) or placebo] for a period of 197 days (RCP).

Enrollment of subjects into the study will stop when a total of 67 AC-determined NMOSD attacks occur, when 252 subjects have been randomized and dosed, or following a recommendation by the independent DMC to stop enrollment, whichever occurs first.

Subjects who complete the RCP without experiencing an NMO/NMOSD attack will be given the option to enroll into an OLP and will initiate or continue treatment with MEDI-551. Subjects who experience an NMO/NMOSD attack during the RCP, and for whom the attack is determined by the AC, will be given the option to enroll into the OLP following rescue

therapy. Subjects for whom the NMO/NMOSD attack is not determined by the AC will continue in the RCP until Day 197 (or until another attack occurs that is determined by the AC). Subjects who are in the RCP when enrollment is discontinued upon recommendation of the independent DMC based on evidence of efficacy and safety, will discontinue the RCP as soon as possible, preferably within 14 days, and be given the option to enter the OLP.

The OLP will continue for a minimum of 1 year and a maximum of 3 years after the last subject enters, or until regulatory approval for MEDI-551 as treatment for NMO in the participating country, or until the Sponsor discontinues development of MEDI-551 in this indication, whichever occurs first as described in Figure 1. Subjects can choose to exit the OLP at any time for any reason, including seeking alternative treatment options, at which point they will enter the Safety Follow-up Period (SFP; unless consent is withdrawn).

All subjects will continue in the SFP for a total of 12 months from last dose to evaluate the long-term safety of the investigational product.

Figure 1 Study Flow Diagram

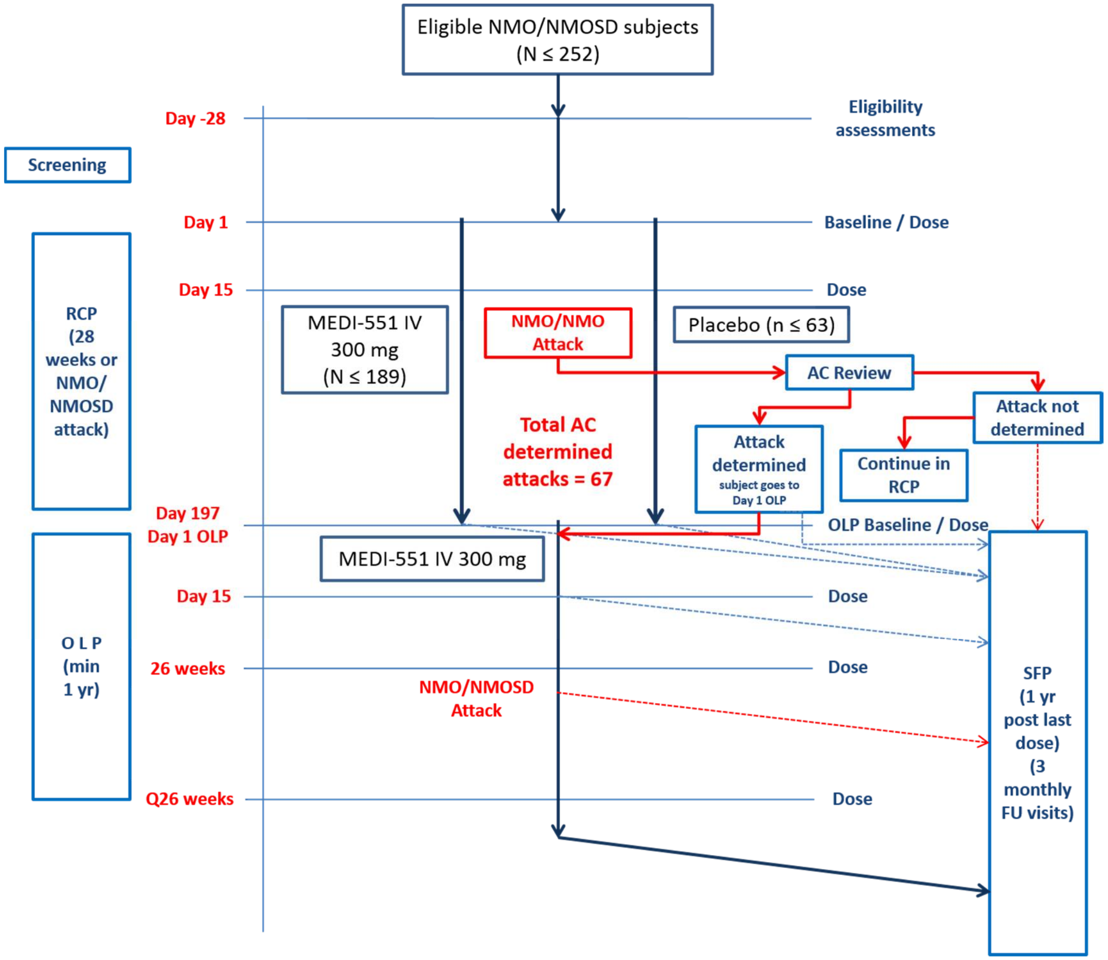

AC = Adjudication Committee; AQP4-IgG = aquaporin-4 immunoglobulin G; FU = follow-up; IV = intravenous; max = maximum; min = minimum; NMO/NMOSD = neuromyelitis optica/neuromyelitis optica spectrum disorders; OLP = Open-label Period; RCP = Randomized-controlled Period; Q26W = every 26 weeks; SFP = Safety Follow-up Period; yr = year.

The endpoints to be measured in this study are described in Section 2.2.

3.1.1.1 Screening Period

Subjects with the diagnosis of NMO/NMOSD will be screened over a 28-day period to establish their eligibility to participate in the study based on the inclusion and exclusion criteria in Section 4.1.2 and Section 4.1.3, respectively. AQP4-IgG serostatus will be determined by a central laboratory. For subjects who are found to be AQP4-IgG negative in the screening period, relevant data documenting their NMO disease will be assessed by an independent Eligibility Committee (see Section 4.2.1.1) to ensure that the data are consistent with the diagnosis of NMO. All subjects who fulfill eligibility criteria will then be randomized into the study. Subjects undergoing screening at the time when the 252nd subject is randomized and dosed, subjects in screening at the time the 67th AC-determined attack is confirmed, and subjects undergoing screening at the time enrollment is terminated for any other reason, will not be randomized.

3.1.1.2 Randomization (Day 1)

Two hundred and fifty-two subjects will be randomized into the study in a 3:1 ratio to receive IV MEDI-551 (300 mg) or placebo as described in Table 1. Randomization will occur on Day 1 (within 28 days of the start of screening) and will be stratified by AQP4-IgG serostatus (in a ratio of approximately 80:20 seropositive and seronegative subjects, respectively) and by region (Japan vs non-Japan).

3.1.1.3 Randomized-controlled Period (Day 1 to Day 197)

Following randomization on Day 1, subjects will be treated with MEDI-551 or placebo on Day 1 and Day 15. An oral corticosteroid course will be initiated on Day 1 (prednisone 20 mg/day or equivalent oral glucocorticoid) and continue until Day 14. Tapering of the oral corticosteroids will occur from Day 15 to Day 21. By Day 21, tapering must be completed.

During the RCP, subjects will be followed at scheduled study visits and by telephone interview as described in Table 6. The planned duration of the RCP for each subject will be 197 days. All subjects who complete the RCP without experiencing an NMO/NMOSD attack will be given the option to enter the OLP. Subjects who are in the RCP when a total of 67 AC-determined NMOSD attacks have occurred, or when enrollment is discontinued upon recommendation of the independent DMC based on evidence of efficacy and safety, will discontinue the RCP as soon as possible, preferably within 14 days, and be given the option to enroll into the OLP. .

The process for the diagnosis of an NMO/NMOSD attack is outlined in Figure 3. Subjects will be monitored for new or worsening symptoms related to NMO/NMOSD during study visits and with follow-up phone calls every 2 weeks between study visits (or if a scheduled visit is missed).

When a possible new or worsening symptom(s) related to NMO/NMOSD is identified, subjects will be required to inform the site. If an Assessment Visit is deemed necessary, this must be scheduled as soon as possible but within 72 hours of reporting of the symptom to the site. At the Assessment Visit, subjects will initially undergo evaluations to determine if the symptoms are related to NMO/NMOSD; if related, the subjects will undergo further evaluations to determine if the symptoms meet at least ONE of the protocol-defined criteria for an NMO/NMOSD attack outlined in Table 11. In cases where a new or worsening symptom(s) does not meet at least one of the protocol-defined criteria for an NMO/NMOSD attack, the subject will continue in the RCP. The data related to the assessment of the symptoms that were determined by the investigator as not related to NMO/NMOSD will be sent to the AC for review.

Assessment of new symptoms or worsening of existing symptoms should be completed within 5 days to determine if an attack has occurred. Treatment of an attack should preferably be initiated after completion of the attack assessment and the determination that the protocol attack criteria have been met. However, the Principal Investigator can initiate rescue therapy at any time before full assessment is completed. Rescue therapy will be given as directed by the investigator. This may include IV corticosteroids, intravenous immunoglobulin (IVIG), and/or plasma exchange (PLEX).

Upon completion of the Assessment Visit, the complete set of data generated from the assessments will be sent to the AC regardless of whether an NMO/NMOSD attack was diagnosed according to the protocol criteria by the Principal Investigator. The adjudication process will be completed within 14 days (+ 3 days) from initiation of the Assessment Visit and the AC determination will be communicated to the Principal Investigator.

The procedures for identifying and adjudicating an NMO/NMOSD attack are described in Section 4.3.1.1.

Subjects for whom the diagnosis of an NMO/NMOSD attack is not determined by the AC will be given the option to continue in the RCP until Day 197. Subjects for whom the diagnosis of an NMO/NMOSD attack is determined by the AC will be given the option to enter the OLP.

In addition, subjects who experience an NMO/NMOSD attack that requires rescue treatment and meets the protocol-defined criteria, regardless of the outcome of the AC review, will undergo an Attack Follow-up Visit 28 days from Day 1 of the Assessment Visit. This visit may correspond with an OLP or SFP visit or may be scheduled separately.

If subjects do not wish to enter the OLP or wish to leave the RCP at any point, they will continue to the SFP (unless consent is withdrawn).

3.1.1.4 Open-label Period

Subjects will be given the option to enter the OLP if they:

1. Complete 197 days of the RCP, or
2. Experience an AC-determined NMO/NMOSD attack during the RCP, or
3. Are in the RCP at the time when 67 AC-determined attacks have occurred
4. Are in the RCP when enrollment is discontinued upon recommendation of the DMC based on evidence of efficacy and safety (see Section 4.4).

Subjects who discontinue from the RCP for reasons other than one of the reasons above will not be eligible for the OLP. Reasons for subjects not entering the OLP will be captured. These subjects will be followed for safety in the SFP.

The first day of the OLP will be Day 1 (OLP Day 1). Upon entering the OLP for one of the three reasons outlined above, subjects will receive MEDI-551 as described in Table 2. During the OLP, subjects will be followed at scheduled study visits and will initiate or continue on MEDI-551 therapy per Table 7. The OLP will continue for a maximum of 3 years (after the last subject enters), until regulatory approval for MEDI-551 in each participating country or until the Sponsor discontinues development of MEDI-551 in this indication, whichever occurs first. Subjects can choose to exit the OLP at any time for any reason, including seeking alternative treatment options, at which point they will enter the SFP (unless consent is withdrawn).

Subjects will be followed for NMO/NMOSD attacks in the same fashion as in the RCP and events will be centrally adjudicated.

3.1.1.5 Safety Follow-up Period

The SFP will start when a subject prematurely discontinues from the RCP or OLP. The length of the SFP will be determined by the time elapsed from the time of the last dose of the investigational product to the time of the premature discontinuation, to complete a total of 52 weeks. During the SFP, the subject will be monitored for AEs/serious adverse events (SAEs), B-cell levels, ADAs, and immunoglobulin levels.

During the SFP, a subject may receive standard treatment for their NMO/NMOSD at the discretion of the investigator.

3.1.2 Treatment Regimen

Randomized-controlled Period

Subjects will be randomized in a 3:1 ratio to receive MEDI-551 or placebo during the 197 days of the RCP as described in Table 1.

Table 1 Randomized-controlled Period Treatment Regimen

Treatment Arm	Treatment Regimen
1	300 mg IV MEDI-551 on Day 1 and Day 15
2	IV Placebo on Day 1 and Day 15

IV = intravenous

Investigational product will be administered as a 90-minute IV infusion via an infusion pump. All subjects in the placebo and MEDI-551 arms will be premedicated on Day 1 and Day 15 with IV methylprednisolone (80-125 mg or equivalent glucocorticoid), oral (PO) diphenhydramine (25-50 mg or equivalent antihistamine), and PO paracetamol (acetaminophen; 500-650 mg) prophylactically to prevent infusion reactions. Sites should utilize local supplies of the premedications where possible. The Sponsor will provide reimbursement during the study.

Additionally, all subjects entering the RCP will be treated for 2 weeks (Day 1 to Day 14) with oral corticosteroids (prednisone 20 mg/day or equivalent oral glucocorticoid). A tapering schedule will be implemented from Day 15 to Day 21 (see Section 4.5.2).

Open-label Period

All subjects who enter the OLP will receive open-label MEDI-551. In order to provide subjects who were receiving placebo during the RCP with 2 doses of MEDI-551 (OLP Day 1 and OLP Day 15) and to ensure maintaining the masking of the study, subjects who were in Treatment Arm 1 (MEDI-551) or Treatment Arm 2 (placebo) will be dosed as described in Table 2.

Table 2 Open-label Period Treatment Regimen

Treatment Arm in RCP	Treatment Regimen
1	300 mg IV MEDI-551 on OLP Day 1, masked IV placebo OLP Day 15, then 300 mg IV MEDI-551 Q26W thereafter [a]
2	300 mg IV MEDI-551 on OLP Day 1, masked 300 mg IV MEDI-551 on OLP Day 15, then 300 mg IV MEDI-551 Q26W thereafter [a]

IV = intravenous; OLP = Open-label Period; Q26W = every 26 weeks; RCP = Randomized-controlled Period; SFP = Safety Follow-up Period.

[a] The OLP will continue for a minimum of 1 year after the last subject enters and a maximum of 3 years (after the last subject enters), or until regulatory approval for MEDI-551 in the participating country, or until the Sponsor discontinues development of MEDI-551 in this indication, whichever occurs first. Subjects can choose to exit the OLP at any time for any reason, including seeking alternative treatment options, at which point they will enter the SFP (unless consent is withdrawn).

Dosing of subjects enrolling into the OLP following an adjudicated NMO/NMOSD attack, following the occurrence of the 67th adjudicated NMO/NMOSD attack, or following

discontinuation of enrollment upon recommendation of the independent DMC based on evidence of efficacy and safety, will follow the OLP dosing regimen as detailed above in Table 2.

Investigational product will be administered as a 90-minute IV infusion via an infusion pump. All subjects will be premedicated on OLP Day 1 and OLP Day 15 and at subsequent dosing visits with IV methylprednisolone (80-125 mg or equivalent glucocorticoid), PO diphenhydramine (25-50 mg or equivalent antihistamine), and PO paracetamol (acetaminophen; 500-650 mg) prophylactically to prevent infusion reactions. Sites should utilize local supplies of the premedications where possible. The Sponsor will provide reimbursement during the study.

3.1.3 Management of Study Medication Related Toxicities

Infusion-related reaction is the only identified risk of IV MEDI-551 to date and has occurred in both MEDI-551 oncology and non-oncology studies. For subjects experiencing an infusion-related reaction, an approach to these reactions based on severity of symptoms is presented in Table 3 and Appendix 2 (National Institute of Allergy and Infectious Diseases [NIAID] and Food Allergy and Anaphylaxis Network [FAAN] Guidance for Anaphylaxis Diagnosis), with suggested treatment options. Final treatment is at the discretion of the investigator and should reflect local standard of care.

Table 3 An Approach to Management of Anaphylactic, Hypersensitivity, and Infusion Reactions

Severity of Symptoms	Treatment	Investigational Product
**Mild reactions (infusion and hypersensitivity)**<br>Mild infusion reactions such as headache, nausea, nonpruritic rash, or mild hypersensitivity reactions including localized cutaneous reactions such as mild pruritus, flushing, rash, dizziness, headache, ≤ 20 mmHg change in systolic BP from pre-infusion measurement	• Evaluate subject, including close monitoring of vital signs<br>• At the discretion of the investigator, treat subject, for example, with:<br>• Normal saline (~500-1000 mL/hour IV) and/or<br>• Diphenhydramine 50 mg IV or equivalent and/or<br>• Acetaminophen 500-650 mg or equivalent dose of paracetamol and/or<br>• Topical antihistamines and/or low-potency topical corticosteroid preparations and/or<br>• Anti-nausea medication, as needed	• Stop IP infusion immediately<br>• Once the event has resolved, resume current IP infusion under observation and complete IP infusion at no more than half the planned infusion rate
**Moderate reactions (infusion)**<br>Infusion reaction such as those listed above under mild reactions but excluding moderate hypersensitivity reactions (see below)	• Evaluate subject, including close monitoring of vital signs<br>• Treat subject, for example, with:<br>• Normal saline (~500-1000 mL/hour IV) and/or<br>• Diphenhydramine 50 mg IV or equivalent and/or<br>• Acetaminophen 500-650 mg or equivalent dose of paracetamol and/or<br>• Anti-nausea and/or antiemetic intramuscular, as needed	• Stop the infusion immediately<br>• Once the event has resolved, resume current IP infusion under observation and at no more than half the planned infusion rate after treatment of current signs and symptoms as suggested
**Moderate hypersensitivity reactions**<br>Infusion reactions which may include generalized rash or urticaria, palpitations, chest discomfort, shortness of breath, hypo- or hypertension with > 20 mmHg change in systolic BP from pre-infusion measurement	• Evaluate subject, including close monitoring of vital signs<br>• Treat subject, for example, with:<br>• Normal saline (~500-1000 mL/hour IV) and/or<br>• Diphenhydramine 50 mg IV or equivalent and/or<br>• Acetaminophen 500-650 mg or equivalent dose of paracetamol and/or<br>• IV corticosteroids, such as hydrocortisone 100 mg or methylprednisolone 20-40 mg	• Stop the infusion immediately<br>• Once the event has resolved, resume current IP infusion under observation and at no more than half the planned infusion rate after treatment of current signs and symptoms as suggested

Table 3 An Approach to Management of Anaphylactic, Hypersensitivity, and Infusion Reactions

Severity of Symptoms	Treatment	Investigational Product
**Severe**<br>Above plus fever with rigors, hypo- or hypertension with ≥ 40 mmHg change in systolic BP, signs of end organ dysfunction (eg, symptomatic hypotension such as hypotonia, syncope, incontinence, seizure) from pre-infusion measurement, or wheezing, angioedema, or stridor	• Evaluate subject, including close monitoring of vital signs<br>• Treat subject, for example, with:<br>• Normal saline (~500-1000 mL/hour IV) and/or<br>• Diphenhydramine 50 mg IV or equivalent and/or<br>• Acetaminophen 500-650 mg or equivalent dose of paracetamol and/or<br>• IV corticosteroids, such as hydrocortisone 100 mg or methylprednisolone 20-40 mg	• Stop the infusion immediately<br>• Once all symptoms have disappeared, resume current IP infusion under observation and at no more than half the planned infusion rate after treatment of current signs and symptoms as suggested<br>• If severe event recurs in the same subject, discontinue further IP administration
**Life threatening**<br>Defined as a reaction that is life threatening and requires pressor and/or ventilator support or shock associated with acidemia and impairing vital organ function due to tissue hypoperfusion	• Evaluate subject, including close monitoring of vital signs<br>• Maintain airway, oxygen if available<br>• Treat subject immediately, for example with:<br>• Normal saline (~500-1000 mL/hour IV)<br>• Epinephrine for bronchospasm, hypotension unresponsive to IV fluids, or angioedema. Dose and route as per local standard of care, example, epinephrine 1:1000, 0.5-1.0 mL administered SC for mild cases and intramuscular for more severe cases<br>• IV corticosteroids, such as hydrocortisone 100 mg or methylprednisolone 20-40 mg<br>• Diphenhydramine 50 mg IV or equivalent<br>• Acetaminophen 500-650 mg or equivalent dose of paracetamol<br>• Call emergency medical transport for transport to emergency hospital based on judgment of the investigator	• Stop IP infusion immediately<br>• Do not resume current infusion<br>• Permanently discontinue IP administration<br>• Consider need for additional oral antihistamine administration or oral corticosteroid administration to prevent reoccurrence of symptoms over subsequent 2-3 days

BP = blood pressure; IP = investigational product; IV = intravenous; mmHg = millimeters mercury; SC = subcutaneous.

3.2 Study Design and Dose Rationale

3.2.1 Study Design Rationale

This study design was developed to provide balance between scientific considerations and patient safety. A placebo-comparator treatment arm was chosen because there are no currently approved medications for the treatment of NMO/NMOSD. While there is anecdotal evidence for using various immunosuppressants in NMO/NMOSD, the efficacy and safety profiles of these agents are not well characterized because there have been no controlled clinical studies using these agents in this disease indication. The use of a placebo arm will allow for a clear and robust evaluation of MEDI-551 and provide for the highest sensitivity to detect efficacy. The therapeutic estimation of the potential effect of MEDI-551 is based on published literature, albeit uncontrolled data, showing that B-cell depletion may have a positive effect in preventing NMO/NMOSD attacks. The Sponsor expects that MEDI-551 will share the same class effects that have been seen with other B-cell depleting agents in subjects with NMO/NMOSD.

A 3:1 randomization scheme was chosen for this study. According to Dumville et al (2006), unequal randomization is particularly useful when the experimental group is expected to benefit greatly from a new treatment that would not have been otherwise available and can provide the clinician with a reduced learning curve by allowing treatment in a higher percentage of subjects (Dumville et al, 2006). The 3:1 randomization ratio is an effective and efficient approach to build an enriched safety database for MEDI-551 while keeping the number of required events or subjects in the placebo arm at a minimum acceptable level. This randomization ratio also addresses, to a certain degree, the serious ethical/safety concerns of investigators and patients regarding enrollment of subjects to a placebo arm. In addition to limiting the number of subjects who receive placebo, the study has been designed to limit the actual duration of placebo exposure to a maximum of 197 days or time to onset of an AC-determined NMO/NMOSD attack, whichever occurs earlier, after which all subjects will have the option to enter the OLP and receive MEDI-551.

Due to the potential severity of NMO/NMOSD attacks and their debilitating nature, the study is further designed to ensure safety in this subject population through careful monitoring and early evaluation of signs and symptoms of NMO/NMOSD attacks, scheduled study visits and study assessments, follow-up telephone contact every 2 weeks with study subjects by study site staff, a liberal "escape clause" (ie, immediate access to rescue therapy following identification of an NMO/NMOSD attack), and monitoring by an independent Data Monitoring Committee (DMC).

Also, the informed consent form (ICF), which will include detailed explanation and risk:benefit expectations, will provide subjects and investigators the opportunity to discuss

appropriate risk estimation and to fully understand the risks and potential benefits of this study.

The rationale for the use of oral corticosteroids (prednisone 20 mg/day or equivalent oral glucocorticoid) for the first 14 days, with a 1-week taper, is to provide prophylaxis against an NMO/NMOSD attack for the period wherein the PD effect of MEDI-551 is not expected. Based on our PK/PD data, a period of approximately 2-4 weeks is required for maximal B-cell depletion to occur.

In addition, studies with rituximab have reported a few attacks in the first month of therapy possibly related to increased B-cell activating factor (BAFF; Bar-Or et al, 2010) and therefore an increase of AQP4-IgG titers. Feedback from external experts has suggested that providing such prophylaxis is viewed as particularly important in a setting where existing immunosuppressive regimens will be withdrawn at randomization.

3.2.2 Dose Rationale

The 300 mg dose of MEDI-551 was selected to achieve complete peripheral B-cell depletion. Based on existing PK and B-cell PD data from Study MI-CP200, a fixed dose of 300 mg MEDI-551 given on Day 1 and Day 15 is predicted to fully deplete peripheral blood B cells to undetectable levels and maintain B-cell suppression for 28 weeks, therefore sustaining B-cell depletion for the duration of the RCP. In theory, an initial dose of MEDI-551 will deplete the peripheral blood B cells; however, additional B cells will then recirculate out of the lymphoid tissues. The second dose of MEDI-551 on Day 15 is timed to deplete the newly recirculated B cells from the peripheral blood.

This dose regimen is supported by clinical data from other B-cell depleting mAbs, where IV dosing on Day 1 and Day 15 will provide optimal B-cell depletion in both blood and tissues (Huffstutter et al, 2011; Hauser et al, 2008; Bar-Or et al, 2008; Kappos et al, 2011).

A fixed dose of 300 mg MEDI-551 administered on OLP Day 1 (and OLP Day 15 for subjects who received placebo in the RCP) and then every 26 weeks is predicted to fully deplete peripheral blood B cells to undetectable levels and maintain B-cell suppression for the dose interval of the OLP (Figure 2).

Figure 2 PD Model Simulation Supporting Dosing Regimen

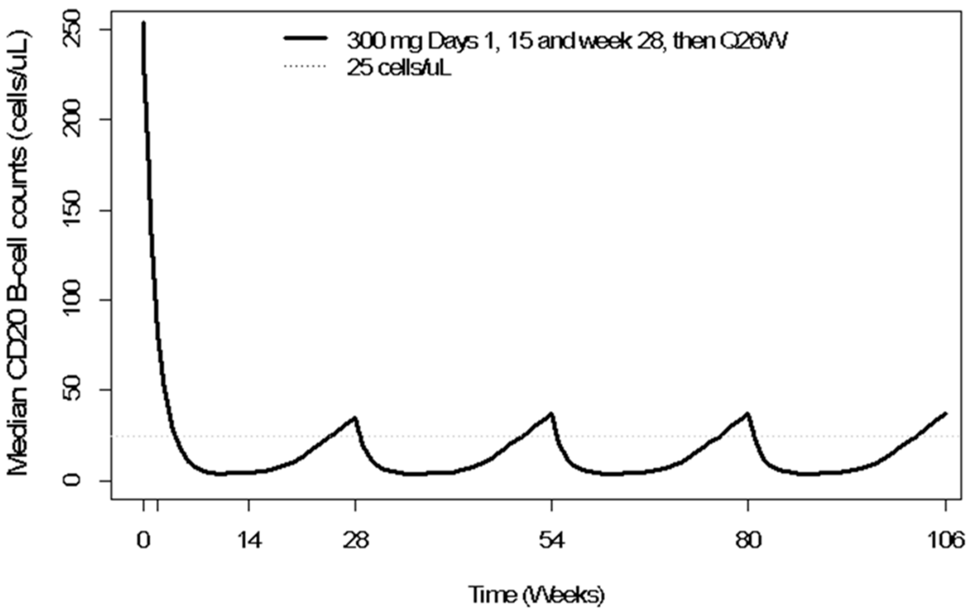

PD = pharmacodynamic; Q26W = every 26 weeks

3.2.3 Rationale for Study Population

This clinical study is designed to study the efficacy and safety of MEDI-551 as a maintenance treatment of subjects with established NMO/NMOSD aimed to reduce the risk of NMO/NMOSD attacks. Therefore, the inclusion/exclusion criteria define a group of adult subjects (women and men aged 18 years and above) who must have a diagnosis of NMO/NMOSD at the time of screening and a documented history of 1 or more NMO/NMOSD acute relapses that required rescue therapy within the last year, or 2 or more NMO/NMOSD acute relapses that required rescue therapy within the 2 years prior to screening.

Both AQP4-IgG seropositive and seronegative subjects will be enrolled in the study to fully capture the entire spectrum of this potentially fatal and rare demyelinating neurological disease. There is a series of clinical studies showing that AQP4-IgG is involved in the pathogenesis of NMO and that AQP4-IgG may be predictive of relapse and/or later conversion to definite NMO (Weinshenker et al, 2006; Matiello et al, 2008). In a multicenter study of 175 Caucasian patients, bilateral ON at onset was more common in AQP4-IgG seronegative patients as was simultaneous ON and myelitis, and the time to diagnosis was shorter in the AQP4-IgG seronegative group. Additionally, the disease course was more often monophasic in AQP4-IgG seronegative patients (Jarius et al, 2012).

There is strong evidence to support the hypothesis that NMO is a pathologically heterogeneous disease, as even using the most reliable of recombinant assays, there are subsets of subjects who do not test seropositive for AQP4-IgG that may still present with NMO and conditions that may cause myelitis and ON by other mechanisms. It has been speculated that different etiological mechanisms are involved in AQP4-IgG seronegative NMO/NMOSD. Recent studies have identified a subset of AQP4-IgG seronegative NMO patients who are positive for antibodies against myelin-oligodendrocyte glycoprotein (MOG), a protein expressed on the outer surface of the myelin sheath and oligodendrocytes (Kitley, Woodhall et al, 2012; Mader et al, 2011), and for antibodies against CV2/CRMP5 (Jarius et al, 2012). Since an anti-CD19 B-cell depleting mAb will remove the source of AQP4-IgG, it should also remove the source of these antibodies. The clinical presentation of an attack is considered by NMO experts to be indistinguishable between seropositive and seronegative NMO patients.

Several assays have shown to be able to detect AQP4-IgG even in samples taken from subjects during remission and treatment with strong immunosuppressants such as rituximab, AZA, mitoxantrone, and/or cyclophosphamide, plausibly ruling out the likelihood that seronegativity is generally the result of insufficient assay sensitivity.

In addition, evidence for the therapeutic effect of B-cell depletion with rituximab in NMO seropositive and seronegative subjects is supported by a number of uncontrolled studies comparing relapse rates and EDSS scores before and after treatment (Jacob et al, 2008; Bedi et al, 2011; Kim SH et al, 2011; Ip et al, 2013).

The AQP4-IgG status was available in all subjects in the previously described rituximab studies, with seronegative status occurring in nearly 20% to 30% of NMO/NMOSD patients tested.

Thus, this study population will include approximately 80% subjects who are AQP4-IgG seropositive and 20% of subjects who are AQP4-IgG seronegative. In subjects who are AQP4-IgG seronegative, the diagnosis of NMO is less clear; therefore, these subjects must meet the clinical criteria for NMO according to Wingerchuk et al, 2006 and not have brain MRI lesions consistent with MS. In addition, screening data of these subjects will be reviewed by an independent Eligibility Committee to confirm the diagnosis of NMO in the absence of AQP4-IgG seropositive status.

The study allows for inclusion of a wide age range of subjects as recent international research has shown under-representation of older participants in clinical studies. It is both unhelpful for optimal healthcare as well as fundamentally unjust to exclude participants from clinical research based solely on an arbitrary age limit (Briggs et al, 2012).

3.2.4 Rationale for Endpoints

3.2.4.1 Primary Endpoint

The primary endpoint of time (days) from Day 1 to onset of an AC-determined NMO/NMOSD attack on or before Day 197 is based on the natural history of the disease, which is characterized by a stepwise deterioration of motor, sensory, visual, and bowel/bladder function (Sellner et al, 2010), and recurrent attacks that can result in blindness, paralysis, and even death. Attacks of NMO/NMOSD are more severe than MS and usually result in incomplete recovery and permanent deficits. Thus, establishing a primary endpoint using an annualized relapse rate as usually utilized in MS studies where subjects typically have mild attacks and exacerbations followed by varying degrees of recovery, would expose subjects to a longer study period with an increased risk to the placebo subjects. Utilizing time to attack as the primary endpoint limits subject exposure but still provides a robust endpoint.

3.2.4.2 Secondary Endpoints

Worsening from Baseline in EDSS at Last Visit During the Randomized-controlled Period

A subject will be considered to have a worsening in overall EDSS score if one of the following criteria is met:

(a) Worsening of 2 or more points in EDSS score for patients with baseline score of 0.

(b) Worsening of 1 or more points in EDSS score for patients with baseline score of 1 to 5.

(c) Worsening of 0.5 points or more in EDSS score for patients with baseline score of 5.5 or more.

Myelitis attacks in NMO/NMOSD patients are severe and may lead to permanent disability. Measurement of disease progression in NMO/NMOSD patients is critically important since the potential for recovery depends on the severity of the attacks. The EDSS is a commonly used measure of disability in MS, and given the lack of any validated scales for NMO, is widely considered appropriate for measuring the level of disability in patients with NMO/NMOSD. Because the time to achieve these EDSS scores is often long, a short-term indicator of disease progression can be applied to the disease course by using an increase in EDSS score sustained over several months called “sustained disease progression.” Several definitions of sustained disease progression for clinical trials and observational studies have been proposed, including a 2- or more point increase in EDSS score for patients with a baseline score of 0; an increase in 1 point or more in EDSS score for patients with a baseline score of 1-5; or an increase of 0.5 points or more in EDSS score for patients with a baseline score of 5.5 or more has been used in the literature.

Change from Baseline in Low-contrast Visual Acuity Score, Measured by Low-contrast Landolt C Broken Rings Chart at Last Visit During the Randomized-controlled Period

Visual loss and dysfunction are recognized disabling clinical manifestations in patients with NMO/NMOSD. In MS clinical trials, low-contrast visual acuity emerged as the leading candidate to measure visual disability in MS. Subsequent studies found that low contrast acuity testing correlated well with brain MRI lesion burden, visual evoked potentials and retinal nerve fiber layer (RNFL) loss as measured by optical coherence tomography (OCT). Worsening of visual function by a clinically significant > 7 letters or approximately 1.5 lines for low-contrast acuity is associated with approximately 4.5 µm reduction in RNFL thickness in MS. Additional longitudinal studies have shown RNFL axonal loss over time that occurs even in the absence of an acute ON attack and correlates with clinical meaningful worsening of vision and quality of life in patients with MS. The low-contrast visual acuity test is used to determine the number of letters that can be read on a standardized low-contrast Landolt C Broken Rings Chart held at a distance of 3 meters. Due to lack of standard visual function measures specific to NMO/NMOSD, and the fact that both MS and NMO/NMOSD patients can experience ON, and this endpoint has been previously used widely in MS studies, there is good rationale for using this endpoint in this NMO/NMOSD study. This secondary endpoint will evaluate whether MEDI-551 will have an effect on improving vision sensitivity.

Reduction in the Cumulative Total Active MRI Lesions (New Gd-enhancing or New/Enlarging T2)

In clinical practice there is wide use of MRI in the diagnosis and management of NMO; however, the role of MRI in this setting is not well established and studied. The actual interpretation of MRI related to the diagnosis and the follow up of patients with NMO/NMOSD are not consistently established. Therefore, this study aims to collect longitudinal MRI data during both study periods including at the time of an attack, to investigate the characteristics and evolution of spinal and brain lesions in NMO/NMOSD.

Number of NMO/NMOSD-related In-patient Hospitalizations During the Randomized-controlled Period

Patients with relapsing NMO/NMOSD have recurrent attacks that can be severe and result in blindness, paralysis, and even death (Oh and Levy, 2012). Consequently, such attacks frequently result in in-patient hospitalizations. Less severe attacks can be managed without the need for hospitalization. Post initial acute phase of an attack, the patient will often require ongoing care and rehabilitation in the recovery phase post an attack. Therefore, this secondary endpoint will evaluate whether MEDI-551 will be effective in reducing NMO/NMOSD-related hospitalizations compared to placebo. All hospitalizations recorded during the RCP will be included in the analysis.

In-patient hospitalization, for the purposes of analysis of the secondary endpoint, is defined as a stay in hospital that goes beyond midnight of the first day of admission. Any duration of stay

(calculated as the difference between discharge date and admission date) of more than 1 day will contribute to this endpoint. This includes the time a subject may enter an emergency department plus subsequent admission to a ward. Hospitalizations for the administration of NMO-related medications or procedures only (ie, the administration or procedure was isolated and not provided during a subject's hospitalization due to NMO for another reason) will not be included in the secondary endpoint analysis.

Annualized Attack Rate (Total Number of Adjudication Committee-determined NMO/NMOSD Attacks Normalized by Total Person-years) During any Exposure to MEDI-551

This endpoint will be evaluated in all subjects who receive at least one dose of MEDI-551.

This secondary endpoint will be an important marker for assessing frequency of AC-determined NMO/NMOSD attacks during long-term treatment with MEDI-551. Although no comparator is available for long-term comparison, a descriptive summary of AC-determined NMO/NMOSD attack rate over various lengths of exposure to MEDI-551 will provide a sense of constancy of attack rate.

Safety and Tolerability of MEDI-551

The safety and tolerability endpoints are designed to evaluate the safety and tolerability of administration of MEDI-551 in subjects with NMO/NMOSD for this study population. These endpoints will be assessed primarily by summarizing TEAEs and TESAEs, and changes from baseline in laboratory measurements (including ADA status and titers and vaccine titers [see below]), significant physical examination findings, and vital sign measurements.

<u>Vaccine Titers</u>

Vaccination results in immunological memory by expansion of antigen specific CD4+ T-helper and CD8- cytotoxic cells as well as through induction of antigen-specific B–cell cell maturation and antibody secretion (Siegrist, 2013). Given the depletion effect of MEDI-551 on B cells, it is mechanistically relevant to investigate whether immunization responses are diminished by following vaccine-generated antibody titers, including tetanus.

3.2.4.3 Exploratory Endpoints

Change from Baseline in the Short Form-36 Physical and Mental Component Scores (4-week Recall Version)

The literature provides evidence of the strong impact NMO has on HRQoL (Chanson et al, 2011; Kanamori et al, 2011). In one study, the main impact was on PCS (Chanson et al, 2011); however, both PCS and MCS scores on the SF-36 Health Survey were significantly lower in NMO than for the general population, even in the cohort of patients outside an NMO attack period and with relatively low EDSS (mean score: 3.5), indicating the importance of the HRQoL evaluation in NMO.

Change from Baseline in Pain Numeric Rating Scale

A number of recent publications have indicated that patients with NMO/NMOSD experience pain throughout the course of their disease. Pain in demyelinating and inflammatory CNS diseases can be disabling and may contribute to a lower quality of life and overall increased health care burden. Neuromyelitis optica is associated with extensive spinal cord injury, brainstem disorders, ON, and sometimes a combination of these (Qian et al, 2012) and pain in five different locations (eyes, legs, arms, upper back and lower back, respectively) will be measured at baseline and monthly thereafter throughout the study.

B-cell Counts (Total and Subsets)

As MEDI-551 binds to and depletes CD19+ B cells, the depletion is a direct measure of PD effect. Additionally, CD19+ plasmablasts are increased in the peripheral blood on NMO/NMOSD patients and produce anti-AQP4 antibodies. Since AQP4-IgG titers correlate with disease activity (Jarius et al, 2008; Kim et al, Sept 2011), the extent and/or duration of B-cell/plasma-cell depletion may correlate with response to MEDI-551 in these subjects.

Plasma Cell Gene Signature

Production of pathogenic autoantibodies by plasma cells is a hallmark of autoimmune diseases; thus, plasma cell levels may be associated with the efficacy of B-cell depleting therapies like MEDI-551. Flow cytometry methods commonly used to enumerate and describe plasma cells are difficult to implement routinely in large clinical studies because of the insufficient stability of plasma cells. For this reason, whole genome microarray analysis of sorted cellular fractions was used to develop a highly sensitive and specific gene expression signature comprised of multiple plasma cell-enriched genes. In Study MI-CP200, MEDI-551 caused a statistically significant, dose-dependent depletion of the plasma cell gene signature. Thus, it would be relevant to characterize the effect of MEDI-551 on plasma gene signature in patients with NMO/NMOSD.

Subpopulations of CD19+ and CD20- B cells showing properties of plasmablasts are increased selectively in the peripheral blood of NMO/NMOSD patients and anti-AQP4 antibodies are produced by these cells. These B-cell subsets have been shown to expand 2 weeks prior to and during NMO/NMOSD attacks. Measuring the depletion of plasma cells/plasmablasts will provide key PD information as MEDI-551 should deplete this cell population. The levels of the plasma cell signature prior to and following MEDI-551 treatment may provide information about which patients are more likely to respond to this therapy and/or predict an attack.

Serum AQP4-IgG Titers

Several previous studies determined that AQP4 antibody levels correlated with clinical disease activity, with relapses associated with up to a 3-fold increase in AQP4 antibody titers (Jarius

et al, 2008). Since CD19+ plasmablasts produce pathogenic anti-AQP4 antibodies, it will be important to characterize the time course and extent of the effect of MEDI-551 on AQP4-IgG titers.

3.2.4.4 Exploratory Investigations

Levels of Soluble and Genomic Biomarkers (eg, Cytokines, Chemokines, Immunoglobulins, and mRNA/Micro-RNA), and Relevant Cell Counts

A number of biomarkers that may provide additional insights into the mechanism of action of MEDI-551 in NMO/NMOSD or potentially predict response to MEDI-551 will be explored. An example of a relevant biomarker is BAFF, which is a cytokine that acts as a potent activator of B cells and plays an important role in the proliferation and differentiation of B cells. As the primary receptor for BAFF is expressed on mature B cells, it has been demonstrated that circulating BAFF is inversely correlated with B-cell depletion. Other serum biomarkers such as CCL19 and CXCL13 are also related to B-cell dynamics and may predict responses to B-cell depleting therapies.

T-cell Responses

There is increasing evidence for the involvement of effector T cells in the pathogenesis of NMO (reviewed in Mitsdoerffer et al, 2013). AQP4-specific antibodies in NMO serum are immunoglobulin G1 (IgG1), a subclass of mature IgG that requires help from T cells. T cells from NMO patients demonstrated greater proliferation to AQP4 and AQP4 peptide fragments than those from healthy controls (Varrin-Doyer et al, 2012). Therefore, antigen-specific T cells may contribute to the generation of AQP4-IgG in the peripheral immune compartment and to the development of NMO lesions in the CNS.

B-cell depletion, both ex vivo and in vivo, resulted in diminished proliferative and proinflammatory cytokine responses of both CD4 and CD8 T cells of MS patients (Bar-Or et al, 2010). It is mechanistically relevant to investigate whether T-cell responses are also diminished following B-cell depletion in NMO patients.

4 MATERIALS AND METHODS

4.1 Subjects

4.1.1 Number of Subjects

A maximum of 252 subjects will be randomized and dosed in this study. Because this study is event-driven where the primary objective is based on a total of 67 AC-determined NMO/NMOSD attacks, and the rate of NMO/NMOSD attacks is not established in the literature, this number of subjects was determined by analyzing masked data of the actual attack rate from the first 78 subjects who completed the RCP in this study. The attack status (attack/no attack) of the 78 subjects was randomly sampled with different attack rate, which gave an estimate of the number of attacks for the total sample size. This simulation process

was repeated 10,000 times to give a distribution of the number of attacks for the total sample size, from which the probability of observing at least 67 attacks could be estimated. Based on the 78 completed subjects, this procedure showed that with 252 subjects there is a 90% probability of reaching the required 67 AC-determined attacks.

Of the total number of subjects enrolled, approximately 80% will be AQP4-IgG seropositive and 20% will be AQP4-IgG seronegative.

4.1.2 Inclusion Criteria

Subjects must meet *all* of the following criteria:

1 Age 18 years or above at the time of screening.

2 Written informed consent and any locally required authorization (eg, Health Insurance Portability and Accountability Act [HIPAA] in the United States of America (USA), European Union [EU] Data Privacy Directive in the EU) obtained from the subject/legal representative prior to performing any protocol-related procedures, including screening evaluations

3 One of the following:

   (a) Positive serum anti-AQP4-IgG result at screening (verified by the allocated central laboratory) and a documented history of one or more NMO/NMOSD acute relapses that required rescue therapy within the last year, or 2 or more NMO/NMOSD acute relapses that required rescue therapy within 2 years prior to screening; **OR**

   (b) Negative serum anti-AQP4-IgG result at screening (verified by the allocated central laboratory) without evidence of brain lesion consistent with MS and meets the clinical criteria for NMO according to Wingerchuk et al, 2006 and a documented history of one or more NMO acute relapses that required rescue therapy within the last year, or 2 or more NMO acute relapses that required rescue therapy within 2 years prior to screening. Note that data from AQP4-IgG seronegative subjects will be reviewed by an independent eligibility committee (see Section 4.2.1.1) to confirm eligibility.
   In the event that a subject has not received rescue therapy for a relapse due to a misdiagnosis or mismanagement of symptoms at a practice or medical center outside of the investigator's control, the subject may still be eligible for the study if, following a review of the relapse data, the medical monitor and investigator are satisfied that the subject experienced a genuine relapse.

4 Subjects who have had a relapse immediately prior to screening must have at least 4 weeks in which their relapse symptoms are stable or improving prior to randomization.

5 Expanded Disability Status Scale score at randomization less than or equal to 7.5. A score of 8.0 may be eligible if the investigator and medical monitor assess that the subject is reasonably able to participate in the study

6 Females of childbearing potential who are sexually active with a nonsterilized male partner must use a highly effective method of contraception (subjects in the Czech Republic only must use 1 additional method of contraception) from screening, and must agree to continue using such precautions for 6 months after the final dose of investigational product; cessation of contraception after this point should be discussed with a responsible physician. Periodic abstinence, the rhythm method, and the withdrawal method are not acceptable methods of contraception.

(a) Females of childbearing potential are defined as those who are not surgically sterile (ie, bilateral tubal ligation, bilateral oophorectomy, or complete hysterectomy) or those who are not postmenopausal (per International Council for Harmonisation [ICH] M3(R2) 11.2: defined as 12 months with no menses without an alternative medical cause)

(b) A highly effective method of contraception is defined as one that results in a low failure rate (ie, less than 1% per year) when used consistently and correctly. The acceptable highly effective methods of contraception are described in Table 4.

7 Nonsterilized males who are sexually active with a female partner of childbearing potential must use a male condom plus spermicide (subjects in the Czech Republic only must use 1 additional method of contraception) (see Table 4) from Day 1 for 3 months after receipt of the final dose of investigational product. Because male condom and spermicide is not a highly effective contraception method, it is strongly recommended that female partners of male subjects to also use a highly effective method of contraception throughout this period.

8 Sterilized males, without the appropriate post-vasectomy documentation on the absence of sperm in the ejaculate, who are sexually active with a female partner of childbearing potential must use a condom and spermicide from Day 1 for 3 months after receipt of the final dose of investigational product.

Table 4 Highly Effective Methods of Contraception

Barrier Methods	Hormonal Methods
• Intrauterine device<br>• Intrauterine hormone-releasing system [a]<br>• Bilateral tubal occlusion<br>• Vasectomized partner [b]<br>• Sexual abstinence [c]	• Combined (estrogen and progestogen containing hormonal contraception)<br>• Oral (combined pill)<br>• Injectable<br>• Transdermal (patch)<br>• Progestogen-only hormonal contraception associated with inhibition of ovulation [d]<br>• Implantable<br>• Intravaginal

[a] This is also considered a hormonal method.

[b] With appropriate post-vasectomy documentation of surgical success (absence of sperm in ejaculate).

Table 4 Highly Effective Methods of Contraception

Barrier Methods	Hormonal Methods

[c] Sexual abstinence is considered a highly effective method only if defined as refraining from heterosexual intercourse during the entire period of the study and if it is the preferred and usual lifestyle of the subject.

[d] Progestogen-only hormonal contraception, where inhibition of ovulation is not the primary mode of action (eg, minipill), is not accepted as a highly effective method).

9 Willing to forego other forms of investigational treatment for NMO/NMOSD during the study

10 Ability and willingness to complete the study

4.1.3 Exclusion Criteria

Any of the following would exclude the subject from participation in the study:

General Exclusion Criteria

1 Any condition that, in the opinion of the investigator, would interfere with the evaluation or administration of the investigational product or interpretation of subject safety or study results

2 Concurrent/previous enrollment in another clinical study involving an investigational treatment within 4 weeks or 5 published half-lives of the investigational treatment, whichever is the longer, prior to randomization

3 An estimated glomerular filtration rate (GFR) of < 60 mL/minute

4 Lactating or pregnant females or females who intend to become pregnant anytime from signing the ICF through the study plus 6 months following last dose of investigational product

5 Known history of allergy or reaction to any component of the investigational product formulation or history of anaphylaxis following any biologic therapy

6 Evidence of alcohol, drug, or chemical abuse, or a recent history of such abuse < 1 year prior to randomization

7 Major surgery within 8 weeks prior to signing the ICF, or elective surgery planned from screening through the duration of the RCP of the study

8 Spontaneous or induced abortion, still or live birth, or pregnancy ≤ 4 weeks prior to signing the ICF

9 Subjects who are unable to undergo an MRI scan (eg, hypersensitivity to Gd-containing MRI contrast agents, implanted pacemakers, defibrillators, or other metallic objects on or inside the body that limit performing MRI scans)

10 At screening (one repeat test may be conducted to confirm results prior to randomization within the same screening period), any of the following:

(a) Aspartate transaminase (AST) > 2.5 × upper limit of normal (ULN)

(b) Alanine transaminase (ALT) > 2.5 × ULN

(c) Total bilirubin > 1.5 × ULN (unless due to Gilbert's syndrome)

(d) Platelet count < 75,000/µL (or < 75 × 10^9/L)

(e) Hemoglobin < 8 g/dL (or < 80 g/L)

(f) Glycosylated hemoglobin (HbA1c) > 8% at screening (subjects with diabetes only)

(g) CD19+ B cell counts below the lower limit of normal (LLN) according to the central laboratory

Exclusion Criteria Related to Concomitant Medications

11 Receipt of the following at any time prior to randomization:

(a) Alemtuzumab

(b) Total lymphoid irradiation

(c) Bone marrow transplant

(d) T-cell vaccination therapy

12 Receipt of rituximab or any experimental B-cell depleting agent within the 6 months prior to screening, unless the subject has B-cell counts above the LLN according to the central laboratory

13 Receipt of IVIG within 1 month prior to randomization

14 Receipt of any of the following within 3 months prior to randomization:

(a) Natalizumab (Tysabri®)

(b) Cyclosporin

(c) Methotrexate

(d) Mitoxantrone

(e) Cyclophosphamide

(f) Tocilizumab

(g) Eculizumab

15 Severe drug allergic history or anaphylaxis to two or more food products or medicine (including known sensitivity to acetaminophen/paracetamol, diphenhydramine or equivalent antihistamine, and methylprednisolone or equivalent glucocorticoid)

Exclusion Criteria Related to NMO and Other Diseases

16 AQP4-IgG seronegative subjects with a brain MRI abnormality that meets the diagnostic criteria for MS (MRIs taken at screening will be assessed centrally)

17 Uncontrolled hypertension as indicated by the treating physician and/or principal investigator

18 Any concomitant disease other than NMO that required treatment with oral or IV steroids at doses > 20 mg/day for > 21 days within the 6 months prior to screening

19 Any subjects diagnosed with a concurrent autoimmune disease that is either uncontrolled or requires the use of disease-modifying agents or immunosuppressive agents

Exclusion Criteria Related to Infection and Malignancy Risk Factors

20 Receipt of any of the following:
   (a) Any live or attenuated vaccine within 3 weeks prior to Day 1 (administration of killed vaccines is acceptable, the Sponsor recommends that investigators ensure all subjects are up to date on required vaccinations prior to study entry)
   (b) Bacillus of Calmette and Guérin (BCG) vaccine within 1 year of signing the ICF
   (c) Blood transfusion within 4 weeks prior to signing the ICF

21 Clinically significant serious active or chronic viral or bacterial infection that requires; treatment with anti-infectives, hospitalization, or, in the investigator’s opinion, represents an additional risk to the subject, within 60 days prior to randomization

22 Known history of a primary immunodeficiency (congenital or acquired) or an underlying condition such as human immunodeficiency virus (HIV) infection or splenectomy that predisposes the subject to infection

23 At screening (one repeat test may be conducted to confirm results prior to randomization within the same screening period), any of the following:
   (a) Total Ig < 600 mg/dL
   (b) Absolute neutrophil count < 1200 cells/μL
   (c) CD4 T lymphocyte count < 300 cells/μL

24 Confirmed positive test for hepatitis B/hepatitis C serology at screening:
   (a) Hepatitis B surface antigen positive,
   (b) Hepatitis B core antibody positive with negative hepatitis B surface antibody
   (c) Hepatitis C antibody positive

25 Subjects with a positive QuantiFERON®-TB Gold test, unless an appropriate course of anti-tuberculosis (TB) treatment has been documented. Subjects with an indeterminate result may be eligible if a chest x-ray shows no evidence of TB and there is no evidence of latent TB

26 History of cancer, apart from squamous cell or basal cell carcinoma of the skin treated with documented success of curative therapy > 3 months prior to randomization

4.1.4 Subject Enrollment and Randomization

4.1.4.1 Enrollment

Study participation begins (ie, a subject is "enrolled") once written informed consent is obtained and a subject identification (SID) number is assigned by a central system (eg, an interactive voice/web response system, IVRS/IWRS). Thereafter, the screening evaluations may begin to assess study eligibility (inclusion/exclusion) criteria. The SID number will be used to identify the subject during the screening process and throughout study participation, if applicable.

A master log of all consented subjects will be maintained at the site and will document all screening failures (ie, subjects who are consented but do not meet study eligibility criteria and/or are not randomized), including the reason(s) for screening failure.

Subjects who fail to meet the inclusion/exclusion criteria (ie, screening failures) should not, under any circumstances, be randomized (if applicable) or receive investigational product. There can be no exceptions to this rule. Subjects who are screening failures should be withdrawn from the study; however, they may be re-screened once in the event that the investigator believes that the subject will subsequently meet all eligibility criteria. Subjects who are rescreened will be assigned a new SID number.

Enrollment into the study will stop when one of the following occurs:

1. A total of 67 AC-determined NMOSD attacks have occurred
2. 252 subjects have been randomized and dosed
3. Recommendation by the independent DMC to cease enrollment

Informed Consent Requirements

Before enrolling into the study, all subjects must review, understand, and sign the ICF. The ICF will include detailed explanations and risk:benefit expectations from the study and will provide subjects and investigators the opportunity to discuss appropriate risk estimation and to fully understand the risks and potential benefits of this study. Study procedures may only be performed after written informed consent is obtained. Reconsent will only be required if a subject fails screening and is subsequently rescreened. Subjects may be rescreened only once.

In addition, subjects will also be required to consent to the use of their medical data and biological samples.

All subjects who enter the OLP will be required to review and sign a specific ICF that highlights the risks and potential benefits of participating in the OLP.

Subjects may give **optional** consent for collection of a deoxyribonucleic acid (DNA) sample for genetic and future research (see Section 4.1.8).

4.1.4.2 Randomization Process

An IVRS/IWRS will be used for randomization to a treatment group and assignment of masked investigational product kit numbers. A subject is considered randomized into the study when the investigator notifies the IVRS/IVWS that a subject meets eligibility criteria and the IVRS/IVWS provides the assignment of masked investigational product kit numbers for the subject.

4.1.5 Withdrawal from the Study

Subjects are at any time free to withdraw from the study (investigational product and assessments), without prejudice to further treatment (withdrawal of consent). Such subjects will always be asked about the reason(s) and the presence of any AEs.

Subjects in the RCP who express a wish to prematurely discontinue from the study will be followed for protocol-specified assessments until Day 197, unless consent is withdrawn specifically for further study participation. In this case the subject will complete the SFP unless the subject is lost to follow up, or the subject is enrolled in another clinical study. Subjects who discontinue from the OLP will enter the SFP, if possible.

In the Czech Republic only, subjects may discontinue from the investigational product and, following an Early Discontinuation Visit (EDV), subjects will enter the SFP if they agree to this safety follow up; unless the subject is lost to follow up, or the subject is enrolled in another clinical study.

Diaries and all study medications should be returned by the subject. If a subject withdraws consent from further participation in the study, then no further study visits or data collection should take place.

A subject’s decision to withdraw from treatment must be documented in the source documents.

4.1.6 Discontinuation of Investigational Product

An individual subject will not receive any further investigational product if any of the following occur in the subject in question:

1 Withdrawal of consent for further treatment with investigational product or the subject is lost to follow-up
2 An AE that, in the opinion of the investigator or the Sponsor, contraindicates further dosing

3 Subject is determined to have met one or more of the exclusion criteria or failed to meet all of the inclusion criteria for study participation and there is a potential safety risk associated with continuation identified upon consultation with the medical monitor

4 Pregnancy

5 Any of the following liver function abnormalities:

(a) ALT or AST > 8 × ULN

(b) ALT or AST > 5 × ULN for more than 2 weeks (in absence of elevated bilirubin and/or other symptoms listed in item 'd')

(c) ALT or AST > 3 × ULN and total bilirubin > 2 × ULN or international normalized ratio [INR] > 1.5; see Section 5.6.2 for additional details regarding reporting of subjects with ALT or AST > 3 × ULN and total bilirubin > 2 × ULN with unknown etiology (ie, Hy's law cases)

(d) ALT or AST > 3 × ULN with the appearance of fatigue, nausea, vomiting, right upper quadrant pain or tenderness, fever, rash, and/or eosinophilia (> 5%)

6 Any life-threatening (Grade 4) clinical event including anaphylaxis related to the investigational product as agreed upon consultation with the medical monitor

7 Recurrent severe (Grade 3) hypersensitivity reaction related to the investigational product as agreed upon consultation with the medical monitor

8 Recurrent severe (Grade 3) infusion reaction related to investigational product as agreed upon consultation with the medical monitor

9 Grade 3 or higher neutropenia that does not improve to at least Grade 2 within 5 days as agreed upon consultation with the medical monitor

10 Receipt of a prohibited medication in the RCP prior to Day 15 or during the OLP following consultation with the medical monitor (see Section 4.7.2).

11 Noncompliance to the study protocol as judged by the investigator and/or the Sponsor

Subjects who are permanently discontinued from receiving investigational product will be followed for protocol-specified assessments including follow-up of any AEs unless consent is withdrawn specifically for further study participation (Section 4.1.5), the subject is lost to follow-up, or the subject is enrolled in another clinical study.

4.1.7 Replacement of Subjects

Dropout in the RCP will not be adjusted by replacing subjects.

4.1.8 Withdrawal of Informed Consent for Donated Biological Samples

Biological Samples Obtained for the Main Study

Study data are protected by the use of an SID number, which is a number specific to the subject. The investigator is in control of the information that is needed to connect a study

sample to a subject. A subject's consent to the use of data does not have a specific expiration date, but the subject may withdraw consent at any time by notifying the investigator. If consent is withdrawn, any samples collected prior to that time may still be given to and used by the Sponsor but no new data or samples will be collected unless specifically required to monitor safety of the subject.

Samples Obtained for Genetic Research and Future Research

Samples obtained for genetic research and future research will be labeled with a sample identification number but will not be labeled with personal identifiers such as the subject's name. A file linking this sample identification number with the SID number will be kept in a secure place at the Sponsor with restricted access. If the subject withdraws consent for participating in genetic research and future research, this link will allow the Sponsor to locate the subject's sample and destroy it. The coding of samples and results is to ensure that these research results are kept confidential by keeping the subject's identity and these results separate.

If the subject consents to have his/her samples used for genetic research and future research, this additional research may not start immediately and may start at any time during the storage period. The subject's sample(s) including any specimens of extracted DNA will be stored by the Sponsor with similar samples from other subjects at a secure central laboratory. The subject's samples will not be kept for more than 25 years after the end of the study in which they were collected. If the subject chooses not to allow his/her study samples to be used for genetic research and future research, the samples will be destroyed by the Sponsor once they are no longer required for the main study.

If consent is withdrawn after a sample has been taken but before the subject's sample is sent to the Sponsor for genetic research and future research, the investigator will arrange to have it destroyed. If consent is withdrawn after the subject's sample(s) have been sent to the Sponsor for genetic research and future research, the Sponsor and the investigator will ensure that these sample(s) are destroyed unless the sample identification number has been removed and the subject can no longer be linked to any sample(s). However, if the subject's samples have already been used for research, the Sponsor is not required to destroy results of this research. In this case only the remaining sample(s) will be destroyed.

4.2 Schedule of Study Procedures

4.2.1 Enrollment/Screening Period

Table 5 shows all procedures to be conducted at the screening visit. The screening period will be up to 28 days, unless otherwise noted below, and all screening procedures must be completed within this time period. Screening assessments are presented in Table 5 and may be performed in any order following the signing of the informed consent.

Any subject who has an AQP4-IgG seronegative status at screening will have their medical history and MRI data reviewed by an independent Eligibilty Committee (see Section 4.2.1.1) to ensure that the subject meets the Wingerchuk et al, 2006 diagnostic criteria for NMO.

If any screening hematology/chemistry blood draws, or urine tests are performed within 10 days of Day 1 that are also required at Day 1, these do not need to be repeated.

The screening period may be extended by up to 28 days to allow for repeat procedures and to allow results for protocol-specified procedures and processes (including the eligibility review) to be obtained. However, if the subject cannot be assessed within the defined screening period, the subject must be withdrawn and screen failed. In addition, any subject who experiences an NMO/NMOSD relapse in screening up to and prior to randomization, should be screen failed to allow appropriate management of the relapse. Such subjects cannot be rescreened for at least 28 days from the completion of the treatment for the NMO/NMOSD relapse.

In the event a subject is re-screened a full neuroaxis MRI need not be repeated if the previous study neuroaxis MRI was conducted within the 3 months prior to the rescreening visit.

Any subject who is a screen failure may be rescreened once, unless the need to maintain the 80:20 ratio of AQP4-IgG seropositive subjects to AQP4-IgG seronegative subjects prevents them from being randomized. In exceptional circumstances an additional re-screening may be permitted, this must be documented by the Principal Investigator and is subject to approval by the medical monitor.

In the event screening is extended, blood samples for serum chemistry and hematology must be redrawn within the 28 days prior to randomization.

Written informed consent and any locally required privacy act document authorization must be obtained prior to performing any protocol-specific procedures, including screening evaluations.

Subjects undergoing screening at the time when the 252nd subject is randomized and dosed, subjects undergoing screening at the time the 67th AC determined attack occurs, and subjects undergoing screening at the time enrollment is terminated for any other reason, will not be randomized.

Table 5 Schedule of Screening Procedures

Study Period	Screening
**Week**	**-4 to -1**
**Day**	**Day -28 to Day -1**
**Procedure / Visit Number**	**1**
**Written informed consent/ assignment of SID number**	X

Table 5 Schedule of Screening Procedures

**Study Period**	**Screening**
**Week**	**-4 to -1**
**Day**	**Day -28 to Day -1**
**Procedure / Visit Number**	**1**
**Verify eligibility criteria**	X
Medical and disease history (including smoking and alcohol history)	X
C-SSRS ("Baseline/Screening" version) [a]	X
Physical/neurological examination	X
Body weight and height	X
Vital signs	X
ECG	X
Chest x-ray [b]	X
**Collect blood for:** [c]	
Serum chemistry and hematology	X
TB assessment (QuantiFERON®-TB Gold)	X
Hepatitis B, C; HIV-1, -2; virology, JCV antibody titers	X
Hemoglobin A1c (subjects with diabetes only)	X
Serum βHCG (females with childbearing potential only)	X
Whole blood for flow cytometry (B-cell count and cell subsets)	X
Serum for AQP4-IgG serostatus assay and titers	X
Total Ig, IgM, IgG, IgA, IgE	X
Collect urine for urinalysis	X
Neuroaxis MRI scan (including optic nerve)	X
Independent EDSS/FSS administration [a]	X
Independent ophthalmology examination (high-/low-contrast visual acuity test, RAPD assessment)	X
Assessment of AEs/SAEs and concomitant medications	X
Distribute subject HCRU diary and provide instructions [d]	X

AE = adverse event; AQP4-IgG = autoantibodies against aquaporin-4; βHCG = beta human chorionic gonadotropin; C-SSRS = Columbia-Suicide Severity Rating Scale; ECG = electrocardiogram; EDSS = Expanded Disability Status Scale; FSS = Functional Systems Scores; HCRU = Healthcare Resource Utilization; HIV = human immunodeficiency virus; JCV = polyomavirus John Cunningham; MRI = magnetic resonance imaging; RAPD = relative afferent pupillary defect; SAE = serious adverse event; SID = subject identification; TB = tuberculosis

[a] If the EDSS and the C-SSRS are performed by the same person, the EDSS must be done first.

[b] Chest x-ray is not mandatory unless meets the conditions described in Section 4.3.3.6.

[c] Blood draws should be fasting.

Table 5 Schedule of Screening Procedures

Study Period	Screening
**Week**	**-4 to -1**
**Day**	**Day -28 to Day -1**
**Procedure / Visit Number**	**1**

[d] Subject diaries will be distributed to aid in subject recollection of HCRU events.

4.2.1.1 Eligibility Committee

Three experts in NMO/NMOSD disease will constitute the independent Eligibility Committee. This committee has the mandate to evaluate the screening visit and medical history data from all subjects who have an AQP4-IgG seronegative result from the central lab and determine whether the subject meets the Wingerchuk et al, 2006 criteria for NMO. The committee will use its judgement and clinical experience to make its determination based on the data provided. The committee's role, function, and procedures are governed by a separate charter.

4.2.2 Treatment Period

4.2.2.1 Randomized-controlled Treatment Period

Randomization will occur on Day 1 (within 28 days of the start of screening, unless screening is extended as noted above). Table 6 shows all procedures to be conducted during the Randomized-controlled treatment period. Patient-reported outcomes should be done first followed by all other assessments/procedures in an order determined by the site. All procedures must be completed prior to administration of investigational product. If necessary, the randomization visit may be split over 2 days, in this case randomization and investigational product administration must occur on the second day. If a subject discontinues from the study early during the RCP, all procedures should be conducted as presented in Table 6 for the Day 197/ EDV. If a subject withdraws from the study, every attempt should be made to have the subject come to the site to conduct EDV assessments.

If a subject withdraws during a scheduled study visit, that visit will change to an EDV and procedures should be conducted according to those presented for Day 197/EDV. Following an EDV, all subjects should continue into the SFP.

At the time 67 AC-determined attacks have occurred or when enrollment is discontinued upon recommendation of the independent DMC based on evidence of efficacy and safety, the sponsor will notify all sites and the sites will notify all subjects who are in the RCP that these subjects must discontinue the RCP as soon as possible, preferably within 14 days, and should be given the option to enroll into the OLP. Procedures should be conducted as presented in Table 6 for the Day 197/ EDV; however, subjects who have had an MRI in the last 3 months need NOT repeat the MRI. Subjects who decide to enroll into the OLP will follow the procedures of the OLP presented in Table 7.

Table 6 Schedule of Randomized-controlled Treatment Period Study Procedures

**Study Period**	**Randomized-controlled Period**								
**Week**	**0**	**1**	**2**	**4**	**8**	**12**	**16**	**22**	**28/EDV**
**Day**	**1** [h]	**8**	**15**	**29**	**57**	**85**	**113**	**155**	**197**
**Visit Window**		**± 1d**	**± 3d**	**± 3d**	**± 3d**	**± 3d**	**± 3d**	**± 3d**	**± 7d**
**Procedure / Visit Number**	**2**	**3**	**4**	**5**	**6**	**7**	**8**	**9**	**10**
Verify eligibility criteria	X								
SF-36v2 Health Survey [a]	X					X			X
Pain NRS	X			X	X	X	X	X	X
Modified Rankin Scale	X			X	X	X	X	X	X
Medical and disease history (including smoking and alcohol history)	X								
C-SSRS ("Since Last Visit" version) [g]	X	X	X	X	X	X	X	X	X
Physical/neurological examination	X	X	X	X	X	X	X	X	X
Body weight	X					X			X
Vital signs	X	X	X	X	X	X	X	X	X
ECG	X								X
**Collect blood for:**									
Hematology and serum chemistry	X	X	X	X	X	X	X	X	X
MEDI-551 concentration (PK)	X [b]	X	X [b]	X	X	X	X	X	X
MEDI-551 ADA	X			X		X			X
Total Ig, IgM, IgG, IgA, IgE	-					X			X
Whole blood for flow cytometry (B-cell count and cell subsets)	X	X	X	X	X	X	X	X	X
Serum for AQP4-IgG assay (titers)	X		X	X	X	X			X
Whole blood for gene expression	X		X	X	X	X	X		X
Whole blood for DNA sample (optional)	X								

Table 6 Schedule of Randomized-controlled Treatment Period Study Procedures

**Study Period**	**Randomized-controlled Period**								
**Week**	**0**	**1**	**2**	**4**	**8**	**12**	**16**	**22**	**28/EDV**
**Day**	**1** [h]	**8**	**15**	**29**	**57**	**85**	**113**	**155**	**197**
**Visit Window**		**± 1d**	**± 3d**	**± 3d**	**± 3d**	**± 3d**	**± 3d**	**± 3d**	**± 7d**
**Procedure / Visit Number**	**2**	**3**	**4**	**5**	**6**	**7**	**8**	**9**	**10**
Serum for exploratory biomarkers	X		X	X	X	X	X	X	X
Vaccination titers (tetanus) [i]	X								X
**Collect urine for:**									
Urine HCG (females with childbearing potential only) (pre-IV dose only) [c]	X		X						X
Urinalysis	X	X	X	X	X	X	X	X	X
Assess subject for new/worsening symptoms related to NMO/NMOSD [d, e]	X	X	X	X	X	X	X	X	X
Independent EDSS/FSS administration [g]	X					X			X
Independent ophthalmology examination (high-/low-contrast visual acuity test, RAPD assessment)	X					X			X
Neuroaxis MRI (including optic nerve)									X
Randomize subjects in IVRS	X								
**Investigational product administration**	X		X [j]						
Dispense/monitor low-dose steroids	X	X	X	X					
Assessment of AEs/SAEs (including infusion reactions)	X	X	X	X	X	X	X	X	X
Concomitant medications	X	X	X	X	X	X	X	X	X
Dispense and review subject HCRU diaries [f]	X	X	X	X	X	X	X	X	X

Table 6 Schedule of Randomized-controlled Treatment Period Study Procedures

Study Period	Randomized-controlled Period								
**Week**	**0**	**1**	**2**	**4**	**8**	**12**	**16**	**22**	**28/EDV**
**Day**	**1** [h]	**8**	**15**	**29**	**57**	**85**	**113**	**155**	**197**
**Visit Window**		**± 1d**	**± 3d**	**± 3d**	**± 3d**	**± 3d**	**± 3d**	**± 3d**	**± 7d**
**Procedure / Visit Number**	**2**	**3**	**4**	**5**	**6**	**7**	**8**	**9**	**10**
Follow-up telephone call	Telephone calls every 2 weeks will commence from Day 43 and continue for the duration of the RCP, except for study visit weeks								

ADA = anti-drug antibody; AE = adverse event; AQP4-IgG = autoantibodies against aquaporin-4; C-SSRS = Columbia-Suicide Severity Rating Scale; d = day(s) D/C = discontinuation; ECG = electrocardiogram; EDSS = Expanded Disability Status Scale; EDV = Early Discontinuation Visit; FSS = Functional Systems Scores; HCG = human chorionic gonadotropin; HCRU = Healthcare Resource Utilization; Ig A/E/G/M = immunoglobulin A/E/G/M; IV = intravenous; IVRS = interactive voice response system; MRI = magnetic resonance imaging; NMO/NMOSD = neuromyelitis optica/neuromyelitis optica spectrum disorders; NRS = numeric rating scale; PK = pharmacokinetic; RAPD = relative afferent pupillary defect; RCP = Randomized-controlled Period; SAE = serious adverse event; SF-36v2 = Short Form-36 Health Survey, version 2

Note: All procedures to be performed prior to randomization.

[a] SF-36v2 Health Survey 4-week recall to be used at all time points. The 1-week recall version will also be used for an attack.

[b] Pharmacokinetic blood samples to be collected predose and approximately 15 minutes postdose (± 5 minutes) after completion of MEDI-551 or placebo administration.

[c] Urine HCG to be followed up to 26 weeks following discontinuation.

[d] If a subject has an NMO/NMOSD attack prior to Day 1, and the subject has not been randomized, the visit should not continue. The subject should be treated for the attack as required, screen failed, and then reassessed for eligibility once the subject's condition is stable.

[e] In the event that an NMO/NMOSD attack is suspected, procedures in Section 4.2.3 should be followed.

[f] Subject diaries will be distributed to aid in subject recollection of HCRU events.

[g] If the EDSS and the C-SSRS are performed by the same person, the EDSS must be done first.

[h] If necessary, the randomization visit may be split over 2 days, in this case randomization and investigational product administration must occur on the second day.

[i] Tetanus vaccine titers will be tested at Day 1 for all subjects. Subjects with a negative result will not continue to be tested. Subjects who test positive will continue to be tested at all specificed vaccination titer timepoints.

[j] In the event that the second dose of investigational product is delayed due to medical/safety reasons, dosing must be discussed with the medical monitor prior to administration of investigational product.

4.2.2.2 Open-label Period

Table 7 shows all procedures to be conducted during the OLP. Patient-reported outcomes should be done first followed by all other assessments/procedures in an order determined by the site. For subjects completing Day 197 of the RCP, Day 1 of the OLP should be the same day; however, it may be delayed for up to 14 days (procedures do not need to be repeated). Subjects are not permitted to enter the OLP after 14 days, unless there is a compelling reason that is discussed with, and agreed to by the medical monitor, in which case a short extension may be granted.

Subjects who experience an AC-determined NMO/NMOSD attack during the RCP must enter the OLP within 28 days of the site receiving confirmation of the attack from the AC. Entering subjects into the OLP beyond the 28 days from the AC confirmation should be discussed with the medical monitor. Procedures that were done during the last visit can be used for Day 1 of the OLP if this occurs within 14 days from the Assessment Visit. Otherwise, procedures required for OLP Day 1 should be performed.

Subjects who are in the RCP at the time the 67th AC-determined attack occurs, or when enrollment is discontinued upon recommendation of the independent DMC based on evidence of efficacy and safety, and wish to enroll in the OLP, should do so as soon as possible, preferably within 14 days. Consulation with the medical monitor is advised in cases where the transition to the OLP is not completed within 14 days.

Administration of MEDI-551 will start on OLP Day 1.

The OLP will continue for a minimum of 1 year after the last subject enters and a maximum of 3 years (after the last subject enters), or until regulatory approval for MEDI-551 in the participating country, or until the Sponsor discontinues development of MEDI-551 in this indication, whichever occurs first. All subjects entering the OLP will be required to reconsent prior to receiving MEDI-551. Subjects can choose to exit the OLP at any time, for any reason, including seeking alternative treatment options, at which point they will enter the SFP (unless consent is withdrawn).

If a subject discontinues from the OLP, all procedures should be conducted as presented in Table 7 for the Day 365/EDV. If a subject withdraws from the study, every attempt should be made to have the subject come to the site to conduct EDV assessments. Following the EDV, the subject will enter the SFP (Section 4.2.4).

If a subject withdraws during a scheduled study visit, that visit will change to an EDV and procedures should be conducted according to those presented for Day 365/EDV for the OLP.

At the point the Sponsor decides to terminate the OLP, the next visit for all ongoing subjects in that period will be an EDV to capture all assessments; this would also represent the first visit of the SFP.

Table 7 Schedule of Open-label Period Study Procedures

**Study Period**	**Open-label Period**						
**Week**	**0**	**2**	**4**	**13**	**26**	**39, then Q26W**	**52, then Q26W/EDV**
**Day**	**1**	**15**	**29**	**92**	**183**	**274+**	**365+**
**Visit Window**	**(+ 14d from Day 197 RCP or +28d AC Determination)**	**± 3d**	**± 3d**	**± 14d**	**± 7d**	**± 14d**	**± 7d**
**Procedure/Visit Number**	**1**	**2**	**3**	**4**	**5**	**6, 8,10, etc**	**7, 9, 11, etc**
Written informed consent	X						
SF-36v2 Health Survey				X	X	X	X
Pain NRS			X	X	X	X	X
Modified Rankin Scale			X	X	X	X	X
Physical/neurological examination	X [a]	X	X	X	X	X	X
C-SSRS ("Since Last Visit" version) [h]	X [a]	X	X	X	X	X	X
Vital signs	X [a]	X	X	X	X	X	X
**Collect blood for:**							
Hematology and serum chemistry	X [a]	X	X	X	X	X	X
MEDI-551 ADA	X [a]			X	X	X	X
Total Ig, IgM, IgG, IgA, IgE	X [a]			X	X	X	X
Whole blood for flow cytometry (B-cell count and cell subsets)		X	X	X	X	X	X
Serum for AQP4-IgG assay (titers)	X [a]		X	X	X		X
Whole blood for gene expression			X	X	X		X
Serum for exploratory biomarkers		X	X	X	X	X	X
Vaccination titers (tetanus) [j]				X	X		X

Table 7 Schedule of Open-label Period Study Procedures

**Study Period**	**Open-label Period**						
**Week**	**0**	**2**	**4**	**13**	**26**	**39, then Q26W**	**52, then Q26W/EDV**
**Day**	**1**	**15**	**29**	**92**	**183**	**274+**	**365+**
**Visit Window**	**(+ 14d from Day 197 RCP or +28d AC Determination)**	**± 3d**	**± 3d**	**± 14d**	**± 7d**	**± 14d**	**± 7d**
**Procedure/Visit Number**	**1**	**2**	**3**	**4**	**5**	**6, 8,10, etc**	**7, 9, 11, etc**
**Collect urine for:**							
Urine HCG (females with childbearing potential only; pre-IV dose only)	X [b]	X			X		X
Urinalysis	X [a]	X	X	X	X	X	X
Assess subject for new/worsening symptoms related to NMO/NMOSD [c]	X [b]	X	X	X	X	X	X
Independent EDSS/FSS administration [h]	-			X	X	X	X
Independent ophthalmology examination (high-/low-contrast visual acuity test, RAPD assessment)				X	X	X	X
Neuroaxis MRI (including optic nerve)	(X) [d]						X [e]
**Investigational product administration**	X [b]	X [i]			X		X [g]
Assessment of AEs/SAEs (including infusion reactions)	X [b]	X	X	X	X	X	X
Concomitant medications	X [b]	X	X	X	X	X	X
Dispense and review subject HCRU diary entries [f]	X [b]	X	X	X	X	X	X

Table 7 Schedule of Open-label Period Study Procedures

Study Period	Open-label Period						
Week	0	2	4	13	26	39, then Q26W	52, then Q26W/EDV
Day	1	15	29	92	183	274+	365+
Visit Window	(+ 14d from Day 197 RCP or +28d AC Determination)	± 3d	± 3d	± 14d	± 7d	± 14d	± 7d
Procedure/Visit Number	1	2	3	4	5	6, 8,10, etc	7, 9, 11, etc

AC = Adjudication Committee; ADA = anti-drug antibody; AE = adverse event; AQP4-IgG = autoantibodies against aquaporin-4; C-SSRS = Columbia-Suicide Severity Rating Scale; d = day(s); EDSS = Expanded Disability Status Scale; EDV = Early Discontinuation Visit; FSS = Functional Systems Scores; HCG = human chorionic gonadotropin; HCRU = Healthcare Resource Utilization; Ig A/E/G/M = immunoglobulin A/E/G/M; IV = intravenous; MRI = magnetic resonance imaging; NMO/NMOSD = neuromyelitis optica/neuromyelitis optica spectrum disorders; NRS = numeric rating scale; OLP = Open-label Period; Q26W = every 26 weeks; RAPD = relative afferent pupillary defect; RCP = Randomized-controlled Period; SAE = serious adverse event; SF-36v2 = Short Form-36 Health Survey, version 2

[a] For subjects who enter the OLP prior to Day 197 of the RCP due to an AC-determined NMO/NMOSD attack and 14 days have elapsed since the Assessment Visit, all procedures listed for Day 1 should be repeated. For subjects completing Day 197 of the RCP, Day 1 of the OLP should be the same day; however, it may be delayed for up to 14 days and procedures do not need to be repeated.

[b] Conduct for all subjects.

[c] In the event that an NMO/NMOSD attack is suspected, procedures in Section 4.2.3 should be followed.

[d] If the subject experienced an NMO/NMOSD attack during the RCP, any part of the neuroaxis MRI that was not done at the time of the Assessment Visit should be conducted at Day 1 of the OLP, otherwise it is not required.

[e] An MRI will be conducted yearly.

[f] Subject diaries will be distributed to aid in subject recollection of HCRU events.

[g] If an EDV is being conducted, investigational product will not be given.

[h] If the EDSS and the C-SSRS are performed by the same person, the EDSS must be done first.

[i] In the event that the second dose of investigational product is delayed due to a medical/safety reason, dosing must be discussed with the medical monitor prior to administration of investigational product.

[j] Tetanus vaccine titers will only be tested in the OLP for subjects who tested positive at Day 1 of the RCP.

4.2.3 Study Procedures for Assessment Visit

Table 8 shows all study procedures to be conducted at Assessment Visits for subjects experiencing new or worsening symptom(s) potentially related to NMO/NMOSD. An Assessment Visit should be scheduled as soon as possible and within 72 hours of symptom report. Procedures to determine whether symptoms are related to NMO/NMOSD should be conducted first, followed by procedures for determination of an NMO/NMOSD attack. All clinical assessments need to be documented with time and date, and the order of the assessments should be determined by the nature of the suspected attack (eg, EDSS before Independent ophthalmology in case of myelitis symptoms). An MRI of all domains should be performed as part of an Assessment Visit and can be done at any time during the Assessment Visit. The order of the MRI domains performed can be determined by the nature of the suspected attack. MRI images/study report should not be reviewed by the Principal Investigator unless a specific criterion for an attack requires review of the MRI. In such cases review of the MRI image MUST be done AFTER the review of all relevant clinical assessment data (eg, EDSS in the case of myelitis/brainstem/brain symptoms or ophthalmology in the case of optic neuritis symptoms) is completed. Assessments should be concluded as soon as possible, but should not extend beyond 4 days from Day 1 of the Assessment Visit.

If the investigator determines that the new or worsening symptom(s) are not related to NMO/NMOSD, then the independent assessments (EDSS, Independent ophthalmology, and MRI) are not required.

Table 8 Schedule of Study Procedures for the Assessment Visit

Procedure	Assessment Visit [a]
Pain NRS	X
Vital signs	X
Modified Rankin Scale	X
Physical/neurological examination	X
**Collect blood for:**	
Hematology and serum chemistry	X
MEDI-551 ADA	X
Total Ig, IgM, IgG, IgA, IgE	X
Whole blood for flow cytometry (B-cell count and cell subsets)	X
Serum for AQP4-IgG assay (titers)	X
Whole blood for gene expression	X
Serum for exploratory biomarkers	X
Collect urine for urinalysis	X
**Procedures for determination of an NMO/NMOSD attack:**	
Independent ophthalmology examination (high-/low-contrast visual acuity test, RAPD assessment)	X
Independent EDSS/FSS administration [c]	X
Neuroaxis MRI scan [b]	X
SF-36 (1-week recall version)	X
C-SSRS (“Since Last Visit” version) [c]	X
ECG	X
Review data generated against protocol defined attack criteria and create a narrative of the Assessment Visit findings	X

Table 8 Schedule of Study Procedures for the Assessment Visit

Procedure	Assessment Visit [a]
Assessment of AEs and SAEs	X
Concomitant medications	X
Review subject diary entries	X

ADA = anti-drug antibody; AE = adverse event; AQP4-IgG = autoantibodies against aquaporin-4; C-SSRS = Columbia Suicide Severity Rating Scale; ECG = electrocardiogram; EDSS = Expanded Disability Status Scale; FSS = Functional Systems Scores; Ig A/E/G/M = immunoglobulin A/E/G/M; MRI = magnetic resonance imaging; NMO/NMOSD = neuromyelitis optica/ neuromyelitis spectrum disorders; NRS = numeric rating scale; RAPD = relative afferent pupillary defect; SAE = serious adverse event; SF-36 = Short Form-36 Health Survey

[a] Visit to be conducted within 72 hours of onset of report to site of new symptom(s) or worsening of an existing symptom(s) potentially related to NMO/NMOSD; visit may be conducted over 5 days.

[b] The MRI image/report is only reviewed by the Principal Investigator in the event it is required by the protocol-defined attack criteria.

[c] If the EDSS and the C-SSRS are performed by the same person, the EDSS must be done first.

4.2.4 Managing Subjects With Worsening Attacks

Following the diagnosis of an attack by the Principal Investigator, the attack symptoms and findings may get worse. In this case, the assessments and management will be done in accordance with the standard of care at the site. The change in symptoms/findings, assessments completed, and treatments administered should all be recorded in the electronic case report form (eCRF).

4.2.5 Study Procedures for Attack Follow-up Visit

Subjects who experience an NMO/NMOSD attack that meets the protocol-defined criteria and requires rescue treatment, regardless of the outcome of the AC review, will undergo an Attack Follow-up Visit. Table 9 shows procedures to be conducted for the Attack Follow-up Visit. Patient reported outcomes should be done first followed by all other assessments/procedures in an order determined by the site. An Attack Follow-up Visit should be conducted 28 days from Day 1 of the Assessment Visit. This visit may correspond with an OLP or SFP visit or may be scheduled separately.

Table 9 Schedule of Study Procedures for the Attack Follow-up Visit

Procedure	Follow-up Assessment Visit [a]
**Visit Window**	**28 days (± 7 days) After Assessment Visit**
Pain NRS	X
Vital signs	X
Modified Rankin Scale	X
SF-36 Health Survey (1-week recall version)	X
C-SSRS ("Since Last Visit" version) [c]	X
Physical/neurological examination	X
**Collect blood for:**	
Hematology and serum chemistry	X
Whole blood for flow cytometry (B-cell count and cell subsets)	X
Serum for AQP4-IgG assay (titers)	X
Collect urine for urinalysis	X
Independent ophthalmology examination (high-/low-contrast visual acuity test, RAPD assessment)	X [b]
Independent EDSS/FSS administration	X [b, c]
Assessment of AEs and SAEs	X
Concomitant medications	X

Table 9 Schedule of Study Procedures for the Attack Follow-up Visit

Procedure	Follow-up Assessment Visit [a]

ADA = anti-drug antibody; AE = adverse event; AQP4-IgG = autoantibodies against aquaporin-4; C-SSRS = Columbia Suicide Severity Rating Scale; EDSS = Expanded Disability Status Scale; FSS = Functional Systems Scores; Ig A/E/G/M = immunoglobulin A/E/G/M; NRS = numeric rating scale; ON = optic neuritis; RAPD = relative afferent pupillary defect; SAE = serious adverse event; SF-36 = Short Form-36 Health Survey.

[a] The Follow-up Assessment Visit may be combined with an OLP or SFP visit if it occurs within the time window allowed for this visit.

[b] Conduct the assessment(s) relevant to the type of attack only (eg, for ON attacks conduct the ophthalmology examination only).

[c] If the EDSS and the C-SSRS are performed by the same person, the EDSS must be done first.

4.2.6 Safety Follow-up Period

Table 10 shows all procedures to be conducted during the SFP.

The SFP will start when a subject prematurely discontinues from the RCP or the OLP. The length of the SFP will be determined by the time elapsed from the time of the last dose of the investigational product to the time of the premature discontinuation, to complete a total of 52 weeks. Subjects who prematurely discontinue during the RCP will continue with the study assessments until Day 197, unless the consent was specifically withdrawn for further study assessments.

Table 10 Schedule of Procedures for Safety Follow-up Period

Study Period	Follow-up Period
**Visit Number**	**Every 3 months for a total of 1 year after last dose of investigational product (± 14d)**
**Procedure/Study**	
Vital signs	X
C-SSRS ("Since Last Visit" version)	X
Whole blood for flow cytometry (B-cell count and cell subsets)	X
Total Ig, IgM, IgG, IgA, and IgE	X
MEDI-551 ADA	X
Assessment of AEs/SAEs	X

ADA = anti-drug antibody; AE = adverse event; C-SSRS = Columbia Suicide Severity Rating Scale; d = day(s); Ig A/E/G/M = immunoglobulin A/E/G/M; SAE = serious adverse event

4.3 Description of Study Procedures

4.3.1 Efficacy

4.3.1.1 Identification, Assessment, and Adjudication of an NMO/NMOSD Attack

Overview

The process for identification, assessment, and adjudication of an NMO/NMOSD attack is presented in Figure 3 and requires that subjects reporting a new or worsening symptom(s) of a potential NMO/NMOSD attack must be evaluated at an Assessment Visit at the clinical site by the investigator as soon as possible, but within 72 hours of the report. Following the start of the Assessment Visit, the procedures required to diagnose an NMO/NMOSD attack per the protocol-defined attack criteria must be completed within a total of 5 days following the start of the Assessment Visit.

Figure 3 Flow Chart of Assessment and Diagnosis of an NMO/NMOSD Attack

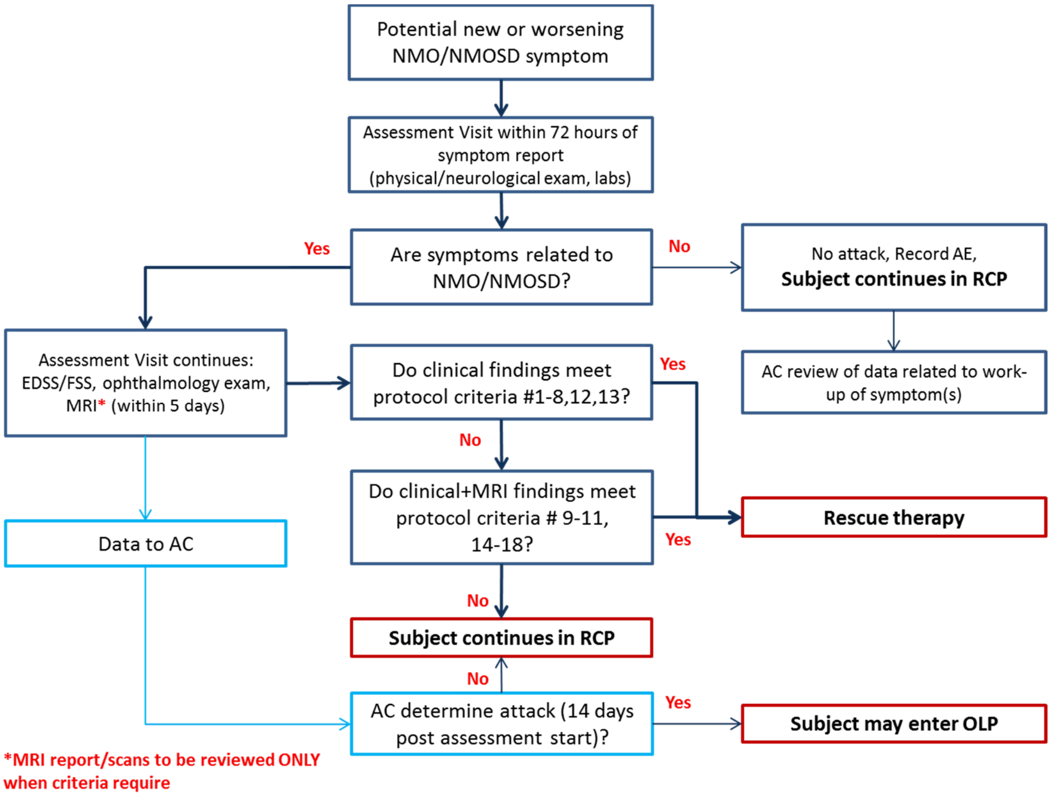

AC = Adjudication Committee; AE = adverse event; EDSS = Expanded Disability Status Scale; FSS = Functional Systems Scores; MRI = magnetic resonance imaging; NMO/NMOSD = neuromyelitis optica/neuromyelitis optica spectrum disorders; OLP = Open-label Period; RCP = Randomized-controlled Period.

The Assessment Visit is designed to determine if an attack per the protocol criteria has occurred. There are two main outcomes of the Assessment Visit:

1 The investigator determines that the symptoms ARE related to NMO/NMOSD and completes the full attack assessment. In this case specified data generated at the Assessment Visit, regardless of whether or not the investigator determined that a protocol-defined NMO/NMOSD attack has occurred, will be submitted to the AC for a real-time review.

2 The investigator determines that the symptoms ARE NOT related to NMO/NMOSD. In this case the data related to the assessment of the symptoms will also be sent to the AC for review (not in real time).

The adjudication process requires that adjudication will be completed within a total of 14 days (+ 3 days) of the start of the Assessment Visit. The determination of the AC as to whether an NMO/NMOSD attack has occurred will be communicated to the clinical site. Only subjects where their attack was determined by the AC will be eligible to enroll into the OLP.

Rescue therapy for an NMO/NMOSD attack will be given by the investigator according to the investigator's clinical judgment that the subject is experiencing an NMO/NMOSD attack. Rescue therapy will not be affected by the AC decision as to whether the NMO/NMOSD attack has or has not been determined. Subjects who receive rescue therapy for an NMO/NMOSD attack that was determined by the AC will have the option to enroll into the OLP. Subjects whose attack was not determined by the AC will have the option to continue in the RCP.

There may be an interval of a few days between the completion of the rescue therapy and the completion of the adjudication process. This period probably does not present a safety concern (possible NMO/NMOSD attack) as the rescue therapy would have only just concluded, and the effects of the rescue therapy will cover the period before the OLP starts.

Detailed Procedures

Identification

There are 3 possibilities for identifying new or worsening symptoms that may relate to an NMO/NMOSD attack:

- The subject reports the symptom directly to the site
- A symptom is identified during a study visit
- A symptom is identified during the telephone call every 2 weeks to the subject

Telephone Calls to Subjects

The investigator or designee will contact all subjects by telephone every 2 weeks, commencing from Day 43 of the RCP through the end of the subject's participation in the RCP (except where timing coincides with study visit weeks).

The purpose of these calls is to assess for any signs and/or symptoms of an NMO/NMOSD attack (examples of possible symptoms are presented in Table 11). The calls will follow a prespecified script.

If the investigator suspects an NMO/NMOSD attack or impending attack, an Assessment Visit will be scheduled within 72 hours.

Recording Symptoms of NMO/NMOSD

Details of any symptoms of NMO/NMOSD that occur or worsen during the study will be recorded at the Assessment Visit. Any symptom determined as not related to NMO/NMOSD should be reported as an AE.

Information regarding new or worsening symptoms may be provided during study visits (including follow-up by the site owing to a missed study visit), during scheduled bi-weekly telephone calls, or from spontaneous reporting from the subject at any time during the study.

In any of the above situations, information will be elicited from the subjects as to whether they have had any changes in their NMO/NMOSD status since the last contact with the site. A standardized worksheet will be used by all sites in order to elicit consistent information in the same manner.

If the investigator suspects an NMO/NMOSD attack or impending attack, an Assessment Visit will be scheduled within 72 hours.

Information to be recorded at the Assessment Visit includes the following: date of contact, each symptom, date and time of onset, date of resolution, treatment administered, date of assessment visit scheduled, and whether the symptom was not related to NMO/NMOSD but to an AE, or an SAE (see Section 5.5, Reporting of Serious Adverse Events).

Assessment Visit

The investigator initially conducts a physical and neurological examination as described in Section 4.3.3.2. If the symptom(s) is assessed by the investigator *as not related* to NMO/NMOSD, an AE must be reported (see Section 5.4) and the subject will continue in the RCP or the OLP, as applicable, without further action. The data related to the assessment of the symptoms that were determined by the investigator as not related to NMO/NMOSD will be sent to the AC for non-real-time review.

If the symptom(s) is assessed as related to NMO/NMOSD, full assessment of the potential NMO/NMOSD attack will be conducted and the investigator will determine if an attack fulfilling the protocol-defined criteria for an NMO/NMOSD attack has occurred (Table 11). The date and time of all clinical assessments, conducted by the independent assessors, must be documented, and the order of the assessments should be driven by the nature of the suspected

attack (eg, EDSS before Independent ophthalmology in case of myelitis symptoms). MRI of all domains should be performed as part of an Assessment Visit and can be done at any time during the Assessment Visit. The order of the MRI domains performed can be determined by the nature of the suspected attack. MRI images/study report should not be reviewed by the Principal Investigator unless a specific criterion for an attack requires review of the MRI.

Following completion of the independent clinical assessment, the Principal Investigator should decide if any of the CLINICAL (without MRI) criteria (numbers 1 to 8, 12 and 13) are met. Within one domain (optic nerve, spinal cord, brain or brainstem), the clinical criterion with the most significant change should be selected. Only in cases where none of the CLINICAL criteria are met, criteria that include MRI review can be selected (numbers 9 to 11, 14 to 18). Only in this situation is MRI review of the relevant domain allowed.

If the attack presents in more than one domain (optic nerve, spinal cord, brain or brainstem), more than one criterion can be selected.

Table 11 Protocol-defined Criteria for an NMO/NMOSD Attack

Example Symptoms of an NMO/NMOSD Attack [a]	Attack Type [b]	Protocol-defined Attack Criteria
Blurred vision<br>Loss of vision<br>Eye pain	ON	1. Greater than 15-character drop in high-contrast Landolt C Broken Rings Chart from last visit as measured in a previously affected eye and no other ophthalmological explanation<br>2. At least 2-step drop [c] in CF to NLP from last visit as measured in a previously affected eye and no other ophthalmological explanation<br>3. At least 7 or more character drop in low-contrast Landolt C Broken Rings Chart from last visit as measured in either eye alone (monocular) AND a new RAPD in affected eye<br>4. At least 7 or more character drop in low-contrast Landolt C Broken Rings Chart from last visit as measured in either eye alone (monocular) AND loss of a previously documented RAPD in fellow eye<br>5. At least 5 or more character drop in high-contrast Landolt C Broken Rings Chart from last visit as measured in either eye alone (monocular) AND a new RAPD in affected eye<br>6. At least 5 or more character drop in high-contrast Landolt C Broken Rings Chart from last visit as measured in either eye alone (monocular) AND loss of a previously documented RAPD in fellow eye<br>7. At least 1-step drop [d] in CF to NLP from last visit as measured in a previously affected eye AND a new RAPD in affected eye<br>8. At least 1-step drop [d] in CF to NLP from last visit as measured in a previously affected eye AND loss of a previously documented RAPD in fellow eye<br>9. At least 7 or more character drop in low-contrast Landolt C Broken Rings Chart from last visit as measured in either eye alone (monocular) AND a new Gd-enhancing or new/enlarging T2 MRI lesion in the corresponding optic nerve [f]<br>10. At least 5 or more character drop in high-contrast Landolt C Broken Rings Chart from last visit as measured in either eye alone (monocular) AND a new Gd-enhancing or new/enlarging T2 MRI lesion in the corresponding optic nerve [f]<br>11. At least 1-step drop [d] in CF to NLP from last visit as measured in a previously affected eye **AND** a new Gd-enhancing or new/enlarging T2 MRI lesion in the corresponding optic nerve [f]
Deep or radicular pain<br>Extremity paraesthesia<br>Weakness<br>Sphincter dysfunction	Myelitis [e]	12. At least 2-point worsening in 1 or more of the relevant (pyramidal, bladder/bowel, sensory) FSS compared to last visit<br>13. At least 1-point worsening in EDSS score compared to last visit if previous EDSS score is 5.5 or more

Table 11 Protocol-defined Criteria for an NMO/NMOSD Attack

Example Symptoms of an NMO/NMOSD Attack [a]	Attack Type [b]	Protocol-defined Attack Criteria
Lhermitte's sign (not in isolation)		14. At least 1-point worsening in 2 or more of the relevant (pyramidal, bladder/bowel, sensory) FSS compared to last visit when the last visit score was 1 or greater AND a new Gd-enhancing or new/enlarging T2 MRI lesion in the spinal cord<br>15. At least 0.5-point worsening in EDSS score compared to last visit if previous EDSS score is 5.5 or more **AND** a new GD-enhancing or new/enlarging T2 MRI lesion in the spinal cord
Nausea<br>Intractable vomiting<br>Intractable hiccups<br>Other neurological signs (eg, double vision, dysarthria, dysphagia, vertigo, oculomotor palsy, weakness, nystagmus, other cranial nerve abnormality)	Brainstem	16. Isolated (not present at last visit) intractable nausea, vomiting, and/or hiccups lasting for greater than 48 hours AND a new Gd-enhancing or new/enlarging T2 MRI lesion in the brainstem<br>17. At least 2-point worsening in 1 or more of the relevant (brainstem, cerebellar) FSS compared to last visit AND a new Gd-enhancing or new/enlarging T2 MRI lesion in the brainstem
Encephalopathy<br>Hypothalamic dysfunction	Brain	18. At least 2-point worsening in 1 or more of the relevant (cerebral, sensory, pyramidal) FSS (with a score of 3 or more at the current visit) compared to last visit **AND** a new Gd-enhancing or new/enlarging T2 MRI lesion in the brain consistent with the clinical presentation

CF = counting fingers; EDSS = Expanded Disability Severity Score; FSS = Functional System Scores; Gd = gadolinium; HM = hand motion; LP = light perception; MRI = magnetic resonance imaging; NLP = no light perception; NMO/NMOSD = neuromyelitis optica/neuromyelitis optica spectrum disorders; ON = optic neuritis; RAPD = relative afferent pupillary defect.

[a] The symptoms listed are examples and are not inclusive of all NMO/NMOSD symptoms.

[b] Four major areas of the body may be affected by an attack: the optic nerve, resulting in ON; the spinal cord, resulting in myelitis; the brainstem, resulting in a number of outcomes; and the brain.

[c] At least 2-step drop can be any of the following worsening: on Landolt C Broken Rings Chart to HM, LP, or NLP; CF to LP or NLP; HM to NLP.

[d] At least 1-step drop can be any of the following worsening: on Landolt C Broken Rings Chart to CF, HM, LP, or NLP; CF to HM or LP or NLP; HM to LP or NLP; LP to NLP.

[e] Note: A 1-point change in a single FSS without a change in the EDSS, with or without a new Gd-enhancing or new/enlarging T2 MRI lesion in the spinal cord, is not considered a clinically significant change and will not count as an attack per this protocol.

[f] Lesions seen in the optic chiasm also count towards these criteria.

Evaluations of the symptom(s) assessed as related to NMO/NMOSD to identify an NMO/NMOSD attack in accordance with the protocol-defined criteria will include the following:

- Refer for ophthalmology examinations (see Section 4.3.1.3)
- An independent EDSS/Functional Systems Scores (FSS) assessment (see Section 4.3.1.4)
- Conduct an MRI (MRI images/study report should not be reviewed by the Principal Investigator unless a specific criterion for an attack requires review of the MRI. In such cases, review of the MRI image MUST be done AFTER the review of all relevant clinical assessment data is complete).

The following flow diagrams illustrate the processes for evaluating symptoms affecting the eye (Figure 4), symptoms affecting the spinal cord (Figure 5), and symptoms affecting the brain or brainstem (Figure 6).

Figure 4 Process for Assessing Symptoms Affecting the Eye

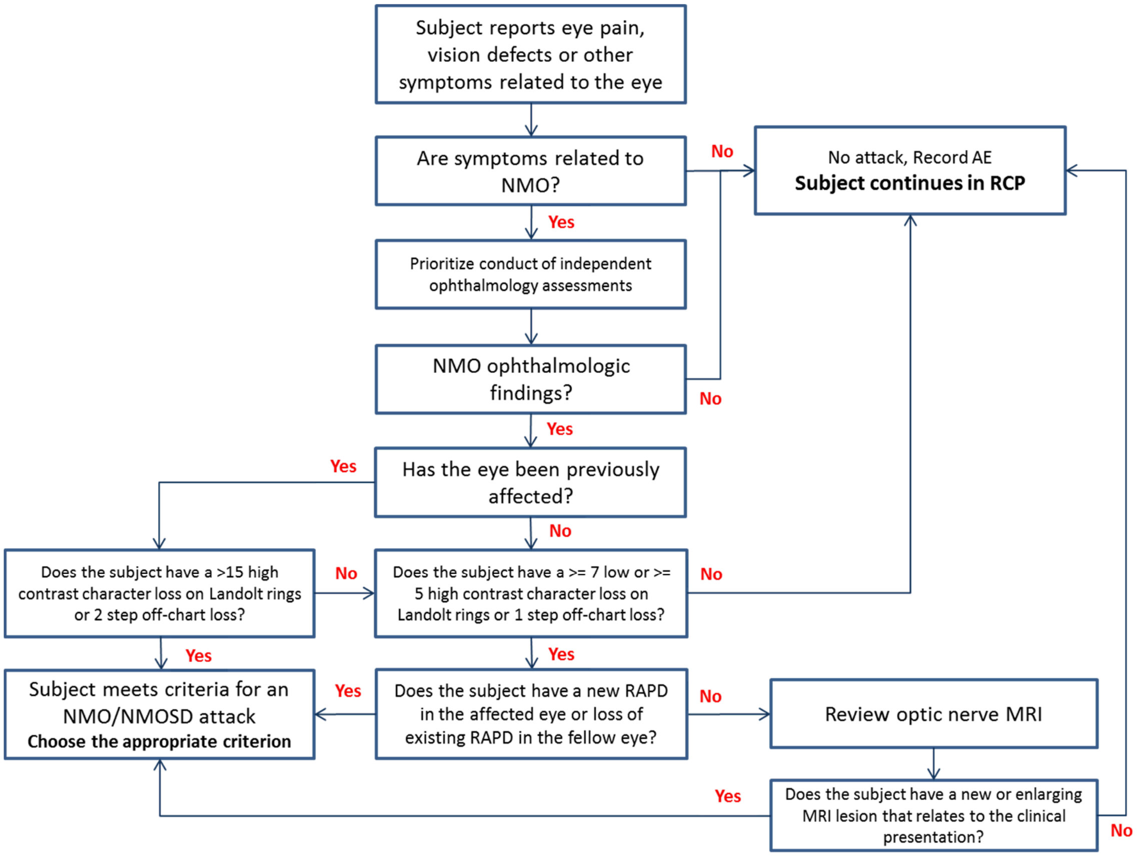

AE = adverse event; MRI = magnetic resonance imaging; NMO = neuromyelitis optica; RAPD = relative afferent pupillary defect.

Figure 5 Process for Assessing Symptoms Affecting the Spinal Cord

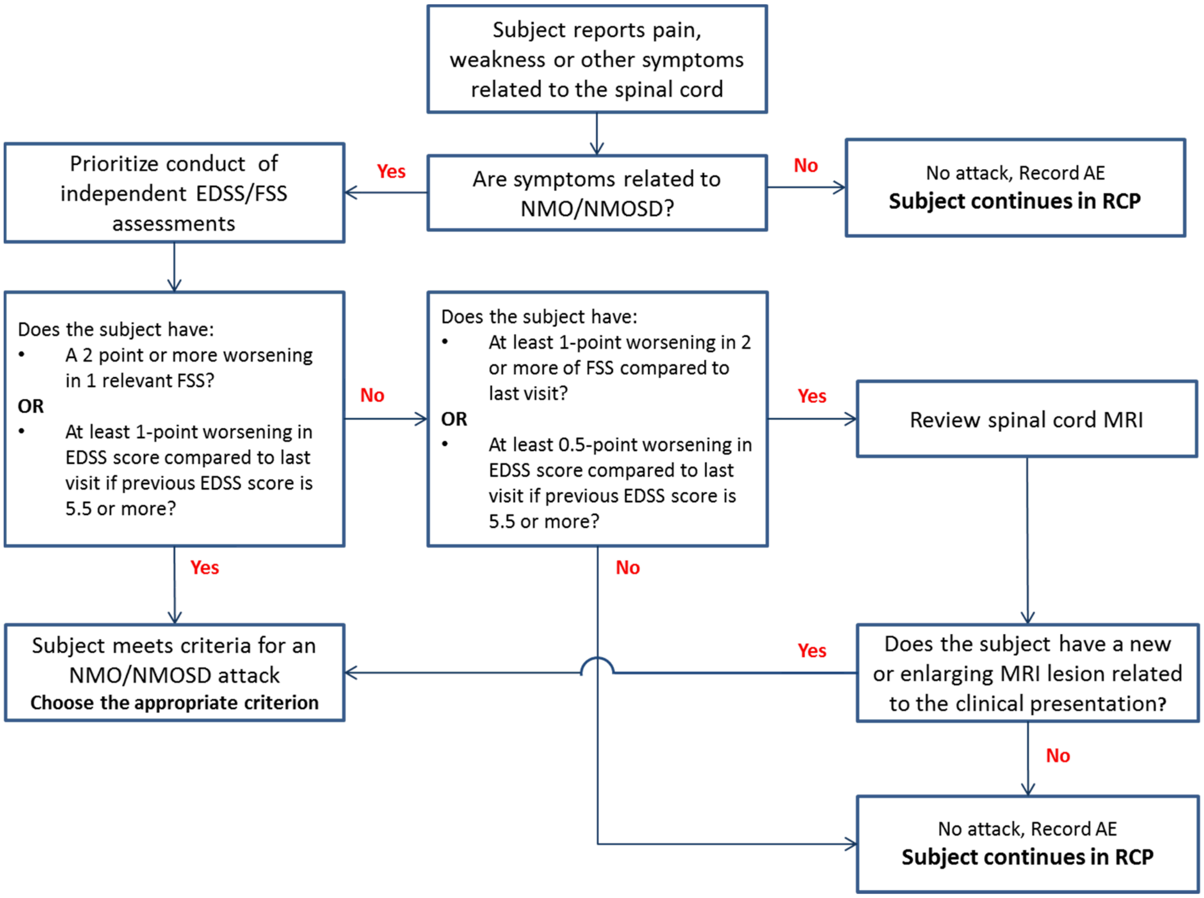

AE = adverse event; EDSS = Expanded Disability Severity Score; FSS = Functional Systems Scores; MRI = magnetic resonance imaging; NMO/NMOSD = neuromyelitis optica/neuromyelitis optica spectrum disorders.

Figure 6 Process for Assessing Symptoms Affecting the Brain or Brainstem

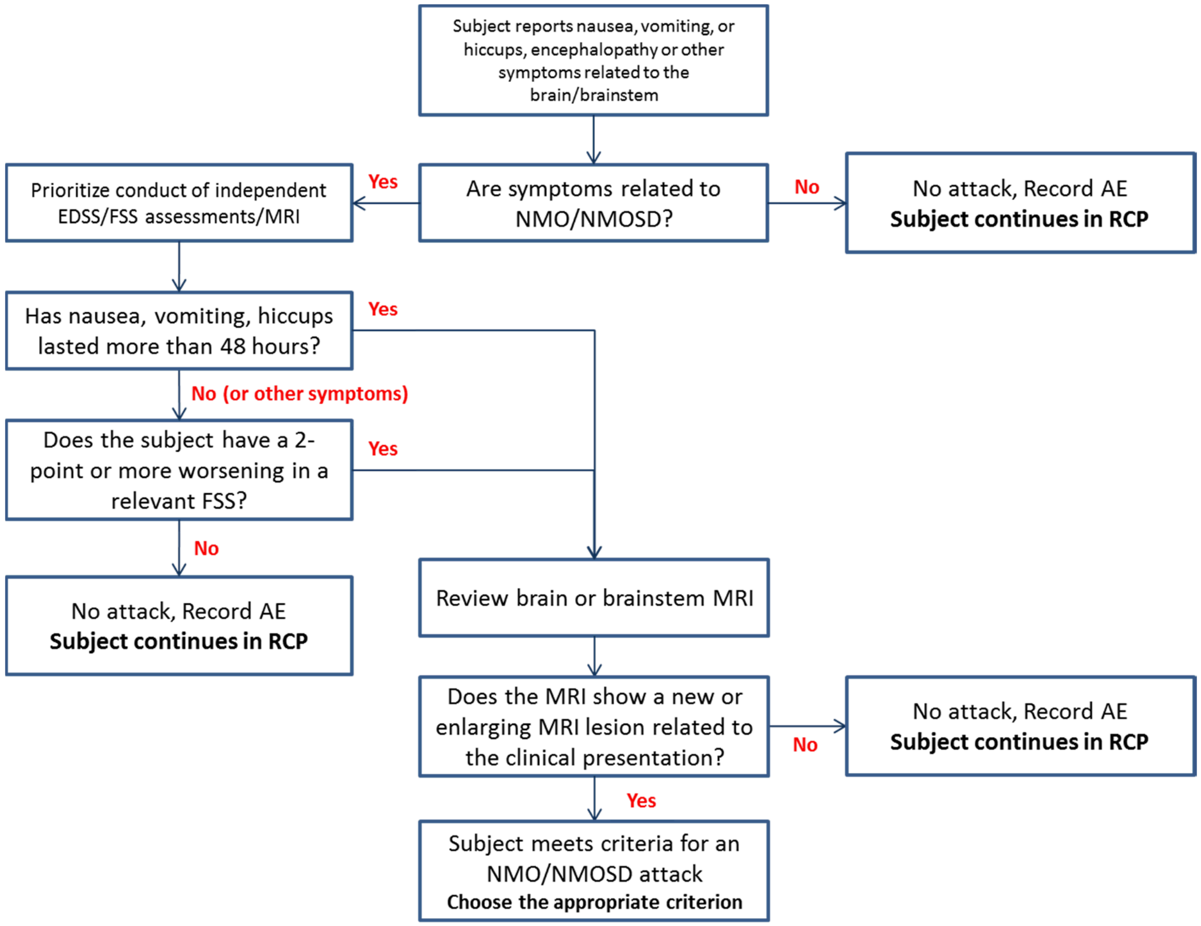

AE = adverse event; EDSS = Expanded Disability Severity Score; FSS = Functional Systems Scores; MRI = magnetic resonance imaging; NMO/NMOSD = neuromyelitis optica/neuromyelitis optica spectrum disorders; RCP = Randomized-controlled Period.

Every effort should be made to initiate rescue therapy only after full assessment is conducted and the NMO/NMOSD attack has been diagnosed in accordance with the protocol-defined criteria. However, the investigators can at any time decide to initiate rescue therapy if, in their clinical judgment, the subject will be at unacceptable risk if such therapy is not immediately initiated.

Adjudication Process (For Attacks Occurring During the RCP)

The entire data generated during the Assessment Visit of the potential attack will be provided to the AC upon completion of the assessment. The adjudication process will be completed when the AC makes their final decision, which will occur in a total of 14 days (+ 3 days) from the start of the Assessment Visit.

The data set that will be delivered to the AC will be identical to the data set generated and used by the Principal Investigator to make the diagnosis of NMO/NMOSD attack and will include the following:

- Description of the new or worsening symptom
- Findings of physical and neurological examination
- Relevant laboratory test results
- Relevant x-ray studies, if performed in relation to the assessment
- Expanded Disability Status Scale score as determined by the independent assessor
- Ophthalmology examination results done by an independent ophthalmologist
- Magnetic resonance imaging scans if done as required by certain protocol attack criteria
- Short narrative written by the Principal Investigator to summarize the assessment WITHOUT disclosing if an NMO/NMOSD attack was diagnosed (narrative template will be provided by the Sponsor)

The data set will be provided to the independent AC by the vendor according to the Adjudication Committee Charter (see Section 4.3.1.5).

The AC will not be provided with the Principal Investigator's opinion of whether an NMO/NMOSD attack has occurred, nor will the AC receive information about which protocol attack criterion was met or whether a rescue medication was provided.

The decision by the AC will be communicated to the Principal Investigator at the clinical site. At this point the following scenarios will be possible:

1 Subjects for whom the NMO/NMOSD attack was determined by the AC will have the option to enter the OLP. If the subject decides not to enter the OLP, the subject will then enter the SFP.
2 Subjects for whom the NMO/NMOSD attack was not determined by the AC will continue in the RCP.

In addition, the dataset that was generated and used by the Principal Investigator to assess the symptom(s) that were determined not to be related to NMO/NMOSD will be delivered to the AC for review and will include the following:

- Description of the new or worsening symptom
- Findings of physical and neurological examinations
- Relevant laboratory test results
- Relevant x-ray studies, if performed in relation to the assessment
- Any other work-up that was performed to assess the symptom(s)

Adjudication Process (For Attacks Occurring During the OLP)

The data generated during the Assessment Visit and the full assessment of the potential attack will be provided to the AC and follow the same process as described above for the RCP.

The data to be adjudicated will be provided to the independent AC by the vendor according to the Adjudication Committee Charter (see Section 4.3.1.5).

Regardless of the decision made by the AC, the subject may choose to continue in the OLP or withdraw from the OLP and enter the SFP.

4.3.1.2 Neuroaxis Magnetic Resonance Imaging Scan

MRI for All Subjects

All subjects will have a full neuroaxis MRI scan, including optic nerve, spinal cord, and brain performed at screening to determine eligibility and establish a baseline (see Section 4.2.1). In the event a subject is re-screened a full neuroaxis MRI need not be repeated if the previous study neuroaxis MRI was conducted within the 3 months prior to the rescreening visit. A neuroaxis MRI scan will be repeated at 28 weeks in subjects who complete the RCP without having experienced an NMO/NMOSD attack. Subjects who discontinue the RCP due to the occurrence of the 67th AC-determined attack who have had an MRI in the last 3 months need NOT repeat the MRI. During the OLP, neuroaxis MRI scans will be performed every 52 weeks. All MRI scans will be centrally read and data collected for analysis at the end of the study.

The Principal Investigator at the site should not use these MRIs for any clinical decision making, but review of the brain MRI is acceptable if there is a safety alert from the central MRI reader indicating the presence of a new lesion(s).

MRI for NMO/NMOSD Attacks

In addition to the scheduled MRIs, at the time of any Assessment Visit, a full neuroaxis MRI scan will be performed of all domains. These scans will be sent for a central reading and data will be collected for analysis at the end of the study. MRI images/study report obtained during an Assessment Visit should not be reviewed by the Principal Investigator unless a specific criterion for an attack requires review of the MRI (Table 11). In such cases review of the MRI image and local report MUST be done AFTER the review of all relevant clinical assessment data is complete.

The Principal Investigator at the site should not use these MRIs for any clinical decision making. Additionally, the Principal Investigator should NOT use the MRI to make any decisions regarding the potential severity of an NMO/NMOSD attack; rather it should only be used to confirm the clinical presentation. The MRI scan will also be read centrally, and the

central report and image will be used by the AC for the adjudication of the NMO/NMOSD attack.

Neuroaxis MRI Documentation and Assessment

All MRI scans are to be performed at the study site. To ensure that MRI scans are of acceptable quality for viewing the optic nerve, spinal cord, and brain, the settings and procedures (specified by the central reader) to be used for the MRI scanners are mandated at a minimum standard and defined in the MRI Imaging Manual (provided separately). The site will be required to perform a qualification MRI scan to send to the central reading site to ensure compliance with the requirements for the study. Qualification MRI scans may require consenting a volunteer subject from the investigative site, depending on local Institutional Review Board/Ethics Committee (IRB/EC) requirements.

All MRI images from the site will be date and time stamped and sent to the central imaging vendor that is independent of the study sites. The images will be read by 2 independent neuro-radiologists at the central reading site for a consensus read and central reporting. The MRI reports and scans produced as part of the protocol-defined criteria for an NMO/NMOSD attack will be sent from the central reading site to the AC as part of the assessment data to be adjudicated.

4.3.1.3 Independent Ophthalmology Assessments

The protocol-defined ophthalmology assessment will consist of a high- and low-contrast visual acuity test, and assessment for relative afferent pupillary defect (RAPD).

All examinations must be conducted by an ophthalmologist or another site member with appropriate training and experience who is independent of the investigator and the subject. If possible, the same person should review a single subject through every ophthalmology assessment. The data generated from the ophthalmology assessments will be entered directly into an electronic data capture device provided to the site with a date and time stamp. In order to minimize bias, the ophthalmology examiner will not have access to prior visual acuity scores or RAPD findings at the time of a new evaluation. The ophthalmology examiner will be instructed NOT to elicit or discuss the subjects' experiences or AEs on the study.

Ophthalmology assessments will be completed at screening, Day 1, at scheduled study visits, and Assessment Visits, according to the schedule of procedures in Table 5, Table 6, Table 7, Table 8, and Table 9.

In the event that a subject has new or worsening symptoms related to NMO/NMOSD, all ophthalmology assessments will also be performed at an Assessment Visit.

The ophthalmology examiner will provide a full report of the examination findings, in accordance with protocol-defined parameters, to the investigator to review against a subject's

previous reports and to determine if the findings meet 1 or more of the protocol-defined criteria for ON attack.

Visual Acuity Assessments

For the purposes of this study, visual acuity will be measured using Landolt C Broken Ring Chart (both high and low contrast). Best-corrected monocular and binocular vision will be tested.

Visual acuity tests are used to determine the smallest characters that can be read on a standardized chart. The Landolt C Broken Ring Chart has a standardized format designed to be equally detectable for normal observers consisting of an incomplete ring resembling the letter “C.” The width of the break and the ring are each one-fifth of its overall diameter. The subject must indicate where the break is located, the break being positioned in any direction. Each line has 5 rings and the spacing between the rings and the lines is proportional to the ring size. The change in visual acuity from one line to the next occurs in equal logarithmic steps.

To standardize the method of assessment, the charts must be used in a retro-illuminated Early Treatment Diabetic Retinopathy Study (ETDRS) cabinet to eliminate the need for standardization of height and room lighting.

Charts and wheeled light cabinets will be supplied to all sites.

To ensure consistency of visual acuity testing across subjects and study sites, the following set-up and procedures are required:

<u>Low-contrast Visual Acuity</u>

- Charts must be used with a retro-illuminated ETDRS cabinet
- Use a well-lit room, but one not exposed to full sunlight to not distract from the subject’s normal eyesight
- Use overhead lights to provide an even distribution of light across the room
- Prevent distractions and limit movement in the room
- Position the subject at a distance of **3 meters** from the chart
- Test best-corrected monocular and binocular vision
- Ask the subject to describe the top line of the chart from left to right. Record the number of rings that the subject successfully describes as the subject describes them. If subject cannot easily read the rings, ask the subject to guess.
- Ask the subject to continue reading each subsequent line below the top line until the subject reaches a line where all the rings cannot be made out. Record the number of rings that the subject successfully describes as the subject reads them
- Compare the subject’s results against the actual rings on each line of the chart
- The last line the subject is able to see and read correctly determines the score

High-contrast Visual Acuity

- Use a well-lit room, but one not exposed to full sunlight to not distract from the subject's normal eyesight
- Use overhead lights to provide an even distribution of light across the room
- Prevent distractions and limit movement in the room
- Position the subject at a distance of **3 meters** from the chart
- Test best-corrected monocular and binocular vision
- Ask the subject to describe the top line of the chart from left to right. Record the number of rings that the subject successfully describes as the subject views them. If subject cannot easily read the rings, ask the subject to guess.
- Ask the subject to continue describing each subsequent line below the top line until the subject reaches a line where all the rings cannot be made out. Record the number of rings that the subject successfully describes as the subject reads them
- Compare the subject's results against the actual rings on each line of the chart
- The last line the subject is able to see and read correctly determines the score

The following visual acuity data are to be reported by the ophthalmology examiner:

- Low-contrast visual acuity score for each eye separately and both eyes together
- High-contrast visual acuity score for each eye separately and both eyes together
- If a subject cannot read any lines on the chart, visual acuity will be tested by counting fingers (CF), hand movement (HM), or light perception (LP) at 1 meter, or no light perception (NLP)/total blindness.

Assessment of Relative Afferent Pupillary Defect

For the purposes of this study, RAPD will be assessed by the "swinging light test," a method of detecting differences between the two eyes in how the pupils respond to light shone in one eye at a time. This test must be administered by an ophthalmologist or another site member with appropriate training and experience (ie, not the investigator) and in accordance with standard of care at the institution. If possible, the same ophthalmology examiner should review a single subject through every assessment.

The RAPD test can be useful in detecting unilateral or asymmetric disease of the retina or optic nerve. The physiological basis of the test is that in healthy eyes the reaction of the pupils in the left and right eyes are linked; that is, a bright light shone in one eye will lead to equal constriction of both pupils. A RAPD is present if the initial consensual pupillary constriction is greater than the initial direct pupillary constriction.

The following procedures are recommended:

- Perform the test in a semi-darkened room. If the room is too dark it will be difficult to observe pupil responses, particularly in heavy pigmented eyes
- Use a bright torch such as a small flashlight that can be focused to provide a narrow, even beam of light
- Ask the subject to focus on a distant object and keep looking at it – use a chart or picture as an object of focus
- Move the flashlight from side to side so the beam of light shines directly into each eye. Do not swing the beam from side to side around a central axis (eg, by holding it in front of the subject's nose) as this can stimulate the near response
- Keep the light source at the same distance from each eye to ensure the light stimulus is equally bright in both
- Keep the beam of light steadily on the first eye for at least 3 seconds to allow the pupil size to stabilize. Note whether the pupil of the eye being illuminated reacts briskly and constricts fully to the light. Also note the response in the other eye – does it also constrict briskly?
- Move the light quickly to shine in the opposite eye. Hold the light steady for at least 3 seconds. Note whether the pupil of the eye being illuminated stays the same size, or whether it gets bigger. Note the response in the opposite eye.
- Repeat the test, observing the response of the pupils of both eyes when one and then the other is illuminated.

The RAPD results will be reported as follows:

- Date and time of examination
- Presence or absence or loss of RAPD in either eye
- If RAPD is present, record pupillary responses on a scale of 1+ to 5+ for each eye according to the criteria in Table 12.

Table 12 Pupil Response Scoring Criteria for Relative Afferent Pupillary Defect

Score	Definition
1 +	Weak initial constriction and greater re-dilatation
2 +	Initial stall and greater re-dilatation
3 +	Immediate pupillary dilatation
4 +	Immediate pupillary dilatation following prolonged illumination of the good eye for 6 seconds
5 +	Immediate pupillary dilatation with no secondary constriction

Source: Bell et al, 1993.

On receipt of the full examination results from the ophthalmology examiner, the investigator must compare the results against the last assessment.

4.3.1.4 Expanded Disability Status Scale and Functional Systems Scores

The EDSS and its associated FSS provide a system for quantifying disability and monitoring changes in the level of disability over time. The EDSS scores and FSS will be captured and date and time stamped using electronic data capture. Standardized guidance for the assessment of the EDSS and FSS has been developed, including a set of definitions for the EDSS and FSS. The latest version of the EDSS/FSS is version 04/10.2, which will be used in this study (Kappos et al, 2015; Kurtzke, 1983; D'Souza et al, 2016).

The EDSS ranges from 0 (normal neurological exam) to 10 (death from MS), with half steps from 1.0 to 9.5. Increasing values represent a higher grade of impairment and disability. The EDSS steps 1.0 to 5.0 refer to patients with MS who are able to walk without any aid and are defined more by the results of the 7 Functional Systems (FS; Visual FS, Brainstem FS, Pyramidal FS, Cerebellar FS, Sensory FS, Bowel and Bladder FS, and Cerebral FS). Each FS is structured in grades from 0 to 5 or 6 (only the Visual FS has a maximal grade 4 after conversion). The EDSS steps ≥ 5.5 are exclusively defined by the ability to ambulate, the assistance required, or the use of a wheelchair. The EDSS and FSS are provided in Appendix 4.

The EDSS assessment will be completed at screening, Day 1 of the RCP, at scheduled study visits, and Assessment Visits, according to the schedule of procedures in Table 5, Table 6, Table 7, Table 8, and Table 9. The assessment must be conducted by a trained and certified neurologist (so called EDSS examiner) who is independent of the treating physician (so called investigator) and the subject. A back-up EDSS examiner must be available so that EDSS assessments can be made at the time of an NMO/NMOSD attack. The same EDSS examiner should perform the assessment for individual subjects for the duration of the study to ensure consistency with assessment, if possible. MedImmune will provide training to all EDSS examining investigators and collect documented evidence of training prior to allowing administration of the EDSS for the purposes of the study. The data generated from the examination will be captured electronically. In order to minimize bias, the EDSS examiner will not have access to prior EDSS/FSS at the time of a new evaluation. The EDSS examiner will be instructed NOT to elicit or discuss the subject's experiences or AEs on the study.

4.3.1.5 Independent Adjudication Committee

Three experts in NMO/NMOSD disease will constitute the independent AC. This committee has the mandate to evaluate each data set that is generated during the Assessment Visit and determine if an NMO/NMOSD attack occurred based on the clinical presentation, assessments, and the protocol-defined criteria for an NMO/NMOSD attack. The committee is completely independent and blinded to treatment assignments and/or to pertinent treatment

unmasking information (eg, B-cell counts, AQP4-IgG titers post-randomization) of study subjects, and to any decision made by the Principal Investigator regarding an NMO/NMOSD attack. The committee will use its judgement and clinical experience to make its determination based on the data provided. The committee will review the same data that are available to the Principal Investigator when he/she makes his/her determination about the attack. In exceptional cases, determined and justified by the committee as such, the committee can request to review additional data that is essential to make a determination. The committee's role, function and procedures are governed by a separate charter.

The data from all subjects who are assessed at an Assessment Visit due to a new symptom(s) or worsening of an existing symptom(s) related to NMO/NMOSD will be sent for adjudication by the AC regardless of whether the Principal Investigator has determined that an NMO/NMOSD attack has occurred. Furthermore, the AC will not receive information about which protocol attack criterion was met and the rescue medication utilized.

Determination of an NMO/NMOSD attack by the AC is for the purpose of determining which events will be included in the primary analysis. It will NOT affect the rescue therapy that a subject may receive.

Only subjects in whom the attack was determined by the AC will be given the option to enter the OLP.

In addition, the data set that was generated and used by the Principal Investigator to assess the symptom(s) that were determined not to be related to NMO/NMOSD in the RCP, will be delivered to the AC for a non-real-time review.

4.3.1.6 Healthcare Resource Utilization

The Healthcare Resource Utilization (HCRU) information is collected to quantify the impact of disease and treatment on the subject's scheduled and medical facility visits. The HCRU information related to NMO/NMOSD will be elicited using a subject diary.

At each scheduled study visit, all subjects will be provided with a paper diary and asked to complete these for the duration of their participation in the study. At each visit, the diary will be returned to the site and the data will be reviewed by the investigator. The diary will constitute source data and will be retained by the site.

In-patient hospitalization, for the purposes of analysis of the secondary endpoint, is defined as a stay in a hospital that goes beyond midnight of the first day of admission. Any duration of stay (calculated as the difference between discharge date and admission date) of more than 1 day will contribute to this endpoint. This includes the time a subject may enter an emergency department plus subsequent admission to a ward. Hospitalizations for the administration of NMO-related medications or procedures only (ie, the administration or procedure was isolated

and not provided during a subject's hospitalization due to NMO for another reason) will not be included in the secondary endpoint analysis.

Information will be collected throughout the RCP and the OLP on all NMO/NMOSD-related HCRU visits as presented in Table 13.

Table 13 Healthcare Resource Utilization Information to be Collected and Frequency

Parameter	Frequency
Ambulance transport	Total number of times since the last visit
Visits to primary healthcare physician	Total number of times since the last visit
Emergency Room/Department visits	Total number of times since the last visit
Hospitalization (general care)	Total number of days since the last visit
Hospitalization (intensive care)	Total number of days since the last visit
Other healthcare visits (eg, physiotherapist)	Total number of times since the last visit
Home visit from a physician	Total number of times since the last visit
Home visit from a nurse	Total number of times since the last visit

Subjects will also be asked to record any medical procedures they undergo for their NMO/NMOSD (outside of scheduled study procedures) in the diary.

4.3.2 Patient-reported Outcomes

Patient-reported outcome (PRO) is an umbrella term referring to all outcomes and symptoms that are directly reported by a subject. Patient-reported outcomes, including HRQoL, have become significant endpoints when evaluating effectiveness of treatments in clinical trials. Five separate pain NRS and the Short Form 36 Health Survey version 2 (SF-36v2) have been selected for this study and will be administered at time points indicated in Table 6 and Table 8.

4.3.2.1 Pain NRS

Eleven-point pain NRS will be used to capture a subject's worst pain levels experienced in the eyes, upper back, lower back, arms, and legs, where 0 = no pain and 10 = worst pain imaginable. The subject will be asked to rate the pain he/she has experienced in each of the 5 locations over the past 24 hours.

The pain NRS will be completed according to the schedule of procedures in Table 5, Table 6, Table 7, Table 8, and Table 9.

4.3.2.2 Short Form-36 Health Survey

The SF-36v2 is a validated, generic health survey that captures information about functional health and well-being from the patient's perspective. The SF-36v2 is for adults aged 18 years and older and it can be used across all diseases. The SF-36v2 consists of 36 items and measures eight domains (see Appendix 6). These are Physical Functioning, Role Physical, Bodily Pain, General Health, Vitality, Social Functioning, Role Emotional, and Mental Health. The standard recall period of the SF-36v2 is 4 weeks with response options for each of the 36 items ranging from 1-3 (10 items), or 1-5 (26 items). The SF-36v2 is a self-completed paper-based survey, available in multiple modes of administration and can be completed in 5 to 10 minutes. Translations are available for multiple countries and languages.

As well as a profile of the eight domain scores, the SF-36v2 provides psychometrically-based PCS and MCS scores. The scores range from 0-100, with a higher score indicting better health and well-being. The scores are calibrated so that 50 is the average score or norm with a standard deviation of 10.

The SF-36v2 with a 4-week recall period will be used at 3-monthly intervals throughout the study to evaluate HRQoL for the duration of the subject's participation in the study.

If a subject has an Assessment Visit, the subject will be asked to complete the SF-36 acute version (1-week recall) to capture the impact of the attack on HRQoL. These data will be used primarily for health economic purposes with the generation of an attack utility profile.

4.3.2.3 Collection of PRO Endpoints

Standard procedures for minimizing bias and enhancing PRO compliance will be followed throughout the study. Site assessments of PROs should be made prior to any other site activities and encounters with physician, with exception of an Assessment Visit wherein procedures to identify an NMO/NMOSD attack will take precedence. The subjects will be instructed to complete the PROs independently. The site will have a designated quiet space for subjects to use when completing the assessments. Dedicated investigational staff at the site will be responsible for ensuring that the PRO administration will be followed according to the specific instructions from the clinical study team. The site staff will be required to monitor that the subjects have completed the appropriate PRO instruments in their entirety during the site visits.

4.3.3 Medical History and Physical Examination, Electrocardiogram, Weight, Vital Signs, and Chest X-ray

4.3.3.1 Medical History

A complete medical history will be completed during screening. The subject's full NMO/NMOSD disease history will also be captured, including date of original diagnosis of NMO/NMOSD, historical AQP4-IgG status, relapse dates and type of relapse (eg, ON,

myelitis, brain and brainstem), and treatment for the relapse. Information related to other autoimmune diseases (eg, rheumatoid arthritis, Hashimoto's thyroiditis, systemic lupus erythematosus, Sjogren's syndrome, diabetes, myasthenia gravis, and pernicious anemia) will also be captured.

On Day 1 of the RCP, the medical and disease history will be reviewed and any changes since screening will be documented, if applicable.

4.3.3.2 Physical and Neurological Examination

Complete physical and neurological examinations will be performed at intervals designated in the schedules of study procedures (see Table 5, Table 6, Table 7, Table 8, and Table 9) and include the following examinations: head, eyes, ears, nose and throat, lungs heart, abdomen, joints, muscles and soft tissues, nervous system, skin, lymph nodes, mental status, cranial nerves, nystagmus, motor system - muscle strength, sensory system - sensation, bowel and bladder function, deep tendon reflexes, gait, station, coordination, and other. Additional neurological exams may be conducted according to local practice.

In the event that a new abnormality is identified, the investigator should perform further investigations according to clinical judgment.

Any abnormalities should be reported as AEs or signs/symptoms of NMO as applicable.

4.3.3.3 Body Weight and Height

Height will be measured at screening. Body weight will be measured at screening and at Days 1, 85, and 197 of the RCP.

Any abnormalities should be reported as AEs or signs/symptoms of NMO/NMOSD as applicable.

4.3.3.4 Electrocardiogram

Electrocardiogram (ECG) recordings will be evaluated at screening, Day 1, Day 197, and at the Assessment Visit as presented in Table 5, Table 6, and Table 8. These will be performed as per local investigative site procedures. The principal investigator or qualified designee will review and indicate if the ECG is normal, abnormal (clinically significant), or abnormal (not clinically significant). Any medically significant changes from the screening ECG will be recorded as an AE or SAE. The QTc interval measurements will be recorded for all subjects.

Any abnormalities should be reported as AEs or signs/symptoms of NMO as applicable.

4.3.3.5 Vital Signs

Vital signs (body temperature, blood pressure [BP], pulse rate, and respiratory rate) will be evaluated at screening, and at various time points as outlined in Table 5, Table 6, Table 7, Table 8, Table 9, and Table 10.

Vital signs should be within 60 minutes prior to investigational product administration, every 15 minutes (± 5 minutes) during the infusion, immediately (+ 5 minutes) after the end of the infusion, and then every 30 minutes (± 5 minutes) for 2 hours after dosing or until stable, whichever is later.

Any abnormalities should be reported as AEs or signs/symptoms of NMO/NMOSD as applicable.

It is important that body temperature is assessed in order to evaluate the subjects of any signs of infection or infusion reaction, especially when assessing for potential attacks.

4.3.3.6 Chest X-ray

A screening chest x-ray (anterior/posterior and lateral) will be mandatory if, in the opinion of the investigator, the subject has a previous history of or a suspected pulmonary infection or disease. The chest x-ray will be performed during the screening period. The chest x-ray may be substituted with documentation of a previous chest x-ray performed within the previous 3 months that meets the inclusion criteria. A previous computerized tomography evaluation of the lungs may be substituted for the chest x-ray if performed within the last 3 months.

4.3.4 Columbia-Suicide Severity Rating Scale

In 2012, the United States Food and Drug Administration/Center for Drug Evaluation (US FDA/CDER) mandated that prospective suicidal ideation and behavioral assessments be conducted in clinical trials using investigational products that may be used for or appear to have an effect on the CNS.

The Center for Suicide Risk Assessment and Columbia University Department of Psychiatry developed the Columbia-Suicide Severity Rating Scale (C-SSRS) to assess suicide risk. The C-SSRS is unique in that it assesses both suicidal behavior and predicts future attempts. The C-SSRS is used extensively across primary care, clinical practice, surveillance, research, and institutional settings.

The C-SSRS involves a series of probing questions that inquire about possible suicidal thinking and behavior, and classifies these events of interest into 11 categories of interest as part of the assessment process. The terminology considered important includes 5 levels of suicidal ideation, 5 levels of suicidal behavior, and the category "self-injurious behavior, no suicidal intent." A different version of the C-SSRS is administered at different visits during the study. The Sponsor will be responsible for ensuring the relevant site staff are appropriately

trained in the C-SSRS. Documented evidence of training of the sites will be required prior to C-SSRS administration to subjects in the study.

The “Baseline/Screening” questionnaire will be administered at screening (Appendix 7). This version of the scale combines the “Baseline” and “Screening” versions to assess suicidality in a patient’s lifetime and over the 6 months prior to screening. This version can assess a subject’s lifetime suicidality for data collection purposes. Although the C-SSRS is a detailed interview, the full questionnaire is needed only if the initial screening questions regarding suicidal ideation and behavior are positive. Typically, the assessment takes approximately 1 to 2 minutes for those who have no positive findings and no more than 10 minutes for subjects who may have a number of positive findings requiring further probing questions.

The “Since Last Visit” version of the C-SSRS will be administered at all scheduled visits and Assessment Visits during the RCP, all visits during the OLP, and every visit during the SFP for all subjects randomized into the study. The “Since Last Visit” version assesses suicidality since the subject’s last visit. This version is used to assess subjects who have completed at least one initial C-SSRS assessment and should be used during the RCP and the OLP. The “Since Last Visit” version assesses any suicidal thoughts or behaviors that the subject may have had since the last time they were administered the C-SSRS (Appendix 8).

In the event that a subject is considered to be at risk based on the outcome of the C-SSRS, the investigator should treat the subject according to their clinical judgment and report the AE according to AE/SAE definitions and reporting requirements outlined in this protocol (see Section 5.1, Section 5.2, and Section 5.4.

4.3.5 Modified Rankin Scale

The modified Rankin Scale (mRS) is a commonly used scale for measuring the degree of disability or dependence in the daily activities of people who have suffered a stroke or other causes of neurological disability (Farrell et al, 1991; van Swieten et al, 1988).

The mRS ranges from 0 to 6, ranging from perfect health without symptoms to death as follows:

- 0 - No symptoms
- 1 - No significant disability. Able to carry out all usual activities, despite some symptoms
- 2 - Slight disability. Able to look after own affairs without assistance, but unable to carry out all previous activities
- 3 - Moderate disability. Requires some help, but able to walk unassisted
- 4 - Moderately severe disability. Unable to attend to own bodily needs without assistance, and unable to walk unassisted
- 5 - Severe disability. Requires constant nursing care and attention, bedridden, incontinent

- 6 - Dead

4.3.6 Clinical Laboratory Tests

A Laboratory Manual will be provided to the sites that specifies the procedures for collection, processing, storage, and shipment of samples, as well as laboratory contact information, specific to this clinical research study.

Clinical laboratory safety tests, including serum pregnancy tests, will be performed in a central clinical laboratory. Urine pregnancy tests may be performed at the site using a licensed test (dipstick); TB test will be performed at the site. Abnormal laboratory results should be repeated as soon as possible (preferably within 24 to 48 hours). Blood samples should be collected with subject in a fasting state. In addition, abnormal laboratory results may be graded according to National Cancer Institute (NCI) Common Terminology Criteria for Adverse Events V4.0 (NCI, 2009).

The following clinical laboratory tests will be performed (see Table 5, Table 6, Table 7, Table 8, and Table 10 for the schedule of tests):

Serum Chemistry

- Calcium
- Chloride
- Magnesium
- Potassium
- Sodium
- Bicarbonate
- Aspartate transaminase (AST)
- Alanine transaminase (ALT)
- Alkaline phosphatase (ALP)
- Total bilirubin
- Gamma glutamyl transferase (GGT)
- Lactic dehydrogenase (LDH)
- Uric acid
- Creatinine
- Blood urea nitrogen (BUN)
- Glucose
- Albumin
- Total protein
- Triglycerides
- Cholesterol

Note for serum chemistries: Tests for AST, ALT, ALP, and total bilirubin must be conducted concurrently and assessed concurrently.

Hematology

- White blood cell (WBC) count with differential
- Red blood cell (RBC) count
- Hematocrit
- Hemoglobin
- Coagulation: prothrombin time (PT), activated partial thromboplastin time (aPTT), international normalized ratio (INR)
- Platelet count
- Mean corpuscular volume (MCV)
- Mean corpuscular hemoglobin concentration (MCHC)
- Hemoglobin A1c (subjects with diabetes only)

Urinalysis

- Color
- Appearance
- Specific gravity
- pH
- Protein
- Glucose
- Ketones
- Blood
- Bilirubin
- Microscopy including WBC/high power field (HPF), RBC/HPF

Pregnancy Test (females of childbearing potential only)

- Urine human chorionic gonadotropin (HCG)
- Serum beta-HCG (at screening only)

Other Safety Tests

- Hepatitis B surface antigen, hepatitis B core antibody (anti-HBc), hepatitis B surface antibody, hepatitis C antibody
- Tuberculosis (TB) test (eg, QuantiFERON-TB Gold test) if applicable, as per local standard of care guidelines, at screening only)
- Human immunodeficiency virus (HIV)-1,-2 antibody (screening only)
- Polyomavirus JCV antibody titers (serum to be collected at screening only and frozen for future testing, if required).
- In a case of suspected PML, a CSF sample for JCV DNA polymerase chain reaction (PCR) will be collected and frozen immediately.
- Tetanus vaccination titers
- Total Ig, IgM, IgG, IgA, IgE

Other Tests

- Serum autoantibodies

Estimated Glomerular Filtration Rate

The estimated GFR must be ≥ 60 mL/minute for study eligibility to minimize the risk of developing nephrogenic systemic fibrosis upon receiving Gd-containing contrast agents. The estimated GFR will be calculated by the central laboratory based on the plasma and urine creatinine samples provided using the Modification of Diet in Renal Disease formula. Glomerular filtration rate eligibility must be confirmed by the site on receipt of the central laboratory report and prior to randomization.

Vaccination Titers

Serum samples for anti-tetanus vaccine antibody titers will be collected from all subjects according to the schedules of procedures in Table 6 and Table 7. The assay will be conducted by the central laboratory and further details will be provided in the Laboratory Manual.

Because MEDI-551 may reduce vaccine titers, the levels reported for this study by the central laboratory will not be made available to the sites or to the sponsor at any point during the study prior to unmasking of the study.

4.3.7 Pharmacokinetic Evaluation and Methods

Samples for MEDI-551 serum concentration will be collected according to the schedules of procedures in Table 6.

Serum samples will be measured for MEDI-551 levels by MedImmune using a validated immunoassay. Detailed procedures for sample collection, processing, storage, and shipment are presented in the Laboratory Manual provided to each study site.

4.3.8 Immunogenicity Evaluation and Methods

Serum samples for ADA will be collected from all subjects according to the schedules of procedures in Table 6, Table 7, Table 8, and Table 10.

Samples will be measured for the presence of ADA by MedImmune using a validated, drug-tolerant solution phase bridging assay. Tiered analyses will be performed to include screening, confirmatory, and titer assay components and positive-negative cut points will be employed that were statistically determined from drug naive validation. Evaluations will be performed at baseline prior to administration of investigational product and at routine time points during the treatment and follow-up phase of the study or at discontinuation.

4.3.9 Biomarker Evaluations and Methods

Samples for measurement of biomarkers listed below will be collected according to the schedules of procedures in Table 6, Table 7, and Table 8. Detailed procedures for sample collection, processing, storage, and shipment are presented in the Laboratory Manual provided to the study site

Serum for AQP4-IgG Serostatus Assay

The AQP4-IgG serostatus assay will be used to determine AQP4-IgG serostatus for stratification purposes. The assay will be conducted by a central laboratory using a commercially available visual fluorescence-observation cell binding assay (EUROIMMUN) that has been validated to Clinical Laboratory Improvement Amendment/College of American Pathologists standards. The method involves detection of IgG binding to cells expressing recombinant AQP4. The serostatus of each subject will be provided to the site prior to randomization.

The screening, baseline, and study visit assays used to determine AQP4-IgG titer will be conducted by a central laboratory using a quantitative flow cytometry assay based on binding of IgG to cells transfected with AQP4. Serum will be collected for the measurement of serum

AQP4-IgG by flow cytometry at visits specified in Table 5, Table 6, Table 7, Table 8, and Table 9.

Because MEDI-551 is hypothesized to reduce AQP4-IgG titers the AQP4-IgG titer levels reported for this study by the central laboratory will not be made available to the sites or the sponsor at any point during the study prior to unmasking of the study.

Whole Blood for Flow Cytometry

Whole blood samples for quantification of relevant immune cells will be collected according to the schedules of procedures in Table 5, Table 6, Table 7, Table 8, Table 9, and Table 10. Flow cytometry performed at a central laboratory will be used to enumerate B-cell, T-cell, and natural killer (NK)-cell levels and to conduct immunophenotyping analyses of various B-cell subsets.

MEDI-551 is known to deplete CD19+ B cells. Therefore, in this study, the results of flow cytometry for B-cell counts could potentially be unmasking. For this reason, these data will not be made available to the sites or the sponsor from post-randomization through the remainder of the study prior to unmasking of the study.

Whole Blood for Gene Expression

Whole blood samples will be used to isolate messenger ribonucleic acid (mRNA) and assess the effect of MEDI-551 on a specific plasma cell gene signature. In addition, the effects of MEDI-551 on the expression of mRNA in pathways thought to be involved in NMO or related to treatment response will be explored. These analyses will be performed using Affymetrix whole genome expression array and/or TaqMan®-based assays on selected gene panels. Whole Blood will be collected at visits specified in Table 6, Table 7, Table 8, and Table 10. Specific procedures for sample collection, processing, storage, and shipment can be found in a separate laboratory manual provided to the sites. MEDI-551 is known to reduce the plasma cell gene signature. Because the results of the the plasma cell gene signature could potentially be unmasking, these samples will not be tested prior to unmasking of the study.

4.3.9.1 Exploratory Investigations

Serum for Exploratory Biomarker Studies

Serum will be collected for the measurement of exploratory biomarkers at visits specified in Table 6, Table 7 and Table 8. The effect of MEDI-551 on relevant protein biomarkers will be explored. These studies may include immunoglobulins, specific autoantibodies or autoantibody arrays, inflammatory proteins (eg, cytokines and chemokines), or other biomarkers and cell-based bioassays that are related to disease mechanisms or the mechanism of action of MEDI-551. The presence of autoantibodies to antigens other than AQP4 may be explored throughout the study.

Whole Blood for DNA Analysis (Optional)

To investigate genetic characteristics that may be associated with subjects' clinical responses to MEDI-551, the following may be evaluated: polymorphisms in B-cell related genes, NK cell-related genes, Fc receptor genes, and changes in antibody or T-cell receptor gene sequences.

The collection of blood for DNA analysis is optional. The completion of a separate ICF (Informed Consent Form for Collection of Blood Samples for DNA Analysis) must be signed by the subject if blood is to be used for DNA analysis (see Section 4.1.4). Subjects who do not wish to have the DNA test will still be eligible for the study. Subjects who elect to have the DNA analysis done may, at any time prior to the end of the study, request that the blood collected for DNA analysis be destroyed. However, this will only be possible prior to samples being de-identified as described below (also see Section 4.1.8).

All specimen and subject identifiers must be removed from the DNA blood samples such that under no circumstances can the DNA blood samples be linked back to a specific subject. In most cases, this will require that DNA blood samples have current labeling removed and the tubes relabeled. Special labeling capable of adhering to frozen tubes must be used. If it is not possible to remove the original labeling, then it must be determined whether the DNA blood samples can go through a freeze thaw and be realiquoted in order to maintain confidentiality. All re-labeled DNA blood samples must be cross referenced to the original demographics in a secure database to ensure that only designated and approved laboratory personnel have access to these data. A special release must be obtained prior to use of specified DNA blood samples.

4.3.10 Estimate of Volume of Blood to Be Collected

The estimated volume of blood to be collected from each subject at each visit (and across all visits) from screening through the SFP is presented in Table 14. If repeats of any blood tests are required, the volume of blood collection will increase accordingly.

Table 14 Estimate of Blood Volume to Be Collected

Visit Day	Estimated Blood Volume (mL)
Screening (Day -28 to Day -1)	31
**Randomized-controlled Period**	
Day 1	37
Day 8	16
Day 15	30.5
Day 29	29.75
Day 57	27.5
Day 85	34.75

Table 14 Estimate of Blood Volume to Be Collected

Visit Day	Estimated Blood Volume (mL)
Day 113	25
Day 155	20
Day 197	37
**Open-label Period**	
Open-label Day 1	19.75 [b]
Open-label Day 15	19
Open-label Day 29	26.5
Open-label Day 92	36
Open-label Day 183	36
Open-label Day 274, then Q26W	26.25
Open-label Day 365, then Q26W	36
**Assessment Visit**	33.75
**Attack Follow-up Visit**	13.3
**Safety Follow-up Period**	
Every 3 months	12.25
**Total**	547.3 [a]

Q26W = every 26 weeks.

[a] Minimum total volume; maximum volume dependent upon subject duration in the study.

[b] Only applicable for subjects where Open-label Day 1 is required.

4.4 Study Suspension or Termination

The Sponsor reserves the right to temporarily suspend or terminate enrollment, RCP, OLP or the entire study at any time for the following reasons:

1. The incidence or severity of AEs in this or other studies indicates a potential health hazard to subjects
2. Subject enrollment is unsatisfactory
3. Non-compliance that might significantly jeopardize the validity or integrity of the trial
4. Sponsor decision to terminate development
5. Sponsor decision to terminate the trial based on a planned futility analysis
6. Recommendation of the independent DMC to discontinue enrollment and transition subjects from the RCP to the OLP due to evidence of efficacy and safety
7. Any other reason which justify this action/actions.

If MedImmune determines that temporary suspension or termination of the study is required, MedImmune will discuss the reasons for taking such action with all participating investigators (or head of the medical institution, where applicable). When feasible, MedImmune will provide advance notice to all participating investigators (or head of the medical institution, where applicable) of the impending action. In the case of the Sponsor discontinuting enrollment upon recommendation of the independent DMC based on evidence of efficacy and safety, subjects in the RCP at the time enrollment is terminated will be given the option to enter the OLP. The rationale for such a decision will be shared and discussed with investigators, IRBs, ethic committees, and regulatory agencies where appropriate. In addition, detailed procedures for transition of patients from the RCP to the OLP will be provided to the investigators in such a case.

If the study or any part of it is suspended or terminated for safety or efficacy reasons, MedImmune will promptly inform all investigators, heads of the medical institutions (where applicable), and/or institutions conducting the study. MedImmune will also promptly inform the relevant regulatory authorities of the suspension/termination along with the reasons for such action. Where required by applicable regulations, the investigator or head of the medical institution must inform the IRB/IEC promptly and provide the reason(s) for the suspension/termination. If the study is suspended for safety reasons and it is deemed appropriate by the Sponsor to resume the study, approval from the relevant regulatory authorities (and IRBs/IECs when applicable) will be obtained prior to resuming the study.

4.5 Investigational Products

4.5.1 Identity of Investigational Product(s)

MedImmune will provide the investigator(s) with investigational product (Table 15) using designated distribution centers.

Table 15 Identification of Investigational Products

Investigational Product	Manufacturer	Concentration and Formulation as Supplied
MEDI-551 (100 mg vial)	MedImmune, LLC	MEDI-551 for IV administration is supplied as 10-mL vials filled with a nominal 10-mL solution of MEDI-551 at a concentration of 10 mg/mL, containing 20 mM histidine/histidine hydrochloride, 70 mM NaCl, 106 mM (4% [w/v]) trehalose dihydrate, and 0.01% (w/v) polysorbate 80, pH 6.0.
Placebo	MedImmune, LLC	Placebo for IV administration is supplied in 10 mL vials filled with 10 mL (nominal) solution containing 10 mM histidine/histidine hydrochloride, 75 mM NaCl, 106 mM (4% [w/v]) trehalose dihydrate, 0.02% (w/v) polysorbate 80, pH 6.0.

IV = intravenous; NaCl = sodium chloride; w/v = weight per volume

The investigational product manager will prepare the investigational product. MEDI-551 and placebo vials must be stored at 2ºC to 8ºC and must not be frozen or shaken. The investigational product (MEDI-551 or placebo) does not contain any novel excipients.

The investigational product must be diluted into a 250 mL 0.9% sodium chloride (NaCl) IV infusion bag for administration as detailed in Section 4.5.1.

Investigational product will be supplied to the site as subject-specific kits containing 3 vials per kit. Each kit has a unique number that is printed on all labels within the kit (ie, the outer carton label and the label of each vial within the carton).

4.5.1.1 Investigational Product Dose Preparation

MEDI-551 or placebo should not be removed from storage until all procedures for subject dosing have been completed. When pulling vials for dosing, immediately remove the vials from the carton and replace the carton in 2ºC to 8°C storage.

If there are any defects noted with the investigational product (MEDI-551 or placebo), immediately notify the investigator and site monitor and refer to the Product Complaint section (Section 4.5.1.4 for further instructions).

Investigational product (MEDI-551 or placebo) must be administered within 4 hours of preparation if the prepared IV bag is stored at room temperature, or within 24 hours if the prepared bag is stored at 2ºC to 8ºC (36°F to 46°F). If there is a delay in the administration of investigational product such that it will not be administered within the specified timeframe, a

new dose must be prepared using a new vial. The dose preparation must be documented in the investigational product accountability log. The same line may be used to administer investigational product (MEDI-551 or placebo) as used to administer premedication.

Investigational Product Inspection

Each vial selected for dose preparation should be inspected. MEDI-551 is supplied as a clear to opalescent, colorless to yellow liquid; free from or practically free from visible particles. The placebo is supplied as a clear to opalescent, colorless to yellow liquid; free from or practically free from visible particles. MEDI-551 is supplied at a concentration of 10 mg/mL for IV infusion after dilution into a 0.9% (weight per volume [w/v]) NaCl IV infusion bag.

If there are any defects noted with the investigational product, the Investigator and Site Monitor should be notified immediately. Refer to the Product Complaint section for further instructions.

Dose Preparation Steps

Preparation of MEDI-551 or placebo into the IV infusion bag is to be performed aseptically. The investigational product manager should remove the tab portion of the vial cap and clean the rubber stopper with 70% ethyl alcohol or equivalent. To avoid foaming, the vial should not be shaken. A vial should only be used one time to prepare a single dose.

MEDI-551 and placebo vials do not contain preservatives and any unused portion must be discarded.

MEDI-551 and placebo have been shown to be compatible when diluted into a polyvinyl chloride (PVC)-free, di(2-ethylhexyl)phthalate (DEHP)-free, and latex-free infusion bag containing a total volume of 250 mL of 0.9% (w/v) NaCl for injection. A single 250 mL infusion bag will be prepared for each subject on each dosing day. To prepare each dose, a 30 mL volume of NaCl must first be removed aseptically from the infusion bag and discarded before either MEDI-551 or placebo is added to the bag. Next, a total of 30 mL of investigational product must be withdrawn aseptically from the 3 supplied investigational product vials in the kit (10 mL per vial) using a syringe and needle and added into the infusion bag using aseptic technique. After the investigational product is added to the infusion bag, the contents of the bag should be gently mixed to ensure homogeneity of the solution within the bag. Do not shake or vigorously agitate the infusion bag.

MEDI-551 and placebo will be identical in appearance and will be clear to opalescent, colorless to yellow liquid, and free from or practically free from particles. The placebo is supplied as a clear to opalescent, colorless to yellow liquid, free from or practically free from particles. MEDI-551 and placebo doses will not be distinguishable during dose preparation, handling, and infusion.

Total in-use storage time from needle puncture of the MEDI-551 or placebo vials to start of administration should not exceed 4 hours at room temperature or 24 hours at 2°C to 8°C (36°F to 46°F). If storage time exceeds these limits, a new dose must be prepared from new vials.

4.5.1.2 Treatment Administration

Randomized-controlled Period

The first day of dosing is considered Day 1.

During the RCP, doses of investigational product must be administered according to the study schedule (Day 1 and Day 15). In the event that a subject or investigative site is unable to adhere to this schedule, a window of ± 3 days is permitted at Day 15. In the event that the second dose of investigational product (scheduled to be given on Day 15 post randomization) is delayed due to a medical/safety reason, dosing must be discussed with the medical monitor prior to administration of investigational product.

If the subject is not dosed within this window they may remain in the RCP.

Open-label Period

For subjects completing Day 197 of the RCP, Day 1 of the OLP should be the same day; however, it may be delayed for up to 14 days (procedures do not need to be repeated). Subjects are not permitted to enter the OLP after 14 days, unless there is a compelling reason discussed with, and agreed to by, the medical monitor, in which case a short extension may be granted.

Subjects in the RCP at the time the 67th AC-determined attack occurs or when enrollment is discontinued upon recommendation of the independent DMC based on evidence of efficacy and safety who wish to enroll in the OLP need to do so as soon as possible, preferably within 14 days. Consulation with the medical monitor is advised in cases where the transition to the OLP is not completed within 14 days.

The first day of dosing of the OLP is considered OLP Day 1.

During the OLP, the same schedule as the RCP applies for OLP Day 1 and Day 15. In the event that the second dose of investigational product (scheduled to be given on Day 15 of the OLP) is delayed due to a medical/safety reason, dosing must be discussed with the medical monitor prior to administration of investigational product. For the 6 monthly dosing visits, a dosing window of ± 7 days will be permitted. If the subject has not returned within the 7-day window in the OLP, the investigator must contact the subject and discuss subsequent subject management with the medical monitor. Subjects may be withdrawn from study medication and enter the SFP at the discretion of the medical monitor.

During both the RCP and the OLP, MEDI-551 (300 mg) or placebo will be administered as an IV infusion using the following guidelines:

1 Women of childbearing potential must have a negative urine pregnancy test prior to receiving MEDI-551 or placebo
2 Subjects with signs or symptoms of clinically significant infections at the time of planned infusion of MEDI-551 or placebo should not be retreated until the infection has resolved.
3 MEDI-551 or placebo must be administered at room temperature by controlled infusion via an infusion pump into a peripheral vein at a rate of 42 mL/hr for the first 30 minutes, with the rate escalated in accordance with Table 16, unless an infusion reaction occurs necessitating the modulation or stopping of the infusion.
4 MEDI-551 or placebo must not be administered via IV push or bolus but as a slow IV infusion. The entire content of the IV bag will be infused using an infusion pump using a 0.22 or 0.2 micron filter.

Table 16 Infusion Times for IV Administration of MEDI-551 300 mg or Placebo

Number of IV Bags	Cumulative Time (minutes)	Infusion Rate (mL/hour)	Infusion Rate (mg/hour)	Total mg Infused
1	0-30	42	50	25
	31-60	125	150	75
	61-90	333	400	200

IV = intravenous

5 A physician must be present at the site or immediately available to respond to emergencies during all administrations of MEDI-551 or placebo. Fully functional resuscitation facilities should be available.

6 At Day 1 and Day 15 of the RCP and OLP Day 1 and OLP Day 15 and at subsequent dosing visits in the OLP, all study subjects will be premedicated with IV methylprednisolone (80-125 mg or equivalent glucocorticoid), PO acetaminophen (500-650 mg) or equivalent dose of paracetamol, and PO diphenhydramine (25-50 mg) or equivalent antihistamine, 30 to 60 minutes prior to MEDI-551 or placebo administration to mitigate infusion reactions.

7 Prior to the start of the infusion, please be sure that the bag content is at room temperature to avoid an infusion reaction due to the administration of the solution at low temperatures.

8 Because of the possible need to vary infusion rates depending on tolerance of the infusion, the total infusion time may exceed the time stated in Table 16. UNLESS AN INFUSION REACTION OCCURS RESULTING IN DISCONTINUATION, THE ENTIRE INFUSION BAG CONTENTS MUST BE ADMINISTERED.

9 If there are no requirements to slow, interrupt or permanently stop the infusion, the anticipated infusion time to deliver each dose is anticipated to be 90 minutes ± 15 minutes.

10 After IV administration, up to an additional 25 mL of saline will be given via infusion pump at the same pump speed utilized at the completion of the initial dosing.

The duration of investigational product infusion and duration of investigational product administration will be calculated as follows:

Duration of Infusion Time: The amount of time elapsed from the infusion start time to the infusion stop time. Infusion start time is defined as the time point wherein investigational product is first infused into the subject. Infusion stop time is defined as the time point where the infusion pump completes infusion of the investigational product, not including the slow clearing of the infusion line. Beware not to rapidly clear the infusion line containing the residual investigational product as it may trigger an infusion reaction.

Duration of Administration: The amount of time elapsed from the infusion pump start time to the infusion pump stop time PLUS the time required to clear the infusion line of residual investigational product. The duration of administration will always be greater than the duration of infusion and will always include the slow clearing of the infusion line.

Both the duration of the investigational product infusion and the duration of investigational product administration will be recorded.

Pharmacokinetic laboratory samples will be collected on Day 1 and Day 15 predose and approximately 15 minutes (± 5 minutes) after the completion of MEDI-551 or placebo administration.

If a subject experiences an AE in the OLP that the investigator assesses as related to investigational product, the subsequent dose may be delayed up to 14 days from the date of the next scheduled dose.

4.5.1.3 Monitoring of Dose Administration

Vital signs (body temperature, BP, pulse rate, and respiratory rate) must be taken with the subject in a semi-supine position as follows:

- Within 60 minutes prior to investigational product administration
- Every 15 minutes (± 5 minutes) during the infusion
- Immediately (± 5 minutes) after the end of the infusion
- Every 30 minutes (± 5 minutes) for 2 hours after dosing or until stable, whichever is later

If a hypersensitivity reaction occurs during the infusion, vital signs will be taken more frequently, as warranted by the severity of the reaction.

As with any antibody, allergic reactions to dose administration are possible. Therefore, appropriate drugs and medical equipment to treat acute anaphylactic reactions must be immediately available, and study personnel must be trained to recognize and treat anaphylaxis.

For Japanese subjects only, the initial dose on Day 1 of the RCP should be administered as a patient in-hospital procedure. The subject can be discharged the next day (Day 2) if no abnormal findings are observed.

4.5.1.4 Reporting Product Complaints

Any defects with the investigational product must be reported *immediately* to the MedImmune Product Complaint Department by the site with further notification to the site monitor. All defects will be communicated to MedImmune and investigated further with the Product

Complaint Department. During the investigation of the product complaint, all investigational products must be stored at labeled conditions unless otherwise instructed.

MedImmune contact information for reporting product complaints:

Email: productcomplaints@medimmune.com

Phone: +1-301-398-2105
+1-877-MEDI-411 (+1-877-633-4411)
Fax: +1-301-398-8800

Mail: MedImmune, LLC
Attn: Product Complaint Department
One MedImmune Way,
Gaithersburg, MD USA 20878

4.5.2 Additional Study Medications

4.5.2.1 Oral Corticosteroid Use

Subjects will receive 2 weeks of oral corticosteroids (prednisone 20 mg/day or equivalent oral glucocorticoid) from Day 1 to Day 14. The following tapering schedule will be implemented from Day 15:

- 15 mg prednisone PO on Day 15
- 10 mg prednisone PO on Day 16
- 7.5 mg prednisone PO on Day 17
- 5 mg prednisone PO on Days 18 and 19
- 2.5 mg prednisone PO on Days 20 and 21

Sites should utilize local supplies of oral corticosteroids, where possible, and monitor compliance. The Sponsor will provide reimbursement during the study.

4.5.2.2 Rescue Medications

Assessment of new symptoms or worsening of existing symptoms should be completed within 5 days to determine if an attack has occurred. Treatment of an attack should preferably be initiated after completion of the attack assessment and the determination that the protocol attack criteria have been met. However, the Principal Investigator can initiate rescue therapy at any time before full assessment is completed. Rescue therapy will be given as directed by the investigator and may include IV corticosteroids, IVIG, and/or PLEX.

Sites should utilize local supplies of IV steroids, where possible, and the Sponsor will provide reimbursement during the study.

All medications provided to the subject during the study will be recorded on the source document and include the dose, start and stop dates, frequency, and route of administration and reason for product administration.

4.5.3 Dose Adjustments and Missed Doses

Dose adjustments are not permitted in this study. If, in the clinical judgment of the investigator, a subject is not well enough to receive investigational product, the dose should be omitted. In the event that a dose of investigational product is delayed due to medical/safety reasons, dosing and subject management must be discussed with the medical monitor prior to administration of further investigational product.

A dosing window of ± 7 days will be permitted at the 6 monthly visits.

4.5.4 Labeling

Labels will be prepared in accordance with Good Manufacturing Practice (GMP) and local regulatory guidelines. The label will fulfill GMP Annex 13 requirements for labeling. Label text will be translated into local languages, as required.

4.5.5 Storage

MEDI-551 or placebo is to be stored at 2°C to 8°C (36°F to 46°F).

4.5.6 Treatment Compliance

Investigational product will be administered by study site personnel, who will monitor compliance.

4.5.7 Accountability

The investigator's or site's designated investigational product manager is required to maintain accurate investigational product accountability records. The MedImmune representative who visits the study site to monitor the data will regularly review these records to ensure completion and compliance. Upon completion of the study, copies of investigational product accountability records will be returned to MedImmune. All unused investigational product will be returned to a MedImmune-authorized depot or disposed of upon authorization by MedImmune.

4.6 Treatment Assignment and Masking

4.6.1 Methods for Assigning Treatment Groups

An IVRS/IWRS will be used for randomization to a treatment group and assignment of masked investigational product kit numbers. A subject is considered randomized into the study when the investigator notifies the IVRS/IWRS that the subject meets eligibility criteria and the IVRS/IWRS provides the assignment of masked investigational product kit numbers to the subject.

Subjects will be randomized at a 3:1 ratio with a permuted block randomization scheme to receive either MEDI-551 or placebo within each AQP4-IgG stratum determined at screening (seropositive versus seronegative). Enrollment will be further stratified by Japan versus non-Japan region.

4.6.2 Methods for Ensuring Masking

This is a double-masked study in which MEDI-551 and placebo are identically labeled and indistinguishable in appearance. As such, neither the subject/legal representative nor any of the investigator or Sponsor staff who are involved in the treatment or clinical evaluation of the subjects will be aware of the treatment received (ICH E9) (see Section 4.6.4.2 for unmasking related to interim analysis). In the event that treatment allocation for a subject becomes known to the investigator or other study staff involved in the management of study subjects, the Sponsor must be notified *immediately*. If the treatment allocation for a subject needs to be known to treat an individual subject for an AE, the investigator must notify the Sponsor *immediately* and, if possible, before unmasking the treatment allocation.

Administration of the masked dose of MEDI-551 or placebo on OLP Day 15 is necessary to correctly administer the loading dose of 600 mg IV MEDI-551 to subjects previously randomized to the placebo group during the RCP, or to ensure that subjects previously randomized to MEDI-551 do not receive an extra treatment dose. This masking mechanism will be implemented through the IVRS to ensure that treatment during the RCP is not revealed to the sites.

4.6.3 Methods for Reducing Bias

The following steps are being planned to mitigate/reduce bias towards the assessment of an NMO/NMOSD attack:

1. Study subjects will be instructed not to discuss AEs or NMO/NMOSD-related symptoms/medications with any of the independent assessors.
2. The EDSS rater will not have access to prior EDSS scores/FSS at the time of a new evaluation and will perform the evaluation of the subject without any knowledge of the subject’s AE history or management of symptoms or findings from physical/neurological

examinations. The EDSS rater will be instructed NOT to elicit or discuss the subjects' experiences or AEs on the study. The EDSS rater must not manage the subject outside of the protocol for routine care or any other purpose. The EDSS scores will be captured electronically and transmitted to the investigator who will have access to the scores to compare against the protocol-defined criteria for an NMO/NMOSD attack. The EDSS scores/FSS will be monitored centrally by an independent expert.

3 The ophthalmology examiner will not have access to prior visual acuity and RAPD at the time of a new evaluation and perform the evaluation of the subject without any knowledge of the subject's AE history or management of symptoms or findings from physical/neurological examination. The ophthalmology examiner will be instructed NOT to elicit or discuss the subjects' experiences or AEs on the study. The ophthalmology examiner must not manage the subject outside of the protocol for routine care or any other purpose. The visual acuity scores and RAPD findings will be captured electronically and transmitted to the investigator who will have access to the score to compare against the protocol-defined criteria for an NMO/NMOSD attack.

4 The neuro-radiologist will not have access to the subject's AE history or management of symptoms or findings from physical/neurological examination.

5 To minimize the potential influence of MRI findings on clinical assessment of possible attacks, MRI images/study report should not be reviewed by the Principal Investigator unless a specific criterion for an attack requires review of the MRI. In such cases, review of the MRI image and local study report MUST be done AFTER all relevant clinical assessments are completed. The time sequence of all procedures and reviews required for assessment will be documented to demonstrate compliance.

6 To further minimize the potential influence of MRI findings on the adjudication of possible attacks, the AC will not be given access to the MRIs (where required) until after their evaluation of the clinical data is complete.

7 To minimize the potential influence of the Principal Investigator's review on the AC, the AC will not be provided with the Principal Investigator's opinion as to whether an attack has occurred or not, nor will the AC receive information about which protocol attack criterion was met and the rescue medication utilized.

8 To assess for a potential bias in the investigator's assessment of new symptoms, the data generated from this assessment, which determined that the symptoms were not related to NMO/NMOSD, will be sent to review by the AC. The level of agreement between the investigator and the AC will be assessed.

4.6.4 Methods for Unmasking

The treatment code should not be broken except in medical emergencies when the appropriate management of the subject requires knowledge of the treatment randomization. The

investigator documents and reports the action to MedImmune, without revealing the treatment given to subject to the MedImmune staff.

MedImmune retains the right to break the code for SAEs that are unexpected and are suspected to be causally related to an investigational product and that potentially require expedited reporting to regulatory authorities.

4.6.4.1 Unmasking in the Event of a Medical Emergency

In the event of a medical emergency, the investigator must first attempt to contact the medical monitor to discuss the medical emergency and the reason for wanting to unmask the subject's investigational product allocation prior to proceeding with unmasking. (Czech Republic only: in the event of a medical emergency that may be related to investigational product, the investigator may unmask an individual subject's investigational product allocation if he/she feels it is required without prior contact to the medical monitor/sponsor.) Unmasking should occur only if the subject's medical emergency would be managed differently as a result of the subject's having received investigational product. In most cases, management of the medical emergency would be the same whether or not the subject received investigational product. If this is the case, the investigational product allocation should *not* be unmasked.

In exceptional circumstances, where in the clinical judgment of the investigator the medical emergency necessitates that the subject's investigational product allocation be immediately unmasked, the investigator may proceed with unmasking without prior discussion with the medical monitor.

Instructions for unmasking an individual subject's investigational product allocation are contained in the IVRS/IWRS manual.

4.6.4.2 Unmasking for Interim Analysis Purposes

An unmasked interim analysis is planned for this study as described in Section 4.8.10. The interim analysis to determine futility will be conducted by the DMC; Sponsor, and sites will remain masked to the treatment assignment post-interim analysis.

4.6.4.3 Unmasking due to Known or Hypothesized Effects of MEDI-551

MEDI-551 is known to deplete CD19+ B-cells; therefore, the results of flow cytometry to count B-cells are potentially unmasking. These data will not be made available to investigation sites post randomization through the remainder of the study.

Data from early phase development in non-oncology subject populations receiving MEDI-551 suggest that MEDI-551 administration may be associated with potential unspecified mild reduction in total Ig in individual subjects. Since this reduction may be potentially unmasking, these data will not be made available to investigational sites post randomization through the remainder of the study.

MEDI-551 is hypothesized to reduce titers of AQP4-IgG. AQP4-IgG titers from the central laboratory will not be made available to investigational sites at any point in the study.

MEDI-551 is known to reduce the plasma cell gene signature. Because the results of the plasma cell gene signature assay could potentially be unmasking, these samples will not be tested prior to un-masking of the study.

MEDI-551 may also reduce tetanus vaccine titers; therefore, the results of the vaccine titer assay will not be mabe available to the sites at any point during the study.

Any potentially unmasking data from the protocol will not be available to the sponsor until the study has been unmasked following completion of the RCP.

Investigators must not request any potentially unmasking laboratory results at a local or other central laboratory.

4.7 Restrictions During the Study and Concomitant Treatment(s)

The investigator must be informed as soon as possible about any medication taken from the time of screening until the end of the study (final study visit). Any concomitant medication(s), including herbal preparations, taken during the study will be recorded.

4.7.1 Permitted Concomitant Medications

Investigators may prescribe concomitant medications or treatments deemed necessary to provide adequate supportive care except for those medications identified as “excluded” as listed in Section 4.7.2. Specifically, subjects should receive full supportive care during the study, including transfusions of blood and blood products, and treatment with antibiotics, anti-emetics, anti-diarrheals, and analgesics, and other care as deemed appropriate, and in accordance with their institutional guidelines.

Subjects who experience an NMO/NMOSD attack should be given rescue therapy according to the judgment of the investigator as described in Section 4.5.2.2.

All concomitant and rescue therapy given to the subject during the study will be recorded on the source document and includes, but is not limited to, the dose, start and stop dates, frequency, route of administration, and reason for administration.

4.7.1.1 Immunosuppressive Medications During the Screening Period

Subjects that have previously been taking immunosuppressive medications (including but not limited to, AZA and MMF) and steroids not specifically excluded in Section 4.1.3 for the prevention or treatment of NMO/NMOSD relapses may continue to take those medications during the screening period as long as the dose remains stable or decreases.

Immunosuppressive medications must be stopped prior to dosing on Day 1. Steroids must continue according to the protocol (see Section 4.5.2.2).

4.7.2 Prohibited Concomitant Medications

The following medications (and procedure) are prohibited from the day of dosing on Day 1 through the end of the subjects' participation in the OLP:

- Any immunosuppressant for the prevention of NMO/NMOSD attacks (this includes but is not limited to AZA, MMF, rituximab, etc)
- High-dose steroids, with the exception of those required to treat an NMO/NMOSD attack
- Low-dose steroids, unless discussed and agreed with the medical monitor, with the exception of those specified by the protocol in the RCP and topical steroids
- PLEX, unless used to treat an NMO/NMOSD attack.
- Alemtuzumab
- Eculizumab
- Total lymphoid irradiation
- Bone marrow transplant
- T-cell vaccination therapy
- Natalizumab
- Cyclosporin
- Methotrexate
- Mitoxantrone
- Cyclophosphamide
- Tocilizumab
- Any other immunosuppressant medication, unless discussed and approved by the medical monitor

In addition to the medications described above, subjects must be instructed not to take any medications, including over-the-counter products, without first consulting with the investigator.

Subjects may exit the OLP at any time for any reason, including seeking alternative treatment options, at which point they will enter the SFP.

There are no prohibited medications during the SFP. During the SFP, a subject may receive standard treatment for their NMO/NMOSD at the discretion of the investigator and in compliance with established clinical practice.

4.8 Statistical Evaluation

4.8.1 General Considerations

Tabular summaries will be presented by treatment group. Categorical data will be summarized by the number and percentage of subjects in each category. Continuous variables will be summarized by descriptive statistics. Additional details of statistical analyses will be described in the statistical analysis plan.

The primary and key secondary endpoints will be analyzed and reported based on both AQP4-IgG seropositive and Intent-to-Treat (ITT) population, defined as all subjects who are randomized into the study and receive at least one dose of investigational product. Additional efficacy analyses will be performed in both seropositive and ITT populations. Treatment groups will be analyzed according to the initial randomization, regardless of whether subjects receive an investigational product different from that to which they were randomized.

The safety data will be presented using the as-treated population, which will include all subjects who receive any investigational product. Specifically, subjects who received all placebo doses will be included in the placebo group for safety analyses; conversely, subjects who received at least one dose of MEDI-551 will be included in the active treatment group for safety analyses.

Efficacy and safety data from the OLP will be presented based on subjects who receive at least one dose during the OLP.

Unless otherwise specified, all statistical inference will be done at a significance level of two-sided $\alpha = 0.05$.

4.8.2 Sample Size and Power Calculations

The current study is being planned to detect a target relative reduction of 60% in risk for time from Day 1 to onset of an AC-determined NMO/NMOSD attack on or before Day 197 with 90% power and $\alpha = 0.05$ (two-sided). Details are provided in Appendix 9. A total of 67 AC-determined NMO/NMOSD attacks are required for the ITT population. Subjects will be randomized in a 3:1 ratio to receive either MEDI-551 or placebo within both AQP4-IgG seropositive and seronegative strata. The stratification ratio is anticipated to be approximately 80:20 with higher allocation to the seropositive cohort. If the seropositive cohort has 80% of the attacks, the study will have approximately 82% power to detect the target relative reduction of 60%.

A maximum of 252 subjects will be randomized and dosed in this study. Because this study is event-driven where the primary objective is based on a total of 67 AC-determined NMO/NMOSD attacks, and the rate of NMO/NMOSD attacks is not established in the literature, this number of subjects was determined by analyzing masked data of the actual

attack rate from the first 78 subjects who completed the RCP in this study. The attack status (attack/no attack) of the 78 subjects was randomly sampled with different attack rate which gave an estimate of the number of attacks for the total sample size. This simulation process was repeated 10,000 times to give a distribution of the number of attacks for the total sample size, from which the probability of observing at least 67 attacks could be estimated. Based on the 78 completed subjects, this procedure showed that with 252 subjects there is a 90% probability of reaching the required 67 AC-determined attacks.

Within each AQP4-IgG stratum, randomization will also be stratified by region (Japan vs non-Japan). The number of Japanese subjects will be determined primarily by feasibility and will not depend on a minimum number of NMO/NMOSD attacks to be observed from Japanese subjects.

4.8.3 Efficacy

4.8.3.1 Primary Efficacy Analysis

The following are the null and alternative hypotheses associated with the primary endpoint:

$$H_{01}: HR^{+} = 1 \text{ vs } H_{11}: HR^{+} \neq 1,$$

$$H_{02}: HR^{ALL} = 1 \text{ vs } H_{12}: HR^{ALL} \neq 1,$$

where HR^{+} indicates the hazard ratio of AC-determined NMO/NMOSD attack for MEDI-551 relative to placebo in AQP4-IgG seropositive subjects. HR^{ALL} denotes the same quantity for the ITT population. The treatment effect in the seropositive cohort will be defined as the relative reduction in HR^{+}; ie, 100 × (1-HR^{+}). A value of $HR^{+} < 1$ or a positive value of relative reduction will indicate the efficacy of MEDI-551 compared to placebo in the seropositive cohort. Similar interpretations will hold for HR^{ALL}. For the current study, the target treatment effect of 60% implies a hazard ratio (HR) of 0.4.

For the AQP4-IgG seropositive cohort, the treatment effect will be assessed using the Cox proportional hazards model with treatment indicator (MEDI-551 or placebo) as an explanatory factor; whereas for the ITT population, the model will also include serostatus as an additional explanatory factor. The HR of MEDI-551 versus placebo will be estimated together with its associated 95% confidence interval (CI). The SAS PROC PHREG will be used for fitting this model. Subjects who complete Day 197 of the RCP or discontinue the study before Day 197 for reasons other than an AC-determined NMO/NMOSD attack will be censored in this model at the time of the Day 197/discontinuation visit. See Section 4.8.9 for details regarding Type I error control.

An indicator variable for Japan versus non-Japan region will not be included in the Cox regression model due to the low number of anticipated Japanese subjects. An evaluation of

consistency of treatment effect in Japanese subjects with that observed in the global study will be determined.

The data cutoff date for the primary analysis will be when the last subject completes the discontinuation visit following the 67th AC-determined attack, after all subjects complete the RCP if 67 AC-determined attacks do not occur, or after all subjects complete the EDV following discontinuation of enrollment upon recommendation of the independent DMC based on evidence of efficacy and safety. Subjects who are in the RCP when a total of 67 AC-determined NMOSD attacks have occurred or when enrollment is discontinued upon recommendation of the DMC based on evidence of efficacy and safety will discontinue the RCP as outlined in Section 4.5.1.2.

Since some AC-determined attacks may not be assessed within the assessment windows, a broader window will be used for primary analysis. Only AC-determined attacks with an assessment visit scheduled within 120 hours (5 days) of reporting symptoms and all assessments done within 10 days of an Assessment Visit will be included in the primary analysis. AC-determined attacks not included in primary analysis will be included in other supportive analyses. Sensitivity analysis will be conducted to assess the impact of the loss of any AC-determined attacks outside of the identified assessment window.

4.8.3.2 Additional Analyses of the Primary Endpoint

An estimate of the cumulative event rates through the end of the RCP will be calculated using the Kaplan-Meier (KM) method. Graphical displays of KM estimates and cumulative incidence estimates will be presented by treatment group.

The following sensitivity analyses of the primary endpoint will be performed:

1. Analysis using the Cox regression model and the following baseline characteristics and treatment as exploratory variables:
    - Number of NMO/NMOSD relapses prior to randomization
    - Baseline EDSS score
2. Analysis similar to primary analysis but considering only unanimous AC-determined NMO/NMOSD attacks (all 3 adjudicators agree) as events, remaining subjects will be considered as censored.
3. Analysis similar to primary analysis but also considering subjects who prematurely discontinue the RCP without experiencing an AC-determined NMO/NMOSD attack as treatment failures (events); remaining subjects will be considered as censored.
4. Sensitivity analysis will be conducted to assess the impact of the loss of any AC-determined attacks outside of the identified assessment window in the primary analysis.

4.8.3.3 Secondary Efficacy Analyses

Secondary endpoints 1 through 4 presented in Section 2.2.2 for AQP4-IgG seropositive cohort will be analyzed as follows:

- Treatment effect for secondary efficacy endpoints based on EDSS worsening will be assessed using a logistic regression model with treatment and baseline EDSS as explanatory variables. The percentage of subjects meeting the endpoints, odds ratios, p-value, and 95% CIs of the odds ratios will be presented.
- The treatment effect for the low-contrast visual acuity measured by change from baseline in low-contrast Landolt C Broken Rings Chart binocular scores will be assessed using an analysis of covariance model using treatment and baseline Landolt C Broken Rings Chart binocular score as explanatory variables.
- The treatment effect for the secondary efficacy endpoints based on the cumulative number of active MRI lesions and number of NMO/NMOSD-related in-patient hospitalizations will be tested using Negative Binomial regression with treatment as an explanatory variable.

Similar analyses will be performed for the ITT population by extending the above mentioned models to include an indicator variable for serostatus as well.

See Section 4.8.9 for details regarding Type I error control.

Annualized attack rate (total number of AC-determined NMO/NMOSD attacks normalized by person-years) during any exposure to MEDI-551 will be summarized descriptively.

4.8.3.4 Subgroup Analyses

Consistency of treatment effect measured by primary and key secondary efficacy endpoints in the following subgroups will be investigated as follows:

- Sex (male vs female)
- Baseline EDSS (< 5 vs ≥ 5)
- Number of prior NMO/NMOSD relapses (< 2 vs ≥ 2)
- Disease duration category (< 5 years vs ≥ 5 years)
- AQP4-IgG serostatus (positive vs negative) as determined at screening

The nominal p-value and 95% CIs of treatment effect will be provided for each subgroup analysis.

4.8.4 Safety

4.8.4.1 Analysis of Adverse Events

The safety and tolerability of MEDI-551 will be assessed at completion of the study primarily by summarizing TEAEs including TESAEs. The occurrence of TEAEs will be collected and

summarized from the commencement of infusion of investigational product through the end of study. Treatment-emergent AEs and SAEs will be summarized by system organ class and preferred terms, by severity, and by relationship to the investigational product. For the OLP, the rate of AEs normalized by patient-years may be provided.

4.8.4.2 Analysis of Clinical Laboratory Parameters, Physical Examination Findings, and Vital Sign Measurements

Laboratory measurements as well as their changes from baseline at each collection time point and shift from baseline if applicable will be summarized descriptively. Significant physical examination findings and vital sign measurements will also be summarized using descriptive analyses.

4.8.5 Patient-reported Outcomes

4.8.5.1 Short Form-36 Health Survey

Treatment effect for the PRO endpoint based on SF-36 PCS (4-week recall) change from baseline at the last visit of the RCP will be tested using an analysis of covariance model using treatment and baseline SF-36 PCS score as explanatory variables. Similar analysis will be done for SF-36 MCS.

For the 8 remaining domains, summaries of change from baseline will be presented.

4.8.5.2 Pain NRS

Treatment effect for pain in 5 locations (eyes, legs, arms, upper back, and lower back) based on separate NRS change from baseline at the last visit of the RCP will be tested using analyses of covariance models using treatment and baseline pain NRS scores as explanatory variables.

4.8.6 Analysis of Immunogenicity, Pharmacokinetics, and Exploratory Biomarkers

4.8.6.1 Analysis of Immunogenicity

The presence of ADAs to MEDI-551 in serum will be assessed over the duration of the study. Immunogenicity results will be analysed by summarizing the number and percentage of subjects who develop detectable ADA by treatment group. The association of ADA with MEDI-551 concentration, blood B-cell levels, attack rate, and TEAEs may be evaluated.

4.8.6.2 Analysis of Pharmacokinetics

MEDI-551 concentration will be summarized descriptively over visits separated by AQP4-IgG seropositive and seronegative NMO/NMOSD subjects. Individual and mean MEDI-551 concentration versus time data will be plotted by AQP4-IgG seropositive and seronegative subjects. Pharmacokinetic parameters will be estimated using noncompartmental methods. Pharmacokinetic parameters will be listed by subjects and separated by AQP4-IgG seropositive and seronegative NMO/NMOSD subjects and summarized descriptively.

Population PK or PK/PD analysis may be conducted to include the exploratory analysis to identify the covariates that affect MEDI-551 PK in this subject population, or to investigate the relationship between MEDI-551 PK, PD, and response as appropriate.

4.8.6.3 Analysis of Exploratory Biomarkers

AQP4-IgG titer, plasma gene signature change from baseline, and B-cell absolute counts and relative percentage based on baseline will be summarized descriptively by treatment groups.

Additional research data and associated exploratory analyses may be reported separately from the main clinical study report.

4.8.7 Additional Analyses

An exploratory analysis of time to AC-determined NMO/NMOSD attack or rescue therapy without an AC-determined NMO/NMOSD attack during the RCP will be conducted by considering rescue therapy as events of interest as well. Subjects who never experience any AC-determined NMO/NMOSD attack nor receive rescue therapy will be censored for this analysis.

The agreement between site-determined NMO/NMOSD attack and AC- determined NMO/NMOSD attacks will be assessed by Kappa statistic.

An inter- and intra-rater reliability assessment will be performed based on a random selection of all events contributing to the primary analysis.

A shift analysis of the scores from the mRS will be performed.

4.8.8 Data Monitoring Committee

An unmasked independent DMC will perform evaluations of safety and clinical outcome data at specified regular intervals throughout the study and make recommendations to the sponsor regarding further conduct of the study. The DMC will conduct an interim analysis for futility as described in the description of the interim analysis in Section 4.8.10.

The DMC will monitor drop-out and missing primary and secondary outcome data on a case by case basis. In addition, the DMC will assess the thoroughness of the screening for possible NMO/NMOSD attacks and the effectiveness of the masking.

The DMC will receive ‘real time’ reports of new NMO/NMOSD attacks and will review the adherence of the investigators and AC to the process of attack diagnosis and adjudication.

Additional details will be provided in a separate DMC charter.

4.8.9 Control of Type I Error

The study is designed to strongly control the overall Type I error rate of $\alpha = 0.05$ based on the Bonferroni-based chain procedure (Bretz et al, 2009; Millen and Dmitrienko, 2011). The primary null hypothesis will be hierarchically tested first at $\alpha = 0.05$ in the AQP4-IgG seropositive cohort, and, if significant, it will be further tested in the ITT population at $\alpha = 0.05$. If and only if the treatment group comparison is statistically significant within the ITT population, secondary hypotheses will be tested. Null hypotheses for the 4 key secondary endpoints will follow the same sequential testing strategy as the primary analysis (testing within seropositive subjects first, followed by the ITT population if the comparison within seropositive subjects is statistically significant). Each secondary hypothesis will be initially tested based on the Bonferroni method at $\alpha = 0.05/4 = 0.0125$. If the null hypothesis for a particular secondary endpoint is rejected across both the seropositive and the ITT populations, the Type I error saved will be propagated equally to other non-rejected sets of secondary null hypotheses. The testing procedure will be repeated until all null hypotheses are rejected or no further null hypothesis can be rejected. Details are described in Appendix 11.

4.8.10 Interim Analysis for Futility

An unmasked interim analysis will be conducted for futility assessment by an independent DMC when approximately 50% of the total planned AC-determined NMO/NMOSD attacks occur in this study. The information fraction will be determined by the ratio of the number of observed cases of AC-determined NMO/NMOSD attacks and the planned number of AC-determined NMO/NMOSD attacks. The predictive power will be calculated for the primary efficacy endpoint in the AQP4-IgG seropositive cohort as well as the ITT population based on the empirical trend observed at the interim analysis (Lan et al, 2009). The study may be declared as "futile" if calculated predictive power at interim is < 20% in both AQP4-IgG seropositive cohort and the ITT population. Details are provided in Appendix 10.

5 ASSESSMENT OF SAFETY

5.1 Definition of Adverse Events

The ICH Guideline for Good Clinical Practice (GCP) E6(R1) defines an AE as:

Any untoward medical occurrence in a patient or clinical investigation subject administered a pharmaceutical product and which does not necessarily have a causal relationship with this treatment. An AE can therefore be any unfavorable and unintended sign (including an abnormal laboratory finding), symptom, or disease temporally associated with the use of a medicinal product, whether or not considered related to the medicinal product.

An AE includes but is not limited to any clinically significant worsening of a subject's pre-existing condition. An abnormal laboratory finding (including ECG finding) that requires medical intervention by the investigator, or a finding judged by the investigator as medically

significant should be reported as an AE. If clinical sequelae are associated with a laboratory abnormality, the diagnosis or medical condition should be reported (eg, renal failure, hematuria) not the laboratory abnormality (eg, elevated creatinine, urine red blood cells increased). Abnormal laboratory values that are not, in the investigator's opinion, medically significant and do not require intervention should not be reported as AEs.

Adverse events may be treatment emergent (eg, occurring after initial receipt of investigational product) or nontreatment emergent. A nontreatment-emergent AE is any new sign or symptom, disease, or other untoward medical event that begins after written informed consent has been obtained but before the subject has received investigational product.

Elective treatment or surgery or preplanned treatment or surgery (that was scheduled prior to the subject being enrolled into the study) for a documented pre-existing condition that did not worsen from baseline is not considered an AE (serious or nonserious). An untoward medical event occurring during the prescheduled elective procedure or routinely scheduled treatment should be recorded as an AE or SAE.

For the purposes of this protocol, NMO/NMOSD attacks will not be recorded as an AE during either the RCP or the OLP. Symptoms that are subsequently diagnosed due to another underlying cause will be recorded as AEs.

5.2 Definition of Serious Adverse Events

An SAE is any AE that:

- Results in death (for the purposes of this protocol, this includes NMO/NMOSD disease progression or attack)
- Is immediately life-threatening
- Requires inpatient hospitalization or prolongation of existing hospitalization
- Results in persistent or significant disability/incapacity
- Is a congenital anomaly/birth defect in offspring of the subject
- Is an important medical event that may jeopardize the subject or may require medical intervention to prevent one of the outcomes listed above

Medical or scientific judgment should be exercised in deciding whether expedited reporting is appropriate in this situation. Examples of medically important events are intensive treatment in an emergency room or at home for allergic bronchospasm, blood dyscrasias, or convulsions that do not result in hospitalizations; infection that requires treatment with IV antibiotics; or development of drug dependency or drug abuse.

For the purposes of this protocol, NMO/NMOSD disease progression or attack that do not result in death, but meet any of the other above listed SAE criteria, will not be reported as an SAE unless it occurs during the screening period or in the SFP.

NMO/NMOSD disease progression or attacks that result in death will be reported as SAEs.

All occurrences of NMO/NMOSD attacks will be carefully monitored by the site staff and the Sponsor to ensure these are identified in the required reporting timeline.

Additionally, hospitalization for any procedure resulting from an NMO/NMOSD attack will *not* be reported as an SAE (eg, PLEX that could result in hospitalization).

5.3 Definition of Adverse Events of Special Interest

An adverse event of special interest (AESI) is one of scientific and medical interest specific to understanding of the Investigational Product and requires close monitoring and rapid communication by the investigator to the Sponsor. An AESI may be serious or non-serious. The rapid reporting of AESIs allows ongoing analysis of these events in order to characterize and understand them in association with the use of this investigational product.

Two adverse events are considered AESIs for this study: 1) hepatic function abnormality meeting the definition of Hy's law; and 2) PML. See Section 5.6.2 and Section 5.6.3 for the definition and reporting of AESIs of hepatic function abnormality and PML, respectively.

5.4 Recording of Adverse Events

Adverse events will be recorded on the eCRF using a recognized medical term or diagnosis that accurately reflects the event. Adverse events will be assessed by the investigator for severity, relationship to the investigational product, possible etiologies, and whether the event meets criteria of an SAE and therefore requires immediate notification to the Sponsor's Patient Safety designee as described in Section 5.5. See Section 5.2 for the definition of SAEs and Appendix 3 and Appendix 12 for guidelines for assessment of severity and relationship. If an AE evolves into a condition that meets the regulatory definition of "serious," it will be reported on the SAE Report Form.

Infusion of biological products is commonly associated with infusion related reactions. Anaphylaxis and infusion related reactions have some common manifestations and may be difficult to distinguish from each other. Infusion related reactions are commonly observed during or shortly after the first time exposure to therapeutic monoclonal antibodies delivered through intravenous infusion. These reactions are less common following subsequent exposures. Unlike infusion related reactions, anaphylaxis is a rare event, usually occurring after subsequent exposure to an antigen, and it is most commonly accompanied by severe

systemic skin and or mucosal reactions. The investigator is advised to carefully examine symptoms of adverse reactions observed during or shortly after exposure to MEDI-551, and consider the above-mentioned facts prior to making a final diagnosis. Reactions occurring at the time of or shortly after subsequent infusions of investigational product are to be judged by the investigator at his/her own discretion. For the investigator's convenience and in order to facilitate consistency in judgments a copy of the NIAID and FAAN guidance for anaphylaxis diagnosis is provided in Appendix 2.

5.4.1 Time Period for Collection of Adverse Events

Adverse events will be collected from the time of signature of informed consent throughout the treatment periods, including the SFP.

All SAEs will be recorded from the time of informed consent.

5.4.2 Follow-up of Unresolved Adverse Events

Any AEs that are unresolved at the subject's last AE assessment or other assessment/visit as appropriate in the study are followed up by the investigator for as long as medically indicated, but without further recording in the eCRF. The Sponsor (or designee) retains the right to request additional information for any subject with ongoing AE(s)/SAE(s) at the end of the study, if judged necessary.

5.5 Reporting of Serious Adverse Events

Within 24 hours of identifying an SAE, regardless of the presumed relationship to the investigational product, the investigator or qualified designee must complete the SAE Report Form and submit by email to the Sponsor's designated safety reporting address below. The report may be submitted by fax if email is unavailable.

**Safety Reporting e-mail address:**	**icon-mads@iconplc.com**
**Fax (if email is unavailable):**	**+1-215-616-3096**

The Sponsor/desginee is responsible for reporting certain SAEs as expedited safety reports to applicable regulatory authorities, ethics committees, and participating investigators, in accordance with ICH Guidelines and/or local regulatory requirements. The Sponsor/designee may be required to report certain SAEs to regulatory authorities within 7 calendar days of being notified about the event; therefore, it is important that investigators submit additional information requested by the Sponsor as soon as it becomes available.

Investigators should provide all available information at the time of SAE Report Form completion. Investigators should not wait to collect additional information to fully document the event before notifying the Sponsor/designee of an SAE. When additional information

becomes available, investigators should submit a follow-up SAE Report Form (separate from the initial report form) with the new information. Any follow-up information to an SAE also needs to be provided to the Sponsor/designee within 24 hours of learning of the new information.

5.6 Other Events Requiring Immediate Reporting

5.6.1 Overdose

An overdose is defined as a subject receiving a dose of investigational product in excess of that specified in this protocol, or receiving investigational product at a time/visit not specified in this protocol.

Any overdose of a study subject with the investigational product, with or without associated AEs/SAEs, must be reported within 24 hours of knowledge of the event to the Sponsor/designee using the Overdose Notification Form and the email address below (or by fax if email is unavailable):

**Safety Reporting e-mail address:**	**icon-mads@iconplc.com**
**Fax (if email is unavailable):**	**+1-215-616-3096**

If the overdose results in an AE, the AE must also be recorded on the AE eCRF (see Section 5.4). Overdose does not automatically make an AE serious, but if the consequences of the overdose are serious, for example death or hospitalization, the event is serious and must be reported as an SAE (see Section 5.4 and Section 5.5). The Sponsor/designee does not recommend specific treatment for an overdose. The investigator will use clinical judgment to treat any overdose.

5.6.2 Hepatic Function Abnormality

Events of hepatic function abnormality of special interest to the Sponsor are defined as any increase in ALT or AST to greater than 3 × ULN **and concurrent** increase in bilirubin to greater than 2 × ULN (ie, Hy's law cases). Concurrent findings are those that derive from a single blood draw or from separate blood draws taken within 8 days of each other. In the event of hepatic function abnormality where the etiology is unknown, timely follow-up investigations and inquiries should be initiated by the investigational site, based on medical judgment, to make an informed decision regarding the etiology of the event.

All occurrences of hepatic function abnormality that meet the criteria of Hy's Law are to be considered SAEs and reported to the Sponsor/designee within 24 hours as described in Section 5.5. If the underlying diagnosis for the hepatic function abnormality is known (including progression of pre-existing disease), the diagnosis should be recorded as the SAE.

If the underlying diagnosis for the hepatic function abnormality remains unknown, the term "hepatic function abnormal" should be used to report the SAE.

The investigator will review the data with the medical monitor. The investigator should then use clinical judgment to establish the cause based on local standard of care and follow the subject by conducting testing as clinically indicated.

If, after appropriate workup, in the opinion of the investigator, the underlying diagnosis for the abnormality remains unexplained, or is considered attributable to investigational product, dosing of the study subject should be permanently discontinued.

Each reported event of hepatic function abnormality will be followed by the investigator and evaluated by the Sponsor.

5.6.3 Progressive Multifocal Leukoencephalopathy

PML is an AESI and a potential risk for MEDI-551. Due to the potential challenge in the differential diagnosis, any possible case of PML should be reported within 24 hours of awareness to the Sponsor/designee using the Possible PML Notification Form and the email address below (or by fax if email is unavailable):

**Safety Reporting e-mail address:**	**icon-mads@iconplc.com**
**Fax (if email is unavailable):**	**+1-215-616-3096**

A confirmed diagnosis of PML requires the presence of JCV demonstrated in either CSF or brain tissue biopsy samples. Contact the medical monitor for instructions on sample handling and analysis.

Details about PML risk are provided in the IB.

5.6.4 Pregnancy

Pregnancy in a female subject who has received investigational product is required to be reported within 24 hours of knowledge of the event to the Sponsor/designee using the Pregnancy Notification Form and the email address below (or by fax if email is unavailable):

**Safety Reporting e-mail address:**	**icon-mads@iconplc.com**
**Fax (if email is unavailable):**	**+1-215-616-3096**

Subjects who become pregnant during the study period must not receive additional doses of investigational product and will be withdrawn from the study. If the subject requests to know which treatment she received, this information will be provided to her. The pregnancy will be

followed for outcome of the mother and child (including any premature terminations) and should be reported to the Sponsor/designee after outcome.

Should the investigator become aware of a pregnancy in the partner of a male study subject who has received investigational product, this should be reported ***within 24 hours of knowledge of the event*** to Sponsor/designee as above. The Sponsor will endeavor to collect follow-up information on such pregnancies provided the partner of the study subject provides consent.

6 STUDY AND DATA MANAGEMENT

6.1 Training of Study Site Personnel

Before the first subject is entered into the study, a MedImmune representative will review and discuss the requirements of the Clinical Study Protocol and related documents with the investigational staff and also train them in any study-specific procedures and system(s) utilized. This will include use of all assessment scales to be used in the study, and the appropriate set-up and use of any study-specific equipment required for evaluation of study-related endpoints.

Attendance at an Investigator Meeting is a requirement wherein key training for assessment of NMO/NMOSD attacks and other study-specific evaluations and assessments will be provided. In the event that study site staff are not able to attend the Investigator Meeting, alternative forums for training will be arranged and documented.

The Principal Investigator will ensure that appropriate training relevant to the study is given to all of these staff, and that any new information relevant to the performance of this study is forwarded to the staff involved.

The Principal Investigator will maintain a record of all individuals involved in the study (medical, nursing and other staff).

6.2 Monitoring of the Study

During the study, a MedImmune representative will have regular contacts with the study site, including visits to:

- Provide information and support to the investigator(s)
- Confirm that facilities remain acceptable, including verification of MRI settings and assessment of environment for ophthalmology examinations
- Confirm that the investigational team is adhering to the protocol, that data are being accurately and timely recorded in the eCRFs, that biological samples are handled in accordance with the Laboratory Manual and that study drug accountability checks are being performed

- Perform source data verification (a comparison of the data in the eCRFs with the subject's medical records at the hospital or practice, and other records relevant to the study) including verification of informed consent of participating subjects. This will require direct access to all original records for each subject (eg, clinic charts)
- Ensure withdrawal of informed consent to the use of the subject's biological samples is reported and biological samples are identified and disposed of/destroyed accordingly, and the action is documented, and reported to the subject.

The MedImmune representative will be available between visits if the investigator(s) or other staff at the patient site needs information and advice about the study conduct.

6.2.1 Source Data

Refer to the Clinical Study Agreement for location of source data.

6.2.1.1 Direct Access to Source Data

The head of the study site and the Principal Investigator/investigator will be expected to cooperate with all monitoring and auditing conducted by the Sponsor as well as inspections by the IRB/IEC or regulatory authorities. All study documents, such as raw data, will be open for direct access to source data at the request of the monitor and the auditor of the Sponsor, the IRB/IEC, or regulatory authorities.

To ensure accuracy and completeness of documentation and to assure that the Principal Investigator has submitted the eCRFs to the Sponsor, the monitor(s) will verify data from the eCRFs against source data before the Principal Investigator signs the eCRFs. If the investigator wishes to amend the submitted eCRFs, the electronic system will document all changes in an electronic audit trail, which will be verified by the study monitor.

6.2.2 Study Agreements

The Principal Investigator at each center should comply with all the terms, conditions, and obligations of the Clinical Study Agreement, or equivalent, for this study. In the event of any inconsistency between this Clinical Study Protocol and the Clinical Study Agreement, the terms of Clinical Study Protocol shall prevail with respect to the conduct of the study and the treatment of subjects and in all other respects, not relating to study conduct or treatment of subjects, the terms of the Clinical Study Agreement shall prevail.

Agreements between MedImmune and the Principal Investigator must be in place before any study-related procedures can take place, or subjects are enrolled.

6.2.3 Archiving of Study Documents

The investigator follows the principles outlined in the Clinical Study Agreement.

Study Files

The Sponsor will provide the Principal Investigator with a file for the organization and retention of all study-related documents. All study documents, including letters from the Sponsor, should be retained in this file by the Principal Investigator. The study monitor will regularly check the file to ensure that all relevant documents have been retained. The contents of this file may be audited/inspected by the Sponsor's auditor, regulatory authorities, or IRB/IEC.

Period of Record Retention

The study site and the Principal Investigator will retain essential documents specified in the ICH GCP (eg, source documents such as medical records, contract, signed consent form). Essential documents should be retained at the study site for at least 15 years following completion of the study, or in accordance with regulatory obligations if longer, and thereafter destroyed only after agreement with the Sponsor. In the event of any inconsistency between the above-mentioned contents and the contract with the study site, the contract shall prevail. If the Sponsor requires the documents to be retained for a longer period, the specific period and method of retention will be separately discussed between the study site and the Sponsor. The Sponsor should notify the head of the study site in writing when the study-related records are no longer needed. The records should be managed by a specific person designated by the head of the study site.

6.3 Study Timetable and End of Study

The end of the study ("study completion") is defined as the date of the last protocol-specified visit/assessment (including telephone contact) for the last subject in the study. This will occur when the last subject completes the 52-week SFP.

An individual subject will be considered to have completed the RCP if the subject was followed through Day 197 or experienced an NMO/NMOSD attack as determined by the AC, regardless of the number of doses the subject received. Additionally, all subjects enrolled in the RCP at time that the 67th AC-determined attack is confirmed, or at the time the Sponsor discontinues enrollment upon recommendation of the independent DMC based on evidence of efficacy and safety, will also be considered as completers, regardless of the number of doses the subject received, provided the subject completes the Day 197/EDV visit. A subject will be considered to have completed the OLP if the subject participated for the duration of the OLP as per Section 3.1.1.4. A subject will be considered to have completed the SFP if the subject was followed for 52 weeks (12 months) after the last dose of investigational product. Subjects will be considered not to have completed a study period if consent was withdrawn, death occurred, or the subject was lost to follow up.

6.3.1 Discontinuation or Suspension of the Study Program

If the Sponsor decides to prematurely terminate or suspend the study, the Principal Investigator/investigator, head of the study site, and regulatory authorities should be given written notification of the reason(s) for the premature termination or suspension of the study.

The Principal Investigator/investigator will immediately notify the subjects of this decision, provide appropriate medical treatment and take necessary measures, and record the treatment or measures taken on the source documents.

6.3.2 Completion of the Study

Upon completion of the study, the Principal Investigator/investigator will notify the head of the study site in writing about the completion of the study as well as the summary of the results in accordance with the rule of the study site. Written notification of the results will be provided to the IRB and the Sponsor.

6.4 Data Management

Data management will be performed by MedImmune Data Management staff according to the Data Management Plan.

Adverse events will be classified according to the terminology of the latest version the Medical Dictionary for Regulatory Activities (MedDRA). Medications will be classified according to the AstraZeneca Drug Dictionary. All coding will be performed by the Medical Coding Team at the MedImmune Data Management Center.

A Web-Based Data Capture system will be used for data collection and query handling. The investigator will ensure that data are recorded on the eCRFs as specified in the study protocol and in accordance with the instructions provided.

The investigator ensures the accuracy, completeness, and timeliness of the data recorded and of the provision of answers to data queries according to the Clinical Study Agreement. The investigator will sign the completed eCRFs. A copy of the completed eCRFs will be archived at the study site.

6.5 Medical Monitor Coverage

Each subject will be provided with contact information for the principal investigator. In addition, each subject will receive a toll-free number intended to provide the subject's physician access to a medical monitor 24 hours a day, 7 days a week in the event of an emergent situation where the subject's health is deemed to be at risk. In this situation, when a subject presents to a medical facility where the treating physician or health care provider requires access to a physician who has knowledge of the investigational product and the clinical study protocol and the Principal Investigator is not available, the treating physician or

health care provider can contact a medical monitor through this system, which is managed by a third party vendor.

7 ETHICAL AND REGULATORY REQUIREMENTS

7.1 Ethical Conduct of the Study

The study will be performed in accordance with ethical principles that have their origin in the Declaration of Helsinki and are consistent with ICH/ GCP, and applicable regulatory requirements. The applicable regulatory requirements in Japan are "Good Clinical Practice for Trials on Drugs (Ministry of Health, Labour, and Welfare [MHLW] Ordinance No. 28, 27 March 1997, partially revised by MHLW Ordinance and their related notifications."

7.2 Subject Data Protection

The ICF will incorporate (or, in some cases, be accompanied by a separate document incorporating) wording that complies with relevant data protection and privacy legislation.

MedImmune will not provide individual genotype results to subjects, any insurance company, any employer, their family members, general physician or any other third party, unless required to do so by law.

Extra precautions are taken to preserve confidentiality and prevent genetic data being linked to the identity of the subject. In exceptional circumstances, however, certain individuals might see both the genetic data and the personal identifiers of a subject. For example, in the case of a medical emergency, a MedImmune Medical Monitor or an investigator might know a subject's identity and also have access to his or her genetic data. Also Regulatory authorities may require access to the relevant files, though the subject's medical information and the genetic files would remain physically separate.

7.3 Ethics and Regulatory Review

An IRB/IEC should approve the final study protocol, including the final version of the ICF and any other written information and/or materials to be provided to the subjects. The investigator will ensure the distribution of these documents to the applicable IRB/IEC, and to the study site staff.

The opinion of the IRB/IEC should be given in writing. The investigator should submit the written approval to MedImmune before enrollment of any subject into the study.

The IRB/IEC should approve all advertising used to recruit subjects for the study.

MedImmune should approve any modifications to the ICF that are needed to meet local requirements.

If required by local regulations, the protocol should be re-approved by the IRB/IEC annually.

Before enrollment of any subject into the study, the final study protocol, including the final version of the ICF, is approved by the national regulatory authority or a notification to the national regulatory authority is done, according to local regulations.

MedImmune (or local country representative) will handle the distribution of any of these documents to the national regulatory authorities.

MedImmune (or local country representative) will provide Regulatory Authorities, IRB/IEC and Principal Investigators with safety updates/reports according to local requirements, including suspected unexpected serious adverse reactions (SUSARs), where relevant.

Each Principal Investigator is responsible for providing the IRB/IEC with reports of any serious and unexpected adverse drug reactions from any other study conducted with the investigational product. MedImmune (or local country representative) will provide this information to the Principal Investigator so that he/she can meet these reporting requirements.

The Principal Investigator is responsible for ensuring that the opinion is sought from the IRB/IEC, with respect to the appropriateness of continuing the study at the study site as per the local IRB/IEC requirements.

A valid contract between the study site and the Sponsor should be signed before the investigator can enroll any subject into the study.

7.3.1 Ethics and Regulatory Review in Japan

In addition, in Japan the head of the study site should submit a notification of direction/determination as well as IRB written approval to the Sponsor and the Principal Investigator before enrollment of any subject into the study.

The Principal Investigator should submit progress reports to the IRB via the head of the study site at the time of any protocol reapproval.

7.4 Informed Consent

The Principal Investigator(s) at each center will:

- Ensure each subject is given full and adequate oral and written information about the nature, purpose, possible risk and benefit of the study
- Ensure each subject is notified that they are free to discontinue from the study at any time
- Ensure that each subject is given the opportunity to ask questions and allowed time to consider the information provided

- Ensure each subject provides signed and dated informed consent before conducting any procedure specifically for the study
- Ensure the original, signed ICF(s) is/are stored in the Investigator's Study File
- Ensure a copy of the signed ICF is given to the subject
- Ensure that any incentives for subjects who participate in the study as well as any provisions for subjects harmed as a consequence of study participation are described in the ICF that is approved by an IRB/IEC.

If any new information about the investigational product becomes available that may influence the subject's decision to continue in the study, the investigator(s) should immediately inform the subject of such information, record this in written form, and confirm with the subject if he/she wishes to continue participating in the study. In addition, if the investigator(s) deems it necessary to revise the ICF, it should be revised immediately (see Section 7.5) and the updated ICF should be explained to the subject, even if the subject(s) has been informed verbally about the new ICF. Written informed consent for the subject to continue participating in the study should be provided separately.

7.5 Changes to the Protocol and Informed Consent Form

Study procedures will not be changed without the mutual agreement of the medical monitor, Principal Investigator, and MedImmune.

If there are any substantial changes to the study protocol, then these changes will be documented in a study protocol amendment and where required in a new version of the study protocol. The amendment is to be approved by the relevant IRB/IEC and if applicable, also the national regulatory authority approval, before implementation. Local requirements are to be followed for revised protocols.

MedImmune will distribute any subsequent amendments and new versions of the protocol to each Principal Investigator(s). For distribution to IRB/IEC see Section 7.3.

If a protocol amendment requires a change to a site's ICF, MedImmune and the site's IRB/IEC are to approve the revised ICF before the revised form is used.

If local regulations require, any administrative change will be communicated to or approved by each IRB/IEC.

7.5.1 Deviation From the Clinical Study Protocol

The investigator(s) must not deviate from or make any changes to the protocol without documented agreement between the Principal Investigator and the Sponsor or IRB/IEC approval, based on deliberations. This shall not apply to cases where the deviation or change is necessary to avoid an immediate hazard to the subjects, for other compelling medical

reasons, or where the changes involve only logistical or administrative aspects of the clinical study (eg, changes to the organization/structure of MedImmune, the name/department of the study site, the address or phone number of the study site or Sponsor, and the job title of the investigator or medical monitor).

The investigator(s) should document any and all deviations from the protocol regardless of the reason(s). Unless the deviation occurred to avoid an immediate hazard to the subjects or for other medically compelling reason(s), the investigator should prepare and submit the records explaining the reason(s) for the deviation(s) to MedImmune and the head of the study site and retain a copy of the records.

The investigator(s) may deviate from or make a change to the protocol without documented agreement between the Principal Investigator and MedImmune or IRB approval only in the event of a medical emergency (eg, to avoid an immediate hazard to the subjects). In such cases, the Principal Investigator must notify MedImmune, the head of the study site, and the IRB as soon as possible about the details of the deviation or change, the reason for the deviation, and a proposed revision in the protocol, if required, to obtain their approval. For study sites in Japan, a certificate of approval by the head of the study site as well as the Sponsor should be obtained via the head of the study site.

7.6 Audits and Inspections

All study data may be subject to a reliability review and onsite GCP inspection by the regulatory authorities.

Authorized representatives of MedImmune, a regulatory authority, or an IRB/IEC may perform audits or inspections at the center, including source data verification. The purpose of an audit or inspection is to systematically and independently examine all study-related activities and documents, to determine whether these activities were conducted, and data were recorded, analyzed, and accurately reported according to the protocol, GCP, ICH guidelines, and any applicable regulatory requirements. The investigator will contact MedImmune immediately if contacted by a regulatory agency about an inspection at the site.

8 REFERENCES

Bar-Or A, Calabresi PA, Arnold D, Markowitz C, Shafer S, Kasper LH, et al. Rituximab in relapsing-remitting multiple sclerosis: a 72-week, open-label, phase I trial. Ann Neurol. 2008 Mar;63(3):395-400.

Bar-Or A, Fawaz L, Fan B, Darlington PJ, Rieger A, Ghorayeb C, et al. Abnormal B-cell cytokine responses a trigger of T-cell-mediated disease in MS? Ann Neurol. 2010 Apr;67(4):452-61.

Bedi GS, Brown AD, Delgado SR, Usmani N, Lam BL, Sheremata WA. Impact of rituximab on relapse rate and disability in neuromyelitis optica. Mult Scler. 2011 Oct;17(10):1225-30.

Bell RA, Waggoner PM, Boyd WM, Akers RE, Yee CE. Clinical grading of relative afferent pupillary defects. Arch Ophthalmol. 1993 Jul;111(7):938-42.

Bretz F, Maurer W, Brannath W, Posch M. A graphical approach to sequentially rejective multiple test procedures. Stat Med. 2009 Feb 15;28(4):586-604.

Briggs R, Robinson S, O'Neill D. Ageism and clinical research. Ir Med J. 2012 Oct;105(9):311-2..

Browning JL. B cells move to centre state: novel opportunities for autoimmune disease treatment. Nat Rev Drug Discov. 2006 Jul;5(7):564-76.

Calabrese LH, Molloy E, Berger J. Sorting out the risks in progressive multifocal leukoencephalopathy. Nat Rev Rheumatol. 2015 Feb;11(2):119-23. doi: 10.1038/nrrheum.2014.167.

Chanson JB, Zéphir H, Collongues N, Outteryck O, Blance F, Fleury M, et al. Evaluation of health-related quality of life, fatigue, and depression in neuromyelitis optica. Eur J Neurol. 2011 Jun;18(6):836-41.

Chihara N, Aranami T, Sato W, Miyazaki Y, Miyake S, Okamoto T, et al. Interleukin 6 signaling promotes anti-aquaporin 4 autoantibody production from plasmablasts in neuromyelitis optica. Proc Natl Acad Sci U S A. 2011 Mar 01;108(9):3701-6.

Costanzi C, Matiello M, Lucchinetti CF, Weinshenker BG, Pittock SJ, Mandrekar J, et al. Azathioprine: tolerability, efficacy, and predictors of benefit in neuromyelitis optica. Neurology. 2011 Aug16;77(7):659-66

Cree BA, Lamb S, Morgan K, Chen A, Waubant E, Genain C. An open label study of the effects of rituximab in neuromyelitis optica. Neurology. 2005 Apr 12;64(7):1270-2.

D'Souza M, Yaldizli O, John R, Vogt DR, Papadopoulou A, Lucassen E, et al. Neurostatus e-Scoring improves consistency of Expanded Disability Status Scale assessments: A proof of concept study. Mult Scler. 2016 Jun 30.

Dumville JC, Hahn S, Miles JNV, Torgerson DJ The use of unequal randomisation ratios in clinical trials: A review. Contemp Clin Trials. 2006 Feb;27(1):1-12.

Farrell B, Godwin J, Richards S, Warlow C. The United Kingdom transient ischaemic attack (UK-TIA) aspirin trial: final results. J Neurol Neurosurg Psychiatry. 1991;54(12):1044-54.

Ghezzi A, Bergamaschi R, Martinelli V, Trojano M, Tola MR, Merelli E, et al. Italian Devic's Study Group (IDESG). Clinical characteristis, course and prognosis of relapsing devic's neuromyelitis optica. J. Neurol. 2004 Jan;251(1):47-52.

Greenberg BM, Graves D, Remington G, Hardeman P, Mann M, Karandikar N, et al. Rituximab dosing and monitoring strategies in neuromyelitis optica patients: creating strategies for therapeutic success. Mult Scler. 2012 Jul;18(7):1022-6.

Hauser SL, Waubant E, Arnold DL, Vollmer T, Antel J, Fox RJ, et al; HERMES Trial Group. B-cell depletion with rituximab in relapsing-remitting multiple sclerosis. N Engl J Med. 2008 Feb 14;358(7):676-88.

Huffstutter JE, Taylor J, Schechtman J, Leszczynski P, Brzosko M, Sedarati F, et al. Single- versus dual-infusion of B-cell-depleting antibody ocrelizumab in rheumatoid arthritis: results from the Phase III FEATURE trial. Int J Clin Rheumatol. 2011;6(6):689-96.

Ip VH, Lau AY, Au LW, Fan FS, Chan AY, Mok VC, et al. Rituximab reduces attacks in Chinese patients with neuromyelitis optica spectrum disorders. J Neurol Sci. 2013. 324:38-9.

Jacob A, Weinshenker BG, Violich I, McLinsky N, Kurpp L, Fox RJ, et al. Treatment of neuromyelitis optica with rituximab: retrospective analysis of 25 patients. Arch Neurol. 2008;65(11):1443-8.

Jacob A, McKeon A, Nakashima I, Sato DK, Elsone L, Fujihara K, de Seze J. Current concept of neuromyelitis optica (NMO) and NMO spectrum disorders. J Neurol Neurosurg Psychiatry. 2013 Aug;84(8):922-30.

Jarius S, Paul F, Franciotta D, Waters P, Zipp F, Hohlfeld R, et al. Mechanisms of disease: aquaporin-4 antibodies in neuromyelitis optica. Nat Clin Pract Neurol. 2008 Apr;4(4):202-14.

Jarius S, Wildemann B. AQP4 antibodies in neuromyelitis optica: diagnostic and pathogenetic relevance. Nat Rev Neurol. 2010 Jul;6(7):383-92.

Jarius S, Ruprecht K, Wildemann B, Kuempfel T, Ringelstein M, Geis C, et al. Contrasting disease patterns in seropositive and seronegative neuromyletis optica: A multicenter study of 175 patients. J Neuroinflammation. 2012 Jan 19;9:14.

Jarius S, Wildemann B. The history of neuromyelitis optica. J Neuroinflammation. 2013 Jan 15;10:8.

Kanamori Y, Nakashima I, Takai Y, Nishiyama S, Kuroda H, Takahashi T, et al. Pain in neuromyelitis optica and its effect on quality of life. Neurology. 2011 Aug:77:652-58.

Kappos L, Li D, Calabresi PA, O'Connor P, Bar-Or A, Barkhof F, et al. Ocrelizumab in relapsing-remitting multiple sclerosis: a phase 2, randomised, placebo-controlled, multicentre trial. Lancet. 2011 Nov 19;378(9805):1779-87.

Kappos L, D'Souza M, Lechner-Scott J, Lienert C. Mult Scler Relat Disord. 2015 May;4(3):182-5.

Kim W, Kim SH, Kim HJ. New insights into neuromyelitis optica. J Clin Neurol. 2011 Sep;7(3):115-27.

Kim SH, Kim W, Li XF, Jung IJ, Kim HJ. Repeated treatment with rituximab based on the assessment of peripheral circulating memory B cells in patients with relapsing neuromyelitis optica over 2 years. Arch Neurol. 2011 Nov;68(11):1412-20.

Kitley J, Leite NI, Nakashima I, Waters P, McNeillis B, Brown R, et al. Prognostic factors and disease course in aquaporin-4 antibody-positive patients with neuromyelitis optica spectrum disorder from the United Kingdom and Japan. Brain. 2012;135:1834-49.

Kitley J, Woodhall M, Waters P, Leite MI, Devenny E, Craig J, et al. Myelin-oligodendrocyte glycoprotein antibodies in adults with a neuromyelitis optica phenotype. Neurology. 2012 Sep 18;79(12):1273-7.

Kurtzke JF. Rating neurologic impairment in multiple sclerosis: an expanded disability status scale (EDSS). Neurology. 1983;33(11):1444-52.

Lan GKK, Hu P, and Proschan MAA. Conditional Power Approach to the Evaluation of Predictive Power. Statistics in Biopharmaceutical Research May 2009, Vol. 1, No. 2.

Mader S, Gredler V, Schanda K, Rostasy K, Dujmovic I, Pfaller K, et al. Complement activating antibodies to myelin oligodendrocyte glycoprotein in neuromyelitis optica and related disorders. J Neuroinflamm. 2011 Dec28;8:184.

Matiello M, Lennon VA, Jacob A, Pittock SJ, Lucchinetti CF, Wingerchuk DM, et al. NMO-IgG predicts the outcome of recurrent optic neuritis. Neurology. 2008;70:2197-2200.

Millen B, Dmitrienko A. Chain procedures: a class of flexible closed testing procedures with clinical trial applications. Stat Biopharm Res. 2011;3:14-30.

Misu T, Fujihara K, Nakashima I, Sato S, Itoyama Y. Intractable hiccup and nausea with periaqueductal lesions in neuromyelitis optica. Neurology. 2005 Nov 8;65(9):1479-82.

Mitsdoerffer M, Kuchroo V, Korn T. Immunology of neuromyelitis optica: a T cell-B cell collaboration. Ann N Y Acad Sci. 2013 Apr;1283:57-66.

National Cancer Institute. Common Terminology Criteria for Adverse Events (CTCAE) Version 4.0. Published May 28, 2009 (v4.03, June 14, 2010). http://evs.nci.nih.gov/ftp1/CTCAE/CTCAE_4.03_2010-06-14_QuickReference_8.5x11.pdf.

Oh J, Levy M. Neuromyelitis optica: an antibody-mediated disorder of the central nervous system. Neurol Res Int. 2012;2012:460825.

Pittock SJ, Lennon VA, McKeon A, Mandrekar J, Weinshenker BG, Lucchinetti CF, O'Toole O, Wingerchuk DM. Eculizumab in AQP4-IgG-positive relapsing neuromyelitis optica spectrum disorders: an open-label pilot study. Lancet Neurol. 2013 Jun;12(6):554-62.

Pittock SJ, Weinshenker BG, Lucchinetti CF, Wingerchuk DM, Corboy JR, Lennon VA. Neuromyelitis optica brain lesions localized at sites of high aquaporin 4 expression. Arch Neurol. 2006 Jul;63(7):964-8

Popescu BF, Lennon VA, Parisi JE, Howe CL, Weigand SD, Cabrera-Gomez JA, et al. Neuromyelitis optica unique area postrema lesions: nausea, vomiting, and pathogenic implications. Neurology. 2011 Apr 5;76(14):1229-37.

Posner K, Brown GK, Stanley B, Brent DA, Yershova KV, Oquendo MA, et al. The Columbia-Suicide Severity Rating Scale: initial validity and internal consistency findings from three multisite studies with adolescents and adults. Am J Psychiatry. 2011. 168:1266-77.

Qian P, Lancia S, Alvarez E, Klawiter C, Cross AH, Naismith RT. Association of neuromyelitis optica with severe and intractable pain. Arch Neurology. 2012;69(11):1482-7.

Sampson HA, Muñoz-Furlong A, Campbell RL, Adkinson NF, Bock SA, Branum A, et al. Second symposium on the definition of and management of anaphylaxis: Summary report- Second National Institute of Allergy and Infectious Disease/Food Allergy and Anaphylaxis Network symposium. J Allergy Clin Immunol. 2006; 1175(3):391-7.

Sellner J, Boggild M, Clanet M, Hintzen RQ, Illes Z, Montalban X, et al. EFNS guidelines on diagnosis and management of neuromyelitis optica. Eur J Neurol. 2010 Aug;17(8):1019-32.

Siegrist CA. Vaccines (Sixth Edition), Chapter 2 Vaccine immunology. 2013.

van Swieten J, Koudstaal P, Visser M, Schouten H, et al. Interobserver agreement for the assessment of handicap stroke patients. Stroke. 1988;19(5):604-7.

Varrin-Doyer M, Spencer CM, Schulze-Topphoff U, Nelson PA, Stroud RM, Cree BA, Zamvil SS. Aquaporin 4-specific T cells in neuromyelitis optica exhibit a Th17 bias and recognize clostridium ABC transporter. Ann Neurol. 2012 Jul;72(1):53-64.

Weinshenker BG, Wingerchuk DM, Vukusic S, Linbo L, Pittock SJ, Lucchinetti CF, et al. Neuromyelitis optica IgG predicts relapse after longitudinally extensive transverse myelitis. Ann Neurol. 2006 Mar;59(3):566-9.

Wingerchuk DM, Hoogancamp WF, O'Brien PC, Weinshenker BG. The clinical course of neuromyelitis optica (Devic's syndrome). Neurology 1999 Sep 22;53(5):1107-14.

Wingerchuk DM, Lennon VA, Pittock SJ, Lucchinetti CF, Weinshenker BG. Revised diagnostic criteria for neuromyelitis optica. Neurology. 2006. 66:1485-9.

Wingerchuk DM, Lennon VA, Lucchinetti CF, Pittock SJ, Weinshenker BG. The spectrum of neuromyelitis optica. Lancet Neurol. 2007 Sep;6(9):805-15.

9 CHANGES TO THE PROTOCOL

All changes described below have been incorporated into the current version of the protocol.

Protocol Amendment 6; 11Oct2018

The primary purpose of this amendment was to introduce language related to changes in the study resulting from the Sponsor's decision to accept recommendations from the independent DMC, based on evidence of efficacy and safety, to stop study enrollment and allow subjects in the RCP at that time the option to enter the OLP. In addition, minor copy-editing and formatting issues were corrected throughout the document. Changes to the protocol reflecting the change above were made throughout the protocol (Synopsis, Sections 3.1, 4.2, 4.4, 4.5, 4.8, and 6.3, and Table 2).

Protocol Amendment 5; 16Jul2018

Text revisions resulting from this amendment are incorporated in the body of Protocol Amendment 5. This amendment incorporates numerous administrative and personnel changes, corrects existing errors, and updates definitions and processes for safety reporting. Table and figure numbering was changed to sequential numbering to align with the new in-house authoring style. Minor copy editing and formatting were corrected throughout the document. Major changes to the protocol were as follows:

1. Title Page: The medical monitor was changed from Armando Flor, MD to John Ratchford, MD, along with contact information.
2. Synopsis and Section 3.1.1 (Overview), Section 3.1.1.1 (Screening Period), Section 4.1.1 (Number of Subjects), Section 4.2.1 (Enrollment/Screening Period), and Section 4.8.2 (Sample Size and Power Calculations): Text was added to these sections to clarify that enrollment of subjects into the study will stop when a total of 67 AC-determined NMOSD attacks have occurred or when 252 subjects have been randomized ***and dosed***, whichever occurs first, to account for subjects that are randomized but do not receive investigational product.
3. Section 4.1.2 (Inclusion Criteria), Table 4 (Highly Effective Methods of Contraception): Removed incorrect abbreviation for intrauterine hormone-releasing system and duplication of "injectable".
4. Section 4.1.3 (Exclusion Criteria): Exclusion Criterion 12 was revised to add the stipulation that rituximab treatment with 6 months prior to screening is not exclusionary if a subject has B-cell counts above the LLN, since the reason for excluding rituximab within the prior 6 months is the possibility of low B cells. Exclusion Criterion19 was revised to read, "or" instead of "and" with reference to therapies for concurrent autoimmune disease.

5 Section 4.2.1 (Enrollment/Screening Period), Table 5 (Schedule of Screening Procedures): Order of footnote references was corrected.

6 Section 4.2.2.1 (Randomized-controlled Treatment Period), Table 6 (Schedule of Randomized-controlled Treatment Period Study Procedures); Section 4.2.2.2 (Open-label Period), Table 7 (Schedule of Open-label Period Study Procedures); and Section 4.5.1.2 (Treatment Administration), Randomized-controlled Period: The window for the Day 15 visit (for both RCP and OLP) was changed from + 3 days to ± 3 days, which provides scheduling flexibility to the sites and is anticipated to have no consequence on the pharmacodynamic effect or safety of the drug.

7 Section 4.2.2.2 (Open-label Period), Table 7 (Schedule of Open-label Period Study Procedures): Urine pregnancy tests for Weeks 13 and 39/then Q26W visits were removed as these are not required because the subjects are not dosed at these visits.

8 Section 4.3.1.2 (Neuroaxis Magnetic Resonance Imaging Scan), MRI for All Subjects: Text was added to stipulate that brain MRI review by the Principal Investigator is acceptable in the event of a safety alert of new brain lesion(s) from the central MRI reader.

9 Section 4.3.1.4 (Expanded Disability Status Scale and Functional Systems Scores): Text was revised to remove “face-to-face” as not all training is conducted person-to-person.

10 Section 4.7.2 (Prohibited Concomitant Medications): Text was added that low-dose steroid may be permissible if discussed and agreed with the medical monitor, as in some situations low-dose steroid administration will have no impact on the NMO disease course. Also added prohibition of any other immunosuppressant medication (beyond those already named) unless discussed and approved by the medical monitor.

11 Section 5.5 (Reporting of Serious Adverse Events): Information for SAE reporting was updated following a change of service provider.

12 Section 5.6.1 (Overdose): The definition of overdose was expanded to include receipt of investigational product at a time/visit not specified in the protocol; information for overdose reporting was updated.

13 Section 5.6.2 (Hepatic Function Abnormality): Text was revised to specify that all occurrences of Hy’s Law are to be considered SAEs and are to be reported to the Sponsor according to SAE reporting procedures.

14 Section 5.6.3 (Progressive Multifocal Leukoencephalopathy): Process and information for reporting possible cases of PML were updated.

15 Section 5.6.4 (Pregnancy): Information for pregnancy reporting was updated.

16 Appendix 1 (Signatures): Text was updated to remove Jorn Drappa, MD and replace with Eliezer Katz, MD FACS for sponsor signature.

Protocol Amendment 4; 08Mar2017

Text revisions resulting from this amendment are incorporated in the body of Protocol Amendment 4. The primary reasons for changes included in Amendment 4 are the removal of the sample size reassessment and the inclusion of clear guidance that stopping enrollment is based on the occurrence of 67 AC-determined NMOSD attacks or when 252 subjects have been enrolled. Minor edits were also addressed.

1 Protocol Synopsis: Revisions were made to the synopsis to reflect changes in the body of the protocol amendment.

2 Section 1.4 (Summary of Clinical Experience): Text was updated that Study CD-IA-MEDI-551-1102 has now been completed. Additionally, text was added that PML has been reported in the setting of B-cell depleting therapies and other immunosuppressive regimens and is a potential risk of treatment with MEDI-551. A subject in Study CD-IA-MEDI-551-1155 developed altered mental state, seizures, and new brain lesions that on brain MRI raised suspicion of PML. CSF testing for JCV was performed, with inconclusive results (two negative results in reference laboratories and one positive result in a local labarotory). Brain tissue was not available for examination. The subject died from cardiopulmonary complications. Based on all available data, the differential diagnosis included ADEM, atypical NMOSD attack, and PML. No conclusive diagnosis could be made. This text was added to provide investigators and others with the latest and most complete information regarding this potential safety risk.

3 Section 3.1.1 (Overview): Text was changed to a maximum of 252 subjects with NMO/NMOSD will be randomized in a 3:1 ratio to receive IV MEDI-551 (300 mg at Day 1 and 300 mg at Day 15) or placebo for a period of 197 days (RCP). Text was added that enrollment of subjects into the study will stop when a total of 67 AC-determined NMOSD attacks occur or when 252 subjects have been randomized, whichever occurs first. For this and all related changes in this amendment, the removal of the interim sample size reassessment resulted from discussion with the FDA regarding the timing of the sample size reassessment as related to the futility analysis. As the study progresses it becomes possible that the futility analysis will occur prior to the sample size reassessment, which would not allow for the performing of the sample size reassessment. Therefore an agreement was reached with the FDA for the removal of the sample size reassessment from the protocol and increasing the sample size to 252 subjects (or until 67 AC-determined NMOSD attacks have occurred).

4 Section 3.1.1.1 (Screening Period): Text was added that subjects who are in screening at the time that 252 subjects are randomized will not be randomized into the study. This condition was added to prevent additional subject exposure to potential risks of the study beyond that required by the protocol objectives.

5 Figure 3.1.1-1 (Study Flow Diagram): The study flow diagram was revised to update the sample size to 252 subjects.

6 Section 3.1.1.2 (Randomization [Day 1]): The maximum number of subjects to be randomized was changed from 212 to 252.

7 Section 3.1.1.3 (Randomized-controlled Period [Day 1 to Day 197]): New text was added to clarify that subjects who are in the RCP when a total of 67 AC-determined NMOSD attacks have occurred will discontinue the RCP within 14 days and be given the option to enroll into the OLP. Management of subjects who do not discontinue within 14 days should be discussed with the medical monitor. This condition was added to prevent additional subject exposure to the potential risks of the study beyond that required by the protocol objectives.

8 Section 3.1.1.4 (Open-label Period): Text was minimally revised for Criterion #2 and Criterion #3 was added that subjects will be given the option to enter the OLP if they are in the RCP at the time 67 AC-determined attacks have occurred. Text was revised to clarify that subjects who discontinue from the RCP for reasons other than an NMO/NMOSD attack or the occurrence of 67 AC-determined attacks will not be eligible for the OLP.

9 Section 3.1.2 (Treatment Regimen) / Open-label Period: Text was added to clarify that dosing of subjects enrolling into the OLP following an adjudicated NMO/NMOSD attack, and following the occurrence of the 67th adjudicated NMO/NMOSD attack, will be identical to the OLP dosing regimen.

10 Section 4.1.1 (Number of Subjects): New text was added that the study is event driven where the primary objective is based on a total of 67 AC-determined NMO/NMOSD attacks. Because the sample size reassessment was removed from the protocol a calculated assessment was performed to determine the number of patients needed to be randomized to reach 67 AC-adjudicated NMOSD attacks. As the rate of NMO/NMOSD attacks is not established in the literature, the attack rate was analyzed based on masked data from the first 78 subjects who completed the RCP in this study. The attack status (attack/no attack) of the 78 subjects was randomly sampled with different attack rates, which gave an estimate of the number of attacks for the total sample size. This simulation process was repeated 10,000 times to give a distribution of the number of attacks for the total sample size, from which the probability of observing at least 67 attacks could be estimated. Based on the 78 completed subjects, this procedure showed that with a sample size of 227 there is a 50% probability of reaching the required 67 AC-determined attacks and with 252 subjects there is a 90% probability of reaching the required 67 AC-determined attacks. The sample size of 252 subjects was selected to give a high degree of confidence that 67 attacks will be observed in this study. Enrollment of subjects into the study will stop when a total of 67 AC-determined NMOSD attacks occur or when 252 subjects have been randomized, whichever occurs first.

11 Section 4.1.2 (Inclusion Criteria): Criteria #6b, 7, and 8 were revised to add text and new table (Table 4.1.2-1, Highly Effective Methods of Contraception) to emphasize that nonsterilized sexually active males with female partners of childbearing potential and

sterilized males must use a condom plus spermicide. It is strongly recommended that female partners of male subjects to also use a highly effective method of contraception throughout this period.

12 Section 4.1.7 (Replacement of Subjects): Text mentioning sample size reassessment was removed as this will not be conducted.

13 Section 4.2.1 (Enrollment/Screening Period): Text was added for clarification and consistency with other sections that subjects undergoing screening at the time 252 subjects are randomized may not be randomized into the study and the management of these subjects should be discussed with the medical monitor. Similarly, subjects in screening at the time of the 67th AC-determined attack will not be randomized. The screening period may be extended to allow for repeat procedures.

14 Section 4.2.2.1 (Randomized-controlled Treatment Period): Text was added to clarify that at the time 67 AC-determined attacks have occurred, the sponsor will notify all sites and the sites will notify subjects ongoing in the RCP that they must discontinue within 14 days and should be given the option to enroll into the OLP. Procedures should be conducted as presented in the Schedule of Randomized-controlled Treatment Period Procedures for the Day 197/ EDV; however, subjects who have had an MRI in the last 3 months need NOT repeat the MRI to reduce the burden on the subjects. Subjects who decide to enroll into the OLP will follow procedures of the OLP as presented in Table 4.2.2.2-1 (Schedule of Open-label Period Study Procedures). This time limit on discontinuation of the RCP is to ensure the timely completion of the study and to prevent any delay in continued receipt of study medication in the OLP, if subjects choose to enroll.

15 Section 4.2.2.2 (Open-label Period): Text was added to clarify that subjects who are in the RCP at the time of the 67th AC-determined attack and want to enroll in the OLP must do so within 2 weeks of the occurrence of the 67th attack. This time limit on discontinuation of the RCP is to ensure the timely completion of the study and to prevent any delay in continued receipt of study medication in the OLP, if subjects choose to enroll.

16 Table 4.2.2.2-1 (Schedule of Open-label Period Study Procedures): Text relating to the follow-up telephone calls has been removed as these calls are not necessary. The purpose of these calls was to mitigate against the risks of placebo use. As there is no placebo in the OLP these calls place an unnecessary burden on sites and subjects. Footnote “b” was updated to delete text that PK samples would be collected at selected sites only.

17 Section 4.3.1.1 (Identification, Assessment, and Adjudication of an NMO/NMOSD Attack): Text amended to remove the need for follow-up telephone calls to subjects during the OLP.

18 Section 4.3.1.2 (Neuroaxis Magnetic Resonance Imaging Scan): Text added to state that subjects who discontinue the RCP at the time of the 67th AC-determined attack need not

have an MRI repeated if the last one was done within 3 months to reduce the burden on the subjects

19 Section 4.3.3.2 (Physical and Neurological Examination): Text was added for clarification that additional neurological exams may be conducted according to local practice.

20 Section 4.3.5 (Modified Rankin Scale): Text indicating that the Principal Investigator will evaluate the mRS was removed as this assessment may be done by any trained and designated member of the site team.

21 Section 4.3.6 (Clinical Laboratory Tests) / Other Safety Tests: Bullet point and text were added to further clarify that only in cases of suspected PML, a CSF sample for JCV DNA PCR will be collected and frozen immediately.

22 Section 4.3.9.1 (Exploratory Investigations) / Serum for Exploratory Biomarker Studies: The last sentence in the subsection was revised to correct that the presence of autoantibodies to antigens other than AQP4 may be explored throughout the study. Additionally, the subsection, Blood for Peripheral Blood Mononuclear Cell Isolation for T-cell Evaluation was removed as it is not feasible to manage these samples within the required stability window.

23 Table 4.3.10-1 (Estimate of Blood Volume to be Collected): Blood volumes for PBMC sample collection was removed from table and footnotes as PBMC will not be collected.

24 Section 4.5.1.2 (Treatment Administration) / Open-label Period: Text was revised for consistency with other sections that subjects who are in the RCP at the time of 67th AC-determined attack is achieved must enter the OLP within two weeks of the occurrence of the 67th attack, unless there is a compelling reason that is discussed and agreed with the medical monitor, in which case a short extension may be granted.

25 Section 4.7.1.1 (Immunosuppressive Medications During the Screening Period): Text was removed regarding the treatment of an NMO/NMOSD attack in screening as this is no longer consistent with Section 4.2.1, which requires that subjects experiencing an attack in the screening period be screen failures.

26 Section 4.8.2 (Sample Size and Power Calculations): Text revised for consistency with other sections that a maximum number of 252 subjects will be randomized. The sample size is based on a masked calculation of the attack rate for the first 78 subjects to complete the RCP and a simulation based on this attack rate that indicates a > 90% probability of achieving the required 67 AC-determined attacks with 252 subjects. Text mentioning masked sample size reassessment was removed as this will not be done.

27 Section 4.8.3.1 (Primary Efficacy Analysis): Text was revised to clarify and for consistency with other sections that the data cutoff date for the primary analysis will be when the last subject completes the discontinuation visit following the 67th AC-determined attack, or after all subjects complete the RCP if 67 AC-determined

attacks do not occur. Subjects who are in the RCP when a total of 67 AC-determined NMOSD attacks have occurred will discontinue the RCP within 14 days.

28 Section 4.8.10.1 (Masked Sample Size Reassessment): This section was removed as the reassessment will not be conducted.

29 Section 5.3 (Definition of Adverse Events of Special Interest): Text was added and revised to clarify that 2 AEs will be considered AESIs for this study: 1) hepatic function abnormality meeting the definition of Hy's Law, and 2) PML.

30 Section 5.6.3 (Progressive Multifocal Leukoencephalopathy): Section and text were added indicating that PML is an AESI and a potential risk for MEDI-551 and that confirmation for PML diagnosis requires the presence of JVC in CSF or brain tissue biopsy sample, any suspected case of PML should be immediately reported to the sponsor and discussed with the medical monitor, and the medical monitor should be contacted for instruction on proper handling of subject samples and analysis in the case of potential PML. Sample transfer procedures are critical to ensure accurate test results, and the sponsor wishes to be involved in these processes to ensure appropriate procedures are followed.

31 Section 6.3 (Study Timetable and End of Study): Text was added to clarify subject completion of the RCP. Any subject in the RCP at the time of the 67th AC-determined attack will be considered as having completed the RCP regardless of the doses the subject received.

32 Appendix 9 (Sample Size Calculation): Text was added to align with changes in Section 4.8.2 and provide explanation that the originally planned masked interim sample size reassessment was removed from the protocol as a result of a discussion with the FDA regarding the timing of the sample size reassessment as related to the futility analysis. As the study progresses it becomes possible that the futility analysis will occur prior to the sample size reassessment, which would not allow the sample size reassessment to be performed. Therefore an agreement was reached with the FDA that the sample size reassessment would be removed from the protocol. The sample size was also increased to a maximum number of 252 randomized subjects to provide a high degree of confidence that 67 NMO/NMOSD attacks will be observed in the study.

33 Appendix 11 (Details of Masked Sample Size Reassessment): Text and table (Table 9-5, Masked Sample Size Reassessment Examples [60% Treatment Effect]) were removed as this reassessment will not be conducted.

<u>Protocol Amendment 3; 18Oct2016</u>

Text revisions resulting from this amendment are incorporated in the body of Protocol Amendment 3. These changes were primarily made to provide additional clarification.

1 Section 4.1.3 (Exclusion Criteria), Criterion 10g: Text was clarified to make it clear that the criterion refers to CD19+ B cell counts.

2 Section 4.2.1 (Enrollment/Screening Period) and Section 4.3.1.2 (Neuroaxis Magnetic Resonance Imaging Scan): Text added to allow the use of a previous MRI scan as part of the screening assessment for subjects that are being re-screened to prevent those subjects having to go through a lengthy and uncomfortable procedure if the previous MRI was done in the last 3 months. The sponsor has consulted with an expert on the matter and confirmed that MRI can be used for up to 3 months as the basis for the evaluation of eligibility, therefore this change which will only apply to a minimal number of subjects, will have not impact on the integrity of the protocol.

3 Section 4.2.1 (Enrollment/Screening Period): The opportunity for a subject to undergo one additional re-sceen has been introduced to ensure that a subject is not unreasonably excluded for reasons that would not prevent the subject from safely participating in the study or that would not impact the integrity of the study. This will not include subjects that have failed to meet entry criteria twice rather subjects that have not been able to enter for logistical reasons such as lab results not reported in the screening window. As such there is no impact on the subject population.

4 Section 4.3.1.3 (Independent Ophthalmology Assessments): wording related to the order of assessments has been removed to ensure consistency with changes made in protocol amendment 2 as the deletion of this text was overlooked in error when amendment 2 was created.

Low and High Contrast Visual Acuity: following advice from expert ophthalmologists, clarification on when the high and low contrast visual acuity assessments should stop has been added to the protocol. The clarification is consistent with standard ophthalmology practice.

5 Section 4.3.1.4 (Expanded Disability Status Scale and Functional Systems Scores): Neurostatus as the name of the EDSS vendor was removed from the text as this group is no longer engaged with this study.

Wording related to the order of assessments ha sbeen removed to ensure consistency with changes made in protocol amendment 2 as deletion of this text was overlooked in error when the amendment was created.

6 Section 4.3.6 (Clinical Laboratory Tests): Coagulation parameters were added to the hematology panel as these are currently being evaluated as part of this panel and were omitted in error.

7 Table 4.3.10.1 (Estimate of Blood Volume to be Collected): blood volumes for the Attack Follow-up Visit and total blood volume were corrected.

8 Table 4.5.1-1 (Identification of Investigational Products): The amount of polysorbate 80 in the investigational product was corrected to 0.01% (w/v).

9 Section 4.8.3.1 (Primary Analyses) and Section 4.8.3.2 (Additional Analyses of the Primary Endpoint): During discussions with the FDA the window in which attacks are assessed was discussed. It is essential for the quality of the data and patient safety that potential attacks are assessed as quickly as possible. The protocol therefore specifies a 3 day window for subjects to come into the site and a further 5 days for assessments to be completed. However the sponsor and the FDA agreed that a wider window will apply for the statisticial analysis of the primary endpoint analysis to prevent valid attacks being excluded from the analysis. A sensitivity analysis to assess the impact of the loss of events outside of the identified assessment window was also added. The operational windows for management of subjects with potential attacks has not changed and there is no impact on the management of the subjects.

10 Section 4.8.10.2 (Futility Assessment) and Appendix 10 (Details of Futility Analysis): Text was clarified to provide further information on how the futility assessment will be performed.

11 Section 8 (References) additional references included in this amendment have been added.

<u>Protocol Amendment 2; 10Dec2015</u>

Text revisions resulting from this amendment are incorporated in the body of Protocol Amendment 2. The majority of these changes were primarily made for clarification to text and tables.

1 Protocol Synopsis and Section 3.1.1.1 (Screening Period), Section 3.2.3 (Rationale for Study Population), Section 4.1.2 (Inclusion Criteria), Criterion #3b, and Section 4.2.1 (Enrollment/Screening Period): Text was amended to clarify that eligibility of AQP4-IgG seronegative subjects will be determined by an independent eligibility committee to reflect actual practice.

2 Protocol Synopsis, Section 3.1.1 (Overview), 3.1.1.4 (Open-label Period), 4.2.2.2 (Open-label Period), and Table 3.1.2-2 (Open-label Period Treatment Regimen), footnote “a”: Text was added or revised to clarify that subjects may exit the OLP at any time for any reason, including seeking alternative treatment options; this text modification was made in response to European Medicines Agency scientific advice.

3 Protocol Synopsis and Section 3.1.1.1 (Randomized-controlled Period (Day 1 to Day 197): Text was revised to clarify tht assessment of new symptoms or worsening of exisiting symptoms should be completed within 5 days to determine if an attack has occurred.

4 Protocol Synopsis and Section 4.1.1 (Number of Subjects), Section 4.1.7 (Replacement of Subjects), and Section 4.8.2 (Sample Size and Power Calculations): Text was revised to clarify that the study is event driven and enrollment of subjects will stop when a total

of 67 AC-determined NMO/NMOSD attacks occur. The planned number of subjects to be enrolled is 212; however, based on sample size reassessment the number maybe increased to a maximum of 252 subjects.

5 Protocol Synopsis, Section 4.8.10.1 (Masked Sample Size Reassessment), and Appendix 11 (Details of Masked Sample Size Reassessment): Text was added for clarification that the study population may be increased up to 252 subjects if the ratio of the cumulative number of AC-determined NMO/NMOSD attacks to subject ratio is approximately 27% or less.

6 Section 1.4 (Summary of Clinical Experience): Text was revised to update the current statuses of clinical studies to align with the updated IB.

7 Section 3.1.1.2 (Randomization [Day 1]): Text was added to clarify that Day 1 is to occur within 28 days of the start of the screening period.

8 Section 3.1.1.5 (Safety Follow-Up Period): Text was added to align with the Czech Republic Local Protocol Amendment 1 as this is applicable globally; "During the SFP, a subject may receive standard treatment for their NMO/NMOSD at the discretion of the investigator."

9 Section 3.2.4.2 (Secondary Endpoints): Subsection for Vaccine Titers and reference were added to provide the rationale for measuring anti-tetanus vaccine titers as one of the laboratory measures for the safety and tolerability of MEDI-551.

10 Section 4.1.2 (Inclusion Criteria), Criteria #3b, #4, #6, #7 and #8: Criterion #3b was revised to clarify that data from AQP4-IgG subjects will be reviewed by an independent eligibility committee to confirm eligibility. Criterion #4 was revised to remove wording related to relapses in the screening period since these subjects should now be screen failed. Also, the time that subjects must be stable or improving prior to randomization was changed from at least 2 weeks to at least 4 weeks to ensure that the subject is appropriately managed and ensure there is less chance of an attack continuing. Criteria #6 and #7 were added to align with local requirements for the Czech Republic. Criterion #8 spelling was corrected to clarify that the criterion applies to males without appropriate post-vasectomy documentation.

11 Section 4.1.3 (Exclusion Criteria), Criteria #3, #10g, #12, #23a, and #24: The text for Criterion #3 was revised to remove "at risk of developing nephrogenic systemic fibrosis after receiving gadolinium (Gd)-containing contrast agents" as all subjects with a score of less than 60 mL/minute may be at risk for developing nephrogenic systemic fibrosis and will be excluded. Criterion #10g was added to clarify that all subjects with B cells below the LLN are excluded as Criterion #12 applies only to subjects on rituximab; however, the same safety concerns apply regardless of prior medications. Criterion #12 was modified to add text to exclude subjects using rituximab in the 6 months prior to screening as experience to date shows that B cells have not usually recovered to the LLN within this time frame. The text reference to B cells was removed as this is now covered

by Criterion #10g. Criterion #23a was modified to lower the total Ig value that is exclusionary as it was unnecessarily restrictive. The LLNs of the most abundant Igs (A, G, M) total to approximately 665 mg/dL, a further 10% under this does not constitute hypoglobulinemia. Criterion #24 text was added to clarify how hepatitis B and hepatitis C serology will be managed. In cases where the surface antigen is negative and the core antibody is positive, the presence of surface antibody will permit entry as it signifies that the subject is immune to hepatitis B, where the absence of surface antibody signifies that the subject is at risk of having hepatitis B.

12 Section 4.1.4.1 (Enrollment): Text was revised to clarify that re-screened subjects will receive a new SID number to reflect actual practice with the INRS/IWRS.

13 Section 4.1.5 (Withdrawal from the Study): Text was added to clarify that subjects at sites in the Czech Republic may discontinue investigational product following an EDV and enter the SFP if the subject agrees to enter the SFP, unless they are lost to follow up or enrolled in another clinical study.

14 Section 4.2.1 (Enrollment/Screening Period): Text in this section was revised for the following reasons: 1) to allow procedures after consent is signed to be conducted in any order to give greater flexibility in scheduling the large number of study procedures; 2) to remove text to extend the screening period in the event that a subject has an NMO attack to ensure that subjects are appropriately stabilized prior to entry to the study; 3) to clarify that any subject who cannot be assessed in the 28-day screening period or experiences an NMO/NMOSD relapse, must be withdrawn and screen failed, and that any subject who is screen failed may be rescreened one further time, unless their participation would exceed the 80:20 ration; 4) to clarify that blood samples for serum chemistry and hematology must be redrawn in the event that screening is extended to ensure that safety laboratory results are up-to-date in the 28 days prior to randomization.

15 Table 4.2.1-1 (Schedule of Screening Procedures): The table was modified for the following reasons: 1) blood samples for Total Ig, IgM, IgG, IgA, and IgE were added to the assessments to align with Administrative Change 1; 2) the ophthalmology assessment was revised to clarify that this should be an independent assessment; and 3) Footnote "d" was added to clarify that if the EDSS and C-SSRS are conducted by the same person that the EDSS should be performed first. This is the case at some sites and conducting the assessments in this same order will eliminate potential EDSS rater bias resulting from information gathered during the C-SSRS assessment.

16 Section 4.2.1.1 (Eligibility Committee): New section was added to clarify that an independent Eligibility Committee will determine if subjects who are AQP4-IgG negative meet the Wingerchuk et al, 2006 criteria for NMO.

17 Section 4.2.2.1 (Randomized-controlled Treatment Period): Text was revised for the following reasons: 1) to clarify that the randomization on Day 1 must occur within 28 days of the start of the screening period, unless screening is extended; 2) to explain

the principle for ordering procedures to facilitate efficient conduct of the visit, including the conduct of the visit over 2 days; and 3) to remove text regarding an NMO attack at the randomization visit as it is now covered in the previous section.

18 Table 4.2.2.1-1 (Schedule of Randomized-controlled Treatment Period Study Procedures): The table was modified for the following reasons: 1) Ig samples were removed for Day 1, Week 0 assessments to align with Administrative Change 1; 2) the ophthalmology assessment was revised to clarify that this should be an independent assessment; 3) footnote “e” was revised to claify that the subject should be treated for the attack as required, screen failed, and then reassessed; 4) footnote ‘h’ was added to clarify that if the EDSS and C-SSRS are conducted by the same person the EDSS should be done first to eliminate the possibility of bias; 5) footnote “i” was added to clarify that the randomization visit may be split over 2 days to accommodate the number of study procedures; and 6) footnote “j”was added to clarify that tetanus vaccine titers will be tested at Day 1 for all subjects and that with negative results will not continue to be tested but those who test positive will continue to be tested at specified titer time points; and 7) footnote “k” was added to clarify that if the second dose of investigational product is delayed due to medical/safety reasons, dosing must be discussed with the medical monitor prior to administration of investigational product to ensure all subjects have the same opportunity to receive investigational product.

19 Section 4.2.2.2 (Open-label Period): Text was revised or added for the following reasons: 1) explain the principle for ordering procedures to facilitate efficient conduct of the visit; 2) to allow some flexibility in the time for subjects completing the RCP without an NMO/NMOSD attack to enter the OLP in the event that there is a safety issue; and 3) to increase the time period in which subjects who experience an AC-determined NMO/NMOSD attack have to enter the OLP to ensure that subjects are not unfairly excluded in the event that it may require a number of weeks for stabilization with rescue medication. Managing acute attacks with rescue medications is expected to prevent further NMO/NMOSD attacks.

20 Table 4.2.2.2-1 (Schedule of Open-label Period Study Procedures): The table was modified for the following reasons: 1) footnote “d” was revised to clarify that if any part of the neuroaxis MRI was not done at the time of the Assessment Visit, it should be conducted at Day 1 of the OLP, otherwise it is not required; 2) footnote “g” was added to clarify that investigational product will not be given if the subject is undergoing an EDV; 3) footnote “h” was added to clarify that if the EDSS and C-SSRS are conducted by the same person the EDSS should be performed first to eliminate potential bias; 4) footnote “i” was added to clarify that in the event that the second dose of investigational product is delayed due to medical/safety reasons, dosing must be discussed with the medical monitor prior to administration of investigational product to ensure that all subjects have the same opportunity to receive investigational product; 5) footnote ‘j’was added to clarify that tetanus vaccine titers will only be tested in the OLP for subjects who tested

positive at Day 1 of the RCP; and 6) to add +28 days to AC determination to the Day 1 visit window for clarification.

21 Section 4.2.3 (Study Procedures for Assessment Visit): Text was revised or added for the following reasons: 1) to clarify that procedures to determine whether symptoms are related to NMO/NMOSD should be conducted first, followed by procedures for determination of an NMO/NMOSD attack; 2) to clarify that all assessments are to be documented with time and date and that the order of the assessments should be determined by the nature of the suspected attack (eg, EDSS before Independent ophthalmology in the case of myelitis symptoms; 3) to clarify that an MRI of all domains may be performed as part of the Assessment Visit and can be done at any time during the Assessment Visit, and that the order of the MRI domains can be determined by the nature of the suspected attack; 4) to clarify that MRI images/study report should not be reviewed by the Principal Investigator unless a specific criterion for an attack requires review of the MRI and in such cases the review of the MRI image must be done after the review of all relevant clinical assessment data is complete; 5) to clarify that assessments should be concluded as soon as possible, but should not extend beyond 4 days from Day 1 of the Assessment Visit; and 6) to clarify that if the investigator determines that new or worsening symptoms are not related to NMO, then the independent assessments will not be required.

22 Table 4.2.3-1 (Schedule of Study Procedures for the Assessment Visit): The following modifications were made to the table: 1) assessments for procedures to determine an NMO/NMOSD attack were moved below the laboratory assessments and neuroaxis MRI scan was moved to be included in these assessments; 2) a new assessment was added to review data generated against protocol-defined attack criteria and create a narrative of the Assessment Visit findings; 3) footnote "b" was revised to change the original text to "the MRIimage/report is only reviewed by the Principal Investigator in the event it is required by the protocol-defined attack criteria; 4) footnote "c" was added to clarify that if the EDSS and C-SSRS are conducted by the same person that the EDSS must be performed first to eliminate the potential for bias; 3) former footnote "c" was removed to avoid confusion; as a full MRI will be performed regardless of the attack criteria.

23 Section 4.2.4 (Managing Subjects With Worsening Attacks): New section was added to describe how to manage and capture data from subjects who experience worsening of an investigator-diagnosed attack. These unscheduled data will provide a narrative of the subject's attacks if they worsen after diagnosis.

24 Section 4.2.5 (Study Procedures for Attack Follow-up Visit): Text in this section was revised or added for the following reasons: 1) to move text to beginning of the paragraph for purpose of highlighing that subjects who experience an NMO/NMOSD attack requiring rescue treatment regardless of the outcome of the AC review will undergo an Attack Follow-up Visit; and 2) text was added to clarify the patient-reported outcomes

should be done first followed by all othr assessments/procedures in an order to be determined by the sites.

25 Table 4.2.5-1 (Schedule of Study Procedures for the Attack Follow-up Visit): The following modifications were made to the table: 1) The order of assessments for SF-36 Health Survey, C-SSRS, indpendent ophthalmology examination, and independent EDSS/FSS were rearranged; 2) footnote “c” was added to clarify that if the EDSS and C-SSRS are conducted by the same person that the EDSS must be performed first to eliminate the potential for bias.

26 Section 4.2.6 (Safety Follow-up Period): Text was revised to remove reference to the order in which study assessments are to be performed as the order has no impact on any of the assessments in the SFP.

27 Section 4.3.1.1 (Identification, Assessment, and Adjudication of an NMO/NMOSD Attack), Overview: Text was revised to clarify that following the start of the Assessment Visit, the procedures required to diagnose an NMO/NMOSD attack must be completed within a total of 5 days and to add greater clarity that there are 2 main outcomes to the Assessment Visit, which are outlined.

28 Figure 4.3.1.1-1 (Flow Chart of Assessment and Diagnosis of an NMO/NMOSD Attack): The flow diagram was updated to better reflect required process; ie, that an MRI is always required at an Assessment Visit; however, review of the images is criterion dependent.

29 Section 4.3.1.1 (Identification, Assessment, and Adjudication of an NMO/NMOSD Attack), Telephone Calls to Subjects: Text was modified to clarify that the telephone calls made to subjects will be every 2 weeks and not twice a week (bi-weekly).

30 Section 4.3.1.1 (Identification, Assessment, and Adjudication of an NMO/NMOSD Attack), Assessment Visit: Text was revised for the following reasons: 1) to clarify that data for the assessment of symptoms that were determined as not related to NMO/NMOSD will be sent to the AC for non-real-time review; 2) to clarify the the date and time of clinical assessments conducted by independent assessors must be documented and the order of the assessments may be driven by the nature of the suspected attack; 3) to clarify that an MRI of all domains should be performed as part of an Assessment Visit and can be done at any time during the Assessment Visit, and that the order of the MRI domains can be determined by the suspected NMO/NMOSD attack; 4) to clarify that MRI images/study report should not be reviewed by the Principal Investigator unless a specific criterion for an attack requires review of the MRI; 5) to clarify that following the independent clinical assessment, the Principal Investigator should decide if any of the clinical criteria have been met and that the criterion with the most significant change should be selected; and 6) if an attack presents in more than one domain, more than one criterion can be selected. These revisions were made to clarify that symptom(s) must meet at least one of the objective criteria for an NMO/NMOSD

attack and that the criteria with the most significant clinical change that the symptoms meet should be selected, subsequent criteria with a lesser clinical change that require MRI review should not be considered. However, the symptom(s) may meet more than one criterion for an NMO/NMOSD attack across a different body system. This is to ensure that MRI images and reports are not reviewed when a significant change meets criteria 1-8, 12, and 13.

31 Section 4.3.1.1 (Identification, Assessment, and Adjudication of an NMO/NMOSD Attack), Adjudication Process (For Attacks Occurring During the RCP): Text was revised to clarify that the AC process will completed when the AC makes their final decision and that this will occur within a total of 14 days (+ 3 days) from the start of the Assessment Visit and that the data set will be provided to the independent AC by the vendor.

32 Table 4.3.1.1-1 (Protocol-defined Criteria for an NMO/NMOSD Attack): The following modifications were made to the table: 1) footnote "c" was removed (re: "Symptoms must meet at least 1 of the objective criteria for NMO/NMOSD attack") because this is explained in the text; 2) footnote "e" (newly designated footnote "d") was modified to add "HM to LP or NLP" to 1-step drop in worsening missing from previous version and to align with Administrative Change 1; and 3) footnote "f" was added to clarify that lesions seen in the optic chiasm also count towards the ON criteria when evaluating Criteria 9, 10, and 11.

33 Figure 4.3.1.1-2 (Process for Assessing Symptoms Affecting the Eye): The flow diagram was updated to better reflect required process; ie, that an MRI is always required at an Assessment Visit; however, review of the images is criterion dependent.

34 Figure 4.3.1.1-3 (Process for Assessing Symptoms Affecting the Spinal Cord): The flow diagram was updated to better reflect required process; ie, that an MRI is always required at an Assessment Visit; however, review of the images is criterion dependent.

35 Figure 4.3.1.1-4 (Process for Assessing Symptoms Affecting the Brain or Brainstem): The flow diagram was updated to better reflect required process; ie, that an MRI is always required at an Assessment Visit; however, review of the images is criterion dependent.

36 Section 4.3.1.2 (Neuroaxis Magnetic Resonance Imaging Scan), MRI for NMO/NMOSD Attacks, and Neuroaxis MRI Documentation and Assessment: The text in these subsections was added or revised for the following reasons: 1) to clarify that a full neuroaxis MRI will be performed of all domains in addition to the scheduled MRI at the time of any Assessment Visit and that the MRI scans will be sent for a central reading and data will be collected for analysis at the end of the study; 2) to clarify the MRI images/study report obtained during an Assessment Visit should not be reviewed by the Principal Investigator unless required for a specific criterion; however, should it become necessary, review of MRI image and local report must be done after the review of all

relevant clinical assessment data is complete. As long as the MRIs are not reviewed by the Principal Investigator prior to review of the clinical assessments no bias will be introduced; 3) to clarify that the Principal Investigator should not use these MRIs for any clinical decision making; and 4) to clarify that qualification MRI scans may require consenting a volunteer subject from the investigative site, depending upon local IRB/EC requirements.

37 Section 4.3.1.3 (Ophthalmology Assessments) and Visual Acuity Assessments: The following text in these subsections was added or revised: 1) text was added to clarify that the ophthalmology examiner is also independent and not otherwise involved in evaluating or examining the subject outside of the protocol to reduce the potential for bias; and 2) text referring to light meters was removed as these are not required.

38 Section 4.3.1.4 (Expanded Disability Status Scale and Functional System Scores): Text was revised to clarify that the EDSS assessment must be conducted by a trained and certified neurologist who is also independent of the treating physician and the subject to reduce the potential for bias.

39 Section 4.3.1.5 (Independent Adjudication Committee): Text was revised following the review of the first few attacks that occurred in the study, and input from the AC necessitated clarifications of the AC role and process. This is outlined in the new text and will be reflected also in an amended charter for the AC. The committee is completely independent and blinded to treatment assignments and/or to pertinent treatment unmasking information of study subjects. Clarified that events not related to NMO/NMOSD that occur in the RCP will be sent to the AC for non-real-time adjudication.

40 Section 4.3.3.4 (Electrocardiogram): Text was revised to add ECG recording to Day 1, which was missing from previous text.

41 Section 4.3.4 (Columbia-Suicide Severity Rating Scale): Text was revised to clarify that the Baseline/Screening questionnaire will be used to assess suicidality in a subject's lifetime and over the 6 months prior to screening.

42 Section 4.3.6 (Clinical Laboratory Tests): Text was modified or added for the following reasons: 1) hepatitis B surface antibody was added to Other Safety Tests for consistency with Exclusion Criterion #24; 2) text was added to clarify that the estimated GFR would be calculated and the GFR must be ≥ 60 mL/minute for study eligibility and for consistency with Exclusion Criterion #3. The estimated GFR would be calculated by the central laboratory based on the plasma and urine creatinine samples provided using the Modification of Diet in Renal Disease formula; 3) tetanus vaccination titers was added to Other Safety Tests; and 4) subsection for Vaccination Titers was added to specify which vaccine titers will be analyzed for the samples already specified in the protocol.

43 Section 4.3.9 (Biomarker Evaluations and Methods): Text was added to explain that plasma cell gene signature data can be considered potentially unmasking and that the

sponsor will not see any potentially unmasking data until after the study has been unmasked for the primary analysis.

44 Table 4.3.10-1 (Estimate of Blood Volume to Be Collected): The total blood volume for Screening was changed from 30 mL to 31 mL to add Ig samples to screening. Total blood volume for Day 1 changed from 38 mL to 37 mL to account for removal of Ig samples at Day 1 and to align with Administrative Change 1.

45 Section 4.5.1.2 (Treatment Administration), Randomized-controlled Period, and Open-label Period: Text in these subsections was revised for the following reasons: 1) to allow some flexibility in the time for subjects completing the RCP without and NMO/NMOSD attack to enter the OLP in the events there is a safety issue; and 2) to clarify that in the event that the second dose of investigational products is delayed due to medical or safety reasons, subsequent dosing must be discussed with the medical monitor prior to administration of investigational product. Additionally, subjects are not permitted to enter the OLP after 14 days, unless there is a safety reason and the medical monitor agrees, in which case a short extension may be granted.

46 Section 4.5.1.3 (Monitoring of Dose Administration): Text was added to state that Japanese subjects must receive their RCP Day 1 dose as in-patients per local Pharmaceuticals and Medical Devices Agency requirements.

47 Section 4.5.2.2 (Rescue Medications): Text was revised to clarify that assessment of new symptoms or worsening of exisiting symptoms should be completed within 5 days to determine if an attack has occurred and that the Principal Investigator can initiate rescue therapy at any time before full assessment is completed and that rescue therapy may include IV corticosteroids, IVIG, and/or PLEX.

48 Section 4.5.3 (Dose Adjustments and Missed Doses): Text was simplified to note that if any dose of investigational product is missed, subsequent dosing and subject management must be discussed with the medical monitor prior to any further dosing.

49 Section 4.6.3 (Methods for Reducing Bias): Text was added or revised for the following reasons: 1) to clarify that the EDSS rater and the ophthalmology examiner must remain independent of the subject during the study to eliminate the potential for bias; and 2) to clarify that the MRI scans should not be reviewed by the Principal Investigator until review of the clinical data for the clinical assessments for an suspected attack type is complete.

50 Section 4.6.4.1 (Unmasking in the Event of a Medical Emergency): Text was added for unmasking in a medical emergency for sites in the Czech Republic to align with the local amendment.

51 Section 4.6.4.3 (Unmasking due to Known or Hypothesized Effects of MEDI-551): Text was added to inform that data from early phase development in non-oncology subject populations receiving MEDI-551 suggest that MEDI-551 administration may be associated with potential unspecified mild reduction in total Ig in individual subjects and

these data will not be made available to investigational sites post randomization through the remainder of the study as this reduction may be potentially unmasking. This addition was made to align with Administrative Change 1. Additionally, text was added to indicate that plasma cell gene signature data and tetanus vaccine titers are potentially unmasking and will not be made available to the sites and plasma cell gene signature samples will not be tested prior to unmasking, and a summary statement that all potentially unmasking data will not be available to the sponsor prior to unmasking of the study at the end of the RCP.

52 Section 4.7.1.1 (Immunosuppressive Medications During the Screening Period): Text was revised to clarify that immunosuppressive medications must be stopped prior to dosing on Day 1, but steroids must continue according to the protocol.

53 Section 4.7.2 (Prohibited Concomitant Medications): Text was added or revised for the following reasons: 1) to clarify that subjects may exit the OLP at any time for any reason, including to seek alternative treatment options as per EMA scientific advice; 2) to clarify that low-dose steroids prohibited by the protocol do not include topical steroids because they are not applied at immunosuppressive doses; and 3) to clarify that there are no prohibited medications during the SFP.

54 Section 4.8.2 (Sample Size and Power Calculations): Text was modified to clarify that the study is event driven and enrollment of subjects will stop when a total of 67 AC-determined NMO/NMOSD attacks occur. The planned number of subjects to be enrolled is 212; however, based on sample size reassessment the number maybe increased to a maximum of 252 subjects. All active randomized subjects at the time that 67 events have occurred will continue in the study to complete the RCP. If eligible, these subjects will be able to roll over to the OLP. Therefore, the sample size will depend on achieving this goal and sample size reassessment.

55 Section 5.2 (Definition of Serious Adverse Events): Text revised for the following reasons: 1) to clarify that for the purposes of this protocol an SAE of death will include NMO/NMOSD disease progression or attack; 2) to clarify that NMO/NMOSD disease progression or attacks that meet the SAE criteria, with the exception of death, do not need to be reported as SAEs in the RCP and OLP since they are study endpoints and to further clarify that NMO/NMOSD attacks that meet the SAE criteria during the screening period or SFP will be reported as SAEs as they are not considered study endpoints during these periods and data would otherwise be lost if not reported; and 3) to reinforce that NMO/NMOSD disease progression or attacks that result in death will be reported as SAEs; and 4) hospitalization for any procedure resulting from an NMO/NMOSD attack will not be reported as an SAE.

56 Section 8 (References): Reference for Siegrist, 2013 was added to the reference list.

57 Appendix 5 (Pain NRS): The NRS form was updated to include a check box for the Attack Follow-up Visit as this was missing from the previous version and was inconsistent with the protocol.

Administrative Change 1; 18Feb2015

Administrative changes were made primarily for consistency and to correct typographical errors. Administrative changes to the protocol are summarized below:

1. Table 4.2.1-1 (Schedule of Screening Procedures): Blood collection for total Ig, IgM, IgG, IgA, and IgE was added for consistency with Exclusion Criterion 23a in Section 4.1.3 (Exclusion Criteria), which states that subjects with a total Ig level of ≤ 900 mg/dL at screening are not eligible for the study.
2. Table 4.2.2.1-1 (Schedule of Randomized-controlled Treatment Period Study Procedures): Blood collection for total Ig, IgM, IgG, IgA, and IgE was removed at Day 1 for consistency with Exclusion Criterion 23a in Section 4.1.3 (Exclusion Criteria), which states that subjects with a total Ig level of ≤ 900 mg/dL at screening are not eligible for the study.
3. Table 4.3.1.1-1 (Protocol-defined Criteria for an NMO/NMOSD Attack): Criterion 8 under ON attack type was corrected to change the word “affected” to “fellow” for consistency with the wording in Criteria 4 and 6 of the same ON attack type as well as in the description of the RAPD assessment in Section 4.3.1.3 (OphthalmologyAssessments).
4. Table 4.3.1.1-1 (Protocol-defined Criteria for an NMO/NMOSD Attack: A fourth example of a 1-step drop in visual acuity was added to Footnote “e,” which described a change in hand motion (HM) to light perception (LP) or no light perception (NLP); this example was omitted in error.
5. Table 4.3.10-1 (Estimate of Blood Volume to Be Collected): The estimated volume of blood to be collected from each subject at Screening was increased 1 mL and the blood to be collected from each subject at Day 1 was decreased by the same amount to account for the transition of Ig testing from Day 1 to Screening.
6. Section 4.6.4.3 (Unmasking due to Known or Hypothesized Effects of MEDI-551): Added that results of Ig testing (total Ig, IgM, IgG, IgA, and IgE) would not be made available to investigation sites post randomization through the remainder of the study.

Protocol Amendment 1; 01Jul2014

Text revisions resulting from this amendment are incorporated in the body of Protocol Amendment 1. The majority of these changes were made in response to feedback from the US FDA Special Protocol Assessment requested by MedImmune. Major changes to the protocol are summarized below.

1. Study Abstract: Updated to be consistent with the changes made to the body of the protocol.

2 Section 1.1 (Disease Background) and Section 1.5 (Rationale for Study): Simplified the description of NMO and its associated characteristics for clarity.

3 Section 1.6.2 (Secondary Hypotheses): The following changes were made: 1) Added MRI lesion hypothesis; and 2) Removed secondary hypotheses pertaining to safety and PK to simplify the section and focus it on the efficacy outcomes.

4 Section 2.1.1 (Primary Objective) and Section 1.6.1 (Primary Hypothesis): Revised the subject population for the primary analysis to include both AQP4-IgG seropositive and seronegative subjects.

5 Section 2.1.2 (Secondary Objectives): Added secondary objective comparing the efficacy of MEDI-551 versus placebo in reducing the cumulative active MRI lesion count (new Gd-enhancing or new/enlarging T2). This study aims to collect longitudinal MRI data during both study periods, including at the time of an attack, to investigate the characteristics and evolution of spinal and brain lesions in NMO/NMOSD.

6 Section 2.1.3 (Exploratory Objectives): Added exploratory objective comparing the effect of MEDI-551 versus placebo on pain as measured using the pain NRS. A number of recent publications have indicated that patients with NMO/NMOSD experience pain throughout the course of their disease. Pain in demyelinating and inflammatory CNS diseases can be disabling and may contribute to a lower quality of life and overall increased health care burden.

7 Section 2.2.1 (Primary Endpoint): The following changes were made: 1) Revised the timeframe of the primary endpoint definition from Day 183 to Day 197 to coincide with the increased length of the RCP, which will be extended by 2 weeks to account for the 2-week oral corticosteroid use between Day 1 and Day 14; 2) Removed the restriction that the primary analysis will be based on AQP4-IgG seropositive subjects. The primary analysis will first be tested in AQP4-IgG seropositive subjects, and if statistically significant, it will be further tested in all subjects; and 3) Removed Table 2.2.1-1 from this section and moved it to Section 4.3.1.1 (Identification, Assessment, and Adjudication of an NMO/NMOSD Attack).

8 Section 2.2.2 (Secondary Endpoints): The following changes were made: 1) Revised the visual acuity endpoint to change the measurement tool from the Sloan Letters Low Contrast Chart to the Landolt C Broken Rings Chart as recommended by MedImmune's NMO Steering Committee; 2) Added an MRI lesion endpoint to coincide with the addition of a secondary objective; and 3) Revised the definition of in-patient hospitalization from hospital stay greater than 24 hours to more than an overnight stay.

9 Section 2.2.3 (Exploratory Endpoint[s]): The following changes were made: 1) Added an endpoint for the pain NRS objective; and 2) Removed the biomarker exploratory endpoint (this endpoint will be analyzed as part of an exploratory investigation.

10 Section 3.1.1 (Overview): The following changes were made: 1) Changed the sample size of the study from 252 to 212 subjects due to the addition of the AQP4-IgG

seronegative subjects as part of the primary endpoints, with the potential to increase the number of subjects to 252 if the sample size needs to be reassessed; 2) Extended the RCP to 197 days to account for the 2-week oral corticosteroid use between Day 1 and Day 14; 3) Revised text for the enrollment of subjects into the OLP and continuation of subjects in the RCP if an NMO/NMOSD attack was not determined by the AC based on comments received from the Agency regarding the identification, assessment, and adjudication of NMO/NMOSD attacks; 4) Revised the time period for the OLP to add a maximum time length of 3 years (after the last subject enters) if regulatory approval or MEDI-551 development is halted after 3 years; 5) Renamed the Long-term Follow-up Period the SFP to evaluate the long-term safety of the investigational product; 6) Added subsections 3.1.1.1 (Screening Period), 3.1.1.2 (Randomization [Day 1]), 3.1.1.3 (Randomized-controlled Period [Day 1 to Day 197]), 3.1.1.4 (Open-label Period), and 3.1.1.5 (Safety Follow-up Period) to clarify existing text (Sections 3.1.1.1 and 3.1.1.2) and to summarize changes made to the design of the study based on the Agency's comments to better define the identification, assessment, and adjudication of NMO/NMOSD attacks and the process of enrolling subjects into the OLP (Sections 3.1.1.3, 3.1.1.4, and 3.1.1.5); 7) Replaced previous Figure 3.1.1-1 (Study Flow Diagram) with new figure.

11 Section 3.1.2 (Treatment Regimen): The following changes were made: 1) Clarified that equivalent antihistamines to diphenhydramine can be used as part of the MEDI-551 premedication regimen; 2) Changed the 2-week tapering reduction schedule to 1 week; and 3) Clarified the text for dosing of subjects in the OLP.

12 Section 3.2.1 (Study Design Rationale): Revised the study design rationale for clarity and for changes made to previous sections of the protocol.

13 Section 3.2.2 (Dose Rationale): Revised the dose rationale for clarity.

14 Section 3.2.3 (Rationale for Study Population): Revised the rationale for study population for clarity, to reflect changes in study design, and to add supporting information.

15 Section 3.2.4 (Rationale for Endpoints): The following changes were made: 1) Revised the rationale for the primary, secondary, and exploratory endpoints for clarity and for consistency with changes made in Sections 2.2.1 (Primary Endpoint), 2.2.2 (Secondary Endpoints), and 2.2.3 (Exploratory Endpoint[s]); and 2) Added Section 3.2.4.4 (Exploratory Investigations) to summarize the rationale for the biomarker endpoint (previously an exploratory endpoint) and added a rationale for investigating T-cell responses since there is increasing evidence for the involvement of effector T cells in the pathogenesis of NMO.

16 Section 3.2.4.1 (Primary Endpoint): The following changes were made: 1) Revised text for primary endpoint to onset of an AC-determined NMO/NMOSD attack on or before

Day 197; 2) Revised text to support limiting subject exposure to placebo by utilizing time to attack as the primary endpoint.

17 Section 3.2.4.2 (Secondary Endpoints): The following changes were made: 1) Changed low contrast visual acuity charts from Sloan Letters Chart to Landolt C Broken Rings Chart; 2) Added MRI-related endpoint rationale and supporting information for collecting longitudinal MRI data during both study periods and at time of attack to investigate the characteristics and evolution of spinal and brain lesions in NMO/NMOSD; 3) Redefined in-patient hospitalization for the purposes of analysis of the secondary endpoint and added supporting information.

18 Section 4.1.1 (Number of Subjects): Clarified the number of subjects planned for the study based on both AQP4-IgG seropositive and seronegative subjects and the addition of subjects for the purposes of reassessing the sample size based on the NMO/NMOSD attack rate.

19 Section 4.1.2 (Inclusion Criteria): The following changes were made: 1) Added to Inclusion Criterion 3b, data from AQP4-IgG seronegative subjects will be reviewed by an independent expert to confirm eligibility; 2) Added to Inclusion Criterion 7 that female partners who are unable to comply with any required contraception method (such as male condom plus spermicide) must use one of the other recommended highly effective contraceptive methods; 3) Changed Inclusion Criterion 7 to state that nonsterilized males must use contraception from Day 1 for 3 months if they are sexually active with a female partner of childbearing potential; and 4) Added Criterion 8, sterilized males, with the appropriate post-vasectomy documentation on the absence of sperm in the ejaculate, who are sexually active with a female partner of childbearing potential, must use a highly effective method of contraception from Day 1 for 3 months after receipt of the final dose of investigational product. Criterion was added to accommodate contraception requirements in the United Kingdom.

20 Section 4.1.3 (Exclusion Criteria): The following changes were made: 1) Clarified Exclusion Criterion 11 to state prior to randomization; 2) Revised Exclusion Criterion 12 to state prior to randomization rather than at the time of screening; and 3) Added Exclusion Criterion 13 excluding subjects who received IVIG within 1 month prior to randomization as it might affect subject outcomes.

21 Section 4.1.4 (Subject Enrollment and Randomization): Added subsection headers (Sections 4.1.4.1 [Enrollment] and 4.1.4.2 [Randomization Process]) for clarity and revised the subsection on Informed Consent Requirements for clarity.

22 Section 4.1.5 (Withdrawal from the Study): Added instructions for subjects who want to discontinue participation in the study during the RCP to consider following protocol-specified assessments until Day 197 and for subjects who want to discontinue the study during the OLP to enter the SFP in order to capture more data on these subjects.

23 Section 4.1.7 (Replacement of Subjects): The following changes were made: 1) Changed adjudicated to AC-determined NMO/NMOSD attacks; and 2) Removed reference to AQP4-IgG serostatus.

24 Section 4.2.1 (Enrollment/Screening Period): The following changes were made: 1) Added that any subject who has an AQP4-IgG seronegative status at screening will have their medical history and MRI data reviewed by an independent NMO expert to ensure that the subject meets the Wingerchuk et al, 2006 diagnostic criteria for NMO; 2) Removed language regarding the definition of NMO/NMOSD relapse as the identification, assessment, and adjudication processes have been updated per comments made by the Agency; 3) Added assessment of JCV antibody titers at screening; 4) Clarified that whole blood for flow cytometry includes B-cell count and cell subsets; 5) Clarified that serum for AQP4-IgG includes serostatus and titer assessments; and 6) Changed APD assessment to RAPD assessment for the ophthalmology examination.

25 Section 4.2.2.1 (Randomized-controlled Treatment Period): The following changes were made: 1) Extended the RCP to Day 197 due to the 2-week oral corticosteroid use between Day 1 and Day 14; this resulted in changing the Day 141 visit to Day 155; 2) Added that subjects should continue in the SFP if they undergo the EDV to follow subjects for safety.

26 Table 4.2.2.1-1 (Schedule of Randomized-controlled Treatment Period Study Procedures): The following changes were made: 1) Changed Study Weeks 20 (Day 141) and 26/EDV (Day 183) to Study Weeks 22 (Day 155) and 28/EDV (Day 197); 2) Changed Pain scale to Pain NRS and deleted assessment at Day 15; 3) Added sample for whole blood for PBMC isolation to be collected a selected sites after the PBMC was added as an exploratory analysis at Days 1, 85, and 197/EDV; 4) Removed ophthalmology examinations for visual fields, OCT, and fundoscopy; and 5) Added mRS to assessments.

27 Section 4.2.2.2 (Open-label Period): The following changes were made: 1) Revised text to clarify that subjects completing Day 197 of the RCP, Day 1 of the OLP should be the same day, but may be delayed up to 14 days without repeating procedures; subjects will not be permitted to enter the OLP after 14 days. The 14 days window was added to give the subject reasonable time to make the decision to enter the OLP without putting the subject at potential increased risk for an attack as he/she will not be treated until entering the OLP or deciding to discontinue from the study and getting another treatment; 2) Added that subjects who have an AC-determined NMO/NMOSD attack during the RCP must enter the OLP within 14 days of AC determination. If more than 14 days elapse since the Assessment Visit, Day 1 procedures should be repeated; 3) Added that MEDI-551 will be administered on OLP Day 1; 4) Revised OLP duration as a minimum of 1 year after the last subject enters and a maximum of 3 years after the last subject enters; 5) Added that subjects entering the OLP will need to reconsent prior to receiving MEDI-551; 6) Changed Day 183+/EDV to Day 365+/EDV for early discontinuation; 7)

Changed Day 183+/EDV to Day 197+/EDV for procedures to be conducted for subjects who withdraw during a scheduled study visit.

28 Table 4.2.2.2-1 (Schedule of Open-label Period Study Procedures): The following changes were made: 1) Added +14 days from Day 197 of the RCP or AC determination to visit window for Day 1 of the OLP; 2) Changed every 12 weeks (Q12W; Day 85) to Week 13 (Day 92), added Week 26 (Day 183) and Week 39, then every 26 weeks (Q26W; Day 274+), changed Q26W/EDV to Week 52, then Q26W/EDV (Day 365+); 3) Added written informed consent to procedures as subjects will require reconsent to enter the OLP; 4) Changed Pain scale to Pain NRS; 5) Removed ophthalmology examinations for visual fields, OCT, and fundoscopy; 6) Added footnotes for procedures to be conducted for clarification.

29 Section 4.2.3 (Study Procedures for Assessment Visits): The following changes were made: 1) Changed Attack Visit to Assessment Visit; 2) Revised that Assessment Visit should be scheduled as soon as possible and within 72 hours of symptom report; 3) Revised to assessments should be concluded as soon as possible, but should not extend beyond 5 days from Day 1 of the Assessment Visit.

30 Table 4.2.3-1 (Schedule of Study Procedures for the Assessment Visit): The following changes were made: 1) Changed Attack Visit to Assessment Visit; 2) Changed Pain scale to Pain NRS; 3) Removed ophthalmology examinations for visual fields, OCT, and fundoscopy; and 4) Added ECG to assessments.

31 Section 4.2.5 (formerly Section 4.2.4, Safety Follow-up Period): The following changes were made: 1) Changed Long-term Follow-up Period to SFP; 2) Added that the SFP will start when a subject discontinues early from the RCP or the OLP, and the length of the SFP will be determined by the time elapsed from time of last dose of investigational product to time of early discontinuation to complete a total of 52 weeks; subjects who discontinue during the RCP will continue with study assessments until Day 197, unless consent is withdrawn; 3) Revised that the SFP will last 52 weeks from the last dose of study drug. The SFP is introduced to follow subjects who prematurely discontinue from the study for safety only as it is not reasonable to request subjects who do not wish to continue in the study to continue with the study procedures.

32 Table 4.2.5-1 (formerly Table 4.2.4-1, Schedule of Procedures for Safety Follow-up Period): The following changes were made: 1) Changed Long-term Follow-up Period to SFP; 2) Removed assessments for assessing new and/or worsening NMO/NMOSD symptoms, whole blood for gene expression, concomitant medications, ophthalmology examinations for visual acuity, low-contrast visual acuity, afferent pupillary defect, and independent EDSS/FSS administration.

33 Section 4.3.1.1 (Identification, Assessment, and Adjudication of an NMO/NMOSD Attack): The following changes were made: 1) Added overview section to explain the process for identification, assessment, and adjudication of an NMO/NMOSD attack;

2) Added revised flow chart; 3) Added process for rescue treatment for an NMO/NMOSD attack and that rescue treatment is to be given at clinical judgment of the investigator; 4) Added subsection, Detailed Procedures, for identifying new or worsening symptoms that may be related to an NMO/NMOSD attack; 5) Updated Table 4.3.1.1-1 (Protocol-defined Criteria for an NMO/NMOSD Attack) and moved to this section from Section 2.2.1; 6) Added 3 flow diagrams for assessing symptoms affecting the eye, spinal cord, and brain or brainstem; 7) Deleted Figure 4.3.1.1-2 (NMO/NMOSD Attack Flow Diagram) as this was replaced by updated Figure 3.1.1-1 (Study Flow Diagram); 8) Added Figures 4.3.1.1-2, 4.3.1.1-3, and 4.4.1.1-4.

34 Section 4.3.1.1 (formerly Section 4.3.1.2), Recording Symptoms of NMO/NMOSD: The following changes were made: 1) Revised that details of NMO/NMOSD symptoms that occur or worsen during the study will be recorded at the Assessment Visit; 2) Added that information regarding new or worsening symptoms may be provided during the study visits, follow-up visit by the site, during bi-weekly telephone calls, or from spontaneous reporting by the subject; 3) Added that a standardized worksheet will be used by the sites to elicit consistent information; 4) Revised that information to be recorded includes date of contact, if symptom(s) was not related to NMO/NMOSD but to an AE, and date of scheduled Assessment Visit.

35 Section 4.3.1.1 (formerly Section 4.3.1.3), Telephone Calls to Subjects Every 2 Weeks: The following changes were made: 1) Added section to define process for telephone contact with subjects and study periods and durations; 2) Added that calls will follow a pre-specified script; 3) Added that if the investigator suspects an NMO/NMOSD attack or impending attack, an Assessment Visit must be scheduled within 72 hours. These changes were made to clarify the telephone call process.

36 Section 4.3.1.2 (formerly Section 4.3.1.4, Neuroaxis Magnetic Resonance Imaging Scan): The following changes were made: 1) Revised that MRI scan will be repeated at 28 weeks instead of at 6 months, and the Principal Investigator should not use these MRIs for any clinical decision making; 2) Added subsection for MRI process for all subjects; 3) Added subsection for process for MRI for NMO/NMOSD attacks; 4) Revised subsection for MRI imaging documentation and assessment; MRI reports and scans will be sent to the AC from the central reading site as part of the assessment data to be used for adjudication.

37 Section 4.3.1.3 (formerly Section 4.3.1.5, Ophthalmology Assessments): The following changes were made: 1) Changed APD to RAPD; 2) Removed examination of visual fields, OCT, and fundoscopy; 3) Added that ophthalmology examiner will not have access to previous visual acuity scores or RAPD findings at the time of a new evaluation to minimize bias, and the ophthalmology assessments should be conducted as specified at the study visit, but prior to MRIs; 4) Changed ophthalmology assessments repeated at Day 183 to Day 197 for both the RCP and OLP; 5) Revised text that ophthalmology examiner provide a full report to the investigator to determine if the findings meet 1 or

more of the protocol-defined criteria for ON attack; 6) Revised subsection to Landolt C Broken Rings Chart and directions for use and conducting the assessments for low-contrast visual acuity and high-contrast visual acuity assessments; 7) Added that the investigator must compare the full examination results received from the ophthalmology examiner against the subject's last assessment.

38 Section 4.3.1.4 (formerly Section 4.3.1.6, Expanded Disability Status Scale and Functional Systems Scores): The following changes were made: 1) Updated text to reflect the latest version of the EDSS/FSS provided by Neurostatus (version 04/10.2) and reference; 2) Revised to inform about process and requirements for the EDSS examiner; 3) Revised text to inform that data generated from the EDSS examination will be entered into an electronic data capture device provided to the site.

39 Section 4.3.1.5 (formerly Section 4.3.1.7, Independent Adjudication Committee): The following changes were made: 1) Revised text to inform that data from all subjects who are assessed at an Assessment Visit will be sent for adjudication by the AC regardless of whether the Principal Investigator has determined that an NMO/NMOSD attack has occurred; 2) Revised text to reflect real-time adjudication, changes to OLP entry, and study design changes.

40 Section 4.3.1.6 (formerly Section 4.3.1.6, Healthcare Resource Utilization): The following changes were made: 1) Added text to define in-patient hospitalization; 2) Revised Table 4.3.1.8-1 (Healthcare Resource Utilization Information to be Collected and Frequency) to change order of parameters; 3) Added text that subjects will be asked to record any medical procedures for NMO/NMOSD in the subject diary.

41 Section 4.3.2 (Patient-reported Outcomes): The following changes were made: 1) Added 5 separate pain NRS to PROs; 2) Revised text for clarity.

42 Section 4.3.2.1 (Pain NRS): The following changes were made: 1) Changed Pain Scale to 11-point pain NRS; 2) Changed text to explain pain NRS.

43 Section 4.3.2.2 (Short Form-36 Health Survey): Removed text that SF-36v2 should be completed prior to any study procedure or information is communicated to the patient and without assistance.

44 Section 4.3.2.3 (Collection of PRO Endpoints): Section heading was added to separate process for collecting PRO endpoints from the SF-36 Healthy Survey.

45 Section 4.3.3.1 (Medical History): The following changes were made: 1) Added date of original diagnosis for NMO/NMOSD is to be collected for subject's disease history; 2) Added diabetes, myasthenia gravis, and pernicious anemia to example of autoimmune diseases to be collected for the subject's medical history.

46 Section 4.3.3.4 (Electrocardiogram): The following changes were made: 1) Changed Day 183 to Day 197 for conducting ECG assessments and added Assessment Visit; 2) Added "abnormal clinically significant" and "abnormal not clinically significant" to ECG review; 3) Removed respiratory rate as a measurement for the ECG.

47 Section 4.3.4 (formerly Section 4.3.3.7, Columbia-Suicide Severity Rating Scale): The following changes were made: 1) Changed administration of the “Since Last Visit” version from 3 and 6 months to all scheduled visits and Assessment Visits; 2) Changed Long-term Follow-up Period to SFP.

48 Section 4.3.6 (formerly Section 4.3.4, Clinical Laboratory Tests): The following changes were made: 1) Removed exogenous corticosteroids from serum chemistry; 2) Added HIV-1 and -2 testing to be done only at screening; 3) Added polyomavius JC antibody titers to be collected only at screening and frozen for future testing, if required; 4) Revised GFR to be calculated by central laboratory based on plasma and urine creatinine samples and that the GFR must be confirmed by the site upon receipt of central laboratory report and prior to randomization.

49 Section 4.3.9 (formerly Section 4.3.7, Biomarker Evaluations and Methods); subsection, Serum for AQP4-IgG Assay: Added screening to baseline and that study visit assays will be used to determine AQP4-IgG serostatus.

50 Section 4.3.9.1 (formerly Section 4.3.7.1, Exploratory Investigations): The following changes were made: 1) Added new section heading to separate exploratory assessments from other study assessments; 2) Added subsection, Blood for Peripheral Blood Mononuclear Cell Isolation for T-cell Evaluation, and text for collection of PBMC.

51 Section 4.3.10 (formerly, Section 4.3.8, Estimate of Volume of Blood to Be Collected): The following changes were made: 1) Changed Long-term Follow-up Period to SFP; 2) Revised Table 4.3.10-1 (Estimate of Blood Volume to Be Collected) and added visit days to the OLP with corresponding blood volumes and adjusted total blood volume; 3) Changed Attack Visit to Assessment Visit; 4) Added footnote for PBMC to be collected at specified sites and adjusted total blood volume for this blood collection.

52 Section 4.5.1.1 (Investigational Product Dose Preparation): The following changes were made: 1) Added that MEDI-551 and placebo are supplied as a clear to opalescent, colorless to yellow liquid and free from or practically free from visible particles to Investigational Product Inspection; 2) Added “to opalescent” and “to yellow liquid” and “free from or practically free from particles” to the description of the appearance of MEDI-551 and placebo.

53 Section 4.5.1.2 (Treatment Administration): The following changes were made: 1) Revised the Open-label Period subsection and added that for subjects completing Day 197 of the RCP, Day 1 of the OLP should be the same day, although it may be delayed for up to 14 days and procedures will not need to be repeated.

54 Section 4.5.2.1 (Oral Corticosteroid Use): The following changes were made: 1) Changed the last day of oral corticosteroid use to Day 14; 2) Modified the tapering schedule to be completed on Day 21.

55 Section 4.5.2.2 (Rescue Medications): The following changes were made: 1) Changed investigator-identified NMO/NMOSD to NMO/NMOSD attack that meets the

protocol-defined criteria; 2) Removed reference to IV corticosteroids; 3) Revised text that all medications given to the subject during the study will be recorded, not only other study medications.

56 Section 4.5.3 (Dose Adjustments and Missed Doses): Revised to extend the length of the RCP by 2 weeks, from Day 183 to Day 197. The RCP was extended for 2 weeks to accommodate the PD of MEDI-551. Full depletion of B cells occurs at least 2 weeks after the investigational product administration. These first 2 weeks will be covered by oral steroid treatment.

57 Section 4.6.3 (Methods for Reducing Bias): Section was added to present steps for mitigating/reducing bias when assessing an NMO/NMOSD attack.

58 Section 4.7.2 (Prohibited Concomitant Medications): The following changes were made: 1) Revised wording for immunosuppressants from the treatment of NMO/NMOSD attacks to the prevention of NMO/NMOSD attacks; 2) Added RCP to low-dose steroids for clarification.

59 Section 4.8.1 (General Considerations): The following changes were made: 1) Revised text for clarity that the primary endpoint and key secondary endpoints will be analyzed and reported; 2) Removed AQP4-IgG seropositive subjects from analysis of major secondary endpoints.

60 Section 4.8.2 (Sample Size and Power Calculations): The following changes were made: 1) Changed adjudicated NMO/NMOSD attack to AC-determined attack and Day 183 to Day 197; 2) Added stratification ratio of approximately 80:20 AQP4-IgG seropositive to seronegative strata; 3) Added hazard rate of 1.0/year for seronegative subjects receiving placebo.

61 Section 4.8.3.1 (Primary Efficacy Analysis): The following changes were made:
1) Updated null and alternative hypotheses associated with the primary endpoint;
2) Revised text regarding treatment effect for the AQP4-IgG seropositive cohort;
3) Revised text that primary analysis will be based on 67 AC-determined NMO/NMOSD attacks observed and analysis will be conducted once all ongoing subjects have been dosed and completed the RCP.

62 Section 4.8.3.2 (Additional Analyses of the Primary Endpoint): Changes were made to the sensitivity analyses to be performed for the primary endpoint. The primary endpoint is determined in this study by the AC, which is composed of 3 adjudicators who review and assess the data related to the diagnosis of an attack. The sensitivity analysis will assess the consistency and objectivity of the AC members in making the diagnosis of an attack.

63 Section 4.8.3.3 (Secondary Efficacy Analyses): The following changes were made:
1) Changed Sloan Letters Low Contrast Chart to Landolt C Broken Rings Chart as an assessment tool for measuring low-contrast visual acuity; 2) Revised text regarding

treatment effect for secondary efficacy endpoints to be based on the cumulative number of active MRI lesions.

64 Section 4.8.3.4 (Exploratory Analyses): Section was moved to new Subsection 4.8.6.3 (Analysis of Exploratory Biomarkers).

65 Section 4.8.3.4 (Subgroup Analyses): A new analysis, AQP4-IgG serostatus (positive vs negative) as determined at screening, was added to the subgroup analyses.

66 Section 4.8.5.2 (Pain NRS): Pain NRS was added as a PRO.

67 Section 4.8.6 (formerly Analysis of Immunogenicity) was changed to Analysis of Immunogenicity, Pharmacokinetics, and Exploratory Biomarkers, and Subsections 4.8.6.1 (Analysis of Immunogenicity), 4.8.6.2 (Analysis of Pharmacokinetics), and 4.8.6.3 (Analysis of Exploratory Biomarkers) were added.

68 Section 4.8.7 (Additional Analyses): The following changes were made: 1) Changed adjudicated to AC-determined NMO/NMOSD attack; 2) Added agreement between site-determined and AC-determined attack will be assessed by Kappa statistic; 3) Added an inter- and intra-rater reliability assessment will be performed based on random selection of all events contributing to the primary endpoint and a shift analysis of scores from the mRS will be performed.

69 Section 4.8.9 (Control of Type I Error): Section changed to “Control of Type I Error” and previous text was deleted. New text was added to present methods of testing for the primary and secondary null hypotheses.

70 Section 4.8.10.1 (Masked Sample Size Reassessment): The following changes were made: 1) Changed threshold for sample size reassessment from 25% to when 104 (approximately 50%) subjects complete the RCP; 2) Changed the ratio of cumulative number of AC-determined NMO/NMOSD attacks to subject ratio to approximately 27% to increase the sample size up to 252 subjects; 3) Added that reassessment will only be performed prior to the futility assessment. The increase in sample size will be necessary only if the number of attacks required (67) cannot be reached with the planned number of subjects. To keep this decision independent from the outcome of the unblinded interim analysis, the reassessment of sample size must be done before the futility assessment.

71 Section 6.2.1.1 (Direct Access to Source Data): Subsection was added to ensure compliance with Japan regulatory authorities regarding monitoring, auditing, and access to study documents for study sites in Japan.

72 Section 6.3 (Study Timetable and End of Study): The following changes were made: 1) Subsection 6.3.1 (Discontinuation or Suspension of the Study Program) and Subsection 6.3.2 (Completion of the Study) were added for clarification and to ensure compliance with Japan regulatory authorities regarding discontinuation or suspension of the study by the Sponsor and to clarify the Principal Investigator’s responsibility for notifying the head of the study site regarding completion of the study for study sites in Japan.

73 Section 7.1 (Ethical Conduct of the Study): Subsection was revised to add text for regulatory requirements regarding ethical conduct of the study in Japan.

74 Section 7.3.1 (Ethics and Regulatory Review in Japan): Subsection was added to ensure compliance with Japan regulatory authorities regarding IRB approval for study sites in Japan.

75 Section 7.4 (Informed Consent): The following changes were made: 1) Added text for clarification for communicating new information about the investigational product to the subject; 2) Added text for instructing the subject if changes are made to the ICF.

76 Section 7.5.1 (Deviation From the Clinical Study Protocol): Subsection was added for clarification regarding deviations to the study protocol and documentation of deviations.

77 Section 7.6 (Audits and Inspections): Text was added to this section for clarification that all study data may be subject to reliability review and onsite GCP inspection by regulatory authorities.

78 Section 8 (References): Section was updated to reflect reference changes throughout the protocol.

79 Appendix 4 (Expanded Disability Status Scale and Functional Systems Scores): The previous version of the EDSS was replaced with the current version.

80 Appendix 5 (Pain NRS): The appendix was changed to Pain NRS and sample assessment was replaced with the Pain NRS.

81 Appendix 9 (Sample Size Calculation): Text was revised to address changes to the sample size calculations and hazard rates.

82 Appendix 10 (Details of Futility Analysis): Title changed from “Futility Criterion” to “Futility Analysis.”

83 Appendix 11 (Details of Masked Sample Size Re-assessment): The following changes were made: 1) Revised text to reflect changes in sample size re-assessment; 2) Updated Table 9-5 (Masked Sample Size Reassessment Examples [60% Treatment Effect]) to reflect changes in text.

84 Appendix 12 (Type I Error Control): Appendix was added to add Type I error control and add Table 9-6 (List of Null Hypotheses Considered Under Multiplicity Adjustment Procedure), Figure 9-7 (Multiplicity Adjustment Strategy), Table 9-7 (Alpha Allocation Rule), and Table 9-8 (Alpha Propagation Rule).

85 Appendix 13 (Assessment and Collection of Adverse Events): This appendix was added to provide AE and SAE variables to be collected during the study and to further explain methods for assessment and collection of AEs and SAEs.

Appendix 1 Signatures

Sponsor Signature(s)

A Double-masked, Placebo-controlled Study with Open-label Period to Evaluate the Efficacy and Safety of MEDI-551 in Adult Subjects with Neuromyelitis Optica and Neuromyelitis Optica Spectrum Disorders

I agree to the terms of this protocol amendment.

Signature and date:

Eliezer Katz, MD FACS

Vice President

Clinical Development Lead

Viela Bio, Inc

One MedImmune Way, Gaithersburg MD, 20878, USA

Telephone number: 1-301-398-1171

Signature of Principal or Coordinating Investigator

A Double-masked, Placebo-controlled Study with Open-label Period to Evaluate the Efficacy and Safety of MEDI-551 in Adult Subjects with Neuromyelitis Optica and Neuromyelitis Optica Spectrum Disorders

I, the undersigned, have reviewed this protocol amendment, and I agree to conduct this protocol in accordance with ethical principles that have their origin in the Declaration of Helsinki and are consistent with the International Council for Harmonisation (ICH) guidelines on Good Clinical Practice (GCP), any applicable laws and requirements, and any conditions required by a regulatory authority and/or Institutional Review Board/Independent Ethics Committee (IRB/IEC).

I understand that the protocol amendment may not be modified without written approval of the Sponsor. All changes to the protocol amendment must be submitted to the applicable regulatory authority and IRB/IEC, and must be approved by the IRB/IEC prior to implementation except when necessary to eliminate immediate hazards to the subjects or when the change(s), as deemed by the Sponsor, involves only logistical or administrative changes. Documentation of IRB/IEC approval must be sent to the Sponsor immediately upon receipt.

Signature and date: ________________________________

Name and title: ________________________________

Address including postal code: ___________________________

____

Telephone number: ________________________________

Site/Center Number (if available) _______

This document contains confidential information, which should not be copied, referred to, released, or published without written approval from MedImmune or AstraZeneca. Investigators are cautioned that the information in this protocol may be subject to change and revision.

Appendix 2 National Institute of Allergy and Infectious Diseases and Food Allergy and Anaphylaxis Network Guidance for Anaphylaxis Diagnosis

Sampson HA, Muñoz-Furlong A, Campbell RL, Adkinson FN Jr, Bock SA, Branum A, et al. Second symposium on the definition and management of anaphylaxis: Summary report -- Second National Institute of Allergy and Infectious Diseases/Food Allergy and Anaphylaxis Network symposium. J Allergy Clin Immunol. 2006;1175(3):391-7.

The National Institute of Allergy and Infectious Diseases and Food Allergy and Anaphylaxis Network define anaphylaxis as a serious allergic reaction that is rapid in onset and may cause death. They recognize 3 categories of anaphylaxis, with criteria designated to capture from 80% of cases (category 1) to > 95% of all cases of anaphylaxis (for all 3 categories).

1 Acute onset of an illness (minutes to several hours) with involvement of the skin, mucosal tissue, or both (eg, generalized hives, pruritus or flushing, swollen lips-tongue-uvula)

   AND AT LEAST ONE OF THE FOLLOWING

   (a) Respiratory compromise (eg, dyspnea, wheeze-bronchospasm, stridor, reduced peak expiratory flow [PEF], hypoxemia)
   (b) Reduced BP or associated symptoms of end-organ dysfunction (eg, hypotonia [collapse], syncope, incontinence)

2 Two or more of the following that occur rapidly after exposure to a likely allergen for that patient (minutes to several hours):
   (a) Involvement of the skin-mucosal tissue (eg, generalized hives, itch-flush, swollen lips-tongue-uvula)
   (b) Respiratory compromise (eg, dyspnea, wheeze-bronchospasm, stridor, reduced PEF, hypoxemia)
   (c) Reduced BP or associated symptoms (eg, hypotonia [collapse], syncope, incontinence)
   (d) Persistent gastrointestinal symptoms (eg, crampy abdominal pain, vomiting)

3 Reduced BP after exposure to known allergen for that patient (minutes to several hours):
   (a) Infants and children: low systolic BP (age specific) or greater than 30% decrease in systolic BP
   (b) Adults: systolic BP of less than 90 mm Hg or greater than 30% decrease from that person's baseline

Appendix 3 Additional Safety Guidance

Assessment of Severity

Assessment of severity is one of the responsibilities of the investigator in the evaluation of AEs and SAEs. The determination of severity should be made by the investigator based upon medical judgment and the severity categories of Grade 1 to 5 as defined below.

Grade 1 (mild)	An event that is usually transient and may require only minimal treatment or therapeutic intervention. The event does not generally interfere with usual activities of daily living.
Grade 2 (moderate)	An event that is usually alleviated with additional specific therapeutic intervention. The event interferes with usual activities of daily living, causing discomfort but poses no significant or permanent risk of harm to the subject.
Grade 3 (severe)	An event that requires intensive therapeutic intervention. The event interrupts usual activities of daily living, or significantly affects the clinical status of the subject.
Grade 4 (life threatening)	An event, and/or its immediate sequelae, that is associated with an imminent risk of death or with physical or mental disabilities that affect or limit the ability of the subject to perform activities of daily living (eating, ambulation, toileting, etc).
Grade 5 (fatal)	Death (loss of life) as a result of an event.

It is important to distinguish between serious criteria and severity of an AE. Severity is a measure of intensity whereas seriousness is defined by the criteria in Section 5.2. A Grade 3 AE need not necessarily be considered an SAE. For example, a Grade 3 headache that persists for several hours may not meet the regulatory definition of an SAE and would be considered a nonserious event, whereas a Grade 2 seizure resulting in a hospital admission would be considered an SAE.

Abnormal laboratory results may be graded according to NCI Common Terminology Criteria for Adverse Events V4.0 (NCI, 2009).

Assessment of Relationship

<u>Relationship to Investigational Product</u>

The investigator is required to provide an assessment of relationship of AEs and SAEs to the investigational product.

An event will be considered "not related" to use of the investigational product if any of the following tests are met:

- An unreasonable temporal relationship between administration of the investigational product and the onset of the event (eg, the event occurred either before, or too long after, administration of the investigational product for it to be considered product-related)
- A causal relationship between the investigational product and the event is biologically implausible (eg, death as a passenger in an automobile accident)
- A clearly more likely alternative explanation for the event is present (eg, typical adverse reaction to a concomitant drug and/or typical disease-related event)

Individual AE/SAE reports will be considered "related" to use of the investigational product if the "not related" criteria are not met.

"Related" implies that the event is considered to be "associated with the use of the drug" meaning that there is "a reasonable possibility" that the event may have been caused by the product under investigation (ie, there are facts, evidence, or arguments to suggest possible causation).

Relationship to Protocol Procedures

The investigator is also required to provide an assessment of relationship of SAEs to protocol procedures on the SAE Report Form. This includes nontreatment-emergent SAEs (ie, SAEs that occur prior to the administration of investigational product) as well as treatment-emergent SAEs. A protocol-related SAE may occur as a result of a procedure or intervention required during the study (eg, blood collection, washout of an existing medication). The following guidelines should be used by investigators to assess the relationship of SAEs to the protocol:

Protocol related: The event occurred due to a procedure/intervention that was described in the protocol for which there is no alternative etiology present in the subject's medical record.

Not protocol related: The event is related to an etiology other than the procedure/ intervention that was described in the protocol (the alternative etiology must be documented in the study subject's medical record).

Appendix 4 Kurtzke Expanded Disability Status Scale and Functional Systems Scores

neurostatus scoring

Definitions for a standardised, quantified neurological examination and assessment of Kurtzke's Functional Systems and Expanded Disability Status Scale in Multiple Sclerosis

Slightly modified from J.F. Kurtzke, Neurology 1983:33,1444-52
©2011 Ludwig Kappos, MD, Neurology, University Hospital Basel, 4031 Basel, Switzerland; Version 04/10.2

EQUIVALENCE WITH PREVIOUS VERSIONS

This version of the neurostatus scoring guidelines is fully compatible with previous versions. Additional help is provided by clarifying some definitions and by introducing an ambulation score in order to reduce measurement noise. But these changes do not imply changes in scoring levels.

GENERAL GUIDELINES

To ensure unbiased EDSS assessment in controlled clinical trials, the EDSS rater should not inquire about the patients' condition except as necessary to perform the EDSS assessment. Patients must be observed to walk the required distance.
The functional system and EDSS scores should reflect the MS related deficits only. In case of doubt the examining physician should assume a relation to MS.
Temporary signs or symptoms that are not due to multiple sclerosis, e.g. temporal immobilisation after fracture of one limb, as well as permanent signs or symptoms that are not due to multiple sclerosis, e.g leg amputation after accident, will not be taken into consideration when assessing the FS scores and EDSS steps, but need to be noted in neurostatus and commented by adding "P" next to the respective field on the scoring sheet for permanent findings and "T" for temporary findings.

FUNCTIONAL SYSTEMS (FS)

A neurostatus score "signs only" is noted when the examination reveals signs of which the patient is unaware.
A score of 1 in a Functional System implies that the patient is not aware of the deficit and that the deficit or sign does not interfere with normal daily activities. However, this general rule does not apply to the Visual, Bowel/Bladder and Cerebral FS.

EXPANDED DISABILITY STATUS SCALE (EDSS)

The EDSS step should not be lower than the score of any individual FS, with the exception of the Visual and Bowel/Bladder FS before conversion.

EDSS steps from 0 up to 4.0 should not change compared to the previous examination, unless there is a change by one grade in at least one FS score.
EDSS steps from 0 up to 1.5 can only apply if ambulation is "unrestricted".
EDSS steps from 2.0 up to 5.0 are defined by the Functional System (FS) scores and/or walking range restriction. As an example, EDSS step 5.0 is possible with an unrestricted ambulation. EDSS steps from 2.0 up to 4.0 does only apply in individuals when at least "fully ambulatory" (able to walk ≥500 meters). If ambulation is assessed as "restricted" the pyramidal or cerebellar FS must be ≥2.
EDSS steps ≥ 5.5 are exclusively defined by the ability to ambulate, the assistance required or the use of a wheelchair.

1 VISUAL (OPTIC) FUNCTIONS

VISUAL ACUITY

The visual acuity score is based on the line in the Snellen chart at 20 feet (5 meters) for which the patient makes no more than one error, using best available correction. Alternatively, best corrected near vision can be assessed, but this should be noted and consistently performed during follow-up examinations. Switching from near to distance visual acuity measurements should be avoided in follow-up examinations.

VISUAL FIELDS

0 normal
1 signs only: deficits present only on formal (confrontational) testing
2 moderate: patient aware of deficit, but incomplete hemianopsia on examination
3 marked: complete homonymous hemianopsia or equivalent

SCOTOMA

0 none
1 small: detectable only on formal (confrontational) testing
2 large: spontaneously reported by patient

*DISC PALLOR

0 not present
1 present

NOTE

When determining the EDSS step, the Visual FS score must be converted to a lower score as follows:

Visual FS Score	6	5	4	3	2	1
Converted Visual FS Score	4	3	3	2	2	1

FUNCTIONAL SYSTEM SCORE

0 normal
1 disc pallor and/or small scotoma and/or visual acuity (corrected) of worse eye less than 20/20 (1.0) but better than 20/30 (0.67)
2 worse eye with maximal visual acuity (corrected) of 20/30 to 20/59 (0.67–0.34)
3 worse eye with large scotoma and/or moderate decrease in fields and/or maximal visual acuity (corrected) of 20/60 to 20/99 (0.33–0.21)
4 worse eye with marked decrease of fields and/or maximal visual acuity (corrected) of 20/100 to 20/200 (0.2–0.1); grade 3 plus maximal acuity of better eye of 20/60 (0.33) or less
5 worse eye with maximal visual acuity (corrected) less than 20/200 (0.1); grade 4 plus maximal acuity of better eye of 20/60 (0.33) or less
6 grade 5 plus maximal visual acuity of better eye of 20/60 (0.33) or less

* = optional part of the examination.

2 BRAINSTEM FUNCTIONS

EXTRAOCULAR MOVEMENTS (EOM) IMPAIRMENT

0 none
1 signs only: subtle and barely clinically detectable EOM weakness, patient does not complain of blurry vision, diplopia or discomfort
2 mild: subtle and barely clinically detectable EOM weakness of which patient is aware; or obvious incomplete paralysis of any eye movement of which patient is not aware
3 moderate: obvious incomplete paralysis of any eye movement of which patient is aware; or complete loss of movement in one direction of gaze in either eye
4 marked: complete loss of movement in more than one direction of gaze in either eye

NYSTAGMUS

0 none
1 signs only or mild: gaze evoked nystagmus below the limits of "moderate" (equivalent to a Brainstem FS score of 1)
2 moderate: sustained nystagmus on horizontal or vertical gaze at 30 degrees, but not in primary position, patient may or may not be aware of the disturbance
3 severe: sustained nystagmus in primary position or coarse persistent nystagmus in any direction that interferes with visual acuity; complete internuclear ophthalmoplegia with sustained nystagmus of the abducting eye; oscillopsia

TRIGEMINAL DAMAGE

0 none
1 signs only
2 mild: clinically detectable numbness of which patient is aware
3 moderate: impaired discrimination of sharp/dull in one, two or three trigeminal branches; trigeminal neuralgia (at least one attack in the last 24 hours)
4 marked: unable to discriminate between sharp/dull or complete loss of sensation in entire distribution of one or both trigeminal nerves

FACIAL WEAKNESS

0 none
1 signs only
2 mild: clinically detectable facial weakness of which patient is aware
3 moderate: incomplete facial palsy, such as weakness of eye closure that requires patching overnight or weakness of mouth closure that results in drooling
4 marked: complete unilateral or bilateral facial palsy with lagophthalmus or difficulty with liquids

HEARING LOSS

0 none
1 signs only: hears finger rub less in one or both sides and has lateralized Weber test but does not complain of any hearing problem
2 mild: as in 1 but is aware of hearing problem
3 moderate: does not hear finger rub on one or both sides, misses several whispered numbers
4 marked: misses all or nearly all whispered numbers

DYSARTHRIA

0 none
1 signs only
2 mild: clinically detectable dysarthria of which patient is aware
3 moderate: obv. dysarthria during ordinary conversation that impairs comprehensibility
4 marked: incomprehensible speech
5 inability to speak

DYSPHAGIA

0 none
1 signs only
2 mild: difficulty with thin liquids
3 moderate: difficulty with liquids and solid food
4 marked: sustained difficulty with swallowing; requires a pureed diet
5 inability to swallow

OTHER CRANIAL NERVE FUNCTIONS

0 normal
1 signs only
2 mild disability: clinically detectable deficit of which patient is usually aware
3 moderate disability
4 marked disability

FUNCTIONAL SYSTEM SCORE

0 normal
1 signs only
2 moderate nystagmus and/or moderate EOM impairment and/or other mild disability
3 severe nystagmus and/or marked EOM impairment and/or moderate disability of other cranial nerves
4 marked dysarthria and/or other marked disability
5 inability to swallow or speak

3 PYRAMIDAL FUNCTIONS

REFLEXES

0 absent
1 diminished
2 normal
3 exaggerated
4 nonsustained clonus (a few beats of clonus)
5 sustained clonus

Cutaneous Reflexes
0 normal
1 weak
2 absent

*Palmomental Reflex
0 absent
1 present

Plantar Response
0 flexor
1 neutral or equivocal
2 extensor

LIMB STRENGTH

The weakest muscle in each group defines the score for that muscle group. Use of optional functional tests (hopping on one foot and walking on heels/toes), is highly recommended in order to assess BMRC grades 3–5.

BMRC RATING SCALE

0 no muscle contraction detected
1 visible contraction without visible joint movement
2 visible movement only on the plane of gravity
3 active movement against gravity, but not against resistance
4 active movement against resistance, but not full strength
5 normal strength

FUNCTIONAL TESTS

*Pronator Drift (upper extremities) Pronation and downward drift:
0 none
1 mild
2 evident

*Position Test (lower extremities – ask patient to lift both legs together, with legs fully extended at the knee) Sinking:
0 none
1 mild
2 evident
3 able to lift only one leg at a time (grade from the horizontal pos. at the hip joints…°)
4 unable to lift one leg at a time

*Walking on heels/toes
0 normal
1 impaired
2 not possible

*Hopping on one foot
0 normal
1 6–10 times
2 1–5 times
3 not possible

LIMB SPASTICITY (AFTER RAPID FLEXION OF THE EXTREMITY)

0 none
1 mild: barely increased muscle tone
2 moderate: moderately increased muscle tone that can be overcome and full range of motion is possible
3 severe: severely increased muscle tone that is extremely difficult to overcome and full range of motion is not possible
4 contracted

GAIT SPASTICITY

0 none
1 barely perceptible
2 evident: minor interference with function
3 permanent shuffling: major interference with function

OVERALL MOTOR PERFORMANCE

0 normal
1 abnormal weakness (as compared to peers) in performing more demanding tasks, e.g. when walking longer distances, but no reduction in limb strength on formal (confrontational) testing
2 Reduction in strength of individual muscle groups at confrontational testing

FUNCTIONAL SYSTEM SCORE

0 normal
1 abnormal signs without disability
2 minimal disability: patient complains of motor-fatigability or reduced performance in strenuous motor tasks (motor performance grade 1) and/or BMRC grade 4 in one or two muscle groups
3 mild to moderate paraparesis or hemiparesis: usually BMRC grade 4 in more than two muscle groups;
and/or BMRC grade 3 in one or two muscle groups (movements against gravity are possible);
and/or severe monoparesis: BMRC grade 2 or less in one muscle group
4 marked paraparesis or hemiparesis: usually BMRC grade 2 in two limbs or monoplegia with BMRC grade 0 or 1 in one limb;
and/or moderate tetraparesis: BMRC grade 3 in three or more limbs
5 paraplegia: BMRC grade 0 or 1 in all muscle groups of the lower limbs;
and/or marked tetraparesis: BMRC grade 2 or less in three or more limbs;
and/or hemiplegia;
6 tetraplegia: BMRC grade 0 or 1 in all muscle groups of the upper and lower limbs

4 CEREBELLAR FUNCTIONS

HEAD TREMOR

0 none
1 mild
2 moderate
3 severe

TRUNCAL ATAXIA

0 none
1 signs only
2 mild: swaying with eyes closed
3 moderate: swaying with eyes open
4 severe: unable to sit without assistance

LIMB ATAXIA (TREMOR / DYSMETRIA AND RAPID ALTERNATING MOVEMENTS)

0 none
1 signs only
2 mild: tremor or clumsy movements easily seen, minor interference with function
3 moderate: tremor or clumsy movements interfere with function in all spheres
4 severe: most functions are very difficult

TANDEM (STRAIGHT LINE) WALKING

0 normal
1 impaired
2 not possible

GAIT ATAXIA

0 none
1 signs only
2 mild: problems with balance realized by patient and/or significant other
3 moderate: abnormal balance with ordinary walking
4 severe: unable to walk more than a few steps unassisted or requires a walking aid or assistance by another person because of ataxia

ROMBERG TEST

0 normal
1 mild: mild instability with eyes closed
2 moderate: not stable with eyes closed
3 severe: not stable with eyes open

OTHER CEREBELLAR TESTS

0 normal
1 mild abnormality
2 moderate abnormality
3 severe abnormality

NOTE

The presence of severe gait and/or truncal ataxia alone (without severe ataxia in three or four limbs) results in a Cerebellar FS score of 3.
If weakness or sensory deficits interfere with the testing of ataxia, score the patient's actual performance. To indicate the possible role of weakness make an "X" after the Cerebellar FS score.

FUNCTIONAL SYSTEM SCORE

0 normal
1 abnormal signs without disability
2 mild ataxia and/or moderate station ataxia (Romberg) and / or tandem walking not possible
3 moderate limb ataxia and / or moderate or severe gait/truncal ataxia
4 severe gait/truncal ataxia and severe ataxia in three or four limbs
5 unable to perform coordinated movements due to ataxia
X pyramidal weakness (BMRC grade 3 or worse in limb strength) or sensory deficits interfere with cerebellar testing

5 SENSORY FUNCTIONS

SUPERFICIAL SENSATION (LIGHT TOUCH AND PAIN)

0 normal
1 signs only: slightly diminished sensation (temperature, figure-writing) on formal testing of which patient is not aware
2 mild: patient is aware of impaired light touch or pain, but is able to discriminate sharp/dull
3 moderate: impaired discrimination of sharp/dull
4 marked: unable to discriminate between sharp/dull and/or unable to feel light touch
5 complete loss: anaesthesia

VIBRATION SENSE (AT THE MOST DISTAL JOINT)

0 normal
1 mild: graded tuning fork 5–7 of 8; alternatively, detects more than 10 seconds but less than the examiner
2 moderate: graded tuning fork 1–4 of 8; alternatively, detects between 2 and 10 sec.
3 marked: complete loss of vibration sense

POSITION SENSE

0 normal
1 mild: 1–2 incorrect responses, only distal joints affected
2 moderate: misses many movements of fingers or toes; proximal joints affected
3 marked: no perception of movement, astasia

*LHERMITTE'S SIGN

Does not contribute to the Sensory FS score
0 negative
1 positive

*PARAESTHESIAE (TINGLING)

Does not contribute to the Sensory FS score
0 none
1 present

FUNCTIONAL SYSTEM SCORE

0 normal
1 mild vibration or figure-writing or temperature decrease only in one or two limbs
2 mild decrease in touch or pain or position sense or moderate decrease in vibration in one or two limbs;
and/or mild vibration or figure-writing or temperature decrease alone in more than two limbs
3 moderate decrease in touch or pain or position sense or marked reduction of vibration in one or two limbs;
and/or mild decrease in touch or pain or moderate decrease in all proprioceptive tests in more than two limbs
4 marked decrease in touch or pain in one or two limbs;
and/or moderate decrease in touch or pain and/or marked reduction of proprioception in more than two limbs
5 loss (essentially) of sensation in one or two limbs;
and/or moderate decrease in touch or pain and/or marked reduction of proprioception for most of the body below the head
6 sensation essentially lost below the head

6 BOWEL AND BLADDER FUNCTIONS

URINARY HESITANCY AND RETENTION

0 none
1 mild: no major impact on lifestyle
2 moderate: urinary retention; frequent urinary tract infections
3 severe: requires catheterisation
4 loss of function: overflow incontinence

URINARY URGENCY AND INCONTINENCE

0 none
1 mild: no major impact on lifestyle
2 moderate: rare incontinence occurring no more than once a week; must wear pads
3 severe: frequent incontinence occurring from several times a week to more than once a day; must wear urinal or pads
4 loss of function: loss of bladder control

BLADDER CATHETERISATION

0 none
1 intermittent self-catheterisation
2 constant catheterisation

BOWEL DYSFUNCTION

0 none
1 mild: no incontinence, no major impact on lifestyle, mild constipation
2 moderate: must wear pads or alter lifestyle to be near lavatory
3 severe: in need of enemata or manual measures to evacuate bowels
4 complete loss of function

*SEXUAL DYSFUNCTION

Male
0 none
1 mild: difficulty to maintain erection during intercourse, but achieves erection and still has intercourse
2 moderate: difficulty to achieve erection, decrease in libido, still has intercourse and reaches orgasm
3 severe: marked decrease in libido, inability to achieve full erection, intercourse with difficulty and hypoorgasmia
4 loss of function

Female
0 none
1 mild: mild lack of lubrication, still sexually active and reaches orgasm
2 moderate: dysparunia, hypoorgasmia, decrease in sexual activity
3 severe: marked decrease in sexual activity, anorgasmia
4 loss of function

NOTE

When determining the EDSS step, the Bowel and Bladder FS score must be converted to a lower score as follows:

Bowel and Bladder FS Score	6	5	4	3	2	1
Converted Bowel and Bladder FS Score	5	4	3	3	2	1

Sexual dysfunction can be documented but in general does not impact on FS score because of obvious difficulties in assessment by examining physician

FUNCTIONAL SYSTEM SCORE

0 normal
1 mild urinary hesitancy, urgency and/or constipation
2 moderate urinary hesitancy/retention and/or moderate urinary urgency/incontinence and/or moderate bowel disfunction
3 frequent urinary incontinence or intermittent self-catheterisation; needs enemata or manual measures to evacuate bowels
4 in need of almost constant catheterisation
5 loss of bladder or bowel function; external or indwelling catheter
6 loss of bowel and bladder function

7 CEREBRAL FUNCTIONS

° DEPRESSION AND EUPHORIA

0 none
1 present: Patient complains of depression or is considered depressed or euphoric by the investigator or significant other.

° Depression and Euphoria are documented on the scoring sheet but are not taken into consideration for FS and EDSS calculation.

DECREASE IN MENTATION

0 none
1 signs only: not apparent to patient and/or significant other
2 mild: Patient and/or significant other report mild changes in mentation. Examples include: impaired ability to follow a rapid course of association and in surveying complex matters; impaired judgement in certain demanding situations; capable of handling routine daily activities, but unable to tolerate additional stressors; intermittently symptomatic even to normal levels of stress; reduced performance; tendency toward negligence due to obliviousness or fatigue.
3 moderate: definite abnormalities on brief mental status testing, but still oriented to person, place and time
4 marked: not oriented in one or two spheres (person, place or time), marked effect on lifestyle
5 dementia, confusion and/or complete disorientation

+FATIGUE

0 none
1 mild: does not usually interfere with daily activities
2 moderate: interferes, but does not limit daily activities for more than 50 %
3 severe: significant limitation in daily activities (> 50 % reduction)

+Because fatigue is difficult to evaluate objectively, in some studies it does not contribute to the Cerebral FS score or EDSS step. Please adhere to the study's specific instructions.

FUNCTIONAL SYSTEM SCORE

0 normal
1 signs only in decrease in mentation; mild fatigue
2 mild decrease in mentation; moderate or severe fatigue
3 moderate decrease in mentation
4 marked decrease in mentation
5 dementia

8 AMBULATION

Unrestricted ambulation means the patient is able to walk a distance without assistance that is regarded as normal, compared with healthy individuals of similar age and physical condition. In this case the EDSS step can be anything between 0 and 5.0, depending on the FS scores.

Fully ambulatory means at least 500 meters of ambulation without assistance, but not unrestricted. The EDSS step can be anything between 2.0 and 5.0, depending on the FS scores. In this case, the pyramidal and/or cerebellar FS must be ≥2 to reflect this „restriction" of ambulation.

If ambulation is < 500 meters, the EDSS step must be ≥ 4.5 depending on the walking ranges provided by the ambulation score (see next page) and combination of FS scores. EDSS steps 5.5 to 8.0 are exclusively defined by the ability to ambulate and type of assistance required, or the ability to use a wheelchair.

If assistance is needed, the definitions of EDSS steps 6.0 or 6.5 include both a description of the type of assistance required when walking and the walking range. Assistance by another person is equivalent to bilateral assistance.

NOTE

The ambulation score represents both a description of walking range and the type of assistance required for ambulation. The score replaces the former use of several checkboxes (paragraph 8 ambulation on the scoring sheet) but does NOT introduce new definitions. The use of wheelchair can now be scored on the scoring sheet.
Please indicate the reported distance and time for the patient in the appropriate field on the scoring sheet, followed by the type of assistance and the walking distance measured during the assessment.

DISTANCE AND TIME REPORTED BY PATIENT

Maximal unassisted walking distance reported by patient (in meters) without rest or assistance and time required to walk max. distance according to patient (in minutes)

ASSISTANCE

0	Without help or assistance (allowing the use of an ankle foot orthotic device, without any other type of assistive device)
1	Unilateral assistance: one stick/crutch/brace
2	Bilateral assistance: two sticks/crutches/braces or assistance by another person
3	Wheelchair

DISTANCE

Measure the distance the patient is able to walk im meters.

Unassisted: observe the patient walking unassisted for a minimum distance of 500 meters and measure the time needed, if possible.

Assisted: observe the patient walking with the assistive device or help by another person for a minimum distance of 130 meters, if possible.

AMBULATION SCORE

0	Unrestricted
1	Fully ambulatory
2	≥ 300 meters, but < 500 meters, without help or assistance (EDSS 4.5 or 5.0)
3	≥ 200 meters, but < 300 meters, without help or assistance (EDSS 5.0)
4	≥ 100 meters, but < 200 meters, without help or assistance (EDSS 5.5)
5	Walking range < 100 meters without assistance (EDSS 6.0)
6	unilateral assistance, ≥ 50 meters (EDSS 6.0)
7	bilateral assistance, ≥ 120 meters (EDSS 6.0)
8	unilateral assistance, < 50 meters (EDSS 6.5)
9	bilateral assistance, ≥ 5 meters, but < 120 meters (EDSS 6.5)
10	Uses wheelchair without help; unable to walk 5 meters even with aid, essentially restricted to wheelchair; wheels self and transfers alone; up and about in wheelchair some 12 hours a day (EDSS 7.0)
11	Uses wheelchair with help; unable to take more than a few steps; restricted to wheelchair; may need some help in transferring and in wheeling self (EDSS 7.5)
12	essentially restricted to bed or chair or perambulated in wheelchair, but out of bed most of day; retains many self-care functions; generally has effective use of arms (EDSS 8.0)

9 EXPANDED DISABILITY STATUS SCALE

0	normal neurological exam (all FS grade 0)
1.0	no disability, minimal signs in one FS (one FS grade 1)
1.5	no disability, minimal signs in more than one FS (more than one FS grade 1)
2.0	minimal disability in one FS (one FS grade 2, others 0 or 1)
2.5	minimal disability in two FS (two FS grade 2, others 0 or 1)
3.0	moderate disability in one FS (one FS grade 3, others 0 or 1) though fully ambulatory; or mild disability in three or four FS (three/four FS grade 2, others 0 or 1) though fully ambulatory
3.5	fully ambulatory but with moderate disability in one FS (one FS grade 3) and mild disability in one or two FS (one/two FS grade 2) and others 0 or 1; or fully ambulatory with two FS grade 3 (others 0 or 1);or fully ambulatory with five FS grade 2 (others 0 or 1)
4.0	ambulatory without aid or rest for ≥500 meters; up and about some 12 hours a day despite relatively severe disability consisting of one FS grade 4 (others 0 or 1) or combinations of lesser grades exceeding limits of previous steps
4.5	ambulatory without aid or rest for ≥300 meters; up and about much of the day, characterised by relatively severe disability usually consisting of one FS grade 4 and combination of lesser grades exceeding limits of previous steps
5.0	ambulatory without aid or rest for ≥200 meters (usual FS equivalents include at least one FS grade 5, or combinations of lesser grades usually exceeding specifications for step 4.5)
5.5	ambulatory without aid or rest for ≥100 meters
6.0	unilateral assistance (cane or crutch) required to walk at least 100 meters with or without resting (see chapter 8, Ambulation)
6.5	constant bilateral assistance (canes or crutches) required to walk at least 20 meters without resting (see chapter 8, Ambulation)
7.0	unable to walk 5 meters even with aid, essentially restricted to wheelchair; wheels self and transfers alone; up and about in wheelchair some 12 hours a day
7.5	unable to take more than a few steps; restricted to wheelchair; may need some help in transferring and in wheeling self
8.0	essentially restricted to bed or chair or perambulated in wheelchair, but out of bed most of day; retains many self-care functions; generally has effective use of arms
8.5	essentially restricted to bed much of the day; has some effective use of arm(s); retains some self-care functions
9.0	helpless bed patient; can communicate and eat
9.5	totally helpless bed patient; unable to communicate effectively or eat/swallow
10	death due to MS

neurostatus scoring

Scoring Sheet for a standardised, quantified neurological examination and assessment of Kurtzke's Functional Systems and Expanded Disability Status Scale in Multiple Sclerosis

STUDY NAME

PERSONAL INFORMATION

Patient	
Date of Birth (04-Jun-1980)	__-___-____
Centre Nr/Country	
Name of EDSS rater	
Date of Examination	__-___-20__

SYNOPSIS

1. Visual	[1]
2. Brainstem	
3. Pyramidal	
4. Cerebellar	
5. Sensory	
6. Bowel/Bladder	[1]
7. Cerebral	

Ambulation Score

EDSS Step

Signature

1. VISUAL (OPTIC) FUNCTIONS

OPTIC FUNCTIONS	OD	OS
Visual acuity ☐ CC ☐ SC		
Visual fields		
Scotoma		
* Disc pallor		
FUNCTIONAL SYSTEM SCORE	→	[1]

2. BRAINSTEM FUNCTIONS

CRANIAL NERVE EXAMINATION	
Extraocular movements (EOM) impairment	
Nystagmus	
Trigeminal damage	
Facial weakness	
Hearing loss	
Dysarthria	
Dysphagia	
Other cranial nerve functions	
FUNCTIONAL SYSTEM SCORE	

3. PYRAMIDAL FUNCTIONS

REFLEXES	R	><	L
Biceps			
Triceps			
Brachioradialis			
Knee			
Ankle			
Plantar response			
Cutaneous reflexes			
* Palmomental reflex			

LIMB STRENGTH	R	L
Deltoid		
Biceps		
Triceps		
Wrist/finger flexors		
Wrist/finger extensors		
Hip flexors		
Knee flexors		
Knee extensors		
Plantar flexion (feet/toes)		
Dorsiflexion (feet/toes)		
* Position test UE, pronation		
* Position test UE, downward drift		
* Position test LE, sinking		
* Able to lift only one leg at a time (grade in °)	°	°
* Walking on heels		
* Walking on toes		
* Hopping on one foot		

SPASTICITY		
Arms		
Legs		
Gait		
OVERALL MOTOR PERFORMANCE		
FUNCTIONAL SYSTEM SCORE		

CC = corrected
SC = without correction
* = optional part of the examination
1 = converted FS Score

4. CEREBELLAR FUNCTIONS

CEREBELLAR EXAMINATION	R	L
Head tremor		
Truncal ataxia		
Tremor/dysmetria UE		
Tremor/dysmetria LE		
Rapid alternating movements UE impairment		
Rapid alternating movements LE impairment		
Tandem walking		
Gait ataxia		
Romberg test		
Other, e. g. rebound		
FUNCTIONAL SYSTEM SCORE		

5. SENSORY FUNCTIONS

SENSORY EXAMINATION	R	L
Superficial sensation UE		
Superficial sensation trunk		
Superficial sensation LE		
Vibration sense UE		
Vibration sense LE		
Position sense UE		
Position sense LE		
* Lhermitte's sign		
* Paraesthesiae UE		
* Paraesthesiae trunk		
* Paraesthesiae LE		
FUNCTIONAL SYSTEM SCORE		

6. BOWEL/ BLADDER FUNCTIONS

Urinary hesitancy/retention	
Urinary urgency/incontinence	
Bladder catheterisation	
Bowel dysfunction	
* Sexual dysfunction	
FUNCTIONAL SYSTEM SCORE	→ [1]

7. CEREBRAL FUNCTIONS

MENTAL STATUS EXAMINATION	
° Depression	
° Euphoria	
Decrease in mentation	
+ Fatigue	
FUNCTIONAL SYSTEM SCORE	

AMBULATION

Distance reported by patient (in meters)	
Time reported by patient (in minutes)	
Assistance	
Distance measured (in meters)	
AMBULATION SCORE	

* = optional part of the examination
1 = converted FS Score
° Depression and Euphoria are not taken into consideration for FS and EDSS calculation.
+ Because fatigue is difficult to evaluate objectively, in some studies it does not contribute to the Cerebral FS score or EDSS step. Please adhere to the study's specific instructions.

UE = upper extremities
LE = lower extremities

Standardised Neurological Examination and Assessment of Kurtzke's Functional Systems and Expanded Disability Status Scale
Slightly modified from J.F. Kurtzke, Neurology 1983;33,1444-52
©2011 Ludwig Kappos, MD, Neurology, University Hospital Basel, 4031 Basel, Switzerland; Version 04/10.2

neurostatus.net

Independent Internet Platform for training and certification of physicians participating in projects that use a standardized, quantified neurological examinationand Kurtzke's Functional Systems and Expanded Disability Status Scale in Multiple Sclerosis

neurostatus Training

Interactive Training DVD-ROM for a standardised, quantified neurological examination and assessment of Kurtzke's Functional Systems and Expanded Disability Status Scale in Multiple Sclerosis

neurostatus e-Test

Interactive Test and Certification Tool for a standardised, quantified neurological examination and assessment of Kurtzke's Functional Systems and Expanded Disability Status Scale in Multiple Sclerosis

neurostatus Forum

Forum for a standardised, quantified neurological examination and assessment of Kurtzke's Functional Systems and Expanded Disability Status Scale in Multiple Sclerosis

www.neurostatus.net

Appendix 5 Pain NRS

CD-IA-MEDI-551-1155
Pain Assessment

Subject Name: ________________ Subject Number: ______________

Visit Date: ______________________

Visit description: ***(please check as applicable)*:**

Randomized Controlled Period:
V2 ☐ V5 ☐ V6 ☐ V7 ☐ V8☐ V9 ☐ V10 ☐ EDV ☐

Open-label Period:
V3 ☐ V4 ☐ V5 ☐ >V5 OLP visit ☐ EDV ☐

Assessment visit: ☐

Attack Follow-up visit: ☐

Please rate your pain for each of the following areas of your body by circling the one number that best describes your **worst pain intensity in the past 24 hours** (where 0 = no pain and 10 = worst pain imaginable)

1. In the past 24 hours, at its worst, how bad was the pain in your eyes?

No pain										Worst pain imaginable
0	1	2	3	4	5	6	7	8	9	10

2. In the past 24 hours, at its worst, how bad was the pain in your legs?

No pain										Worst pain imaginable
0	1	2	3	4	5	6	7	8	9	10

Page 1 of 2
Am 1 FINAL Version dated: 04 August 2014

CD-IA-MEDI-551-1155
Pain Assessment

Subject Name: _______________ **Subject Number:** _______________

3. In the past 24 hours, at its worst, how bad was the pain in your arms?

No pain										Worst pain imaginable
0	1	2	3	4	5	6	7	8	9	10

4. In the past 24 hours, at its worst, how bad was the pain in your upper back?

No pain										Worst pain imaginable
0	1	2	3	4	5	6	7	8	9	10

5. In the past 24 hours, at its worst, how bad was the pain in your lower back?

No pain										Worst pain imaginable
0	1	2	3	4	5	6	7	8	9	10

Page 2 of 2
Am 1 FINAL Version dated: 04 August 2014

Appendix 6 Short Form-36v2

Your Health and Well-Being

This survey asks for your views about your health. This information will help keep track of how you feel and how well you are able to do your usual activities. ***Thank you for completing this survey!***

For each of the following questions, please mark an ☒ in the one box that best describes your answer.

1. In general, would you say your health is:

Excellent	Very good	Good	Fair	Poor
☐ 1	☐ 2	☐ 3	☐ 4	☐ 5

2. <u>Compared to one year ago</u>, how would you rate your health in general <u>now</u>?

Much better now than one year ago	Somewhat better now than one year ago	About the same as one year ago	Somewhat worse now than one year ago	Much worse now than one year ago
☐ 1	☐ 2	☐ 3	☐ 4	☐ 5

SF-36v2® Health Survey © 1992, 1996, 2000 Medical Outcomes Trust and QualityMetric Incorporated. All rights reserved.
SF-36® is a registered trademark of Medical Outcomes Trust.
(SF-36v2® Health Survey Standard, United States (English))

SAMPLE

3. The following questions are about activities you might do during a typical day. Does <u>your health now limit you</u> in these activities? If so, how much?

		Yes, limited a lot	Yes, limited a little	No, not limited at all
a	Vigorous activities, such as running, lifting heavy objects, participating in strenuous sports	☐ 1	☐ 2	☐ 3
b	Moderate activities, such as moving a table, pushing a vacuum cleaner, bowling, or playing golf	☐ 1	☐ 2	☐ 3
c	Lifting or carrying groceries	☐ 1	☐ 2	☐ 3
d	Climbing several flights of stairs	☐ 1	☐ 2	☐ 3
e	Climbing one flight of stairs	☐ 1	☐ 2	☐ 3
f	Bending, kneeling, or stooping	☐ 1	☐ 2	☐ 3
g	Walking more than a mile	☐ 1	☐ 2	☐ 3
h	Walking several hundred yards	☐ 1	☐ 2	☐ 3
i	Walking one hundred yards	☐ 1	☐ 2	☐ 3
j	Bathing or dressing yourself	☐ 1	☐ 2	☐ 3

SAMPLE

SF-36v2® Health Survey © 1992, 1996, 2000 Medical Outcomes Trust and QualityMetric Incorporated. All rights reserved.
SF-36® is a registered trademark of Medical Outcomes Trust.
(SF-36v2® Health Survey Standard, United States (English))

4. During the past 4 weeks, how much of the time have you had any of the following problems with your work or other regular daily activities as a result of your physical health?

	All of the time	Most of the time	Some of the time	A little of the time	None of the time
a Cut down on the amount of time you spent on work or other activities	☐ 1	☐ 2	☐ 3	☐ 4	☐ 5
b Accomplished less than you would like	☐ 1	☐ 2	☐ 3	☐ 4	☐ 5
c Were limited in the kind of work or other activities	☐ 1	☐ 2	☐ 3	☐ 4	☐ 5
d Had difficulty performing the work or other activities (for example, it took extra effort)	☐ 1	☐ 2	☐ 3	☐ 4	☐ 5

5. During the past 4 weeks, how much of the time have you had any of the following problems with your work or other regular daily activities as a result of any emotional problems (such as feeling depressed or anxious)?

	All of the time	Most of the time	Some of the time	A little of the time	None of the time
a Cut down on the amount of time you spent on work or other activities	☐ 1	☐ 2	☐ 3	☐ 4	☐ 5
b Accomplished less than you would like	☐ 1	☐ 2	☐ 3	☐ 4	☐ 5
c Did work or other activities less carefully than usual	☐ 1	☐ 2	☐ 3	☐ 4	☐ 5

SAMPLE

SF-36v2® Health Survey © 1992, 1996, 2000 Medical Outcomes Trust and QualityMetric Incorporated. All rights reserved.
SF-36® is a registered trademark of Medical Outcomes Trust.
(SF-36v2® Health Survey Standard, United States (English))

6. During the past 4 weeks, to what extent has your physical health or emotional problems interfered with your normal social activities with family, friends, neighbors, or groups?

Not at all	Slightly	Moderately	Quite a bit	Extremely
☐ 1	☐ 2	☐ 3	☐ 4	☐ 5

7. How much bodily pain have you had during the past 4 weeks?

None	Very mild	Mild	Moderate	Severe	Very severe
☐ 1	☐ 2	☐ 3	☐ 4	☐ 5	☐ 6

8. During the past 4 weeks, how much did pain interfere with your normal work (including both work outside the home and housework)?

Not at all	A little bit	Moderately	Quite a bit	Extremely
☐ 1	☐ 2	☐ 3	☐ 4	☐ 5

SAMPLE

SF-36v2® Health Survey © 1992, 1996, 2000 Medical Outcomes Trust and QualityMetric Incorporated. All rights reserved.
SF-36® is a registered trademark of Medical Outcomes Trust.
(SF-36v2® Health Survey Standard, United States (English))

9. These questions are about how you feel and how things have been with you <u>during the past 4 weeks</u>. For each question, please give the one answer that comes closest to the way you have been feeling. How much of the time during the <u>past 4 weeks</u>…

	All of the time	Most of the time	Some of the time	A little of the time	None of the time
a Did you feel full of life?	☐ 1	☐ 2	☐ 3	☐ 4	☐ 5
b Have you been very nervous?	☐ 1	☐ 2	☐ 3	☐ 4	☐ 5
c Have you felt so down in the dumps that nothing could cheer you up?	☐ 1	☐ 2	☐ 3	☐ 4	☐ 5
d Have you felt calm and peaceful?	☐ 1	☐ 2	☐ 3	☐ 4	☐ 5
e Did you have a lot of energy?	☐ 1	☐ 2	☐ 3	☐ 4	☐ 5
f Have you felt downhearted and depressed?	☐ 1	☐ 2	☐ 3	☐ 4	☐ 5
g Did you feel worn out?	☐ 1	☐ 2	☐ 3	☐ 4	☐ 5
h Have you been happy?	☐ 1	☐ 2	☐ 3	☐ 4	☐ 5
i Did you feel tired?	☐ 1	☐ 2	☐ 3	☐ 4	☐ 5

10. During the <u>past 4 weeks</u>, how much of the time has your <u>physical health or emotional problems</u> interfered with your social activities (like visiting with friends, relatives, etc.)?

All of the time	Most of the time	Some of the time	A little of the time	None of the time
☐ 1	☐ 2	☐ 3	☐ 4	☐ 5

SAMPLE

SF-36v2® Health Survey © 1992, 1996, 2000 Medical Outcomes Trust and QualityMetric Incorporated. All rights reserved.
SF-36® is a registered trademark of Medical Outcomes Trust.
(SF-36v2® Health Survey Standard, United States (English))

11. How TRUE or FALSE is <u>each</u> of the following statements for you?

	Definitely true	Mostly true	Don't know	Mostly false	Definitely false
a I seem to get sick a little easier than other people	☐ 1	☐ 2	☐ 3	☐ 4	☐ 5
b I am as healthy as anybody I know	☐ 1	☐ 2	☐ 3	☐ 4	☐ 5
c I expect my health to get worse	☐ 1	☐ 2	☐ 3	☐ 4	☐ 5
d My health is excellent	☐ 1	☐ 2	☐ 3	☐ 4	☐ 5

Thank you for completing these questions!

SAMPLE

SF-36v2® Health Survey © 1992, 1996, 2000 Medical Outcomes Trust and QualityMetric Incorporated. All rights reserved.
SF-36® is a registered trademark of Medical Outcomes Trust.
(SF-36v2® Health Survey Standard, United States (English))

Appendix 7 Columbia-Suicide Severity Rating Scale: Baseline/Screening Version

COLUMBIA-SUICIDE SEVERITY RATING SCALE (C-SSRS)

Baseline/Screening Version

Version 1/14/09

Posner, K.; Brent, D.; Lucas, C.; Gould, M.; Stanley, B.; Brown, G.; Fisher, P.; Zelazny, J.; Burke, A.; Oquendo, M.; Mann, J.

Disclaimer:

This scale is intended to be used by individuals who have received training in its administration. The questions contained in the Columbia-Suicide Severity Rating Scale are suggested probes. Ultimately, the determination of the presence of suicidal ideation or behavior depends on the judgment of the individual administering the scale.

Definitions of behavioral suicidal events in this scale are based on those used in **The Columbia Suicide History Form**, *developed by John Mann, MD and Maria Oquendo, MD, Conte Center for the Neuroscience of Mental Disorders (CCNMD), New York State Psychiatric Institute, 1051 Riverside Drive, New York, NY, 10032. (Oquendo M. A., Halberstam B. & Mann J. J., Risk factors for suicidal behavior: utility and limitations of research instruments. In M.B. First [Ed.]* Standardized Evaluation in Clinical Practice, *pp. 103 -130, 2003.)*

For reprints of the C-SSRS contact Kelly Posner, Ph.D., New York State Psychiatric Institute, 1051 Riverside Drive, New York, New York, 10032; inquiries and training requirements contact posnerk@childpsych.columbia.edu

© 2008 The Research Foundation for Mental Hygiene, Inc.

SUICIDAL IDEATION		
Ask questions 1 and 2. If both are negative, proceed to "Suicidal Behavior" section. If the answer to question 2 is "yes", ask questions 3, 4 and 5. If the answer to question 1 and/or 2 is "yes", complete "Intensity of Ideation" section below.	**Lifetime: Time He/She Felt Most Suicidal**	**Past __ Months**
**1. Wish to be Dead**<br>Subject endorses thoughts about a wish to be dead or not alive anymore, or wish to fall asleep and not wake up.<br>***Have you wished you were dead or wished you could go to sleep and not wake up?***<br><br>If yes, describe:	Yes No<br>☐ ☐	Yes No<br>☐ ☐
**2. Non-Specific Active Suicidal Thoughts**<br>General non-specific thoughts of wanting to end one's life/commit suicide (e.g., *"I've thought about killing myself"*) without thoughts of ways to kill oneself/associated methods, intent, or plan during the assessment period.<br>***Have you actually had any thoughts of killing yourself?***<br><br>If yes, describe:	Yes No<br>☐ ☐	Yes No<br>☐ ☐
**3. Active Suicidal Ideation with Any Methods (Not Plan) without Intent to Act**<br>Subject endorses thoughts of suicide and has thought of at least one method during the assessment period. This is different than a specific plan with time, place or method details worked out (e.g. thought of method to kill self but not a specific plan). Includes person who would say, *"I thought about taking an overdose but I never made a specific plan as to when, where or how I would actually do it…and I would never go through with it."*<br>***Have you been thinking about how you might do this?***<br><br>If yes, describe:	Yes No<br>☐ ☐	Yes No<br>☐ ☐
**4. Active Suicidal Ideation with Some Intent to Act, without Specific Plan**<br>Active suicidal thoughts of killing oneself and subject reports having some intent to act on such thoughts, as opposed to *"I have the thoughts but I definitely will not do anything about them."*<br>***Have you had these thoughts and had some intention of acting on them?***<br><br>If yes, describe:	Yes No<br>☐ ☐	Yes No<br>☐ ☐
**5. Active Suicidal Ideation with Specific Plan and Intent**<br>Thoughts of killing oneself with details of plan fully or partially worked out and subject has some intent to carry it out.<br>***Have you started to work out or worked out the details of how to kill yourself? Do you intend to carry out this plan?***<br><br>If yes, describe:	Yes No<br>☐ ☐	Yes No<br>☐ ☐
INTENSITY OF IDEATION		
The following features should be rated with respect to the most severe type of ideation (i.e., 1-5 from above, with 1 being the least severe and 5 being the most severe). Ask about time he/she was feeling the most suicidal.<br><br>Lifetime - ***Most Severe Ideation:*** _______ (***Type # (1-5)***) ____________________ (***Description of Ideation***)<br><br>Past X Months - ***Most Severe Ideation:*** _______ (***Type # (1-5)***) ____________________ (***Description of Ideation***)	Most Severe	Most Severe
**Frequency**<br>***How many times have you had these thoughts?***<br>(1) Less than once a week (2) Once a week (3) 2-5 times in week (4) Daily or almost daily (5) Many times each day	——	——
**Duration**<br>***When you have the thoughts how long do they last?***<br>(1) Fleeting - few seconds or minutes<br>(2) Less than 1 hour/some of the time<br>(3) 1-4 hours/a lot of time<br>(4) 4-8 hours/most of day<br>(5) More than 8 hours/persistent or continuous	——	——
**Controllability**<br>***Could/can you stop thinking about killing yourself or wanting to die if you want to?***<br>(1) Easily able to control thoughts<br>(2) Can control thoughts with little difficulty<br>(3) Can control thoughts with some difficulty<br>(4) Can control thoughts with a lot of difficulty<br>(5) Unable to control thoughts<br>(0) Does not attempt to control thoughts	——	——
**Deterrents**<br>***Are there things - anyone or anything (e.g., family, religion, pain of death) - that stopped you from wanting to die or acting on thoughts of committing suicide?***<br>(1) Deterrents definitely stopped you from attempting suicide<br>(2) Deterrents probably stopped you<br>(3) Uncertain that deterrents stopped you<br>(4) Deterrents most likely did not stop you<br>(5) Deterrents definitely did not stop you<br>(0) Does not apply	——	——
**Reasons for Ideation**<br>***What sort of reasons did you have for thinking about wanting to die or killing yourself? Was it to end the pain or stop the way you were feeling (in other words you couldn't go on living with this pain or how you were feeling) or was it to get attention, revenge or a reaction from others? Or both?***<br>(1) Completely to get attention, revenge or a reaction from others<br>(2) Mostly to get attention, revenge or a reaction from others<br>(3) Equally to get attention, revenge or a reaction from others and to end/stop the pain<br>(4) Mostly to end or stop the pain (you couldn't go on living with the pain or how you were feeling)<br>(5) Completely to end or stop the pain (you couldn't go on living with the pain or how you were feeling)<br>(0) Does not apply	——	——

Version 1/14/09

SUICIDAL BEHAVIOR *(Check all that apply, so long as these are separate events; must ask about all types)*	**Lifetime**	**Past __ Years**
**Actual Attempt:** A potentially self-injurious act committed with at least some wish to die, *as a result of act*. Behavior was in part thought of as method to kill oneself. Intent does not have to be 100%. If there is ***any*** intent/desire to die associated with the act, then it can be considered an actual suicide attempt. ***There does not have to be any injury or harm***, just the potential for injury or harm. If person pulls trigger while gun is in mouth but gun is broken so no injury results, this is considered an attempt.<br>Inferring Intent: Even if an individual denies intent/wish to die, it may be inferred clinically from the behavior or circumstances. For example, a highly lethal act that is clearly not an accident so no other intent but suicide can be inferred (e.g., gunshot to head, jumping from window of a high floor/story). Also, if someone denies intent to die, but they thought that what they did could be lethal, intent may be inferred.<br>***Have you made a suicide attempt?***<br>***Have you done anything to harm yourself?***<br>***Have you done anything dangerous where you could have died?***<br>***What did you do?***<br>***Did you______ as a way to end your life?***<br>***Did you want to die (even a little) when you_____?***<br>***Were you trying to end your life when you _____?***<br>***Or Did you think it was possible you could have died from_____?***<br>***Or did you do it purely for other reasons / without ANY intention of killing yourself (like to relieve stress, feel better, get sympathy, or get something else to happen)?*** (Self-Injurious Behavior without suicidal intent)<br>If yes, describe:	**Yes No**<br>☐ ☐<br>Total # of Attempts<br>_____	**Yes No**<br>☐ ☐<br>Total # of Attempts<br>_____
**Has subject engaged in Non-Suicidal Self-Injurious Behavior?**	**Yes No**<br>☐ ☐	**Yes No**<br>☐ ☐
**Interrupted Attempt:** When the person is interrupted (by an outside circumstance) from starting the potentially self-injurious act *(if not for that, actual attempt would have occurred).*<br>Overdose: Person has pills in hand but is stopped from ingesting. Once they ingest any pills, this becomes an attempt rather than an interrupted attempt. Shooting: Person has gun pointed toward self, gun is taken away by someone else, or is somehow prevented from pulling trigger. Once they pull the trigger, even if the gun fails to fire, it is an attempt. Jumping: Person is poised to jump, is grabbed and taken down from ledge. Hanging: Person has noose around neck but has not yet started to hang - is stopped from doing so.<br>***Has there been a time when you started to do something to end your life but someone or something stopped you before you actually did anything?***<br>If yes, describe:	**Yes No**<br>☐ ☐<br>Total # of interrupted<br>_____	**Yes No**<br>☐ ☐<br>Total # of interrupted<br>_____
**Aborted Attempt:** When person begins to take steps toward making a suicide attempt, but stops themselves before they actually have engaged in any self-destructive behavior. Examples are similar to interrupted attempts, except that the individual stops him/herself, instead of being stopped by something else.<br>***Has there been a time when you started to do something to try to end your life but you stopped yourself before you actually did anything?***<br>If yes, describe:	**Yes No**<br>☐ ☐<br>Total # of aborted<br>_____	**Yes No**<br>☐ ☐<br>Total # of aborted<br>_____
**Preparatory Acts or Behavior:** Acts or preparation towards imminently making a suicide attempt. This can include anything beyond a verbalization or thought, such as assembling a specific method (e.g., buying pills, purchasing a gun) or preparing for one's death by suicide (e.g., giving things away, writing a suicide note).<br>***Have you taken any steps towards making a suicide attempt or preparing to kill yourself (such as collecting pills, getting a gun, giving valuables away or writing a suicide note)?***<br>If yes, describe:	**Yes No**<br>☐ ☐	**Yes No**<br>☐ ☐
**Suicidal Behavior:** Suicidal behavior was present during the assessment period?	**Yes No**<br>☐ ☐	**Yes No**<br>☐ ☐

Answer for Actual Attempts Only	Most Recent Attempt Date:	Most Lethal Attempt Date:	Initial/First Attempt Date:
**Actual Lethality/Medical Damage:**<br>0. No physical damage or very minor physical damage (e.g., surface scratches).<br>1. Minor physical damage (e.g., lethargic speech; first-degree burns; mild bleeding; sprains).<br>2. Moderate physical damage; medical attention needed (e.g., conscious but sleepy, somewhat responsive; second-degree burns; bleeding of major vessel).<br>3. Moderately severe physical damage; *medical* hospitalization and likely intensive care required (e.g., comatose with reflexes intact; third-degree burns less than 20% of body; extensive blood loss but can recover; major fractures).<br>4. Severe physical damage; *medical* hospitalization with intensive care required (e.g., comatose without reflexes; third-degree burns over 20% of body; extensive blood loss with unstable vital signs; major damage to a vital area).<br>5. Death	*Enter Code*<br>______	*Enter Code*<br>______	*Enter Code*<br>______
**Potential Lethality: Only Answer if Actual Lethality=0**<br>Likely lethality of actual attempt if no medical damage (the following examples, while having no actual medical damage, had potential for very serious lethality: put gun in mouth and pulled the trigger but gun fails to fire so no medical damage; laying on train tracks with oncoming train but pulled away before run over).<br>0 = Behavior not likely to result in injury<br>1 = Behavior likely to result in injury but not likely to cause death<br>2 = Behavior likely to result in death despite available medical care	*Enter Code*<br>______	*Enter Code*<br>______	*Enter Code*<br>______

Appendix 8 Columbia-Suicide Severity Rating Scale: Since Last Visit Version

COLUMBIA-SUICIDE SEVERITY RATING SCALE (C-SSRS)

Since Last Visit

Version 1/14/09

Posner, K.; Brent, D.; Lucas, C.; Gould, M.; Stanley, B.; Brown, G.; Fisher, P.; Zelazny, J.; Burke, A.; Oquendo, M.; Mann, J.

Disclaimer:

This scale is intended to be used by individuals who have received training in its administration. The questions contained in the Columbia-Suicide Severity Rating Scale are suggested probes. Ultimately, the determination of the presence of suicidal ideation or behavior depends on the judgment of the individual administering the scale.

Definitions of behavioral suicidal events in this scale are based on those used in **The Columbia Suicide History Form***, developed by John Mann, MD and Maria Oquendo, MD, Conte Center for the Neuroscience of Mental Disorders (CCNMD), New York State Psychiatric Institute, 1051 Riverside Drive, New York, NY, 10032. (Oquendo M. A., Halberstam B. & Mann J. J., Risk factors for suicidal behavior: utility and limitations of research instruments. In M.B. First [Ed.]* Standardized Evaluation in Clinical Practice, *pp. 103 -130, 2003.)*

For reprints of the C-SSRS contact Kelly Posner, Ph.D., New York State Psychiatric Institute, 1051 Riverside Drive, New York, New York, 10032; inquiries and training requirements contact posnerk@nyspi.columbia.edu
© 2008 The Research Foundation for Mental Hygiene, Inc.

SUICIDAL IDEATION	
Ask questions 1 and 2. If both are negative, proceed to "Suicidal Behavior" section. If the answer to question 2 is "yes", ask questions 3, 4 and 5. If the answer to question 1 and/or 2 is "yes", complete "Intensity of Ideation" section below.	Since Last Visit
**1. Wish to be Dead**<br>Subject endorses thoughts about a wish to be dead or not alive anymore, or wish to fall asleep and not wake up.<br>***Have you wished you were dead or wished you could go to sleep and not wake up?***<br><br>If yes, describe:	**Yes** ☐ **No** ☐
**2. Non-Specific Active Suicidal Thoughts**<br>General, non-specific thoughts of wanting to end one's life/commit suicide (e.g., *"I've thought about killing myself"*) without thoughts of ways to kill oneself/associated methods, intent, or plan during the assessment period.<br>***Have you actually had any thoughts of killing yourself?***<br><br>If yes, describe:	**Yes** ☐ **No** ☐
**3. Active Suicidal Ideation with Any Methods (Not Plan) without Intent to Act**<br>Subject endorses thoughts of suicide and has thought of at least one method during the assessment period. This is different than a specific plan with time, place or method details worked out (e.g., thought of method to kill self but not a specific plan). Includes person who would say, *"I thought about taking an overdose but I never made a specific plan as to when, where or how I would actually do it…and I would never go through with it."*<br>***Have you been thinking about how you might do this?***<br><br>If yes, describe:	**Yes** ☐ **No** ☐
**4. Active Suicidal Ideation with Some Intent to Act, without Specific Plan**<br>Active suicidal thoughts of killing oneself and subject reports having some intent to act on such thoughts, as opposed to *"I have the thoughts but I definitely will not do anything about them."*<br>***Have you had these thoughts and had some intention of acting on them?***<br><br>If yes, describe:	**Yes** ☐ **No** ☐
**5. Active Suicidal Ideation with Specific Plan and Intent**<br>Thoughts of killing oneself with details of plan fully or partially worked out and subject has some intent to carry it out.<br>***Have you started to work out or worked out the details of how to kill yourself? Do you intend to carry out this plan?***<br><br>If yes, describe:	**Yes** ☐ **No** ☐

INTENSITY OF IDEATION	
The following features should be rated with respect to the most severe type of ideation (i.e., 1-5 from above, with 1 being the least severe and 5 being the most severe).<br><br>***Most Severe Ideation:*** ____ ________________________________<br>***Type # (1-5)*** ***Description of Ideation***	Most Severe
**Frequency**<br>***How many times have you had these thoughts?***<br>(1) Less than once a week (2) Once a week (3) 2-5 times in week (4) Daily or almost daily (5) Many times each day	____
**Duration**<br>***When you have the thoughts, how long do they last?***<br>(1) Fleeting - few seconds or minutes<br>(2) Less than 1 hour/some of the time<br>(3) 1-4 hours/a lot of time<br>(4) 4-8 hours/most of day<br>(5) More than 8 hours/persistent or continuous	____
**Controllability**<br>***Could/can you stop thinking about killing yourself or wanting to die if you want to?***<br>(1) Easily able to control thoughts<br>(2) Can control thoughts with little difficulty<br>(3) Can control thoughts with some difficulty<br>(4) Can control thoughts with a lot of difficulty<br>(5) Unable to control thoughts<br>(0) Does not attempt to control thoughts	____
**Deterrents**<br>***Are there things - anyone or anything (e.g., family, religion, pain of death) - that stopped you from wanting to die or acting on thoughts of committing suicide?***<br>(1) Deterrents definitely stopped you from attempting suicide<br>(2) Deterrents probably stopped you<br>(3) Uncertain that deterrents stopped you<br>(4) Deterrents most likely did not stop you<br>(5) Deterrents definitely did not stop you<br>(0) Does not apply	____
**Reasons for Ideation**<br>***What sort of reasons did you have for thinking about wanting to die or killing yourself? Was it to end the pain or stop the way you were feeling (in other words you couldn't go on living with this pain or how you were feeling) or was it to get attention, revenge or a reaction from others? Or both?***<br>(1) Completely to get attention, revenge or a reaction from others<br>(2) Mostly to get attention, revenge or a reaction from others<br>(3) Equally to get attention, revenge or a reaction from others and to end/stop the pain<br>(4) Mostly to end or stop the pain (you couldn't go on living with the pain or how you were feeling)<br>(5) Completely to end or stop the pain (you couldn't go on living with the pain or how you were feeling)<br>(0) Does not apply	____

Version 1/14/09

SUICIDAL BEHAVIOR<br>*(Check all that apply, so long as these are separate events; must ask about all types)*	Since Last Visit
**Actual Attempt:**<br>A potentially self-injurious act committed with at least some wish to die, *as a result of act*. Behavior was in part thought of as method to kill oneself. Intent does not have to be 100%. If there is ***any*** intent/desire to die associated with the act, then it can be considered an actual suicide attempt. ***There does not have to be any injury or harm***, just the potential for injury or harm. If person pulls trigger while gun is in mouth but gun is broken so no injury results, this is considered an attempt.<br>Inferring Intent: Even if an individual denies intent/wish to die, it may be inferred clinically from the behavior or circumstances. For example, a highly lethal act that is clearly not an accident so no other intent but suicide can be inferred (e.g., gunshot to head, jumping from window of a high floor/story). Also, if someone denies intent to die, but they thought that what they did could be lethal, intent may be inferred.<br>***Have you made a suicide attempt?***<br>***Have you done anything to harm yourself?***<br>***Have you done anything dangerous where you could have died?***<br>***What did you do?***<br>***Did you______ as a way to end your life?***<br>***Did you want to die (even a little) when you_____?***<br>***Were you trying to end your life when you _____?***<br>***Or did you think it was possible you could have died from_____?***<br>***Or did you do it purely for other reasons / without ANY intention of killing yourself (like to relieve stress, feel better, get sympathy, or get something else to happen)?*** (Self-Injurious Behavior without suicidal intent)<br>If yes, describe:<br><br>**Has subject engaged in Non-Suicidal Self-Injurious Behavior?**	Yes ☐ No ☐<br><br>Total # of Attempts<br>_____<br><br>Yes ☐ No ☐
**Interrupted Attempt:**<br>When the person is interrupted (by an outside circumstance) from starting the potentially self-injurious act *(if not for that, actual attempt would have occurred).*<br>Overdose: Person has pills in hand but is stopped from ingesting. Once they ingest any pills, this becomes an attempt rather than an interrupted attempt. Shooting: Person has gun pointed toward self, gun is taken away by someone else, or is somehow prevented from pulling trigger. Once they pull the trigger, even if the gun fails to fire, it is an attempt. Jumping: Person is poised to jump, is grabbed and taken down from ledge. Hanging: Person has noose around neck but has not yet started to hang - is stopped from doing so.<br>***Has there been a time when you started to do something to end your life but someone or something stopped you before you actually did anything?***<br>If yes, describe:	Yes ☐ No ☐<br><br>Total # of interrupted<br>_____
**Aborted Attempt:**<br>When person begins to take steps toward making a suicide attempt, but stops themselves before they actually have engaged in any self-destructive behavior. Examples are similar to interrupted attempts, except that the individual stops him/herself, instead of being stopped by something else.<br>***Has there been a time when you started to do something to try to end your life but you stopped yourself before you actually did anything?***<br>If yes, describe:	Yes ☐ No ☐<br><br>Total # of aborted<br>_____
**Preparatory Acts or Behavior:**<br>Acts or preparation towards imminently making a suicide attempt. This can include anything beyond a verbalization or thought, such as assembling a specific method (e.g., buying pills, purchasing a gun) or preparing for one's death by suicide (e.g., giving things away, writing a suicide note).<br>***Have you taken any steps towards making a suicide attempt or preparing to kill yourself (such as collecting pills, getting a gun, giving valuables away or writing a suicide note)?***<br>If yes, describe:	Yes ☐ No ☐
**Suicidal Behavior:**<br>Suicidal behavior was present during the assessment period?	Yes ☐ No ☐
**Suicide:**	Yes ☐ No ☐
Answer for Actual Attempts Only	Most Lethal Attempt Date:
**Actual Lethality/Medical Damage:**<br>0. No physical damage or very minor physical damage (e.g., surface scratches).<br>1. Minor physical damage (e.g., lethargic speech; first-degree burns; mild bleeding; sprains).<br>2. Moderate physical damage; medical attention needed (e.g., conscious but sleepy, somewhat responsive; second-degree burns; bleeding of major vessel).<br>3. Moderately severe physical damage; *medical* hospitalization and likely intensive care required (e.g., comatose with reflexes intact; third-degree burns less than 20% of body; extensive blood loss but can recover; major fractures).<br>4. Severe physical damage; *medical* hospitalization with intensive care required (e.g., comatose without reflexes; third-degree burns over 20% of body; extensive blood loss with unstable vital signs; major damage to a vital area).<br>5. Death	*Enter Code*<br><br>_____
**Potential Lethality: Only Answer if Actual Lethality=0**<br>Likely lethality of actual attempt if no medical damage (the following examples, while having no actual medical damage, had potential for very serious lethality: put gun in mouth and pulled the trigger but gun fails to fire so no medical damage; laying on train tracks with oncoming train but pulled away before run over).<br>0 = Behavior not likely to result in injury<br>1 = Behavior likely to result in injury but not likely to cause death<br>2 = Behavior likely to result in death despite available medical care	*Enter Code*<br><br>_____

Appendix 9 Sample Size Calculation

The proposed study is being planned to detect a target relative reduction of 60% in risk for time (days) from Day 1 to onset of an AC-determined NMO/NMOSD attack on or before Day 197 with 90% power and $\alpha = 0.05$ (two-sided), assuming an unequal randomization ratio of 3:1 to MEDI-551 versus placebo. Using the following formula, a total of 67 AC-determined NMO/NMOSD attacks will be required:

$$E = \left[\frac{z_{1-\alpha/2} + z_{1-\beta}}{ln(HR)}\right]^2 \times \frac{(r+1)^2}{r},$$

where $z_{1-\alpha/2}$ and $z_{1-\beta}$ are $100(1 - \alpha/2)^{th}$ and $100\ (1 - \beta)^{th}$ percentile of a standard normal distribution, $\boldsymbol{r} = 3$ is the randomization ratio and $\boldsymbol{HR}$ = hazard ratio = 0.4.

Assuming hazard rates of 1.5/year and 1.0/year for an NMO/NMOSD attack in the placebo arm for seropositive and seronegative groups, respectively, and using the following formula:

$$N = \frac{E}{\Pr(Fail)},$$

a total of 212 (with an upward adjustment) subjects are expected to be enrolled in this study where

$$\Pr(\text{Fail}) = s_1 P_1 + s_2 P_2$$

is the weighted average of the failure probabilities P_1 and P_2 in seropositive and seronegative cohorts, respectively, with associated stratum specific weights $s_1 = 0.8$ and $s_2 = 0.2$. Recall that subjects will be enrolled to seropositive and seronegative cohorts in an 80:20 ratio. Furthermore, the probability of an attack within i^{th} cohort (i = 1, 2) is given by

$$P_i = (1 - w)p_{i0} + wp_{i1}$$

the weighted average of the failure probabilities p_{i0} and p_{i1} for placebo and MEDI-551 groups, respectively; $p_{i0} = 1 - e^{-\lambda_{i0}F}$, $p_{i1} = 1 - e^{-\lambda_{i1}F}$; F = 197/365 = 0.54 to indicate the 197 days (28 weeks) duration of the controlled portion of the study; note that based on the assumptions for this study, $\lambda_{10} = 1.5, \lambda_{11} = 0.6, \lambda_{20} = 1$ and $\lambda_{21} = 0.4$ and $w = \frac{r}{1+r}. = 0.75$.

Since the primary endpoint will be tested using the Cox proportional model, a simulation approach was used to investigate the power under alternative hypotheses mentioned above. Based on 5,000 simulations, the power for the overall and seropositive populations were observed as 94% and 91%, respectively. Moreover, the total required 67 events (212 subjects) were, on an average, distributed in seropostive and seronegative cohorts as 57 (168 subjects) and 10 (44 subjects) respectively.

The underlying hazard rate of time to an NMO/NMOSD attack in the seropositive placebo group is estimated based on a meta-analytic approach from several open-label cohort studies (see Table 17) in the absence of any placebo-controlled study in this population to date. These

studies consisted of both seropositive and seronegative subjects and hence any finding related to hazard rate derived from these studies can be considered as an underestimation when applied for seropositive subgroup in the current study. Following are the 5 open-label studies considered with a total of 161 subjects:

Table 17 Open-label Cohort Studies in Neuromyelitis Optica

Author	Title
Bedi et al	Impact of rituximab on relapse rate and disability in neuromyelitis optica. Mult Scler. 2011 Oct;17(10):1225-30
Costanzi et al	Costanzi C, Matiello M, Lucchinetti CF, Weinshenker BG, Pittock SJ, Mandrekar J, et al. Azathioprine: tolerability, efficacy, and predictors of benefit in neuromyelitis optica. Neurology. August 16, 2011;77(7):659-66.
Jacob et al	Treatment of neuromyelitis optica with rituximab: retrospective analysis of 25 patients. Muscle Nerve. 2008 Jan;39(1):87-90
Kim et al	Kim SH, Kim W, Li XF, Jung IJ, Kim HJ. Repeated treatment with rituximab based on the assessment of peripheral circulating memory B cells in patients with relapsing neuromyelitis optica over 2 years. Arch Neurol. 2011 Nov;68(11):1412-20.
Pittock et al	Pittock SJ, Lennon VA, McKeon A, Mandrekar J, Weinshenker BG, Lucchinetti CF, O'Toole O, Wingerchuk DM. Eculizumab in AQP4-IgG-positive relapsing neuromyelitis optica spectrum disorders: an open-label pilot study. Lancet Neurol. 2013 Jun;12(6):554-62.

The approximate time (months) between the first 2 pretreatment relapses was visually enumerated from the published figures for each study (Figure 7, Figure 8, Figure 9, Figure 10, and Figure 11). Subjects with second pretreatment relapses occurring within 12 months of the first relapse were considered failures. Hazard rate (assuming exponential distribution) was calculated from each study based on this failure rate. Overall hazard rate for time to second pretreatment relapse was calculated based on the weighted average of individual hazard rates (weight was based on number of failures from each study). The findings from this analysis are summarized in Table 18.

Table 18 Estimated Pretreatment Hazard Rate for Time to Relapse

Study	Number of Subjects	No. of Subjects with Relapse by 1st Year	Percentage of Subjects Relapse Free by 1st Year	Hazard Rate (per year)
Bedi et al, 2011	23	14	39%	0.94
Costanzi et al, 2011	69	48	30.4%	1.2
Jacob et al, 2008	25	21	16%	1.83
Kim et al, Nov 2011	30	19	37%	0.99

Table 18 Estimated Pretreatment Hazard Rate for Time to Relapse

Study	Number of Subjects	No. of Subjects with Relapse by 1st Year	Percentage of Subjects Relapse Free by 1st Year	Hazard Rate (per year)
Pittock et al, 2006	14	12	14.3%	1.94
**Overall**	161	114	29.2%	1.23

The weighted average of the hazard rates was determined as 1.33/year. As none of the cohorts in these studies received pure placebo treatment, the hazard rate for the placebo treatment for the seropositive subgroup in the current study is estimated to be 1.5/year. Conservatively, the hazard rate for the placebo treatment in seronegative subgroup is chosen as 1.0/year.

Figure 7 Neuromyelitis Optica Spectrum of Disorders Relapses Before and After Treatment with Azathioprine for 70 Patients with More Than 12 Months Follow Up

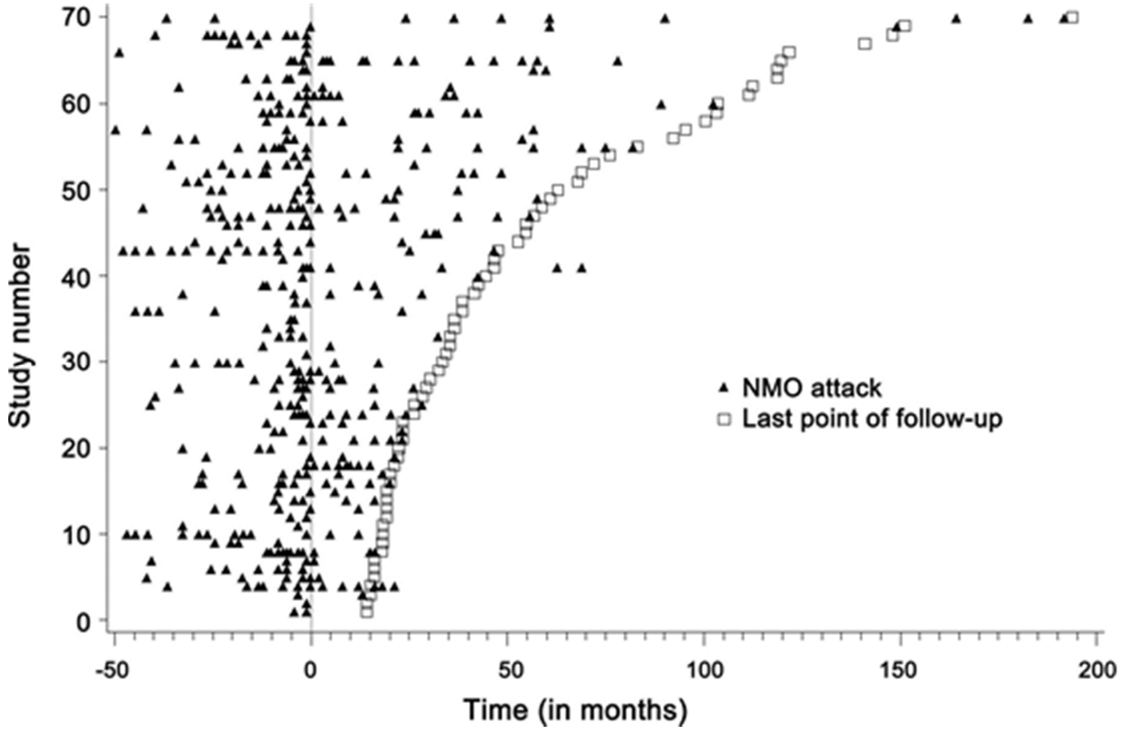

NMO = neuromyelitis optica.

Source: Constanzi et al, 2011.

Figure 8 Attack Frequency Before, During, and After Eculizumab Treatment

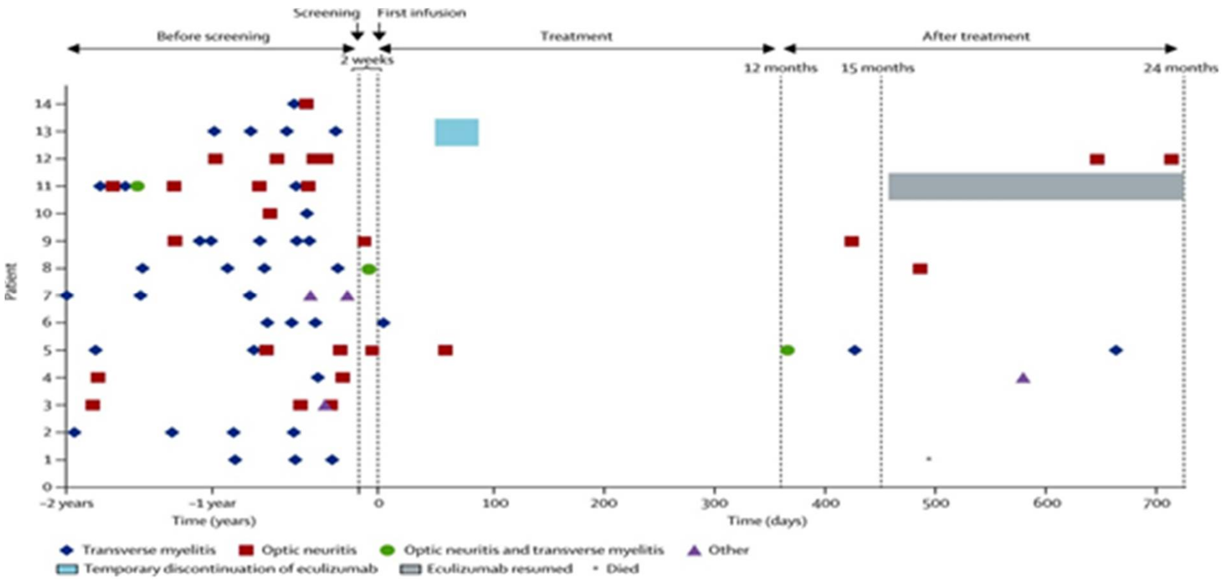

Source: Pittock et al, 2006.

Figure 9 Relapses in Patients with Neuromyelitis Optica Before and After Treatment with Rituximab

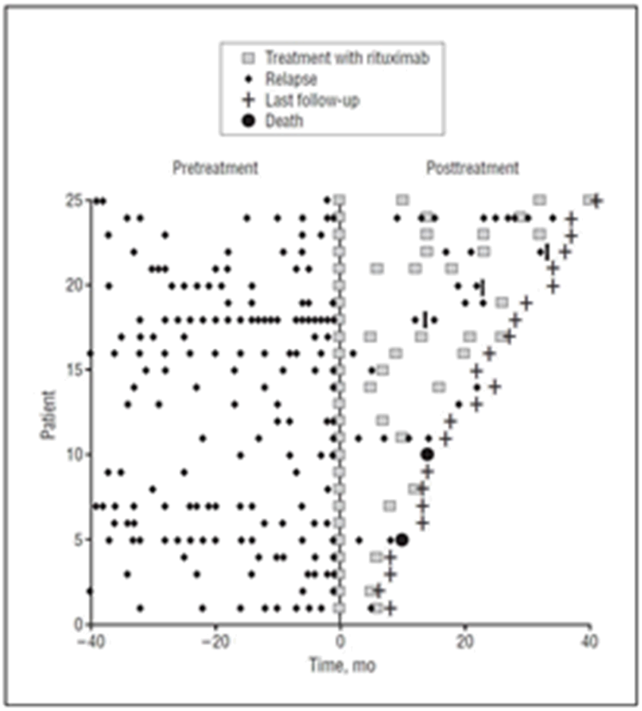

mo = months.

Source: Jacob et al, 2008.

Figure 10 Disease Course of Patients Treated with Rituximab

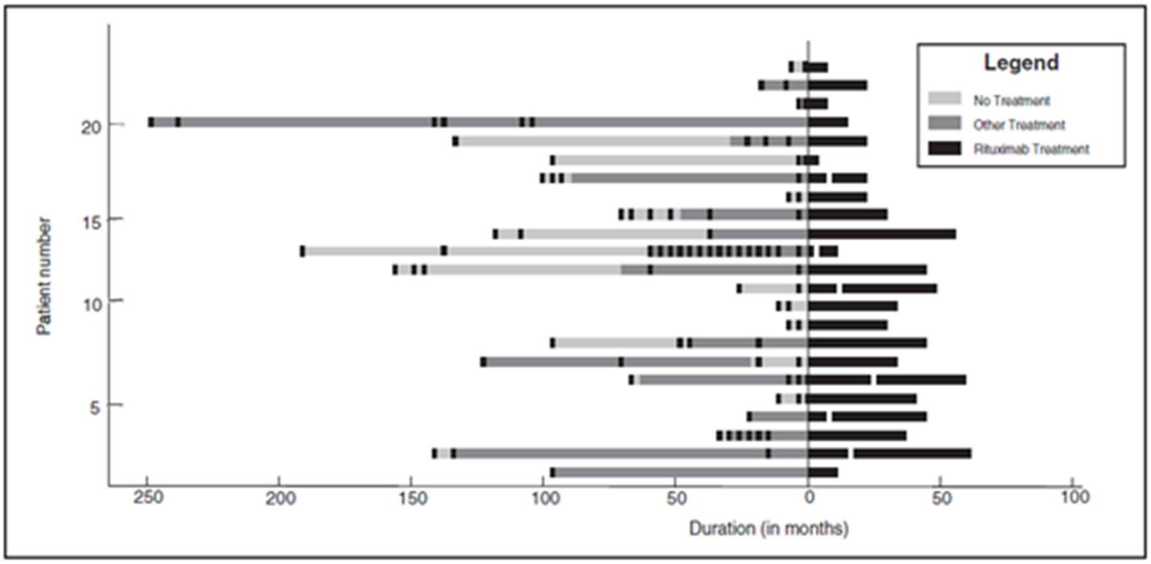

Note: Disease course is depicted longitudinally, with each bar representing one patient. Each bar represents the type of treatment administered at a given time, a light gray bar representing no treatment, a dark gray representing other treatment (immunomodulatory, immunosuppressant) and black representing rituximab treatment. Each relapse is marked along the patient's course by a solid black or white mark. Time 0 on the X axis marks the initiation of rituximab treatment and divides pre- and post-rituximab treatment phases. Numbers on the X and Y axes represent months before/after rituximab initiation and patient number respectively. Most (17 of 23) patients had no recorded clinical relapses, and the remaining six had reduction in relapse frequency with rituximab treatment.

Source: Bedi et al, 2011.

Figure 11 Relapses in Patients with Neuromyelitis Optica Before and After Treatment with Rituximab

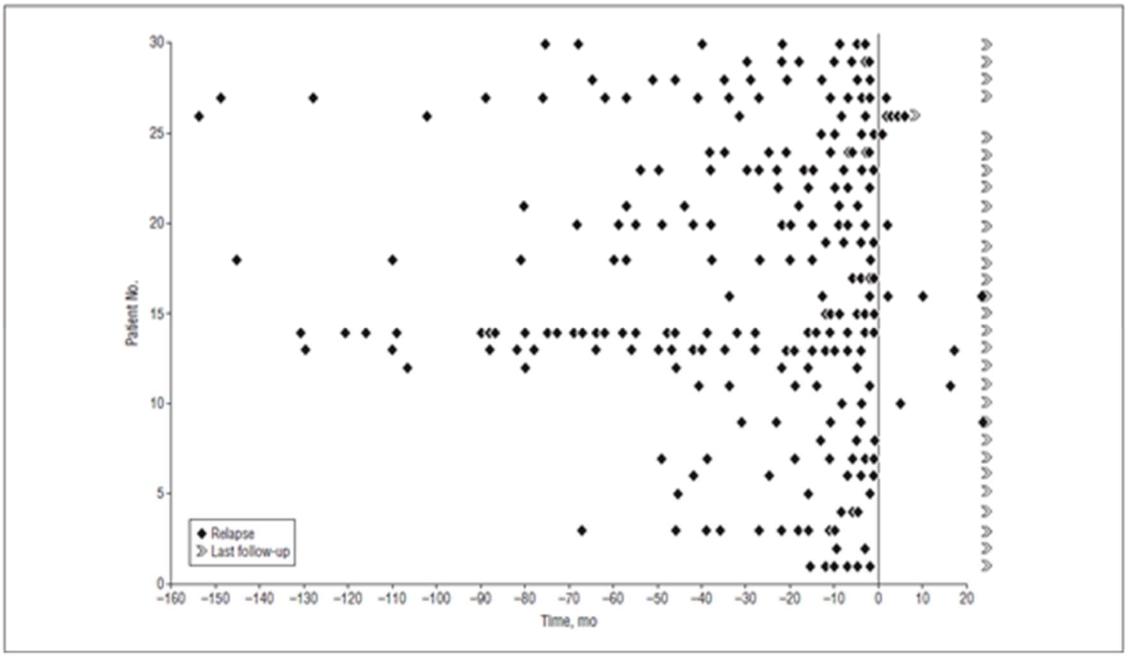

mo = months.

Note: On the x-axis, 0 indicates start date of treatment. Each interrupted line on the y-axis represents a patient.

Source: Kim et al, Nov 2011.

The target treatment effect of 60% has been estimated by comparing similar empirical evidence from Costanzi et al, 2011 (treatment with AZA) and 3 articles from rituximab treatment (Bedi et al, 2011, Jacob et al, 2008, and Kim et al, Nov 2011). For each of these studies, the subjects who were relapse free within the first 12 months of respective treatment were visually identified (Table 19). Based on the survival rate thus obtained, the yearly constant hazard rate was calculated and the weighted average was determined.

Table 19 Calculated Yearly Constant Hazard Rate Based on Relapse-free Subjects in First 12 Months of Treatment

Study	N	No. of Subjects with Relapse by 1st Year	Percentage of Subjects Relapse-free by 1st Year	Hazard Rate (per year)
Bedi et al, 2011	23	4	83%	0.19
Jacob et al, 2008	25	7	72%	0.33
Kim et al, Nov 2011	30	6	80%	0.22
Overall	78	17	78%	0.25

The weighted average of the hazard for time to first relapse based on rituximab studies was determined as 0.258/year. Similarly, the hazard rate based on AZA treatment was calculated (Table 20). Based on this comparison, the relative reduction in risk of relapse was calculated as 57%.

Table 20 Relative Reduction in Risk of Relapse Based on Calculated Hazard Rate From Azathioprine and Rituximab NMO Studies

Agent	N	Events Within 1st Year	Estimated Hazard Rate	Hazard Ratio	Relative Reduction in Risk of Relapse
Rituximab	78	17	0.25	0.43	57%
Azathioprine	70	31	0.58		
Total	148	48	N/A		

N/A = not applicable; NMO = neuromyelitis optica

Since the treatment effect from a placebo-controlled study is not available at this point, the 60% relative reduction in hazard rate was used as a conservative estimate for the target treatment effect. It is recognized that there are a number of caveats behind this approach; such as, the cohorts compared were not randomized and the selection of the patient population from each study may not be a true reflection of the currently proposed study.

As the rate of NMO/NMOSD attacks is not established in the literature, a masked sample size reassessment when 50% of the 212 planned subjects completed the RCP was originally part of the protocol. This reassessment was important to protect against the loss of statistical power in the seropositive cohort as well as the ITT population due to not achieving the required number of events. However, the masked interim sample size reassessment was removed from the protocol as a result of a discussion with the FDA regarding the timing of the sample size reassessment as related to the futility analysis. As the study progresses it becomes possible that the futility analysis will occur prior to the sample size reassessment, which would not allow the sample size reassessment to be performed. Therefore, an agreement was reached with the FDA that the sample size reassessment would be removed from the protocol. Instead the study, which is event driven, will continue to enroll until 67 AC-determined NMOSD attacks have occurred, or until a maximum of 252 patients have been randomized (whichever occurs first).

The maximum number of 252 randomized subjects was determined by analyzing the actual attack rate based on masked data from the first 78 subjects who completed the RCP in this study. The attack status (attack/no attack) of the 78 subjects was randomly sampled with different attack rate which gave an estimate of the number of attacks for the total sample size. This simulation process was repeated 10, 000 times to give a distribution of the number of attacks for the total sample size, from which the probability of observing at least 67 attacks could be estimated. Based on the 78 completed subjects, this procedure showed that with a

sample size of 227 there is a 50% probability of reaching the required 67 AC-determined attacks and with 252 subjects there is a 90% probability of reaching the required 67 AC-determined attacks. The sample size of 252 subjects was selected to give a high degree of confidence that 67 attacks will be observed in this study.

Appendix 10 Details of Futility Analysis

An unmasked interim analysis will be conducted for futility assessment by the DMC when approximately 50% of the total planned AC-determined NMO/NMOSD attacks occur in this study. This will be triggered when approximately 34 AC-determined NMO/NMOSD attacks occur in the ITT population. The assessment of futility will be based on predictive power, which is based on the average conditional power calculated at the observed treatment effect at the time of the interim analysis. The study will be considered futile if the predictive power is < 20% in both the ITT population and the AQP4-IgG seropositive cohort. Figure 12 provides estimated predictive power when observed treatment effect varies from 0 to 90% in relative reduction in risk of an AC-determined NMO/NMOSD attack in the ITT population. The 20% predictive power translates into a treatment effect of approximately 27%. A value of 50% and 90% predictive power will result in 43% and 60% treatment effects, respectively, which are minimally detectable treatment effect and target treatment effect for this study. The actual predictive power will be calculated based on observed data.

Figure 12 Estimated Predictive Power Against Observed Interim Treatment Effect When 50% of AC-determined NMO/NMOSD Attacks are Available

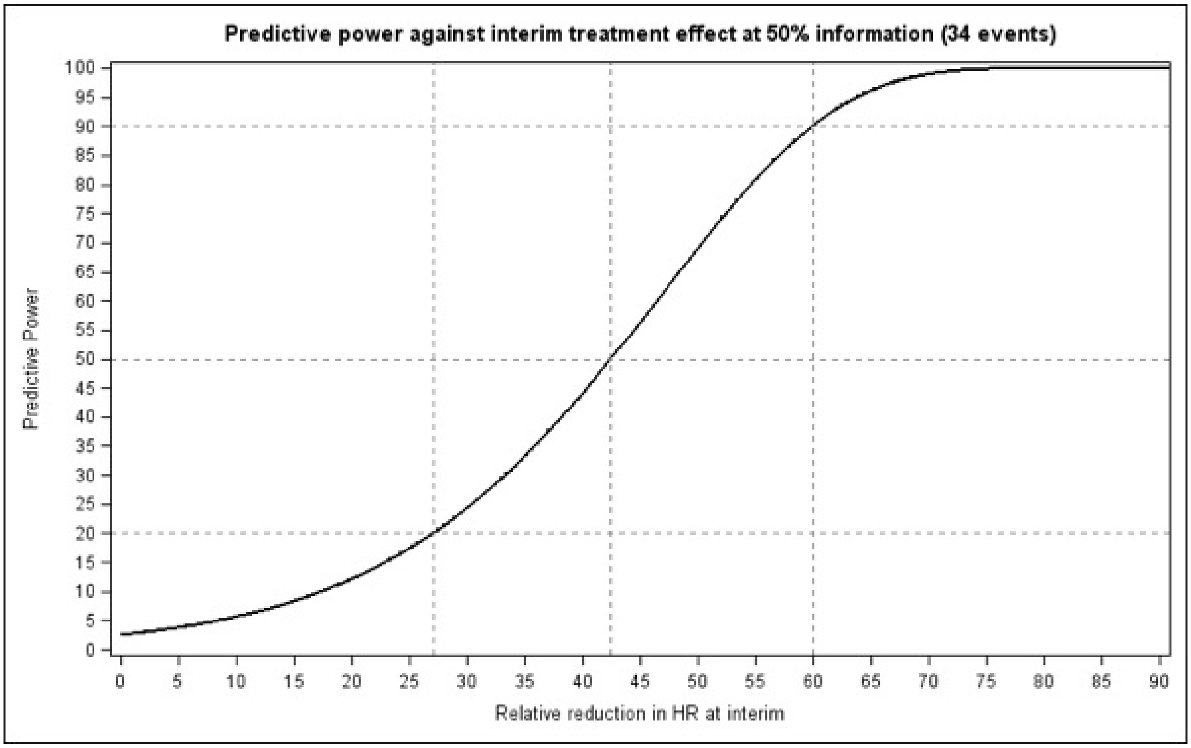

AC = Adjudication Committee; HR = hazard rate; NMO/NMOSD = neuromyelitis optica/neuromyelitis optica spectrum disorders

Appendix 11 Type I Error Control

To establish Type I error control, the primary endpoint, and the 4 secondary endpoints will be denoted by E1 through E5, respectively, as follows:

- E1: Time to AC-determined NMO/NMOSD attack
- E2: Worsening from baseline in EDSS at last visit during the RCP
- E3: Change from baseline in low-contrast visual acuity binocular score measured by low-contrast Landolt C Broken Rings Chart, at last visit during RCP
- E4: Cumulative total active MRI lesions (new Gd-enhancing or new/enlarging T2) during the RCP
- E5: Number of NMO/NMOSD-related in-patient hospitalizations

Based on 2 populations of interest (seropositive and ITT populations), this will result in testing 10 null hypotheses of no treatment effect labeled as follows (Table 21):

Table 21 List of Null Hypotheses Considered Under Multiplicity Adjustment Procedure

Hypothesis	Test
S1	Test E1 in seropositive subjects
S2	Test E2 in seropositive subjects
S3	Test E3 in seropositive subjects
S4	Test E4 in seropositive subjects
S5	Test E5 in seropositive subjects
O1	Test E1 in ITT population
O2	Test E2 in ITT population
O3	Test E3 in ITT population
O4	Test E4 in ITT population
O5	Test E5 in ITT population

The multiplicity adjustment strategy based on Bonferroni-based chain procedure (Bretz et al, 2009; Millen and Dmitrienko, 2011) for testing these 10 hypotheses is defined in Figure 13. Each hypothesis is represented by a rectangular box. The connections among the hypotheses are visualized using arrows. A solid arrow is used to define the decision path after a hypothesis is rejected, eg, the hypothesis O1 is tested if and only if the hypothesis S1 is rejected. The Bonferroni-based chain procedures are characterized by 2 rules:

- The alpha allocation rule specifies the initial distribution of the Type I error rate among the null hypotheses according to the relative importance of the null hypotheses
- The alpha propagation rule determines the process of re-distributing the available Type I error rate among the non-rejected null hypotheses after each rejection

Figure 13 Multiplicity Adjustment Strategy

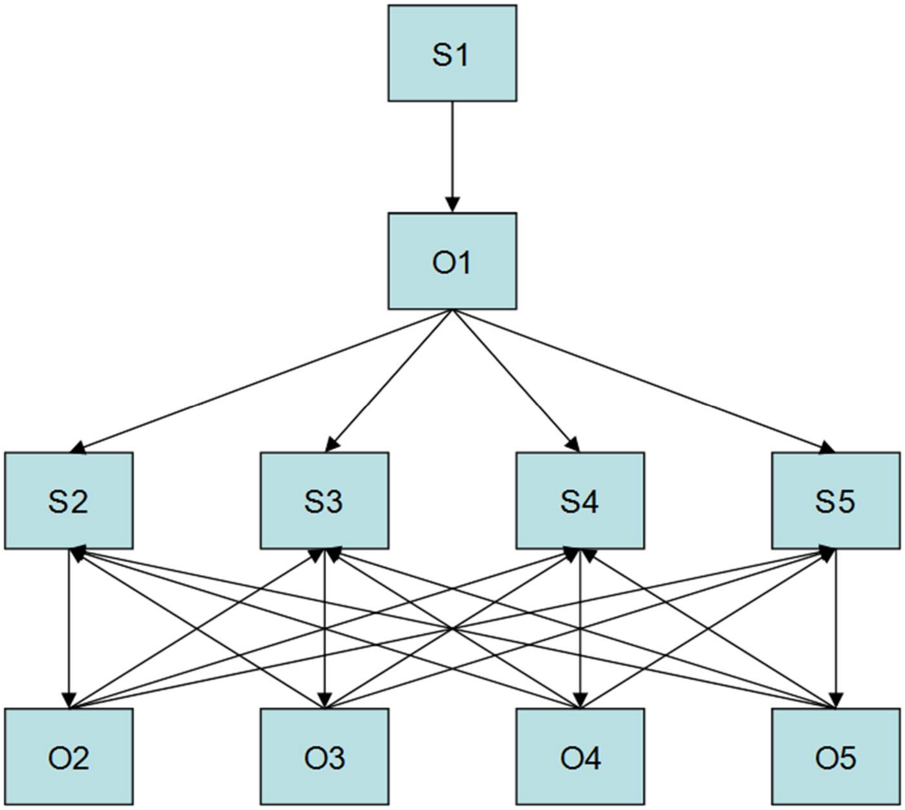

The alpha allocation rule is specified in Table 22. Specifically, the null hypothesis S1 receives the initial weight of 1 (ie, it is tested at the full $\alpha = 0.05$) and the other null hypotheses receive zero weights. The alpha propagation rule is specified in Table 23. The alpha propagation rules are defined using transition parameters, eg, the first row in Table 23 shows that the fraction of the Type I error rate used in testing the null hypothesis S1 will be transferred to the null hypothesis O1 if the null hypothesis S1 is rejected. Similarly, if the null hypothesis O1 is rejected, the available Type I error rate will be split equally among the null hypotheses S2, S3, S4, and S5 (a quarter of the available Type I error rate will be allocated to each null hypothesis). The alpha allocation and alpha propagation rule uniquely define the Bonferroni-based chain procedure and the associated multiplicity-adjusted p-values can be computed using Algorithm 2 given in Bretz et al (2009).

Table 22 Alpha Allocation Rule

Hypothesis	Initial Alpha Allocation
S1	α
S2	0
S3	0
S4	0
S5	0
O1	0
O2	0
O3	0
O4	0
O5	0

Table 23 Alpha Propagation Rule

Hypothesis	S1	S2	S3	S4	S5	O1	O2	O3	O4	O5
S1	0	0	0	0	0	1	0	0	0	0
S2	0	0	0	0	0	0	1	0	0	0
S3	0	0	0	0	0	0	0	1	0	0
S4	0	0	0	0	0	0	0	0	1	0
S5	0	0	0	0	0	0	0	0	0	1
O1	0	1/4	1/4	1/4	1/4	0	0	0	0	0
O2	0	0	1/3	1/3	1/3	0	0	0	0	0
O3	0	1/3	0	1/3	1/3	0	0	0	0	0
O4	0	1/3	1/3	0	1/3	0	0	0	0	0
O5	0	1/3	1/3	1/3	0	0	0	0	0	0

Appendix 12 Assessment and Collection of Adverse Events

If an investigator learns of any SAEs, including death, at any time after a subject has completed the study and he/she considers there is a reasonable possibility that the event is related to MEDI-551, the investigator should notify the Sponsor.

Variables to be Collected for AEs and SAEs

AEs

The following variables will be collected for each AE:

- AE (verbatim)
- Date when the AE started and stopped
- Whether the AE is serious
- Investigator causality rating against the investigational product (yes or no)
- Action taken with regard to investigational product
- If AE caused the subject to be withdrawn from the study (yes or no)
- Outcome

SAEs

For SAEs, other variables will be collected, including treatment given for the event.

It is important to distinguish between serious and severe AEs. Severity is a measure of intensity, whereas, seriousness is defined by the criteria presented in Section 5.2. An AE of severe intensity need not necessarily be considered serious. For example, nausea that persists for several hours may be considered severe nausea, but not an SAE. On the other hand, a stroke that results in only a limited degree of disability may be considered a mild stroke, but it would become an SAE when it satisfies the criteria.

Causality

The investigator will assess the causal relationship between the investigational product and each AE, and answer ‘yes’ or ‘no’ to the question, ‘Do you consider there is a reasonable possibility that the event may have been caused by the investigational product?’

For SAEs, the causal relationship will also be assessed for other medication and study procedures. For SAEs that could be associated with any study procedure, the causal relationship is implied as ‘yes.’

AEs Based on Signs and Symptoms

All AEs spontaneously reported by the subject or care provider, or reported in response to the question, ‘Have you had any health problems since the previous visit/you were last asked?’, or

any AEs that were observed, will be collected and recorded in the eCRF. When collecting AEs, recording the diagnosis is preferred to a listing of signs and symptoms. However, if a diagnosis is known and there are other signs or symptoms that are not generally considered part of the diagnosis, the diagnosis and each sign or symptom should be recorded separately.

AEs Based on Examinations and Tests

The results of protocol-mandated laboratory tests and vital signs will be summarized in the clinical study report. Deterioration in protocol-mandated laboratory test values or vital signs compared to baseline should only be reported as AEs if they fulfill any of the SAE criteria, or are a reason for the discontinuation of treatment with the investigational product.

If deterioration in a laboratory test value or vital sign is associated with clinical signs and symptoms, the clinical sign or symptom should be reported as an AE and the associated laboratory test result or vital sign will be considered additional information. Wherever possible, the reporting investigator should use the clinical term rather than the laboratory term (eg, “anemia” versus “low hemoglobin value”). In the absence of clinical signs or symptoms, clinically-relevant deteriorations in nonmandated parameters should be reported as AE(s).

Any new or aggravated clinically-relevant abnormal medical finding at a physical examination as compared with the baseline assessment will be reported as an AE (baseline data are collected at Visit 2).
